# Supplementary material for: Nitroalkanes as thioacyl equivalents to access thioamides and thiopeptides
Source: Nat Commun. 2023 Aug 2;14:4626. doi: 10.1038/s41467-023-40334-6 (PMC10397191; doi:10.1038/s41467-023-40334-6)
Supplement: Supplementary file 1 — Supplementary Information [file 41467_2023_40334_MOESM1_ESM.pdf]

Supplementary Information

Nitroalkanes as Thioacyl Equivalents to Access Thioamides and Thiopeptides

Xiaonan Wang[1], Silong Xu[1], Yuhai Tang[1], Martin J. Lear[2], Wangxiao He[3] and Jing Li[1]*

[1]School of Chemistry, and Xi'an Key Laboratory of Sustainable Energy Materials Chemistry, Xi'an Jiaotong University, Xi'an 710049, P. R. China

[2]School of Chemistry, University of Lincoln, Brayford Pool, Lincoln LN6 7TS, United Kingdom

[3]The First Affiliated Hospital of Xi'an Jiao Tong University, Xi'an 710061, China

Correspondence and requests for materials should be addressed to J. Li (email: jingli@xjtu.edu.cn)

*Correspondence to: jingli@xjtu.edu.cn

Contents

1. Supplementary Methods 3

1.1 General Information 3

1.2 Synthesis of Starting Materials. 3

1.2.1 Synthesis of electrophilic *S*-sources. 3

1.2.2 Synthesis of nitro compounds. 4

1.2.3 General procedure to prepare peptide nitroalkanes. 8

1.3. General procedure and characterization of thioamide products 11

1.3.1 Optimization of reaction conditions 11

1.3.2 General thioamidation procedure. 11

1.3.3 Structural Characterization of thioamide products 12

1.3.4 General synthesis of thiopeptides from chiral nitro compounds and chiral amino acid esters 29

2 Supplementary Discussion 58

2.1 Possible mechanistic pathways and control experiments 58

2.1.1 Control reactions to interrogate path A. 58

2.1.2 Control reactions to interrogate path B. 59

2.1.3 Control reactions to trap proposed thioacyl intermediate **20** (Path C). 61

2.2 Control reaction to determine the thioacyl nitrate formation. 67

2.2.1 Control reactions to rule out Path a and Path b 67

2.2.2 Control reaction to distinguish Path b and Path c 68

2.2.3 Control reaction to detect the formation of S_3 radical anion 73

3. Supplementary Notes 75

3.1 NMR spectra 75

4. Supplementary References 151

1. Supplementary Methods

1.1 General Information

Unless otherwise stated, all reagents were used as received from commercial suppliers. Reaction progress was monitored by thin layer chromatography (TLC) performed on aluminum plates coated with silica gel F_{254} with 0.2 mm thickness. Chromatograms were visualized by fluorescence quenching with UV light at 254 nm or by staining using potassium permanganate. 1-Nitropropane was purchased from energy-chemical Co. Flash column chromatography was performed using silica gel (200-300 mesh, Merck and Co.). Neat infra-red spectra were recorded using a Perkin-Elmer Spectrum 100 FT-IR spectrometer. Wavenumbers (ν_{max}) are reported in cm^{-1}. Mass spectra were obtained using a Finnigan MAT 8200 or (70 eV) or an Agilent 5973 (70 eV) spectrometer, using electrospray ionization (ESI). All ^{1}H NMR and ^{13}C NMR spectra were recorded using a JEOL JNM AL 400 (400 MHz) at 300K. Chemical shifts are given in parts per million (ppm, δ), referenced to the solvent peak of $CDCl_3$, defined at δ = 7.26 ppm (^{1}H NMR) and δ = 77.16 (^{103}C NMR). Coupling constants are quoted in Hz (*J*). ^{1}H NMR splitting patterns were designated as singlet (*s*), doublet (*d*), triplet (*t*), quartet (*q*), pentet (*p*). Splitting patterns that could not be interpreted or adequately resolved are designated as multiplet (*m*) or broad (*br*). All the absorption spectra were measured using a Perkin Elmer Lambda 25 UV/Vis Spectrophotometer.

1.2 Synthesis of Starting Materials.

1.2.1 Synthesis of electrophilic *S*-sources.

The **S** sources **3a**, **3b**, **3c** were prepared according to a reported procedure[1].

The **S** source **3d** was prepared in 87% yield (5 mmol) from isopropanol and sulfur chloride according to a reported procedure[2].

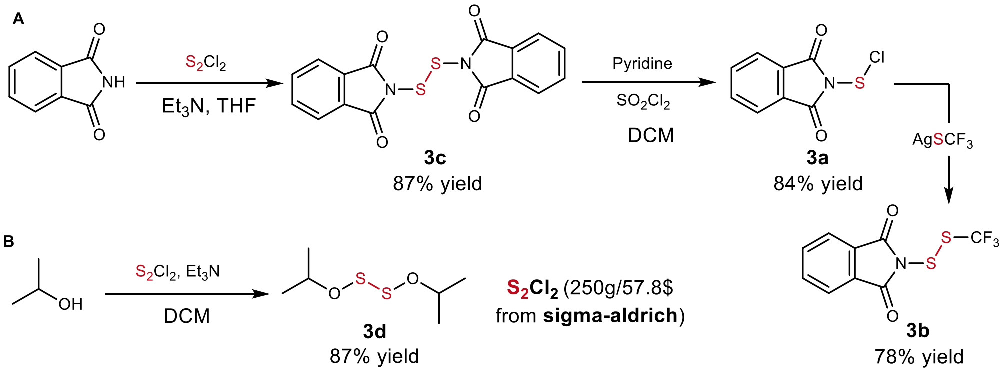

Supplementary Fig. S1 Preparation of electrophilic ***S***-sources **3a–3d**

1.2.2 Synthesis of nitro compounds.

(4-Nitrobutyl)benzene (**1a**)

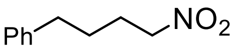

1a

The nitro compound **1a** was prepared in 51% yield (5 mmol) from 3-phenylpropanal in two steps according to a reported procedure. Spectral data was consistent with literature reported[3].

(2-Nitroethyl)benzene (**1b**)

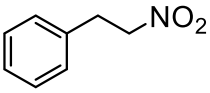

1b

The nitro compound was prepared in 92% yield (2.5 mmol) according to a reported procedure[4].

Methyl 4-nitrobutanoate (**1c**)

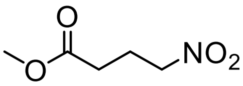

1c

The nitro compound (**1c**) was prepared in 64% yield (4.2 mmol) according to a reported procedure[5].

2-(2-Nitroethyl)-1,3-dioxolane (**1d**)

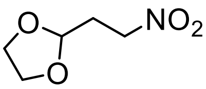

1d

The nitro compound was prepared in 76% yield (5 mmol) from 3-nitropropanal in one step according to a reported procedure[6].

3-Nitropropan-1-ol (**1e**)

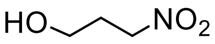

1e

The nitro compound was prepared in 70% yield (3 mmol) from acrylaldehyde in two steps according to a reported procedure[3].

(Nitromethyl)benzene (**1f**)

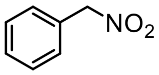

1f

The nitro compound was prepared in 89% yield (3.6 mmol) according to a reported procedure[7].

1-Chloro-5-nitropentane (**1g**)

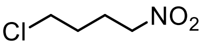

1g

The nitro compound was prepared in 70% yield (5 mmol) according to a reported procedure[8].

(1-Nitropropan-2-yl)benzene (**1h**)

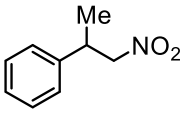

1h

The nitro compound was prepared in 92% yield (5 mmol) according to a reported procedure[9].

(1,1,1-Trifluoro-3-nitropropan-2-yl)benzene (**1i**)

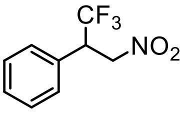

1i

The nitro compound was prepared in 71% yield (2.3 mmol) from (*E*)-(3,3,3-trifluoro-1-nitroprop-1-en-2-yl)benzene in one step according to a reported procedure[10].

Benzyl (2-nitro-1-phenylethyl)carbamate (**1j**)

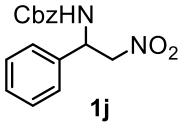

1j

The nitro compound was prepared in 70% yield (5 mmol) according to a reported procedure[11].

***N*-(2-nitro-1-phenylethyl)acetamide** (**1k**)

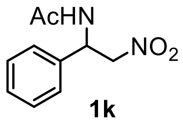

1k

The nitro compound was prepared in 51% yield (5 mmol) according to a reported procedure[12].

2-nitro-1-phenylethan-1-one (1l)

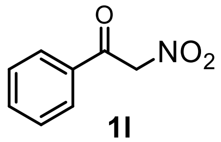

1l

The nitro compound was prepared in 78% yield (5 mmol) according to a reported procedure[13].

__1-Iodo-4-(nitromethyl)benzene__ (**1m**)

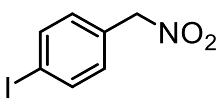

1m

The nitro compound was prepared in 53% yield (5 mmol) from 1-Iodo-4-(iodomethyl)benzene in one step according to a reported procedure[14].

^{1}H NMR (101 MHz, $CDCl_3$) δ 7.78 (d, *J* = 8.3 Hz, 2H), 7.19 (d, *J* = 8.3 Hz, 2H), 5.37 (s, 2H). ^{13}C NMR (101 MHz, $CDCl_3$) δ 138.4, 131.8, 129.2, 96.5, 79.5.

__4-(2-Nitroethyl)phenol__ (**1n**)

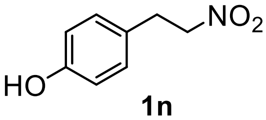

1n

The nitro compound was prepared in 79% yield (5 mmol) from 3-phenylpropanal in two steps according to a reported procedure[15].

__***tert*-Butyl (*R*)-(1-nitro-4-phenylbutan-2-yl)carbamate**__ (**7a**)

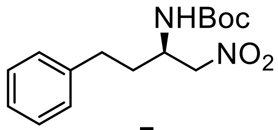

7a

The nitro compound was prepared in 95% yield (5 mmol) from 3-phenylpropanal through two steps according to a reported procedure[11], and used after recrystallization (PE/EA=20:1). The ee value of the chiral nitro compound was determined by HPLC using a chiral stationary phase, ee = 99% (Chiralpak AD-H, hexane/*i*-PrOH = 95:5, 254 nm, 0.75 mL/min, t_{major} = 19.6 min, t_{minor} = 21.8 min).

__***tert*-Butyl (*R*)-(3-methyl-1-nitrobutan-2-yl)carbamate**__ (**7b**)

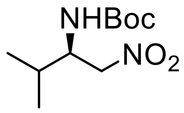

7b

The nitro compound was prepared in 92% yield (5 mmol) from isobutyraldehyde through two steps according to a reported procedure[11], and used after recrystallization (PE/EA=20:1). The ee value of the chiral nitro compound was determined by HPLC using a chiral stationary phase, ee > 99% (Chiralpak AD-H, hexane/i-PrOH = 90:10, 254 nm, 0.75 mL/min, t_{major} = 9.1 min, t_{minor} = 13.0 min).

***tert*-Butyl (*R*)-(1-cyclohexyl-2-nitroethyl)carbamate** (**7c**)

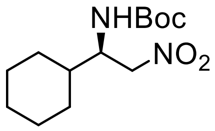

7c

The nitro compound was prepared in 81% yield (5 mmol) from cyclohexanecarbaldehyde through two steps according to a reported procedure[11], and used after recrystallization (PE/EA=20:1). The ee value of the chiral nitro compound was determined by HPLC using a chiral stationary phase, ee > 99% (Chiralpak AD-H, hexane/*i*-PrOH = 90:10, 254 nm, 1 mL/min, t_{major} = 7.5 min, t_{minor} = 9.5 min).

***tert*-Butyl (*R*)-(4-methyl-1-nitropentan-2-yl)carbamate** (**7d**)

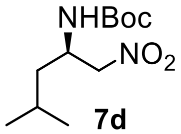

7d

The nitro compound was prepared in 85% yield (5 mmol) from 3-methylbutanal through two steps according to a reported procedure[16], and used after recrystallization (PE/EA=20:1). The ee value of the chiral nitro compound was determined by HPLC using a chiral stationary phase, ee > 99% (Chiralpak AD-H, hexane/*i*-PrOH = 90:10, 254 nm, 0.8 mL/min, t_{major} = 8.1 min, t_{minor} = 9.5 min).

***tert*-Butyl (*R*)-(1-nitro-3-phenylpropan-2-yl)carbamate** (**7e**)

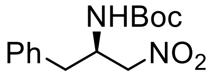

7e

The nitro compound was prepared in 70% yield (5 mmol) from 2-phenylacetaldehyde through two steps according to a reported procedure[16], and used after recrystallization (PE/EA=20:1). The ee value of the chiral nitro compound was determined by HPLC using a chiral stationary phase, ee > 99% (Chiralpak AD-H, hexane/*i*-PrOH = 90:10, 254 nm, 1 mL/min, t_{major} = 8.5 min, t_{minor} = 10.7 min).

***tert*-Butyl (*R*)-(2-nitro-1-phenylethyl)carbamate** (**7f**)

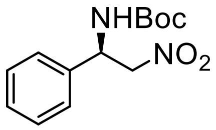

7f

The nitro compound was prepared in 91% yield (5 mmol) from benzaldehyde through two steps according to a reported procedure[11], and used after recrystallization (PE/EA=20:1). The ee value of the chiral nitro compound was determined by HPLC using a chiral stationary phase, ee > 99% (Chiralpak AD-H, hexane/*i*-PrOH = 95:5, 254 nm, 0.75 mL/min, t_{major} = 51.6 min, t_{minor} = 44.9 min).

(*R*)-1-nitro-4-phenylbutan-2-amine hydrochloride (**7g**)[17]

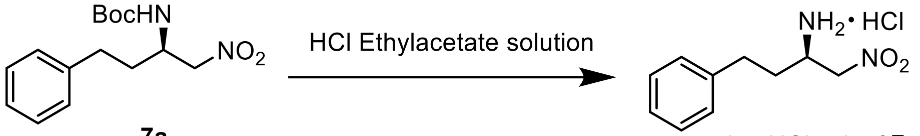

7a

The deprotected amine salt of **7a** was prepared by addition of HCl in ethylacetate, and was used directly for the next step of the reaction.

1.2.3 General procedure to prepare peptide nitroalkanes.

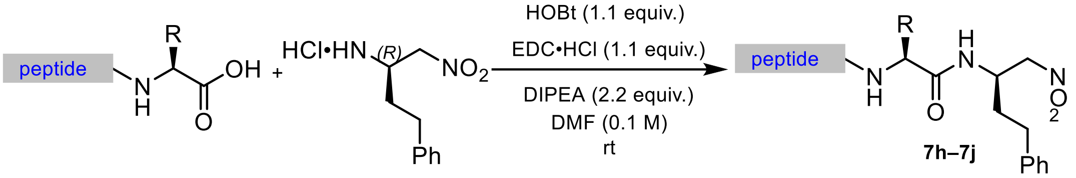

7h–7j

General procedure: *N*-BOC protected amino acid (1 equiv.) and **7g** (1 equiv.) were added to a 50 mL round bottom flask, followed by adding DMF (10 mL). After stirring for 10 minutes in an ice bath, HOBt (1.1 equiv.) and EDC (1.1 equiv.) were added. The reaction was further stirred in an ice bath for 10 minutes, then DIPEA (2.2 equiv.) was added. The reaction was further stirred in an ice bath for 30 min before rising to room temperature and stirred overnight, quenched with H_2O, extracted with ethyl acetate and the organic phase was collected. The organic phase was extracted with 1M HCl, *sat.* $NaHCO_3$ solution, *sat.* NaCl solution, dried over anhydrous $MgSO_4$. After filtration, the solution was concentrated under reduced pressure and the crude residue was purified by flash-column chromatography (PE/EA = Petroleum ether/Ethyl acetate).

***tert*-Butyl ((*S*)-1-(((*R*)-1-nitro-4-phenylbutan-2-yl)amino)-1-oxo-3-phenylpropan-2-yl)carbamate** (**7h**)

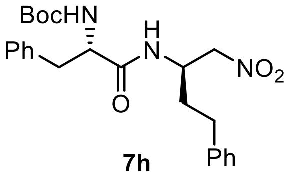

7h

Following general procedure: **7h** was isolated as white solid in 74 yield (980 mg; 3 mmol) which was purified by silica gel chromatography (DCM: MeOH = 20:1~10:1, R_f = 0.45 (DCM: MeOH = 10:1)).

^{1}H NMR (400 MHz, $CDCl_3$) δ 7.29 (q, *J* = 7.0 Hz, 4H), 7.21 (q, *J* = 8.1 Hz, 4H), 7.09 (d, *J* = 7.4 Hz, 2H), 6.51 (d, *J* = 8.5 Hz, 1H), 5.05 (d, *J* = 7.7 Hz, 1H), 4.47 (d, *J* = 4.7 Hz, 1H), 4.34 (q, *J* = 7.4 Hz, 2H), 3.12 – 3.00 (m, 2H), 2.52 (t, *J* = 9.6 Hz, 2H), 1.80 (q, *J* = 7.4 Hz, 2H), 1.41 (s, 9H).

^{13}C NMR (101 MHz, $CDCl_3$) δ 166.9, 150.9, 135.5, 131.8, 124.7, 124.5, 124.1, 123.9, 123.7, 122.5, 121.7, 75.9, 51.4, 42.6, 33.2, 31.8, 28.3, 27.2, 23.6.

HRMS (ESI) m/z: $[M+Na]^+$ calcd. for $C_{24}H_{31}N_3O_5Na$ 464.2156; found, 464.2160.

FT-IR (neat): 3304, 2926, 2859, 1687, 1658, 1548, 1495, 1452, 1379, 1082, 1032, 912, 699 cm^{-1}.

Benzyl *tert*-butyl ((*S*)-6-(((*S*)-1-(((*R*)-1-nitro-4-phenylbutan-2-yl)amino)-1-oxopropan-2-yl)amino)-6-oxohexane-1,5-diyl)dicarbamate (**7i**)

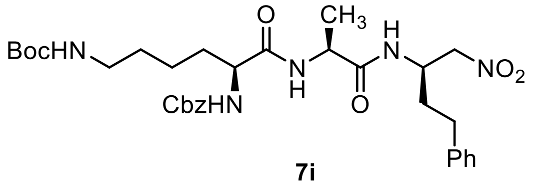

7i

Following general procedure: **7i** was isolated as yellow solid in 69% yield (866 mg; 2 mmol) which was purified by silica gel chromatography (DCM: MeOH = 20:1~10:1, R_f = 0.40 (DCM: MeOH = 10:1)).

^{1}H NMR (400 MHz, $CDCl_3$) δ 7.42 – 7.07 (m, 11H), 6.65 (s, 1H), 6.33 (s, 1H), 5.19 – 4.97 (m, 2H), 4.78 (s, 1H), 4.62 – 4.26 (m, 4H), 4.04 (s, 1H), 3.10 (d, *J* = 28.6 Hz, 2H), 2.91 – 2.51 (m, 2H), 2.11 – 1.63 (m, 4H), 1.40 (dd, *J* = 16.8, 10.2 Hz, 15H).

^{13}C NMR (101 MHz, $CDCl_3$) δ172.6, 172.2, 157.6, 157.1, 140.8, 140.4, 136.3, 135.9, 128.7, 128.6, 128.5, 126.2, 79.7, 78.2, 67.7, 56.5, 49.3, 47.7, 38.8, 33.2, 32.1, 30.6, 30.0, 28.5, 22.2, 17.7.

HRMS (ESI) m/z: $[M+Na]^+$ calcd. for $C_{32}H_{45}N_5O_8Na$ 650.3160; found, 650.3182.

FT-IR (neat): 3355, 3292, 3055, 3029, 2982, 2934, 2863, 1681, 1649, 1530, 1454, 1394, 1366, 1168, 1093, 1040, 912, 696 cm^{-1}.

***tert*-Butyl (2-((*R*)-2-(((*R*)-1-nitro-4-phenylbutan-2-yl)carbamoyl)pyrrolidin-1-yl)-2-oxoethyl)carbamate** (**7j**)

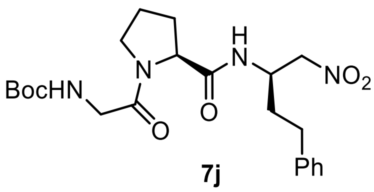

7j

Following general procedure: **7j** was isolated as yellow solid in 76% yield (682 mg; 2.0 mmol) which was purified by silica gel chromatography (DCM: MeOH = 20:1~10:1, R_f = 0.40 (DCM: MeOH = 10:1)).

^{1}H NMR (400 MHz, $CDCl_3$) δ 7.38 – 7.27 (m, 3H), 7.23 – 7.16 (m, 3H), 5.36 (s, 1H), 4.64 – 4.51 (m, 2H), 4.48 – 4.35 (m, 2H), 4.07 – 3.83 (m, 2H), 3.55 (td, *J* = 9.1, 3.2 Hz, 1H), 3.40 (td, *J* = 9.6, 7.4 Hz, 1H), 2.80 – 2.63 (m, 2H), 2.47 – 2.39 (m, 1H), 2.16 – 2.05 (m, 1H), 2.01 (ddd, *J* = 11.5, 6.5, 2.3 Hz, 1H), 1.97 – 1.79 (m, 3H), 1.45 (s, 9H).

^{13}C NMR (101 MHz, $CDCl_3$) δ 171.1, 169.5, 140.4, 128.7, 128.5, 126.4, 80.0, 78.1, 60.1, 47.7, 46.3, 43.3, 33.4, 32.1, 28.4, 26.9, 24.9.

HRMS (ESI) m/z: $[M+Na]^+$ calcd. for $C_{22}H_{32}N_4O_6Na$ 471.2214; found, 471.2228.

FT-IR (neat): 3323, 1712, 1652, 1552, 1453, 1437, 1366, 1164, 1049, 1032, 915, 702 cm^{-1}.

***tert*-Butyl (3-nitropropyl)carbamate** (**12**)

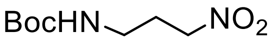

12

The nitro compound was prepared in 48% yield (20 mmol) in one step according to a reported procedure[18].

1-Methoxy-4-(nitromethyl)benzene (**15**)

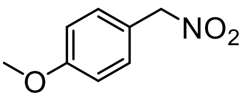

15

The nitro compound was prepared in 58% yield (5 mmol) according to a reported procedure[19].

1.3. General procedure and characterization of thioamide products

1.3.1 Optimization of reaction conditions

Supplementary Table S1. Details on the optimization of yhioamidation reaction conditions[a]

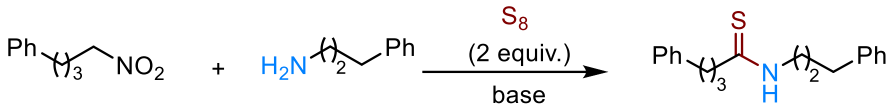

1a **2a** **4a**

entry	solvent	base	yield (%)[b]
1	THF	-	12
2	CH_3CN	K_2CO_3	21
3	DMSO	K_2CO_3	25
4	DCE	K_2CO_3	16
5	1,4-dioxane	K_2CO_3	23
6	acetone	K_2CO_3	trace
7	DMF	K_2CO_3	31
8	THF	K_2CO_3	37
9	THF	$KHCO_3$	30
10	THF	Na_2CO_3	24
11	THF	$LiOH·H_2O$	32
12	THF	Cs_2CO_3	28
13	THF	Na_2S	98
14	THF	Et_3N	16
15	THF	DBU	trace
16	THF	pyridine	trace
17	THF	DIPEA	15
18[c]	THF	Na_2S	78

[a]Unless noted otherwise, reactions were carried out with 0.2 mmol of **1a**, 0.4 mmol of **2a**, 0.4 mmol of S_8 and 0.4 mmol of base in 2 mL of solvent until **1a** was consumed monitored with TLC (typically complete for 24h). [b]Yield of isolated product. [c]S_8 (1.25 equiv.) was used.

1.3.2 General thioamidation procedure.

General procedure A: The reaction was conducted with no special precautions from air or water. Nitro compound **1** (0.2 mmol) was added to a 10 mL reaction tube, followed by adding THF (2 mL). then S_8 (2.0 equiv., 102.4 mg), Na_2S (2.0 equiv., 31.2

mg) and the amine **2** (2.0 equiv.) were added. The reaction was monitored by TLC until the nitroalkane was consumed (typically completed within 24h), quenched with *sat.* NH_4Cl, extracted with ethyl acetate, and the organic phase was collected and dried over anhydrous Na_2SO_4. After filtration, the solution was concentrated under reduced pressure and the crude residue was purified by flash-column chromatography (PE/EA = Petroleum ether/Ethyl acetate).

1.3.3 Structural Characterization of thioamide products

4-Phenyl-*N*-(3-phenylpropyl)butanethioamide (**4a**)

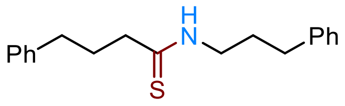

4a

Following procedure A: **4a** was isolated as yellow oil in 98% yield (58.2 mg; 0.196 mmol) which was purified by silica gel chromatography (PE:EA = 20:1~5:1, R_f = 0.45 (PE:EA = 5:1)).

^{1}H NMR (400 MHz, $CDCl_3$) δ 7.29 – 7.24 (m, 4H), 7.19 – 7.13 (m, 6H), 3.69 – 3.62 (m, 2H), 2.64 (dt, *J* = 23.3, 7.5 Hz, 4H), 2.55 – 2.49 (m, 2H), 2.08 – 1.91 (m, 4H).

^{13}C NMR (101 MHz, $CDCl_3$) δ 205.0, 141.5, 141.2, 128.7, 128.6, 128.6, 128.5, 126.4, 126.2, 46.3, 45.9, 35.0, 33.6, 30.8, 29.5.

HRMS (ESI) m/z: $[M+H]^+$ calcd. for $C_{19}H_{24}NS$ 298.1624; found, 298.1619.

FT-IR (neat): 3237, 2929, 2857, 1530, 1495, 1453, 1406, 1337, 1123, 1084, 1031, 909, 699 cm^{-1}.

***N*-Benzyl-4-phenylbutanethioamide** (**4b**)

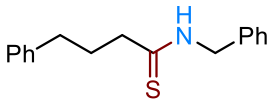

4b

Following procedure A: **4b** was isolated as yellow oil in 98% yield (52.7 mg; 0.196 mmol) which was purified by silica gel chromatography (PE:EA = 20:1~5:1, R_f = 0.45 (PE:EA = 5:1)).

^{1}H NMR (400 MHz, $CDCl_3$) δ 7.35 – 7.20 (m, 8H), 7.17 – 7.10 (m, 3H), 4.77 (d, *J* = 5.2 Hz, 2H), 2.62 (q, *J* = 7.9 Hz, 4H), 2.16 – 2.05 (m, 2H).

^{13}C NMR (101 MHz, $CDCl_3$) δ 205.2, 141.4, 136.3, 129.1, 128.6, 128.6, 128.5, 128.3, 126.2, 50.3, 46.2, 34.9, 30.9

HRMS (ESI) m/z: $[M+H]^+$ calcd. for $C_{17}H_{20}NS$ 270.1311; found, 270.1301.

FT-IR (neat): 3221, 3025, 1528, 1495, 1453, 1405, 1340, 1125, 947, 698 cm^{-1}.

<u>*N*-(4-Hydroxybutyl)-4-phenylbutanethioamide</u> (**4c**)

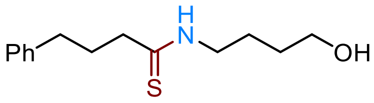

4c

Following procedure A: **4c** was isolated as yellow oil in 88% yield (44.2 mg; 0.176 mmol) which was purified by silica gel chromatography (PE:EA = 5:1~1:1, R_f = 0.2 (PE:EA = 1:1)).

^{1}H NMR (400 MHz, $CDCl_3$) δ 8.07 (s, 1H), 7.30 – 7.24 (m, 2H), 7.21 – 7.13 (m, 3H), 3.63 (dt, *J* = 8.3, 6.6 Hz, 4H), 2.63 (dt, *J* = 9.4, 7.8 Hz, 4H), 2.10 (ddd, *J* = 7.8, 6.6, 1.5 Hz, 2H), 1.73 (p, *J* = 7.0 Hz, 2H), 1.60 (p, *J* = 6.4 Hz, 2H).

^{13}C NMR (101 MHz, $CDCl_3$) δ 204.8, 141.5, 128.6, 128.5, 126.1, 62.1, 46.1, 45.9, 34.9, 30.8, 29.7, 24.6.

HRMS (ESI) m/z: $[M+H]^+$ calcd. for $C_{14}H_{22}NOS$ 252.1417; found, 252.1411.

FT-IR (neat): 3241, 3024, 2934, 2860, 1541, 1495, 1453, 1410, 1346, 1126, 1069, 1028, 909, 700 cm^{-1}.

<u>*N*-Allyl-4-phenylbutanethioamide</u> (**4d**)

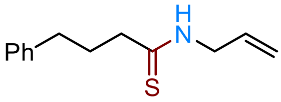

4d

Following procedure A: **4d** was isolated as yellow oil in 88% yield (38.5 mg; 0.176 mmol) which was purified by silica gel chromatography (PE:EA = 20:1~5:1, R_f = 0.45 (PE:EA = 5:1)).

^{1}H NMR (400 MHz, $CDCl_3$) δ 7.27 (dd, *J* = 8.3, 6.3 Hz, 2H), 7.21 – 7.13 (m, 3H), 5.94 – 5.81 (m, 1H), 5.28 – 5.19 (m, 2H), 4.29 – 4.24 (m, 2H), 2.69 – 2.61 (m, 4H), 2.12 (tt, *J* = 8.9, 6.8 Hz, 2H).

^{13}C NMR (101 MHz, $CDCl_3$) δ 205.4, 141.4, 131.9, 128.6, 126.2, 118.7 (2C), 48.5, 46.2, 34.9, 30.9.

HRMS (ESI) m/z: $[M+H]^+$ calcd. for $C_{13}H_{18}NS$ 220.1155; found, 220.1143.

FT-IR (neat): 3237, 3024, 3006, 2987, 2924, 2861, 1646, 1527, 1496, 1453, 1398, 1317, 1180, 1124, 924, 700 cm^{-1}.

<u>*N*-Cyclohexyl-4-phenylbutanethioamide</u> (**4e**)

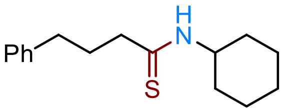

4e

Following procedure A: **4e** was isolated as yellow oil in 97% yield (50.6 mg; 0.194 mmol) which was purified by silica gel chromatography (PE:EA = 20:1~5:1, R_f = 0.5 (PE:EA =5:1)).

^{1}H NMR (400 MHz, $CDCl_3$) δ 7.30 – 7.24 (m, 2H), 7.20 – 7.15 (m, 3H), 4.43 – 4.31 (m, 1H), 2.62 (dt, *J* = 22.4, 7.6 Hz, 4H), 2.16 – 2.01 (m, 4H), 1.69 (ddt, *J* = 32.5, 12.9, 3.7 Hz, 3H), 1.45 – 1.33 (m, 2H), 1.26 – 1.13 (m, 3H).

^{13}C NMR (101 MHz, $CDCl_3$) δ 203.3, 141.5, 128.6, 128.5, 126.1, 54.3, 46.6, 34.8, 31.7, 30.8, 25.5, 24.8.

HRMS (ESI) m/z: $[M+H]^+$ calcd. for $C_{16}H_{24}NS$ 262.1624; found, 262.1613.

FT-IR (neat): 3237, 3025, 2929, 2853, 1529, 1495, 1451, 1410, 1347, 1117, 1089, 1028, 892, 699 cm^{-1}.

<u>1-Morpholino-4-phenylbutane-1-thione</u> (**4f**)

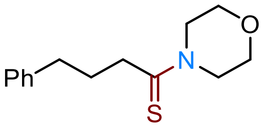

4f

Following procedure A: **4f** was isolated as yellow oil in 93% yield (46.3 mg; 0.186 mmol) which was purified by silica gel chromatography (PE:EA = 20:1~5:1, R_f = 0.3 (PE:EA = 5:1)).

^{1}H NMR (400 MHz, $CDCl_3$) δ 7.32 – 7.27 (m, 2H), 7.20 (td, *J* = 7.3, 1.3 Hz, 3H), 4.34 – 4.30 (m, 2H), 3.78 – 3.73 (m, 2H), 3.65 (dd, *J* = 5.7, 3.6 Hz, 2H), 3.57 (dd, *J* = 5.7, 3.7 Hz, 2H), 2.90 – 2.81 (m, 2H), 2.72 (t, *J* = 7.4 Hz, 2H), 2.09 – 1.99 (m, 2H).

^{13}C NMR (101 MHz, $CDCl_3$) δ 203.4, 141.2, 128.5, 128.5, 126.2, 66.5, 66.5, 50.0, 49.9, 42.6, 35.3, 30.7.

HRMS (ESI) m/z: $[M+H]^+$ calcd. for $C_{14}H_{20}NOS$ 250.1260; found, 250.1242.

FT-IR (neat): 2963, 2917, 2852, 1485, 1432, 1112, 1004, 921, 699 cm^{-1}.

***N*-Benzyl-*N*-methyl-4-phenylbutanethioamide** (**4g**)

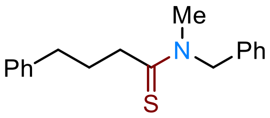

4g

Following procedure A: **4g** was isolated as yellow oil in 88% yield (49.8 mg; 0.176 mmol) which was purified by silica gel chromatography as a NMR observable rotamers of two thioamide forms A and B on the NMR time scale. (PE:EA = 20:1~5:1, R_f = 0.4 (PE:EA = 5:1)).

^{1}H NMR (400 MHz, $CDCl_3$) δ 7.39 – 7.09 (m, 11H, A+B), 7.05 – 7.00 (m, 1H), 5.32 (s, 1H, B), 4.69 (s, 1H, A), 3.43 (s, 1H, B), 3.04 (s, 2H, A), 2.88 – 2.81 (m, 2H), 2.75 (t, *J* = 7.5 Hz, 1H), 2.67 (t, *J* = 7.5 Hz, 1H), 2.14 (dddd, *J* = 10.1, 7.5, 5.9, 3.7 Hz, 2H).

^{13}C NMR (101 MHz, $CDCl_3$) δ 205.3(A), 205.1(B), 141.5(A), 141.5(B), 135.7(A), 135.1(B), 129.2(A), 128.9(B), 128.6(A), 128.5, 128.5(B), 128.1, 128.0(A), 126.4(B), 126.2(A), 126.1(B), 58.5(A), 57.4(B), 43.3(A), 43.1(B), 42.6(A), 38.6(B), 35.4(A), 35.3(B), 31.3(A), 30.7(B).

HRMS (ESI) m/z: $[M+H]^+$ calcd. for $C_{18}H_{22}NS$ 284.1468; found, 284.1454.

FT-IR (neat): 2925, 1495, 1452, 1398, 1127, 1110, 1075, 1027, 963, 699 cm^{-1}.

<u>4-Phenyl-1-(pyrrolidin-1-yl)butane-1-thione</u> (**4h**)

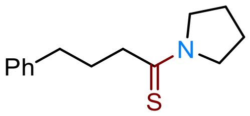

4h

Following procedure A: **4h** was isolated as yellow oil in 93% yield (43.3 mg; 0.186 mmol) which was purified by silica gel chromatography (PE:EA = 20:1~5:1, R_f = 0.5 (PE:EA =5:1)).

^{1}H NMR (400 MHz, $CDCl_3$) δ 7.31 – 7.25 (m, 2H), 7.22 – 7.15 (m, 3H), 3.84 (t, *J* = 6.9 Hz, 2H), 3.48 (t, *J* = 6.8 Hz, 2H), 2.70 (dt, *J* = 18.7, 7.8 Hz, 4H), 2.13 (dq, *J* = 10.0, 7.7 Hz, 2H), 1.98 (ddt, *J* = 30.8, 13.3, 6.7 Hz, 4H).

^{13}C NMR (101 MHz, $CDCl_3$) δ 200.2, 141.7, 128.6, 128.5, 126.0, 53.9, 50.5, 43.1, 35.3, 30.3, 26.4, 24.4.

HRMS (ESI) m/z: $[M+H]^+$ calcd. for $C_{14}H_{20}NS$ 234.1311; found, 234.1308.

FT-IR (neat): 2967, 2870, 1485, 1472, 1450, 1329, 1100, 910, 701 cm^{-1}.

<u>N, N'</u>**<u>-(Propane-1,3-diyl)bis(4-phenylbutanethioamide)</u>** (**4i**)

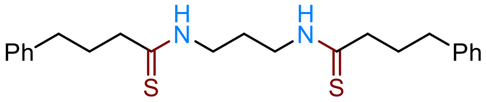

4i

Following procedure A: doubly thioacylated compound **4i** was isolated as yellow oil in 88% yield (70.1 mg; 0.176 mmol) which was purified by silica gel chromatography (PE:EA= 10:1~1:1, R_f = 0.45 (PE:EA = 1:1)).

^{1}H NMR (400 MHz, $CDCl_3$) δ 8.20 (s, 2H), 7.31 – 7.15 (m, 10H), 3.70 (q, *J* = 6.1 Hz, 4H), 2.67 (td, *J* = 7.8, 2.9 Hz, 8H), 2.12 (p, *J* = 7.6 Hz, 4H), 1.90 (p, *J* = 6.1 Hz, 2H).

^{13}C NMR (101 MHz, $CDCl_3$) δ 205.9, 141.2, 128.5, 128.5, 126.1, 46.5, 42.1, 34.9, 30.8, 26.8.

HRMS (ESI) m/z: $[M+H]^+$ calcd. for $C_{23}H_{31}N_2S_2$ 399.1923; found, 399.1915.

FT-IR (neat): 3219, 2928, 2855, 1536, 1495, 1453, 1406, 1125, 700 cm^{-1}.

<u>(R)</u>**<u>-4-Phenyl-</u>*****<u>N</u>*****<u>-(1-phenylethyl)butanethioamide</u>** (**4j**)

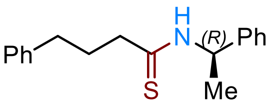

4j

Following procedure A using chiral amine of 98 % ee: **4j** was isolated as yellow oil in 97% yield (54.9 mg; 0.194 mmol) which was purified by silica gel chromatography (PE:EA = 20:1~5:1, R_f = 0.6 (PE:EA =5:1)). The ee value was calculated as 98% (Chiralpak AD-H, hexane/*i*-PrOH = 95:5, 254 nm, 1 mL/min, t_{major}= 14.52 min, t_{minor}= 12.84 min). ^{1}H NMR (400 MHz, $CDCl_3$) δ 7.39 – 7.27 (m, 6H), 7.26 – 7.23 (m, 1H), 7.20 – 7.11 (m, 3H), 5.78 (p, *J* = 7.0 Hz, 1H), 2.62 (ddt, *J* = 9.5, 7.1, 4.0 Hz, 4H), 2.12 (p, *J* = 7.8 Hz, 2H), 1.59 (d, *J* = 6.9 Hz, 3H).

HPLC traces of compound 4j as racemate and as isolated from reaction:

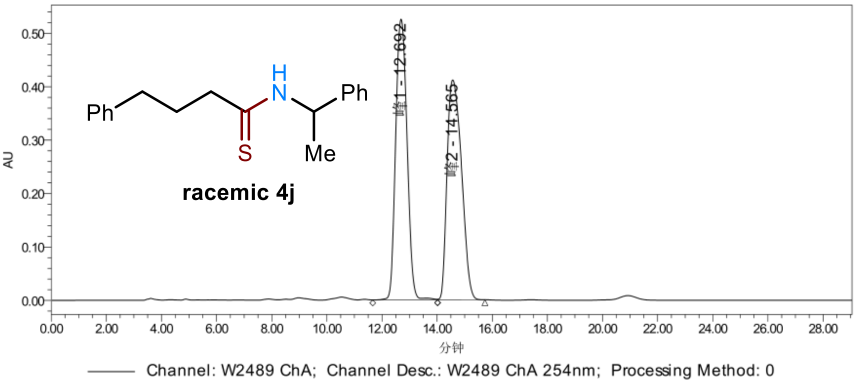

	Channel Description	Peak Name	RT (min)	Area (磺*sec)	% Area	Height (磺)
1	W2489 ChA 254nm	峰1	12.692	15088970	50.21	525854
2	W2489 ChA 254nm	峰2	14.565	14960524	49.79	411786

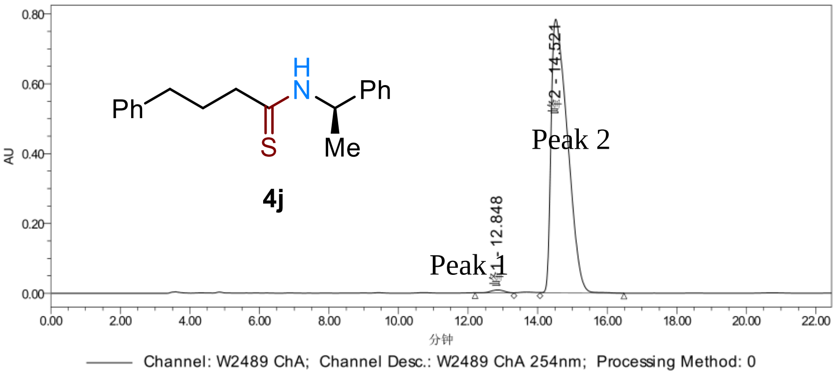

	Channel Description	Peak Name	RT (min)	Area (磺*sec)	% Area	Height (磺)
1	W2489 ChA 254nm	峰1	12.848	215375	0.83	8212
2	W2489 ChA 254nm	峰2	14.521	25693593	99.17	784715

Methyl (4-phenylbutanethioyl)-*L*-serinate (**4k**)

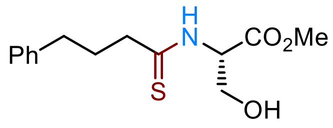

4k

Following procedure A: **4k** was isolated as yellow oil in 82% yield (46.1 mg; 0.164 mmol) which was purified by silica gel chromatography (PE:EA = 5:1~1:1, R_f = 0.5).

^{1}H NMR (400 MHz, $CDCl_3$) δ 8.09 (d, *J* = 7.4 Hz, 1H), 7.31 – 7.25 (m, 2H), 7.22 – 7.16 (m, 3H), 5.34 – 5.28 (m, 1H), 4.07 (d, *J* = 3.3 Hz, 2H), 3.80 (s, 3H), 2.69 (dt, *J* = 12.1, 7.0 Hz, 4H), 2.14 (pd, *J* = 7.4, 2.1 Hz, 2H).

^{13}C NMR (101 MHz, $CDCl_3$) δ 206.5, 170.4, 141.4, 128.6, 128.6, 126.2, 62.1, 59.6, 53.1, 46.0, 34.8, 30.8.

HRMS (ESI) m/z: $[M+H]^+$ calcd. for $C_{14}H_{20}NO_3S$ 282.1158; found, 282.1147.

FT-IR (neat): 3310, 2951, 1739, 1517, 1496, 1437, 1406, 1329, 1125, 1067, 909, 701 cm^{-1}.

***tert*-Butyl (4-phenylbutanethioyl)-*L*-phenylalaninate** (**4l**)

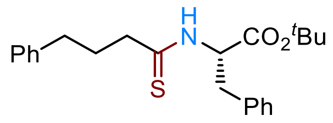

4l

Following procedure A: **4l** was isolated as yellow oil in 80% yield (61.4 mg; 0.160 mmol) which was purified by silica gel chromatography (PE:EA = 20:1~5:1, R_f = 0.5 (PE:EA = 5:1)).

^{1}H NMR (400 MHz, $CDCl_3$) δ 7.66 (d, *J* = 7.7 Hz, 1H), 7.28 (d, *J* = 6.9 Hz, 1H), 7.26 – 7.19 (m, 4H), 7.19 – 7.11 (m, 5H), 5.26 (td, *J* = 7.1, 4.7 Hz, 1H), 3.34 (dd, *J* = 13.9, 6.7 Hz, 1H), 3.22 (dd, *J* = 13.9, 4.8 Hz, 1H), 2.63 (td, *J* = 7.6, 2.9 Hz, 4H), 2.13 – 2.03 (m, 2H), 1.41 (s, 9H).

^{13}C NMR (101 MHz, $CDCl_3$) δ 204.9, 170.3, 141.4, 135.9, 129.8, 129.6, 128.6, 128.5, 127.3, 126.1, 83.2, 58.8, 46.3, 36.3, 34.9, 30.8, 28.1.

HRMS (ESI) m/z: $[M+Na]^+$ calcd. for $C_{23}H_{29}NO_2SNa$ 406.1811; found, 406.1800.

FT-IR (neat): 3303, 2977, 1722, 1512, 1496, 1454, 1401, 1367, 1153, 700 cm^{-1}.

2-Phenyl-*N*-(3-phenylpropyl)ethanethioamide (**5a**)

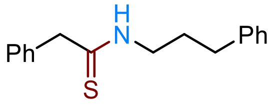

5a

Following procedure A: **5a** was isolated as yellow oil in 93% yield (50.1 mg; 0.186 mmol) which was purified by silica gel chromatography (PE:EA = 20:1~5:1, R_f = 0.5 (PE:EA = 5:1)).

^{1}H NMR (400 MHz, $CDCl_3$) δ 7.38 – 7.30 (m, 3H), 7.25 – 7.15 (m, 5H), 7.07 – 7.03 (m, 2H), 4.05 (s, 2H), 3.67 – 3.56 (m, 2H), 2.57 – 2.48 (m, 2H), 1.85 (p, J = 7.5 Hz, 2H).

^{13}C NMR (101 MHz, $CDCl_3$) δ 202.0, 141.0, 135.1, 129.6, 129.4, 128.7, 128.5, 128.4, 128.0, 126.3, 53.2, 45.7, 33.2, 29.4.

HRMS (ESI) m/z: $[M+H]^+$ calcd. for $C_{17}H_{20}NS$ 270.1311; found, 270.1293.

FT-IR (neat): 3359, 2926, 1531, 1494, 1453, 1406, 1343, 1121, 1030, 913, 699 cm^{-1}.

***N*-(3-Phenylpropyl)propanethioamide** (**5b**)

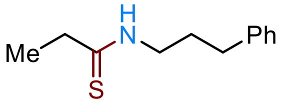

5b

Following procedure A: **5b** was isolated as yellow oil in 99% yield (41.0 mg; 0.198 mmol) which was purified by silica gel chromatography (PE:EA = 20:1~5:1, R_f = 0.5 (PE:EA = 5:1)).

^{1}H NMR (400 MHz, $CDCl_3$) δ 7.30 – 7.25 (m, 2H), 7.19 (td, *J* = 6.6, 1.3 Hz, 3H), 3.70 – 3.62 (m, 2H), 2.72 – 2.64 (m, 2H), 2.58 (q, *J* = 7.5 Hz, 2H), 1.98 (p, *J* = 7.5 Hz, 2H), 1.22 (t, *J* = 7.5 Hz, 3H).

^{13}C NMR (101 MHz, $CDCl_3$) δ 206.6, 141.2, 128.7, 128.5, 126.3, 46.0, 40.1, 33.5, 29.5, 13.8.

HRMS (ESI) m/z: $[M+H]^+$ calcd. for $C_{12}H_{18}NS$ 208.1155; found, 208.1143.

FT-IR (neat): 3231, 2976, 1533, 1496, 1452, 1409, 1364, 1145, 1097, 1029, 955, 699 cm^{-1}.

Methyl 4-((3-phenylpropyl)amino)-4-thioxobutanoate (**5c**)

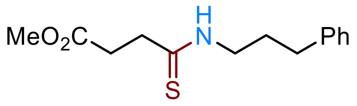

5c

Following procedure A: **5c** was isolated as yellow oil in 58% yield (30.8 mg; 0.116 mmol) which was purified by silica gel chromatography (PE:EA = 20:1~5:1, R_f = 0.4 (PE:EA = 5:1)).

^{1}H NMR (400 MHz, $CDCl_3$) δ 7.31 – 7.26 (m, 2H), 7.22 – 7.17 (m, 3H), 3.70 – 3.61 (m, 6H), 2.84 (q, *J* = 1.8 Hz, 4H), 2.70 – 2.65 (m, 2H), 1.98 (p, *J* = 7.5 Hz, 2H).

^{13}C NMR (101 MHz, $CDCl_3$) δ 203.1, 174.0, 141.2, 128.6, 128.5, 126.2, 52.1, 45.9, 40.8, 33.4, 32.9, 29.5.

HRMS (ESI) m/z: $[M+H]^+$ calcd. for $C_{14}H_{20}NO_2S$ 266.1209; found, 266.1202.

FT-IR (neat): 3321, 2947, 1736, 1535, 1496, 1437, 1343, 1171, 1133, 1088, 919, 700 cm^{-1}.

2-(1,3-Dioxolan-2-yl)-*N*-(3-phenylpropyl)ethanethioamide (**5d**)

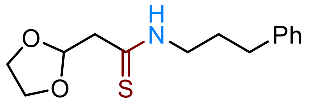

5d

Following procedure A: **5d** was isolated as yellow oil in 98% yield (52.0 mg; 0.196 mmol) which was purified by silica gel chromatography (PE:EA = 20:1~5:1, R_f = 0.5 (PE:EA = 5:1)).

^{1}H NMR (400 MHz, $CDCl_3$) δ 7.31 – 7.24 (m, 2H), 7.21 – 7.15 (m, 3H), 5.13 (t, *J* = 4.6 Hz, 1H), 3.98 – 3.84 (m, 4H), 3.70 – 3.63 (m, 2H), 3.04 (d, *J* = 4.6 Hz, 2H), 2.71 – 2.65 (m, 2H), 1.98 (p, *J* = 7.5 Hz, 2H).

^{13}C NMR (101 MHz, $CDCl_3$) δ 198.1, 141.1, 128.6, 128.5, 126.3, 102.3, 65.1, 50.9, 45.9, 33.3, 29.5.

HRMS (ESI) m/z: $[M+H]^+$ calcd. for $C_{14}H_{20}NO_2S$ 266.1209; found, 266.1199.

FT-IR (neat): 3277, 2925, 2884, 1541, 1496, 1453, 1408, 1361, 1123, 1083, 1033, 943, 909, 701 cm^{-1}.

3-Hydroxy-*N*-(3-phenylpropyl)propanethioamide (**5e**)

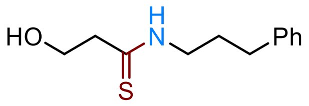

5e

Following procedure A: **5e** was isolated as yellow oil in 90% yield (40.1 mg; 0.18 mmol) which was purified by silica gel chromatography (PE:EA=5:1~1:1, R_f = 0.3 (PE:EA = 5:1)).

^{1}H NMR (400 MHz, $CDCl_3$) δ 7.81 (s, 1H), 7.33 – 7.27 (m, 2H), 7.21 (td, *J* = 7.7, 6.7, 1.5 Hz, 3H), 3.95 – 3.88 (m, 2H), 3.75 – 3.65 (m, 2H), 2.81 – 2.66 (m, 4H), 2.01 (p, *J* = 7.4 Hz, 2H).

^{13}C NMR (101 MHz, $CDCl_3$) δ 203.0, 141.1, 128.7, 128.5, 126.4, 60.7, 47.6, 45.8, 33.5, 29.5.

HRMS (ESI) m/z: $[M+H]^+$ calcd. for $C_{12}H_{18}NOS$ 224.1104; found, 224.1102.

FT-IR (neat): 3239, 2931, 2861, 1542, 1496, 1452, 1404, 1135, 1085, 1045, 914, 700 cm^{-1}.

***N*-(3-Phenylpropyl)benzothioamide** (**5f**)

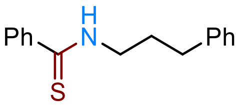

5f

Following procedure A: **5f** was isolated as yellow oil in 92% yield (46.9 mg; 0.184 mmol) which was purified by silica gel chromatography (PE:EA = 20:1~5:1, R_f = 0.6 (PE:EA = 5:1)).

^{1}H NMR (399 MHz, $CDCl_3$) δ 7.61 – 7.48 (m, 3H), 7.46 – 7.38 (m, 1H), 7.35 – 7.27 (m, 4H), 7.26 – 7.18 (m, 3H), 3.84 (td, *J* = 7.0, 5.4 Hz, 2H), 2.76 (t, *J* = 7.6 Hz, 2H), 2.09 (p, *J* = 7.1 Hz, 2H).

^{13}C NMR (101 MHz, $CDCl_3$) δ 199.1, 141.9, 141.2, 131.1, 128.8, 128.5 (2C), 126.7, 126.4, 46.8, 33.8, 29.6.

HRMS (ESI) m/z: $[M+H]^+$ calcd for $C_{16}H_{18}NS$ 256.1155; found, 256.1138.

FT-IR (neat): 3361, 2925, 2858, 1522, 1487, 1449, 1388, 1334, 1178, 1100, 1070, 1029, 943, 695 cm^{-1}.

5-Chloro-*N*-(3-phenylpropyl)pentanethioamide (**5g**)

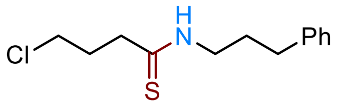

5g

Following procedure A: **5g** was isolated as yellow oil in 62% yield (31.7 mg; 0.124 mmol) which was purified by silica gel chromatography (PE:EA = 20:1~5:1, R_f = 0.45 (PE:EA = 5:1)).

^{1}H NMR (400 MHz, $CDCl_3$) δ 7.32 – 7.25 (m, 2H), 7.19 (dd, *J* = 17.8, 7.2 Hz, 3H), 3.22 (q, *J* = 5.3 Hz, 4H), 2.69 (dt, *J* = 13.7, 7.3 Hz, 4H), 2.06 (dp, *J* = 35.3, 6.8 Hz, 4H).

^{13}C NMR (101 MHz, $CDCl_3$) δ 173.2, 142.2, 128.5, 128.3, 125.7, 57.2, 38.8, 33.8, 33.8, 31.9, 27.0.

HRMS (ESI) m/z: $[M+H]^+$ calcd. for $C_{13}H_{19}ClNS$ 256.0921; found, 256.0901.

FT-IR (neat): 3268, 2934, 2886, 1538, 1495, 1453, 1409, 1121, 943, 701 cm^{-1}.

***N*-(3-phenylpropyl)thioformamide** (**5h**)

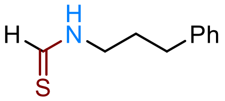

5h

Following procedure A: the thioformamide **5h** was isolated as yellow oil in 95% yield (34.0 mg; 0.19 mmol) which was purified by silica gel chromatography (PE:EA = 10:1~2:1, R_f = 0.45 (PE:EA = 2:1)).

^{1}H NMR (400 MHz, $CDCl_3$) δ 7.30 – 7.24 (m, 2H), 7.22 – 7.13 (m, 3H), 5.81 (s, 1H), 3.31 (s, 2H), 2.65 (t, *J* = 7.5 Hz, 2H), 1.88 (p, *J* = 7.2 Hz, 2H).

^{13}C NMR (101 MHz, $CDCl_3$) δ 181.4, 141.1, 128.7, 128.5, 126.3, 43.8, 33.3, 30.4.

HRMS (ESI) m/z: $[M+H]^+$ calcd. for $C_{10}H_{14}NS$ 180.0842; found, 180.0837.

FT-IR (neat): 3257, 2936, 1601, 1552, 1495, 1453, 1354, 1180, 1120, 1030, 907, 700 cm^{-1}.

2-Oxo-2-phenyl-*N*-(3-phenylpropyl)ethanethioamide (**5i**)

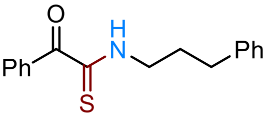

5i

Following procedure A: **5i** was isolated as yellow oil in 62% yield (35.1 mg; 0.124 mmol) which was purified by silica gel chromatography (PE:EA = 20:1~5:1, R_f = 0.45 (PE:EA = 5:1)).

^{1}H NMR (400 MHz, $CDCl_3$) δ 8.41 (s, 1H), 8.03 – 7.98 (m, 2H), 7.60 – 7.53 (m, 1H), 7.42 (t, *J* = 7.8 Hz, 2H), 7.33 – 7.26 (m, 2H), 7.20 (td, *J* = 5.2, 4.8, 2.3 Hz, 3H), 3.86 – 3.75 (m, 2H), 2.79 – 2.72 (m, 2H), 2.10 (p, *J* = 7.5 Hz, 2H).

^{13}C NMR (101 MHz, $CDCl_3$) δ 193.8, 188.0, 140.8, 134.0, 133.8, 130.8, 128.7, 128.5, 128.3, 126.4, 44.9, 33.4, 29.3.

HRMS (ESI) m/z: $[M+H]^+$ calcd. for $C_{17}H_{18}NOS$ 284.1104; found, 284.1103.

FT-IR (neat): 3281, 2925, 2861, 1663, 1594, 1530, 1448, 1399, 1177, 1119, 1065, 895, 694 cm^{-1}.

(*rac*)-2-Phenyl-*N*-(3-phenylpropyl)propanethioamide (**5j**)

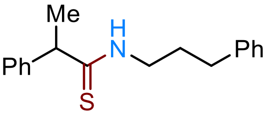

(±) 5j

Following procedure A: racemic **5j** was isolated as yellow oil in 72% yield (40.8 mg; 0.144 mmol) which was purified by silica gel chromatography (PE:EA = 20:1~5:1, Rf = 0.5 (PE:EA =5:1)).

^{1}H NMR (400 MHz, $CDCl_3$) δ 7.40 – 7.16 (m, 8H), 7.08 – 7.02 (m, 2H), 6.91 (s, 1H), 4.03 (q, *J* = 7.2 Hz, 1H), 3.61 (td, *J* = 7.0, 5.8 Hz, 2H), 2.56 – 2.49 (m, 2H), 1.86 (dt, *J* = 14.4, 7.1 Hz, 2H), 1.67 (d, *J* = 7.2 Hz, 3H).

^{13}C NMR (101 MHz, $CDCl_3$) δ 207.4, 140.9, 129.1, 128.6, 128.3, 127.8, 126.2, 54.9, 45.5, 33.2, 29.3, 21.2.

HRMS (ESI) m/z: $[M+H]^+$ calcd for $C_{18}H_{22}NS$ 284.1468; found, 284.1460.

FT-IR (neat): 3244, 2923, 1537, 1495, 1453, 1412, 1126, 1085, 699 cm^{-1}.

(*rac*)-3,3,3-Trifluoro-2-phenyl-*N*-(3-phenylpropyl)propanethioamide (5k)

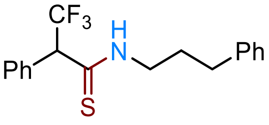

(±) 5k

Following procedure A: racemic **5k** was isolated as yellow oil in 60% yield (40.4 mg; 0.120 mmol) which was purified by silica gel chromatography (PE:EA = 20:1~5:1, R_f = 0.45 (PE:EA = 5:1)).

^{1}H NMR (400 MHz, $CDCl_3$) δ 7.42 – 7.36 (m, 4H), 7.31 – 7.17 (m, 5H), 7.13 – 7.07 (m, 2H), 4.90 (q, *J* = 9.3 Hz, 1H), 3.65 (tq, *J* = 13.4, 6.7 Hz, 2H), 2.57 (dt, *J* = 13.5, 7.6 Hz, 2H), 1.94 (p, *J* = 7.3 Hz, 2H).

^{13}C NMR (101 MHz, $CDCl_3$) δ 194.1, 140.7, 129.3, 129.3, 129.2, 129.1, 128.7, 128.6, 128.5, 128.4, 126.3, 126.1, 124.3 (d, *J* = 281.6 Hz), 64.7 (q, *J* = 26.9 Hz), 46.0, 33.1, 29.1.

^{19}F NMR (377 MHz, $CDCl_3$) δ 64.71.

HRMS (ESI) m/z: $[M+H]^+$ calcd. for $C_{18}H_{19}F_3NS$ 338.1185; found, 338.1173.

FT-IR (neat): 3276, 2927, 1558, 1540, 1496, 1456, 1363, 1162, 1116, 913, 700 cm^{-1}.

(*rac*)-*N*-(1-Phenyl-2-((3-phenylpropyl)amino)-2-thioxoethyl)acetamide (5l)

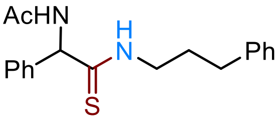

(±) 5l

Following procedure A: racemic **5l** was isolated as yellow oil in 89% yield (58.1 mg; 0.178 mmol) which was purified by silica gel chromatography (PE:EA = 20:1~5:1, R_f= 0.4 (PE:EA = 5:1)).

^{1}H NMR (400 MHz, $CDCl_3$) δ 9.69 (s, 1H), 7.73 (d, *J* = 8.0 Hz, 1H), 7.50 – 7.44 (m, 2H), 7.26 – 7.18 (m, 5H), 7.17 – 7.11 (m, 1H), 7.04 – 6.98 (m, 2H), 6.20 (d, *J* = 8.0 Hz, 1H), 3.63 – 3.40 (m, 2H), 2.51 (t, *J* = 7.7 Hz, 2H), 2.05 (s, 3H), 1.84 (dtd, *J* = 14.7, 6.3, 3.2 Hz, 2H).

^{13}C NMR (101 MHz, $CDCl_3$) δ 201.2, 169.4, 141.1, 139.6, 128.7, 128.5, 128.4, 128.2, 126.7, 126.1, 60.6, 45.7, 33.1, 29.1, 23.7.

HRMS (ESI) m/z: $[M+Na]^+$ calcd. for $C_{19}H_{22}N_2OSNa$ 349.1345; found, 349.1343.

FT-IR (neat): 3248, 2963, 1654, 1496, 1452, 1124, 1080, 969, 699 cm^{-1}.

(*rac*)-Benzyl-(1-phenyl-2-((3-phenylpropyl)amino)-2-thioxoethyl)carbamate (**5m**)

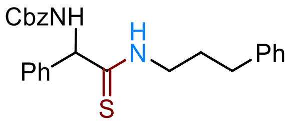

(±) 5m

Following procedure A: racemic **5m** was isolated as yellow oil in 75% yield (62.7 mg; 0.15 mmol) which was purified by silica gel chromatography (PE:EA = 20:1~5:1, R_f=0.4 (PE:EA = 5:1)).

^{1}H NMR (399 MHz, $CDCl_3$) δ 8.39 (d, J = 81.5 Hz, 1H), 7.47 – 7.13 (m, 14H), 7.05 – 6.93 (m, 2H), 6.79 (d, J = 23.9 Hz, 1H), 5.69 – 5.51 (m, 1H), 5.10 – 4.98 (m, 2H), 3.53 (dqt, J = 26.5, 13.2, 6.7 Hz, 2H), 2.48 (h, J = 7.4 Hz, 2H), 1.91 – 1.71 (m, 2H).

^{13}C NMR (100 MHz, $CDCl_3$) δ 201.1, 155.6, 140.9, 139.3, 135.9, 128.9, 128.6, 128.5, 128.5, 128.3, 127.9, 127.8, 126.8, 126.2, 67.2, 63.1, 45.6, 33.1, 29.2.

HRMS (ESI) m/z: $[M+H]^+$ calcd. for $C_{25}H_{27}N_2O_2S$ 419.1788; found, 419.1785.

FT-IR (neat): 3285, 1701, 1602, 1541, 1497, 1452, 1374, 1217, 1155, 1129, 1084, 1061, 1027, 913, 698 cm^{-1}.

***N*-(1-Benzylpiperidin-4-yl)-4-iodobenzothioamide** (**6a**)

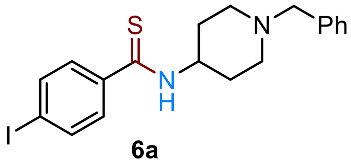

6a

Following procedure A: **6a** was isolated as yellow solid in 72% yield (62.7 mg; 0.144 mmol) which was purified by silica gel chromatography (DCM:EA = 100:1~5:1, R_f = 0.5 (PE:EA = 5:1)).

^{1}H NMR (400 MHz, $CDCl_3$) δ 7.73 (d, *J* = 1.9 Hz, 1H), 7.71 (d, *J* = 1.9 Hz, 1H), 7.43 (d, *J* = 2.2 Hz, 1H), 7.42 (d, *J* = 2.2 Hz, 1H), 7.34 – 7.26 (m, 5H), 4.59 – 4.48 (m, 1H), 3.54 (s, 2H), 2.87 (dt, *J* = 12.4, 3.4 Hz, 2H), 2.30 – 2.13 (m, 4H), 1.71 – 1.56 (m, 2H).

^{13}C NMR (101 MHz, $CDCl_3$) δ 197.1, 141.5, 138.0, 137.7, 129.3, 128.4, 128.3, 127.4, 97.9, 63.1, 53.3, 52.1, 30.8.

HRMS (ESI) m/z: $[M+H]^+$ calcd. for $C_{19}H_{22}IN_2S$ 437.0543; found, 437.0532.

FT-IR (neat): 2921, 1580, 1517, 1478, 1398, 1140, 1079, 1005, 943, 825, 697 cm^{-1}.

<u>*N*-(2,6-Dioxopiperidin-3-yl)-2-(4-hydroxyphenyl)ethanethioamide</u> (**6b**)

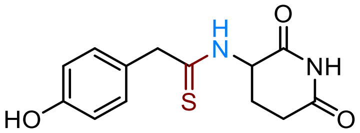

6b

Following procedure A: **6b** was isolated as yellow solid in 88% yield (48.9 mg; 0.176 mmol) which was purified by silica gel chromatography (PE:EA = 5:1~1:1, R_f = 0.2 (PE:EA = 5:1)).

^{1}H NMR (400 MHz, DMSO-d_6) δ 10.99 (s, 1H), 10.37 (d, *J* = 8.2 Hz, 1H), 9.27 (s, 1H), 7.13 (d, *J* = 8.5 Hz, 2H), 6.68 (d, *J* = 8.5 Hz, 2H), 5.34 (ddd, *J* = 12.9, 8.0, 5.2 Hz, 1H), 3.83 (s, 2H), 2.73 (ddd, *J* = 18.4, 13.3, 5.5 Hz, 1H), 2.53 (t, *J* = 3.8 Hz, 1H), 2.14 – 2.03 (m, 1H), 1.90 (qd, *J* = 12.6, 4.7 Hz, 1H).

^{13}C NMR (101 MHz, DMSO-d_6) δ 203.5, 173.2, 171.5, 156.6, 130.3, 128.0, 115.5, 55.2, 51.0, 31.1, 23.4.

HRMS (ESI) m/z: $[M+H]^+$ calcd. for $C_{13}H_{15}N_2O_3S$ 279.0798; found, 279.0916.

FT-IR (neat): 3234, 2860, 1730, 1682, 1530, 1512, 1453, 1330, 1120, 713 cm^{-1}.

<u>1-(4-(8-Chlorodibenzo[b,f][1,4]oxazepin-11-yl)piperazin-1-yl)-4-phenylbutane-1-thione</u> (**6c**)

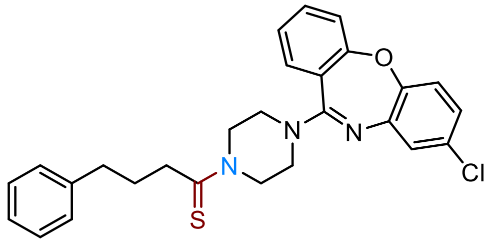

6c

Following procedure A: **6c** was isolated as yellow solid in 90% yield (85.5 mg; 0.180 mmol) which was purified by silica gel chromatography (PE:EA = 20:1~5:1, R_f=0.3 (PE:EA = 5:1)).

^{1}H NMR (400 MHz, $CDCl_3$) δ 7.40 (dd, *J* = 8.7, 2.6 Hz, 1H), 7.32 – 7.24 (m, 3H), 7.22 – 7.13 (m, 5H), 7.13 – 7.07 (m, 2H), 7.05 – 6.99 (m, 1H), 4.38 (s, 2H), 3.64 (d, *J* = 56.9 Hz, 6H), 2.92 – 2.84 (m, 2H), 2.72 (t, *J* = 7.4 Hz, 2H), 2.11 – 2.00 (m, 2H).

^{13}C NMR (101 MHz, $CDCl_3$) δ 203.8, 159.5, 158.4, 151.8, 141.3, 139.7, 133.2, 130.6, 128.9, 128.6, 128.6, 127.2, 126.3, 126.1, 125.3, 124.6, 123.1, 120.4, 60.5, 49.4, 48.9, 47.2, 43.1, 35.4, 30.9.

HRMS (ESI) m/z: $[M+H]^+$ calcd. for $C_{27}H_{27}ClN_3OS$ 476.1558; found, 476.1559.

FT-IR (neat): 2926, 1602, 1588, 1559, 1470, 1433, 1187, 1111, 1002, 700 cm^{-1}.

4-Cyclopropyl-6-fluoro-1-oxo-7-(4-(4-phenylbutanethioyl)piperazin-1-yl)-1,4-dihydronaphthalene-2-carboxylic acid (6d)

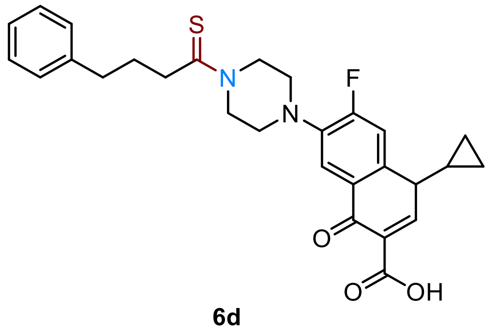

6d

Following procedure A: **6d** was isolated as yellow solid in 56% yield (55.1 mg; 0.112 mmol) which was purified by silica gel chromatography (DCM: MeOH = 100:1~20:1, R_f = 0.5 (DCM: MeOH = 20:1)).

^{1}H NMR (400 MHz, $CDCl_3$) δ 14.88 (s, 1H), 8.68 (s, 1H), 7.95 (d, *J* = 12.9 Hz, 1H), 7.35 – 7.27 (m, 3H), 7.23 – 7.19 (m, 2H), 4.59 – 4.54 (m, 1H), 3.89 – 3.85 (m, 1H), 3.56 (dp, *J* = 7.1, 4.1 Hz, 1H), 3.47 – 3.39 (m, 3H), 2.96 – 2.84 (m, 2H), 2.75 (t, *J* = 7.6 Hz, 2H), 2.15 – 1.97 (m, 2H), 1.43 – 1.13 (m, 6H).

^{13}C NMR (101 MHz, $CDCl_3$) δ 204.0, 177.0, 166.8, 152.2, 147.6, 144.82 (d, *J* = 9.8 Hz), 141.3, 139.1, 128.6, 128.6, 126.3, 120.20 (d, *J* = 7.8 Hz), 112.55 (d, *J* = 23.1 Hz), 108.1, 105.0, 49.9, 49.0, 48.6, 42.9, 35.5, 35.4, 30.9, 8.4.

^{19}F NMR (377 MHz, $CDCl_3$) δ -121.22.

HRMS (ESI) m/z: $[M+H]^+$ calcd. for $C_{28}H_{30}FN_2O_3S$ 493.1956; found, 493.1948.

FT-IR (neat): 3008, 2920, 1723, 1628, 1508, 1491, 1467, 1110, 892, 704 cm^{-1}.

1-(4-(8-Chloro-5,6-dihydro-11*H*-benzo[5,6]cyclohepta[1,2-b]pyridin-11-ylidene)piperidin-1-yl)-4-phenylbutane-1-thione (**6e**)

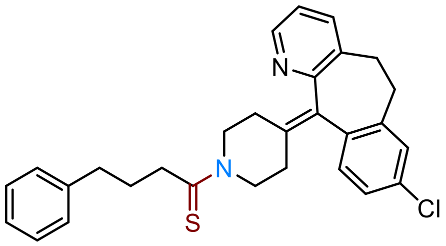

6e

Following procedure A: **6e** was isolated as yellow solid in 86% yield (81.2 mg; 0.172 mmol) which was purified by silica gel chromatography (PE:EA = 20:1~5:1, R_f=0.3 (PE:EA = 5:1)).

^{1}H NMR (400 MHz, $CDCl_3$) δ 8.40 (d, *J* = 4.9 Hz, 1H), 7.45 (ddd, *J* = 8.0, 3.3, 1.8 Hz, 1H), 7.29 – 7.23 (m, 3H), 7.20 – 7.07 (m, 7H), 4.71 (dt, *J* = 13.2, 5.2 Hz, 1H), 3.89 – 3.71 (m, 2H), 3.50 – 3.24 (m, 3H), 2.93 – 2.65 (m, 7H), 2.62 – 2.31 (m, 3H), 2.11 – 1.94 (m, 2H).

^{13}C NMR (101 MHz, $CDCl_3$) δ 202.6, 156.6, 146.8, 141.4, 139.6, 138.0, 137.3, 135.4, 133.6, 130.5, 130.3, 129.2, 128.5, 126.4, 126.1, 122.6, 50.9, 49.4, 43.3, 35.5, 31.7, 31.2, 30.8, 29.8, 29.5.

HRMS (ESI) m/z: $[M+H]^+$ calcd. for $C_{29}H_{30}ClN_2S$ 473.1813; found, 473.1804.

FT-IR (neat): 2919, 2861, 1589, 1560, 1494, 1478, 1457, 1437, 1361, 1100, 990, 701 cm^{-1}.

1.3.4 General synthesis of thiopeptides from chiral nitro compounds and chiral amino acid esters.

Supplementary Table S2. Optimization of reaction conditions with β-amino chiral nitroalkanes[a]

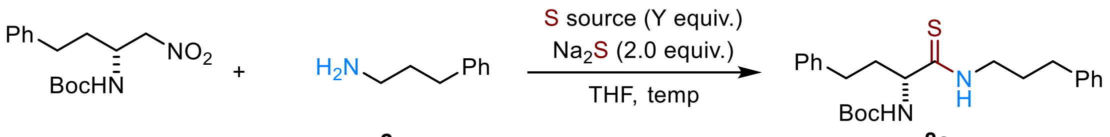

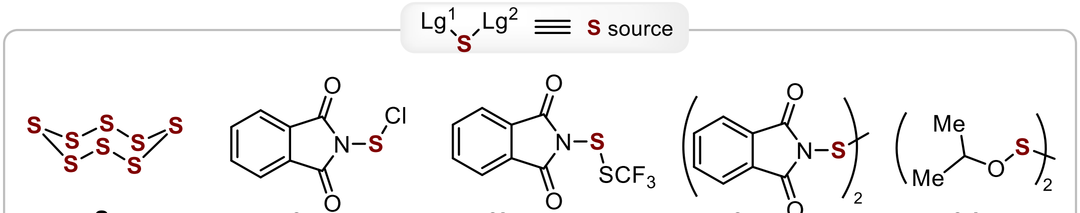

entry	S source (equiv.)	Temperature	time (h)	yield (%)	ee (%)
1	**S_8** (1.25)	rt	24	96	76
2	**S_8** (1.25)	50°C	24	99	45
3	**S_8** (1.25)	0°C	36	72	98
4	**S_8** (2.0)	0°C	36	74	98
5	**S_8** (2.0)	-10°C	36	40	99
6	**3a** (2)	-10°C	36	trace	-
7	**3b** (2)	-10°C	36	trace	-
8	**3c** (2)	-10°C	36	37	99
9	**3d** (2)	-10°C	36	81	99
10	**3d** (2)	-10°C	24	73	99
11	**3d** (2)	-15°C	36	80	99

[a]Unless noted otherwise, reactions were carried out with 0.1 mmol of **7a**, 0.2 mmol of **2a**, 0.2 mmol S source and 0.2 mmol of Na_2S in 1 mL of THF. [b]Yield of isolated product.

General procedure B: The chiral *β*-amino nitro compound **7** (0.1 mmol) was added to a 10 mL reaction tube, followed by the addition of THF (1 mL) and cooled down to -10℃. Next Na_2S (2.0 equiv., 0.2 mmol), **3d** (2.0 equiv.) and the amine **9** (2.0 equiv.) were added and stirred at -10℃. The reaction was monitored by TLC until the reaction was complete (typically within 36h), quenched with saturated ammonium chloride, and the crude product was extracted with ethyl acetate, and the organic phase collected and dried over anhydrous Na_2SO_4. After filtration, the solution was concentrated under reduced pressure and the crude residue was separated by flash-column chromatography.

***tert*-Butyl (*R*)-(4-phenyl-1-((3-phenylpropyl)amino)-1-thioxobutan-2-yl)carbama-te (8a)**

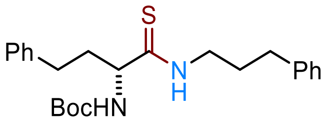

8a

Following procedure B: **8a** was isolated as colorless oil in 81% yield (33.4 mg; 0.081 mmol) which was purified by silica gel chromatography (PE:EA = 20:1~5:1, R_f = 0.45 (PE:EA = 5:1)). $[\alpha]_{D}^{25}$= 15.6 (*c* = 1.00, $CHCl_3$). ee = 99% (Chiralpak AD-H, hexane/*i*-PrOH=95:5, 214 nm, 1 mL/min, t_{major}= 21.47 min, t_{minor}= 14.32 min).

^{1}H NMR (400 MHz, $CDCl_3$) δ 8.51 (s, 1H), 7.30 – 7.11 (m, 11H), 5.45 (d, *J* = 8.7 Hz, 1H), 4.36 (q, *J* = 8.2 Hz, 1H), 3.63 (ddt, *J* = 26.5, 13.1, 6.8 Hz, 2H), 2.64 (t, *J* = 7.6 Hz, 4H), 2.20 – 1.85 (m, 4H), 1.41 (s, 9H).

^{13}C NMR (101 MHz, $CDCl_3$) δ 204.3, 156.0, 141.1, 140.9, 128.7, 128.6, 128.6, 128.5, 126.5, 126.2, 80.4, 60.3, 45.4, 37.3, 33.4, 32.3, 29.4, 28.5.

HRMS (ESI) m/z: $[M+Na]^+$ calcd. for $C_{24}H_{32}N_2O_2SNa$ 435.2077; found, 435.2072.

FT-IR (neat): 3274, 3003, 2986, 1692, 1553, 1496, 1454, 1366, 1166, 1129, 1051, 699 cm^{-1}.

HPLC traces of compound **8a**:

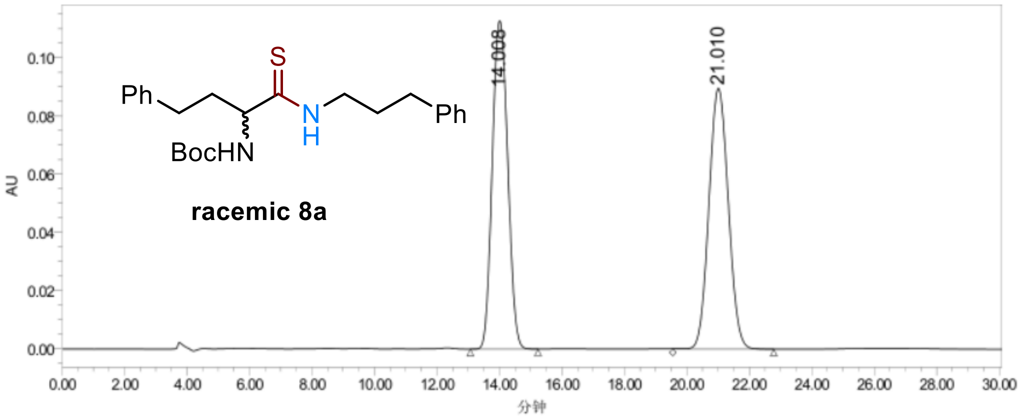

Channel: W2489 ChA; Channel Desc.: W2489 ChA 254nm; Processing Method: 0

	Channel Description	RT (min)	Area (礦*sec)	% Area	Height (礦)
1	W2489 ChA 254nm	14.008	3858020	49.95	112780
2	W2489 ChA 254nm	21.010	3865105	50.05	89595

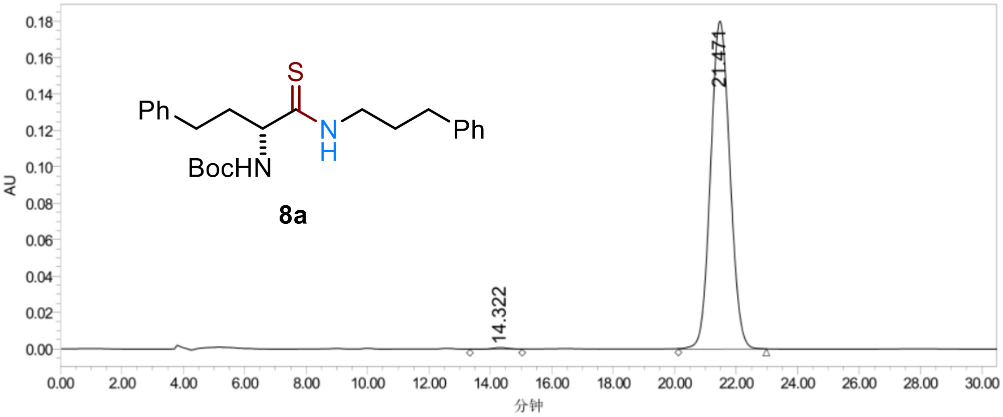

Channel: W2489 ChA; Channel Desc.: W2489 ChA 254nm; Processing Method: 0

	Channel Description	RT (min)	Area (礦*sec)	% Area	Height (礦)
1	W2489 ChA 254nm	14.322	41097	0.52	900
2	W2489 ChA 254nm	21.471	7869211	99.48	180159

<u>*tert*-Butyl (*R*)-(1-phenyl-2-((3-phenylpropyl)amino)-2-thioxoethyl)carbamate</u> (**8b**)

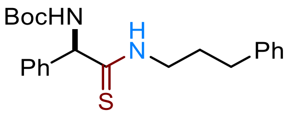

8b

Followed the general procedure B (at -30℃): **8b** was isolated as yellow oil in 49% yield. The ee value was 86% (Chiralpak AD-H, hexane/*i*-PrOH=95:5, 254 nm, 1 mL/min, t_{major}= 28.95 min, t_{minor}= 14.37 min).

^{1}H NMR (400 MHz, $CDCl_3$) δ 7.94 (s, 1H), 7.41 – 7.15 (m, 10H), 7.12 – 7.05 (m, 2H), 6.15 (s, 1H), 5.40 (d, *J* = 6.4 Hz, 1H), 3.64 (d, *J* = 26.4, 6.8 Hz, 2H), 2.55 (t, *J* = 7.6 Hz, 2H), 1.90 (dh, *J* = 13.7, 6.4 Hz, 2H), 1.41 (s, 9H).

^{13}C NMR (101 MHz, $CDCl_3$) δ 201.8, 155.2, 141.0, 139.5, 129.0, 128.6, 128.5, 127.0, 126.3, 80.5, 63.6, 45.6, 33.3, 29.3, 28.4.

HRMS (ESI) m/z: $[M+Na]^+$ calcd. for $C_{22}H_{28}N_2O_2S$ 407.1764; found, 407.1782.

FT-IR (neat): 3279, 2976, 1689, 1553, 1495, 1454, 1366, 1165, 1058, 699 cm^{-1}.

HPLC traces of compound **8b**:

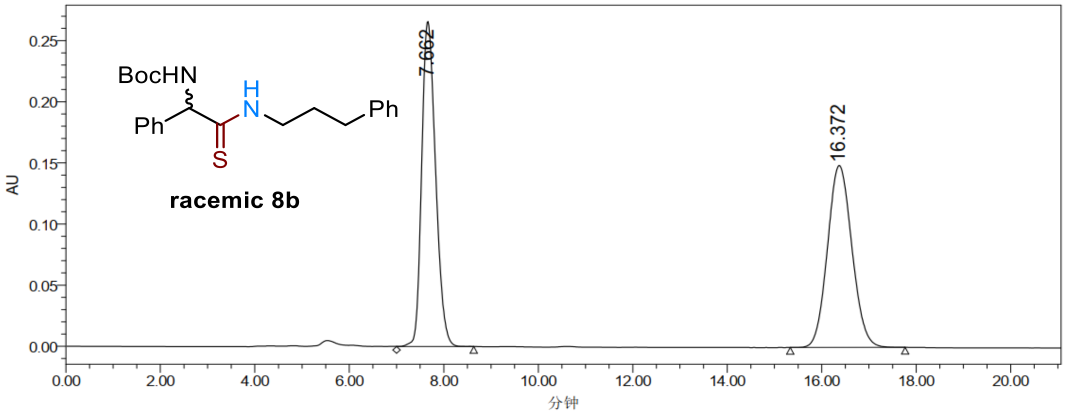

Channel: W2489 ChB; Channel Desc.: W2489 ChB 254nm; Processing Method: 1

	Channel Description	RT (min)	Area (礦*sec)	% Area	Height (礦)
1	W2489 ChB 254nm	7.662	5414338	50.28	265937
2	W2489 ChB 254nm	16.372	5354513	49.72	148881

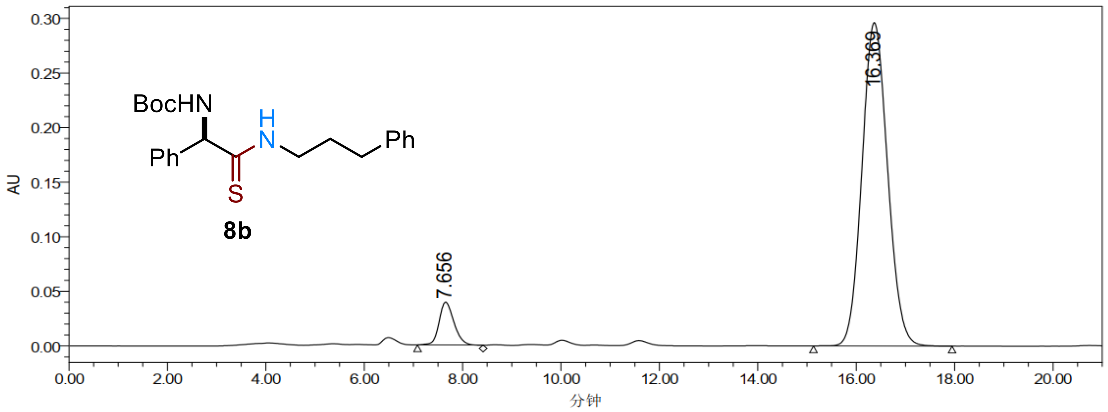

Channel: W2489 ChB; Channel Desc.: W2489 ChB 254nm; Processing Method: 1

	Channel Description	RT (min)	Area (礦*sec)	% Area	Height (礦)
1	W2489 ChB 254nm	7.656	824480	7.15	39245
2	W2489 ChB 254nm	16.369	10714201	92.85	296243

tert-Butyl ((_R_)-2-((_tert_-butoxycarbonyl)amino)-4-phenylbutanethioyl)-_L_-phenylalaninate (10a)

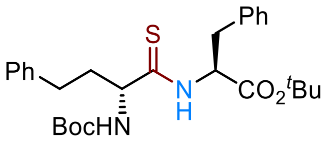

10a

Following procedure B: **10a** was isolated as yellow oil in 83% yield (41.0 mg; 0.083 mmol) which was purified by silica gel chromatography (PE:EA = 20:1~5:1, R_f = 0.55 (PE:EA = 5:1)).

$[\alpha]_{25\ D}$= 25.2 (c = 1.20, $CHCl_3$)

^{1}H NMR (400 MHz, $CDCl_3$) δ 8.26 (s, 1H), 7.34 – 7.04 (m, 11H), 5.25 – 5.12 (m, 2H), 4.46 – 4.26 (m, 1H), 3.25 (q, J = 7.8 Hz, 2H), 2.68 – 2.54 (m, 2H), 2.22 (dq, J = 14.3, 6.4 Hz, 1H), 1.97 (td, J = 14.8, 8.7 Hz, 1H), 1.42 (s, 9H), 1.39 (s, 10H).

^{13}C NMR (101 MHz, $CDCl_3$) δ 203.9, 169.6, 155.5, 140.9, 135.8, 129.5, 128.6, 128.6, 128.5, 127.2, 126.2, 83.1, 80.3, 60.7, 58.8, 37.5, 36.4, 32.1, 28.4, 28.0.

HRMS (ESI) m/z: $[M+Na]^+$ calcd. for $C_{28}H_{38}N_2O_4SNa$ 521.2445; found, 521.2446.

FT-IR (neat): 3293, 2978, 1729, 1700, 1603, 1512, 1497, 1454, 1367, 1156, 699 cm^{-1}.

***tert*-Butyl ((*R*)-2-(((benzyloxy)carbonyl)amino)-4-phenylbutanethioyl)-*L*-phenylalaninate (10b)**

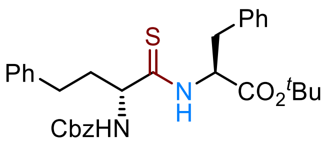

10b

Following procedure B: **10b** was isolated as yellow oil in 75% yield (40.0 mg; 0.075 mmol) which was purified by silica gel chromatography (PE:EA = 20:1~5:1, R_f = 0.5 (PE:EA = 5:1)).

$[\alpha]_{25\ D}$= 23.4 (c = 1.10, $CHCl_3$).

^{1}H NMR (400 MHz, $CDCl_3$) δ 8.22 (s, 1H), 7.38 – 7.29 (m, 5H), 7.26 – 7.08 (m, 10H), 5.60 (d, J = 8.5 Hz, 1H), 5.20 (q, J = 6.0 Hz, 1H), 5.15 – 5.00 (m, 2H), 4.56 – 4.42 (m, 1H), 3.37 – 3.14 (m, 2H), 2.66 – 2.54 (m, 2H), 2.17 (q, J = 8.0 Hz, 1H), 2.06 – 1.92 (m, 1H), 1.40 (s, 9H).

^{13}C NMR (101 MHz, $CDCl_3$) δ 203.5, 169.6, 156.0, 140.8, 136.3, 135.8, 129.5, 128.7, 128.6, 128.6, 128.5, 128.3, 128.2, 127.3, 126.3, 83.2, 67.2, 61.0, 58.8, 37.7, 36.3, 31.9, 28.0.

HRMS (ESI) m/z: $[M+Na]^+$ calcd. for $C_{31}H_{36}N_2O_4SNa$ 555.2288; found, 555.2298.

FT-IR (neat): 3331, 3294, 2977, 1728, 1708, 1655, 1603, 1510, 1497, 1454, 1422, 1368, 1153, 1096, 1050, 911, 844, 698 cm^{-1}.

tert-Butyl (R)-(2-((tert-butoxycarbonyl)amino)-4-phenylbutanethioyl)glycinate (10c)

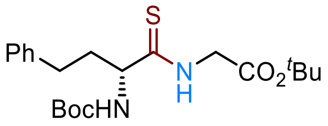

10c

Following procedure B: **10c** was isolated as yellow oil in 58% yield (23.7 mg; 0.058 mmol) which was purified by silica gel chromatography (PE:EA = 20:1~5:1, R_f = 0.55 (PE:EA = 5:1)).

$[\alpha]_{25\ D}$= 17.8 (*c* = 1.30, $CHCl_3$).

^{1}H NMR (400 MHz, $CDCl_3$) δ 8.53 (s, 1H), 7.30 – 7.23 (m, 2H), 7.21 – 7.14 (m, 3H), 5.37 (s, 1H), 4.46 (d, *J* = 6.6 Hz, 1H), 4.31 (dd, *J* = 19.0, 4.9 Hz, 1H), 4.16 (d, *J* = 4.7 Hz, 1H), 2.74 – 2.63 (m, 2H), 2.24 (d, *J* = 6.6 Hz, 1H), 2.05 (dt, *J* = 13.7, 7.4 Hz, 1H), 1.47 (s, 9H), 1.44 (s, 9H).

^{13}C NMR (101 MHz, $CDCl_3$) δ 205.0, 167.5, 155.7, 140.9, 128.6, 128.5, 126.2, 83.0, 80.4, 60.4, 47.8, 37.5, 32.2, 28.4, 28.1.

HRMS (ESI) m/z: $[M+Na]^+$ calcd. for $C_{21}H_{32}N_2O_4SNa$ 431.1975; found, 431.1958.

FT-IR (neat): 3270, 2978, 1743, 1688, 1497, 1455, 1367, 1157, 700 cm^{-1}.

tert-Butyl ((R)-2-((tert-butoxycarbonyl)amino)-4-phenylbutanethioyl)-L-alaninate (10d)

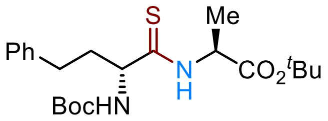

10d

Following procedure B: **10d** was isolated as light yellow oil in 84% yield (35.4 mg; 0.084 mmol) which was purified by silica gel chromatography (PE:EA = 20:1~5:1, R_f = 0.5 (PE:EA = 5:1)).

$[\alpha]_{25\ D}$= 17.1 (*c* = 0.90, $CHCl_3$).

^{1}H NMR (400 MHz, $CDCl_3$) δ 8.58 (s, 1H), 7.31 – 7.24 (m, 3H), 7.20 – 7.16 (m, 3H), 5.41 (s, 1H), 4.93 (p, *J* = 7.0 Hz, 1H), 4.58 – 4.34 (m, 1H), 2.69 (dt, *J* = 16.5, 8.1 Hz, 2H), 2.21 (s, 1H), 2.04 (tt, *J* = 14.0, 7.1 Hz, 1H), 1.49 – 1.45 (m, 12H), 1.44 (s, 9H).

^{13}C NMR (101 MHz, $CDCl_3$) δ 203.8, 171.2, 155.6, 141.0, 128.6, 128.5, 126.2, 82.7, 80.3, 60.4, 53.8, 37.5, 32.2, 28.4, 28.0, 16.9.

HRMS (ESI) m/z: $[M+Na]^+$ calcd. for $C_{22}H_{34}N_2O_4SNa$ 445.2132; found, 445.2123.

FT-IR (neat): 3301, 2976, 1740, 1698, 1603, 1555, 1503, 1454, 1366, 1164, 701 cm^{-1}.

tert-Butyl _S_-benzyl-_N_-((_R_)-2-((_tert_-butoxycarbonyl)amino)-4-phenylbutanethio-yl)-_L_-cysteinate (**10e**)

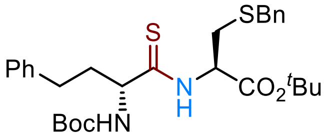

10e

Following procedure B: **10e** was isolated as yellow oil in 62% yield (33.7 mg; 0.062 mmol) which was purified by silica gel chromatography (PE:EA = 20:1~5:1, R_f= 0.6 (PE:EA = 5:1)).

$[\alpha]_{25\ D}$= 5.6 (*c* = 0.80, $CHCl_3$).

^{1}H NMR (400 MHz, $CDCl_3$) δ 8.46 (s, 1H), 7.71 – 7.63 (m, 1H), 7.47 (ddd, *J* = 8.4, 6.7, 3.0 Hz, 1H), 7.28 (d, *J* = 4.0 Hz, 5H), 7.25 – 7.15 (m, 4H), 5.17 (dd, *J* = 13.3, 8.3 Hz, 2H), 4.43 (s, 1H), 3.70 (d, *J* = 1.9 Hz, 2H), 3.18 (dd, *J* = 13.7, 4.9 Hz, 1H), 2.91 (dd, *J* = 13.7, 5.2 Hz, 1H), 2.70 (t, *J* = 7.9 Hz, 2H), 2.36 – 2.21 (m, 1H), 2.04 (dq, *J* = 15.3, 7.9 Hz, 1H), 1.45 (d, *J* = 7.1 Hz, 18H).

^{13}C NMR (101 MHz, $CDCl_3$) δ 204.4, 168.7, 137.7, 132.3, 132.2, 132.1, 129.1, 128.7, 128.5, 127.3, 126.3, 83.5, 80.4, 61.0, 57.4, 37.3, 36.9, 32.1, 31.9, 29.8, 28.4, 28.0.

HRMS (ESI) m/z: $[M+Na]^+$ calcd for $C_{29}H_{40}N_2O_4S_2Na$ 567.2322; found, 567.2326.

FT-IR (neat): 3392, 3327, 2976, 1739, 1699, 1602, 1553, 1496, 1454, 1391, 1367, 1165, 1120, 1048, 1028, 913, 699 cm^{-1}.

***tert*-Butyl ((*R*)-2-((*tert*-butoxycarbonyl)amino)-4-phenylbutanethioyl)-*L*-methion-inate (10f)**

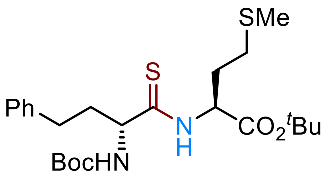

10f

Following procedure B: **10f** was isolated as light yellow oil in 74% yield (35.6 mg; 0.074 mmol) which was purified by silica gel chromatography (PE:EA = 20:1~5:1, R_f = 0.5 (PE:EA = 5:1)).

$[\alpha]_{25\,D}$= 30.6 (*c* = 1.60, $CHCl_3$).

^{1}H NMR (400 MHz, $CDCl_3$) δ 8.61 (s, 1H), 7.32 – 7.27 (m, 2H), 7.21 – 7.16 (m, 3H), 5.23 (d, *J* = 7.7 Hz, 1H), 5.12 – 5.03 (m, 1H), 4.42 (s, 1H), 2.74 – 2.62 (m, 2H), 2.50 (ddd, *J* = 8.2, 6.6, 3.0 Hz, 2H), 2.38 – 2.23 (m, 2H), 2.20 – 2.10 (m, 1H), 2.08 (s, 3H), 2.07 – 1.99 (m, 1H), 1.48 (s, 9H), 1.44 (s, 9H).

^{13}C NMR (101 MHz, $CDCl_3$) δ 204.1, 169.9, 155.5, 140.8, 128.7, 128.5, 126.3, 83.2, 80.5, 61.0, 57.4, 37.1, 32.2, 30.4, 29.7, 28.4, 28.1, 15.6.

HRMS (ESI) m/z: $[M+Na]^+$ calcd. for $C_{24}H_{38}N_2O_4S_2Na$ 505.2165; found, 505.2160.

FT-IR (neat): 3295, 2977, 1724, 1698, 1553, 1497, 1453, 1367, 1158, 699 cm^{-1}.

***tert*-Butyl ((*R*)-2-((*tert*-butoxycarbonyl)amino)-4-phenylbutanethioyl)-*L*-leucinate (10g)**

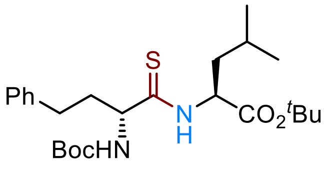

10g

Following procedure B: **10g** was isolated as yellow oil in 78% yield (36.2 mg; 0.078 mmol) which was purified by silica gel chromatography (PE:EA = 20:1~5:1, R_f = 0.45 (PE:EA = 5:1)).

$[\alpha]_{25\,D}$= 13.6 (*c* = 1.00, $CHCl_3$).

^{1}H NMR (400 MHz, $CDCl_3$) δ 8.49 (s, 1H), 7.31 – 7.24 (m, 2H), 7.22 – 7.15 (m, 3H), 5.31 (s, 1H), 5.02 – 4.94 (m, 1H), 4.43 (d, *J* = 7.7 Hz, 1H), 2.76 – 2.58 (m, 2H), 2.25 (d, *J* = 14.0 Hz, 1H), 2.11 – 1.98 (m, 1H), 1.77 – 1.68 (m, 2H), 1.44 (d, *J* = 8.5 Hz, 18H), 0.95 (dd, *J* = 12.1, 6.3 Hz, 6H).

^{13}C NMR (101 MHz, $CDCl_3$) δ 204.1, 170.8, 155.6, 141.0, 128.6, 128.5, 126.2, 82.5, 80.4, 60.9, 57.0, 40.4, 37.0, 32.2, 28.4, 28.1, 25.1, 22.7, 22.5.

HRMS (ESI) m/z: $[M+Na]^+$ calcd. for $C_{25}H_{40}N_2O_4SNa$ 487.2601; found, 487.2603.

FT-IR (neat): 3304, 2976, 1727, 1697, 1553, 1497, 1454, 1367, 1159, 699 cm^{-1}.

<u>*tert*-Butyl ((*R*)-2-((*tert*-butoxycarbonyl)amino)-4-phenylbutanethioyl)-*L*-alloisoleucinate</u> (10h)

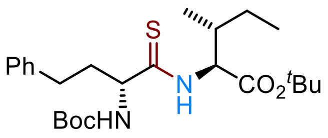

10h

Following procedure B: **10h** was isolated as yellow oil in 72% yield (33.4 mg; 0.072 mmol) which was purified by silica gel chromatography (PE:EA = 20:1~5:1, R_f = 0.55 (PE:EA = 5:1)).

$[\alpha]_{25}$ $_D$= 7.6 (*c* = 0.80, $CHCl_3$).

^{1}H NMR (400 MHz, $CDCl_3$) δ 8.49 (s, 1H), 7.31 – 7.25 (m, 2H), 7.19 (td, *J* = 7.2, 1.4 Hz, 3H), 5.21 (s, 1H), 5.06 – 4.98 (m, 1H), 4.40 (s, 1H), 2.77 – 2.61 (m, 2H), 2.33 (dd, *J* = 14.2, 6.1 Hz, 1H), 2.18 – 1.98 (m, 2H), 1.64 – 1.52 (m, 1H), 1.48 (s, 9H), 1.44 (s, 9H), 1.36 – 1.20 (m, 2H), 0.98 (t, *J* = 7.4 Hz, 3H), 0.90 (d, *J* = 6.9 Hz, 3H).

^{13}C NMR (101 MHz, $CDCl_3$) δ 203.5, 169.7, 155.5, 140.9, 128.6, 128.5, 126.3, 82.8, 80.5, 61.7, 61.1, 37.2, 37.0, 32.2, 28.4, 28.2, 26.3, 15.2, 11.9.

HRMS (ESI) m/z: $[M+H]^+$ calcd. for $C_{25}H_{41}N_2O_4S$ 465.2782; found, 465.2773.

FT-IR (neat): 3310, 2974, 1724, 1701, 1497, 1454, 1366, 1159, 699 cm^{-1}.

<u>*tert*-Butyl ((*R*)-2-((*tert*-butoxycarbonyl)amino)-4-phenylbutanethioyl)-*L*-valinate</u> (10i)

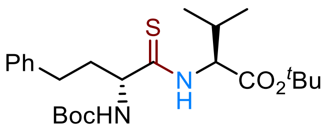

10i

Following procedure B: **10i** was isolated as yellow oil in 68% yield (30.6 mg; 0.068 mmol) which was purified by silica gel chromatography (PE:EA = 20:1~5:1, R_f = 0.55 (PE:EA = 5:1)).

$[\alpha]_{25\ D}$= 29.6 (*c* = 1.30, $CHCl_3$).

^1H NMR (400 MHz, $CDCl_3$) δ 8.52 (s, 1H), 7.28 (td, *J* = 5.4, 2.5 Hz, 2H), 7.19 (td, *J* = 6.3, 1.8 Hz, 3H), 5.25 (d, *J* = 8.5 Hz, 1H), 4.96 (dd, *J* = 8.0, 4.1 Hz, 1H), 4.43 (s, 1H), 2.36 (ddt, *J* = 14.8, 12.4, 7.4 Hz, 2H), 2.14 – 1.98 (m, 1H), 1.48 (s, 9H), 1.44 (s, 9H), 1.03 (d, *J* = 6.9 Hz, 3H), 0.95 (d, *J* = 6.9 Hz, 3H).

^{13}C NMR (101 MHz, $CDCl_3$) δ 204.2, 169.7, 155.6, 140.9, 128.6, 128.5, 126.3, 82.7, 80.4, 62.8, 61.2, 37.0, 32.2, 30.9, 28.4, 28.2, 18.7, 18.4.

HRMS (ESI) m/z: $[M+Na]^+$ calcd. for $C_{24}H_{38}N_2O_4SNa$ 473.2445; found, 473.2452.

FT-IR (neat): 3311, 2976, 1735, 1701, 1497, 1454, 1366, 1158, 702 cm^{-1}.

<u>*tert*-Butyl ((*R*)-2-((tert-butoxycarbonyl)amino)-4-phenylbutanethioyl)-*L*-tyrosina-te</u> (**10j**)

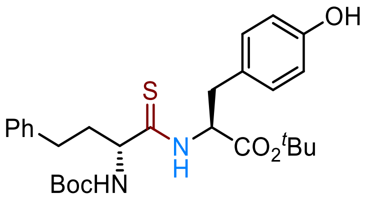

10j

Following procedure B: **10j** was isolated as yellow oil in 63% yield (32.4 mg; 0.063 mmol) which was purified by silica gel chromatography (PE:EA = 20:1~5:1, R_f = 0.3 (PE:EA = 5:1)).

$[\alpha]_{25\ D}$= 15.8 (*c* = 1.00, $CHCl_3$).

^1H NMR (400 MHz, $CDCl_3$) δ 8.39 (s, 1H), 7.25 (d, *J* = 14.6 Hz, 2H), 7.20 – 7.09 (m, 3H), 6.99 – 6.94 (m, 2H), 6.68 (d, *J* = 8.5 Hz, 2H), 5.39 (d, *J* = 8.5 Hz, 1H), 5.17 (q, *J* = 6.7 Hz,

1H), 4.41 (s, 1H), 3.25 – 3.08 (m, 2H), 2.59 (s, 2H), 2.22 (dq, *J* = 15.1, 7.7 Hz, 1H), 1.96 (p, *J* = 8.8 Hz, 1H), 1.44 (s, 9H), 1.41 (s, 9H).

^{13}C NMR (101 MHz, $CDCl_3$) δ 203.9, 170.0, 155.7, 155.4, 140.9, 130.6, 128.6, 128.5, 127.1, 126.2, 115.7, 83.2, 80.6, 60.6, 59.1, 37.5, 35.6, 32.1, 28.4, 28.1.

HRMS (ESI) m/z: $[M+Na]^+$ calcd. for $C_{28}H_{38}N_2O_5SNa$ 537.2394; found, 537.2392.

FT-IR (neat): 3334, 2978, 1724, 1698, 1614, 1515, 1454, 1367, 1156, 700 cm^{-1}.

<u>*tert*-Butyl (*S*)-3-(4-(*tert*-butoxy)phenyl)-2-((*R*)-2-((*tert*-butoxycarbonyl)amino)-4-phenylbutanethioamido)propanoate</u> (10k)

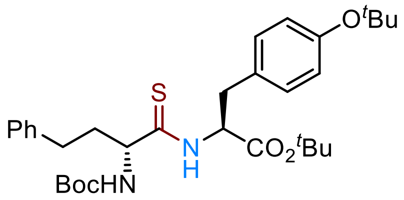

10k

Following procedure B: **10k** was isolated as yellow oil in 75% yield (42.3 mg; 0.075 mmol) which was purified by silica gel chromatography (PE:EA = 20:1~5:1, R_f = 0.5 (PE:EA = 5:1)).

$[\alpha]_{25}$ $_D$= 12.4 (c = 1.50, $CHCl_3$).

^{1}H NMR (400 MHz, $CDCl_3$) δ 8.33 (s, 1H), 7.30 – 7.26 (m, 2H), 7.21 – 7.14 (m, 3H), 7.08 – 7.03 (m, 2H), 6.89 – 6.85 (m, 2H), 5.25 (d, J = 7.4 Hz, 1H), 5.21 – 5.13 (m, 1H), 4.40 (s, 1H), 3.20 (t, J = 4.8 Hz, 2H), 2.68 (dt, J = 22.3, 8.0 Hz, 2H), 2.35 – 2.17 (m, 1H), 2.06 – 1.76 (m, 2H), 1.44 (s, 9H), 1.37 (s, 9H), 1.30 (s, 9H).

^{13}C NMR (101 MHz, $CDCl_3$) δ 203.8, 169.7, 154.5, 140.9, 130.7, 130.0, 128.7, 128.6, 128.5, 126.5, 126.2, 124.3, 83.1, 80.4, 78.5, 60.6, 59.0, 37.5, 35.9, 32.1, 28.9, 28.4, 28.0.

HRMS (ESI) m/z: $[M+Na]^+$ calcd. for $C_{32}H_{46}N_2O_5SNa$ 593.3020; found, 593.3026.

FT-IR (neat): 3326, 2977, 1718, 1701, 1607, 1554, 1506, 1454, 1366, 1160, 700 cm^{-1}.

<u>Methyl ((*R*)-2-((*tert*-butoxycarbonyl)amino)-4-phenylbutanethioyl)-*L*-tryptophanate</u> (10l)

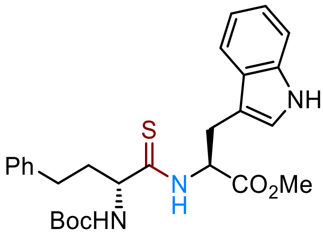

10l

Following procedure B: **10l** was isolated as yellow oil in 58% yield (28.7 mg; 0.058 mmol) which was purified by silica gel chromatography (PE:EA = 20:1~5:1, R_f = 0.35 (PE:EA = 5:1)).

$[\alpha]_{25\ D}$= 39.2 (*c* = 1.60, $CHCl_3$).

^{1}H NMR (400 MHz, $CDCl_3$) δ 8.39 (s, 1H), 8.08 (s, 1H), 7.31 (d, *J* = 8.0 Hz, 1H), 7.27 – 7.21 (m, 3H), 7.21 – 7.14 (m, 2H), 7.10 – 7.03 (m, 3H), 6.97 (d, *J* = 2.5 Hz, 1H), 5.44 – 5.36 (m, 1H), 5.21 (d, *J* = 8.2 Hz, 1H), 4.36 (s, 1H), 3.67 (s, 3H), 3.53 (d, *J* = 13.2 Hz, 1H), 3.41 (dd, *J* = 14.8, 5.8 Hz, 1H), 2.66 – 2.45 (m, 2H), 2.13-2.19 (m, 1H), 1.96 (s, 1H), 1.40 (s, 9H).

^{13}C NMR (101 MHz, $CDCl_3$) δ 204.4, 171.4, 155.6, 140.9, 136.2, 128.6, 128.5, 127.4, 126.2, 123.1, 122.5, 119.9, 118.5, 111.4, 109.4, 80.4, 60.5, 58.1, 52.7, 37.2, 31.9, 28.4, 26.4.

HRMS (ESI) m/z: $[M+Na]^+$ calcd. for $C_{27}H_{33}N_3O_4SNa$ 518.2084; found, 518.2078.

FT-IR (neat): 3325, 2976, 1731, 1692, 1502, 1454, 1366, 1164, 700 cm^{-1}.

1-(*tert*-Butyl) 4-methyl ((*R*)-2-((*tert*-butoxycarbonyl)amino)-4-phenylbutanethio-yl)-*L*-aspartate (10m)

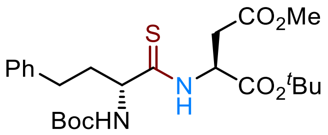

10m

Following procedure B: **10m** was isolated as yellow oil in 73% yield (35.1 mg; 0.073 mmol) which was purified by silica gel chromatography (PE:EA = 20:1~5:1, R_f = 0.5 (PE:EA = 5:1)).

$[\alpha]_{25\ D}$= 8.2 (*c* = 1.00, $CHCl_3$).

^{1}H NMR (400 MHz, $CDCl_3$) δ 8.65 (d, *J* = 5.8 Hz, 1H), 7.33 – 7.26 (m, 2H), 7.21 – 7.15 (m, 3H), 5.30 – 5.15 (m, 2H), 4.43 (s, 1H), 3.64 (s, 3H), 3.08 (qd, *J* = 17.0, 4.3 Hz, 2H), 2.76 – 2.55 (m, 2H), 2.25 (ddt, *J* = 13.7, 9.9, 5.9 Hz, 1H), 2.12 – 1.95 (m, 1H), 1.47 (s, 9H), 1.44 (s, 9H).

^{13}C NMR (101 MHz, $CDCl_3$) δ 203.9, 171.2, 168.6, 155.2, 140.8, 128.6, 128.5, 126.3, 83.4, 80.4, 61.1, 54.1, 52.1, 37.4, 34.6, 32.0, 28.4, 28.0.

HRMS (ESI) m/z: $[M+Na]^+$ calcd. for $C_{24}H_{36}N_2O_6SNa$ 503.2186; found, 503.2185.

FT-IR (neat): 3305, 2978, 1739, 1700, 1557, 1497, 1436, 1367, 1160, 701 cm^{-1}.

Diethyl ((*R*)-2-((*tert*-butoxycarbonyl)amino)-4-phenylbutanethioyl)-*L*-glutamate (10n)

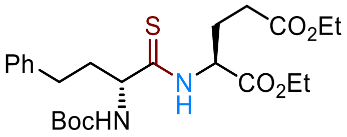

10n

Following procedure B: **10n** was isolated as light yellow oil in 64% yield (30.7 mg; 0.064 mmol) which was purified by silica gel chromatography (PE:EA = 20:1~5:1, R_f = 0.4 (PE:EA = 5:1)).

$[\alpha]_{25\ D}$= 25.6 (*c* = 2.40, $CHCl_3$).

^{1}H NMR (400 MHz, $CDCl_3$) δ 8.76 (s, 1H), 7.31 – 7.25 (m, 2H), 7.19 (td, *J* = 5.9, 1.8 Hz, 3H), 5.28 (d, *J* = 8.0 Hz, 1H), 5.10 (td, *J* = 7.1, 5.1 Hz, 1H), 4.44 (d, *J* = 6.0 Hz, 1H), 4.21 (qd, *J* = 7.1, 2.2 Hz, 2H), 4.11 (qd, *J* = 7.1, 1.4 Hz, 2H), 2.78 – 2.61 (m, 2H), 2.51 – 2.12 (m, 5H), 2.06 (h, *J* = 8.5 Hz, 1H), 1.44 (s, 9H), 1.26 (dt, *J* = 20.9, 7.1 Hz, 6H).

^{13}C NMR (101 MHz, $CDCl_3$) δ 204.9, 172.9, 170.6, 155.6, 140.9, 128.6, 128.5, 126.3, 80.4, 70.1, 62.0, 61.0, 57.0, 39.8, 37.0, 32.1, 30.2, 28.4, 26.1, 14.2.

HRMS (ESI) m/z: $[M+Na]^+$ calcd. for $C_{24}H_{36}N_2O_6SNa$ 503.2186; found, 503.2193.

FT-IR (neat): 3303, 2983, 1736, 1700, 1497, 1453, 1367, 1166, 701 cm^{-1}.

***tert*-Butyl ((*R*)-2-((*tert*-butoxycarbonyl)amino)-4-phenylbutanethioyl)-*L*-glutaminate (10o)**

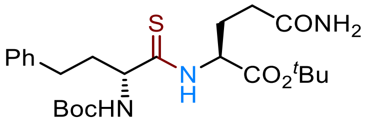

10o

Following procedure B: **10o** was isolated as light yellow oil in 88% yield (42.2 mg; 0.088 mmol) which was purified by silica gel chromatography (PE:EA = 10:1~1:1, R_f = 0.3 (PE:EA = 1:1)).

$[\alpha]_{25}$ $_{D}$= 7.6 (*c* = 1.20, $CHCl_3$).

^{1}H NMR (400 MHz, $CDCl_3$) δ 8.93 (s, 1H), 7.29 (d, *J* = 8.0 Hz, 2H), 7.23 – 7.16 (m, 3H), 5.87 (s, 1H), 5.50 (s, 1H), 5.18 (d, *J* = 7.1 Hz, 1H), 4.95 (q, *J* = 6.3 Hz, 1H), 4.44 (q, *J* = 7.6 Hz, 1H), 2.68 (dpd, *J* = 20.6, 13.9, 5.7 Hz, 2H), 2.39 – 2.14 (m, 5H), 2.04 (dq, *J* = 18.4, 7.2 Hz, 1H), 1.47 (s, 9H), 1.44 (s, 9H).

^{13}C NMR (101 MHz, $CDCl_3$) δ 198.5, 174.5, 169.7, 155.6, 140.8, 128.7, 128.5, 126.3, 83.1, 80.7, 57.6, 37.0, 32.2, 31.4, 29.8, 28.4, 28.1, 26.6.

HRMS (ESI) m/z: $[M+H]^+$ calcd. for $C_{24}H_{38}N_3O_5S$ 480.2527; found, 480.2525.

FT-IR (neat): 3331, 2977, 1724, 1704, 1682, 1669, 1655, 1558, 1520, 1506, 1456, 1393, 1367, 1161, 1046, 913, 697 cm^{-1}.

<u>*tert*-Butyl ((*R*)-2-((*tert*-butoxycarbonyl)amino)-4-phenylbutanethioyl)-*L*-serinate</u> (**10p**)

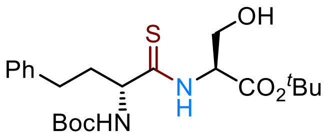

10p

Following procedure B: **10p** was isolated as yellow oil in 67% yield (29.3 mg; 0.067 mmol) which was purified by silica gel chromatography (PE:EA= 10:1~1:1, R_f= 0.4 (PE:EA = 1:1)).

$[\alpha]_{25}$ $_{D}$= 5.6 (*c* = 1.20, $CHCl_3$).

^{1}H NMR (400 MHz, $CDCl_3$) δ 8.77 (s, 1H), 7.30 – 7.25 (m, 2H), 7.19 (td, *J* = 6.0, 1.6 Hz, 3H), 5.28 (s, 1H), 5.06 – 4.98 (m, 1H), 4.51 – 4.39 (m, 1H), 4.24 (s, 1H), 3.97 (d, *J* = 10.2

Hz, 1H), 2.70 (tt, J = 13.7, 6.9 Hz, 3H), 2.31 (dddd, J = 14.3, 9.6, 6.6, 4.9 Hz, 1H), 2.06 (td, J = 14.6, 9.1 Hz, 1H), 1.49 (s, 9H), 1.43 (s, 9H).

^{13}C NMR (101 MHz, $CDCl_3$) δ 203.8, 168.8, 156.0, 140.7, 128.7, 128.5, 126.3, 83.4 (2C), 80.9, 61.5, 60.0, 36.9, 32.2, 28.3, 28.1.

HRMS (ESI) m/z: $[M+Na]^+$ calcd. for $C_{22}H_{34}N_2O_5SNa$ 461.2081; found, 461.2085.

FT-IR (neat): 3304, 2978, 1727, 1697, 1498, 1454, 1368, 1159, 699 cm^{-1}.

tert-Butyl ((_R_)-2-((_tert_-butoxycarbonyl)amino)-4-phenylbutanethioyl)-_L_-histidin-ate (10q)

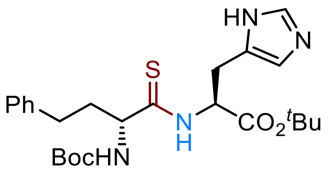

10q

Following procedure B: **10q** was isolated as yellow oil in 43% yield (21.0 mg; 0.043 mmol) which was purified by silica gel chromatography (PE:EA = 20:1~5:1, R_f = 0.2 (PE:EA = 5:1)).

$[\alpha]_{25\ D}$= 28.4 (c = 1.00, $CHCl_3$).

^{1}H NMR (400 MHz, $CDCl_3$) δ 9.50 (s, 1H), 7.45 (s, 1H), 7.30 – 7.27 (m, 1H), 7.26 (d, J = 1.8 Hz, 1H), 7.21 – 7.16 (m, 3H), 6.79 (s, 1H), 5.37 (d, J = 7.7 Hz, 1H), 5.17 – 5.05 (m, 1H), 4.46 (td, J = 8.2, 4.9 Hz, 1H), 3.36 (d, J = 11.3 Hz, 1H), 3.17 (dd, J = 15.2, 5.4 Hz, 1H), 2.68 (dddd, J = 23.6, 13.7, 9.8, 5.8 Hz, 2H), 2.42 – 2.28 (m, 1H), 2.08 (s, 1H), 1.43 (s, 9H), 1.40 (s, 9H).

^{13}C NMR (101 MHz, $CDCl_3$) δ 203.8, 175.2, 169.1, 155.9, 140.9, 135.3, 128.6, 128.5, 126.2, 118.4, 82.8, 80.6, 61.7, 58.3, 37.3, 32.1, 28.4, 28.0, 21.8.

HRMS (ESI) m/z: $[M+Na]^+$ calcd. for $C_{25}H_{36}N_4O_4SNa$ 511.2350; found, 511.2348.

FT-IR (neat): 3218, 2977, 1720, 1705, 1496, 1454, 1467, 1367, 1157, 699 cm^{-1}.

tert-Butyl _N^6_-((benzyloxy)carbonyl)-_N^2_-((_R_)-2-((_tert_-butoxycarbonyl)amino)-4-phenylbutanethioyl)-_L_-lysinate (10r)

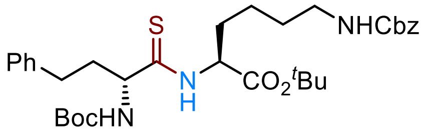

10r

Following procedure B: **10r** was isolated as yellow oil in 68% yield (41.7 mg; 0.068 mmol) which was purified by silica gel chromatography (PE:EA = 20:1~5:1, R_f= 0.45 (PE:EA = 5:1)).

$[\alpha]_{25\ D}$= 9.0 (*c* = 1.50, $CHCl_3$).

^{1}H NMR (400 MHz, $CDCl_3$) δ 8.56 (s, 1H), 7.37 – 7.23 (m, 7H), 7.21 – 7.13 (m, 3H), 5.25 (t, *J* = 6.9 Hz, 1H), 5.08 (s, 2H), 4.94 (q, *J* = 5.9 Hz, 2H), 4.42 (s, 1H), 3.15 (p, *J* = 6.9 Hz, 2H), 2.67 (dt, *J* = 9.3, 6.0 Hz, 2H), 2.38 – 2.21 (m, 1H), 2.11 – 1.93 (m, 2H), 1.90 – 1.76 (m, 1H), 1.57 – 1.49 (m, 2H), 1.46 (s, 9H), 1.42 (s, 9H), 1.40 – 1.21 (m, 3H).

^{13}C NMR (101 MHz, $CDCl_3$) δ 203.8, 170.4, 156.6, 155.5, 140.9, 136.7, 128.6, 128.6, 128.5, 128.2, 128.2, 126.3, 82.9, 80.5, 66.7, 61.0, 57.7, 40.5, 37.0, 32.2, 30.5, 29.6, 28.4, 28.1, 22.0.

HRMS (ESI) m/z: $[M+Na]^+$ calcd. for $C_{33}H_{47}N_3O_6SNa$ 636.3078; found, 636.3079.

FT-IR (neat): 3314, 2977, 1730, 1701, 1769, 1653, 1521, 1496, 1453, 1366, 1157, 699 cm^{-1}.

tert-Butyl N^2-((*R*)-2-((tert-butoxycarbonyl)amino)-4-phenylbutanethioyl)-N^4-(tert-butyl)-*L*-asparaginate (10s)

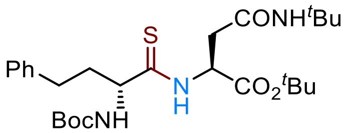

10s

Following procedure B: **10s** was isolated as yellow oil in 67% yield (34.9 mg; 0.067 mmol) which was purified by silica gel chromatography (PE:EA = 20:1~5:1, R_f = 0.4 (PE:EA = 5:1)).

$[\alpha]_{25\ D}$= 11.4 (*c* = 0.90, $CHCl_3$).

^{1}H NMR (400 MHz, $CDCl_3$) δ 9.05 (s, 1H), 7.29 – 7.23 (m, 2H), 7.20 – 7.14 (m, 3H), 5.42 (s, 1H), 5.33 (d, *J* = 6.3 Hz, 1H), 5.23 (dt, *J* = 8.0, 4.1 Hz, 1H), 4.49 (s, 1H), 2.86 – 2.75

(m, 2H), 2.74 – 2.56 (m, 2H), 2.29 – 2.16 (m, 1H), 2.06 – 1.96 (m, 1H), 1.46 (s, 9H), 1.43 (s, 9H), 1.28 (s, 9H).

^{13}C NMR (101 MHz, $CDCl_3$) δ 203.6, 169.1, 168.9, 155.1, 141.0, 128.5, 128.5, 126.2, 82.9, 80.1, 61.2, 55.0, 51.7, 38.0, 36.9, 32.1, 28.7, 28.4, 28.0.

HRMS (ESI) m/z: $[M+Na]^+$ calcd. for $C_{27}H_{43}N_3O_5SNa$ 544.2816; found, 544.2826.

FT-IR (neat): 3339, 2976, 1730, 1703, 1662, 1511, 1454, 1366, 1161, 699 cm^{-1}.

Methyl ((*R*)-2-((*tert*-butoxycarbonyl)amino)-4-phenylbutanethioyl)-*L*-phenylalaninate (10t)

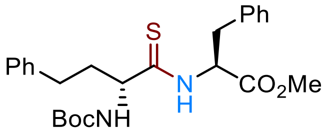

10t

Following procedure B: **10t** was isolated as yellow oil in 73% yield (33.3 mg; 0.073 mmol) which was purified by silica gel chromatography (PE:EA = 20:1~5:1, R_f = 0.55 (PE:EA = 5:1)).

$[\alpha]_{25}\ _D$= 18.6 (*c* = 0.90, $CHCl_3$).

^{1}H NMR (400 MHz, $CDCl_3$) δ 8.28 (s, 1H), 7.32 – 7.16 (m, 7H), 7.15 – 7.06 (m, 4H), 5.37 (q, *J* = 5.9 Hz, 1H), 5.17 (d, *J* = 6.5 Hz, 1H), 4.43 – 4.31 (m, 1H), 3.73 (s, 3H), 3.36 (dd, *J* = 13.8, 5.1 Hz, 1H), 3.20 (dd, *J* = 14.0, 5.7 Hz, 1H), 2.68 – 2.52 (m, 2H), 2.27 – 2.15 (m, 1H), 1.98 (dt, *J* = 14.0, 7.1 Hz, 1H), 1.43 (s, 9H).

^{13}C NMR (101 MHz, $CDCl_3$) δ 204.6, 171.0, 155.6, 140.8, 135.4, 129.3, 128.8, 128.6, 128.5, 127.4, 126.2, 60.6, 58.4, 52.6, 37.3, 36.5, 32.0, 29.8, 28.4.

HRMS (ESI) m/z: $[M+H]^+$ calcd. for $C_{25}H_{33}N_2O_4S$ 457.2156; found, 457.2186.

FT-IR (neat): 3295, 2976, 1740, 1701, 1603, 1496, 1454, 1437, 1366, 1169, 1047, 700 cm^{-1}.

***tert*-Butyl ((*R*)-2-((*tert*-butoxycarbonyl)amino)-4-phenylbutanethioyl)-*L*-prolinate (10u)**

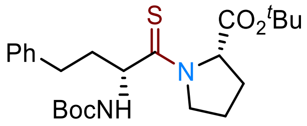

10u

Following procedure B: **10u** was isolated as yellow oil in 82% yield (36.8 mg; 0.082 mmol) which was purified by silica gel chromatography (PE:EA = 20:1~5:1, R_f= 0.45 (PE:EA = 5:1)).

$[\alpha]_{25}$ $_D$= -14.1 (*c* = 2.00, $CHCl_3$).

A mixture of two stereoisomers A and B in approximately 5.6:1 ratio.

^1H NMR (400 MHz, $CDCl_3$) δ **major rotamer:** 7.32 – 7.13 (m, 5H), 5.63 (dd, *J* = 22.2, 9.4 Hz, 1H), 4.83 – 4.61 (m, 1H), 3.96 – 3.65 (m, 1H), 3.48 – 3.16 (m, 1H), 2.84 – 2.53 (m, 2H), 2.36 – 1.86 (m, 6H), 1.46 (d, *J* = 3.8 Hz, 9H), 1.43 (d, *J* = 1.9 Hz, 9H).

^{13}C NMR (101 MHz, $CDCl_3$) **major rotamer:** δ 202.6, 169.1, 155.4, 141.3, 128.7, 128.6, 126.2, 81.9, 79.7, 66.4, 55.8, 50.6, 38.5, 32.0, 29.0, 28.5, 28.0, 24.4.

HRMS (ESI) m/z: $[M+Na]^+$ calcd. for $C_{24}H_{36}N_2O_4SNa$ 471.2288; found, 471.2283.

FT-IR (neat): 3000, 2979, 1737, 1711, 1498, 1469, 1452, 1366, 1153, 704 cm^{-1}.

<u>*tert*-Butyl ((*R*)-2-((tert-butoxycarbonyl)amino)-3-methylbutanethioyl)-*L*-tyrosin-ate</u> (10v)

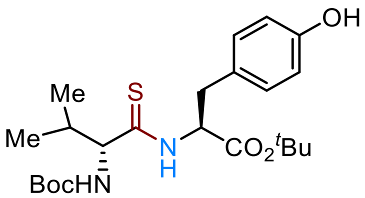

10v

Following procedure B: **10v** was isolated as yellow oil in 59% yield (26.7 mg; 0.059 mmol) which was purified by silica gel chromatography (PE:EA = 20:1~5:1, R_f = 0.5 (PE:EA = 5:1)).

$[\alpha]_{25}$ $_D$= 8.1 (*c* = 1.60, $CHCl_3$).

^{1}H NMR (400 MHz, $CDCl_3$) δ 8.21 (s, 1H), 7.03 – 6.98 (m, 2H), 6.75 – 6.69 (m, 2H), 5.21 (dt, J = 13.2, 7.7 Hz, 2H), 4.20 – 4.13 (m, 1H), 3.20 – 3.12 (m, 2H), 2.39 – 2.28 (m, 1H), 1.43 (s, 9H), 1.41 (s, 9H), 0.86 (t, J = 7.5 Hz, 6H).

^{13}C NMR (101 MHz, $CDCl_3$) δ 203.6, 169.9, 155.9, 155.2, 130.6, 127.4, 115.6, 83.1, 80.4, 66.5, 59.1, 35.8, 33.2, 28.4, 28.0, 19.8, 17.2.

HRMS (ESI) m/z: $[M+Na]^+$ calcd. for $C_{23}H_{36}N_2O_5SNa$ 475.2237; found, 475.2240.

FT-IR (neat): 3340, 2980, 1729, 1692, 1515, 1450, 1367, 1156, 843 cm^{-1}.

tert-Butyl ((_R_)-2-((tert-butoxycarbonyl)amino)-2-cyclohexylethanethioyl)-_L_-tyrosinate (10w)

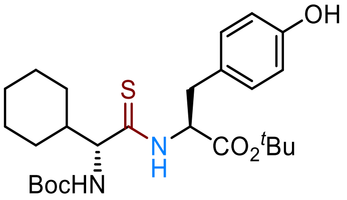

10w

Following procedure B: **10w** was isolated as yellow oil in 78% yield (38.4 mg; 0.078 mmol) which was purified by silica gel chromatography (PE:EA = 20:1~5:1, R_f = 0.5 (PE:EA = 5:1)).

$[\alpha]_{25}$ $_D$= 17.4 (*c* = 1.00, $CHCl_3$).

^{1}H NMR (400 MHz, $CDCl_3$) δ 8.13 (s, 1H), 7.04 – 6.99 (m, 2H), 6.74 (d, J = 2.0 Hz, 1H), 6.73 (d, J = 2.0 Hz, 1H), 5.33 – 5.14 (m, 2H), 4.21 – 4.08 (m, 1H), 3.17 (d, J = 5.8 Hz, 2H), 1.76 – 1.51 (m, 5H), 1.43 (s, 9H), 1.41 (s, 9H), 1.27 – 1.05 (m, 4H), 0.88 (ddt, J = 15.0, 11.6, 4.7 Hz, 2H).

^{13}C NMR (101 MHz, $CDCl_3$) δ 203.7, 169.8, 155.8, 155.1, 130.6, 127.5, 115.6, 83.1, 80.3, 65.9, 59.1, 42.7, 35.8, 30.1, 28.4, 28.0, 27.8, 26.2, 26.0, 26.0.

HRMS (ESI) m/z: $[M+Na]^+$ calcd. for $C_{26}H_{40}N_2O_5SNa$ 515.2550; found, 515.2552.

FT-IR (neat): 3342, 2979, 1724, 1701, 1686, 1513, 1458, 1367, 1156, 844 cm^{-1}.

tert-Butyl ((_R_)-2-((tert-butoxycarbonyl)amino)-4-methylpentanethioyl)-_L_-tyrosinate (10x)

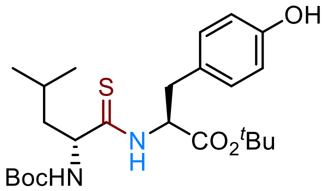

10x

Following procedure B: **10x** was isolated as yellow oil in 84% yield (39.2 mg; 0.084 mmol) which was purified by silica gel chromatography (PE:EA = 20:1~5:1, R_f = 0.5 (PE:EA = 5:1)).

$[\alpha]_{25}^{D}$= 11.4 (*c* = 1.00, $CHCl_3$).

^{1}H NMR (400 MHz, $CDCl_3$) δ 8.30 (s, 1H), 7.00 – 6.95 (m, 2H), 6.73 – 6.68 (m, 2H), 6.13 (s, 1H), 5.21 – 5.04 (m, 2H), 4.40 (td, *J* = 8.8, 5.1 Hz, 1H), 3.76 (ddt, *J* = 5.8, 4.1, 2.2 Hz, 1H), 3.16 (td, *J* = 14.6, 4.7 Hz, 2H), 1.89 – 1.83 (m, 1H), 1.42 (d, *J* = 2.5 Hz, 18H), 0.90 (dd, *J* = 8.5, 6.3 Hz, 6H).

^{13}C NMR (101 MHz, $CDCl_3$) δ 204.9, 170.0 (2C), 155.2, 130.6, 127.3, 115.6, 83.2, 68.1, 58.9, 45.0, 35.5, 28.4, 28.1, 25.7, 25.0, 23.0.

HRMS (ESI) m/z: $[M-H]^-$ calcd. for $C_{24}H_{37}N_2O_5S$ 465.2429; found, 465.2480.

FT-IR (neat): 3373, 3322, 2976, 1727, 1690, 1603, 1551, 1495, 1478, 1454, 1392, 1367, 1153, 1078, 1030, 993, 913, 847, 700 cm^{-1}.

tert-Butyl ((R)-2-((tert-butoxycarbonyl)amino)-3-phenylpropanethioyl)-L-leucinate (**10y**)

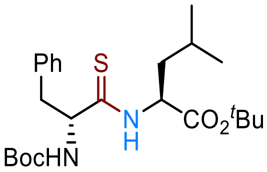

10y

Following procedure B: **10y** was isolated as yellow oil in 72% yield (32.4 mg; 0.072 mmol) which was purified by silica gel chromatography (PE:EA = 20:1~5:1, R_f = 0.5 (PE:EA = 5:1)).

$[\alpha]_{25}^{D}$= 6.8 (*c* = 1.20, $CHCl_3$).

^{1}H NMR (400 MHz, $CDCl_3$) δ 8.07 (s, 1H), 7.31 – 7.18 (m, 6H), 5.32 (s, 1H), 4.91 (dd, *J* = 7.8, 4.0 Hz, 1H), 4.60 (q, *J* = 6.9 Hz, 1H), 3.18 (d, *J* = 7.1 Hz, 2H), 1.91 – 1.78 (m, 1H), 1.73 (d, *J* = 3.3 Hz, 1H), 1.45 (s, 9H), 1.41 (s, 9H), 0.92 (t, *J* = 7.4 Hz, 3H), 0.58 (d, *J* = 6.9 Hz, 3H).

^{13}C NMR (101 MHz, $CDCl_3$) δ 202.4, 169.5, 155.0, 136.6, 129.3, 128.8, 127.1, 82.8, 80.4, 61.7, 41.8, 37.3, 28.3, 28.2, 26.3, 14.9, 11.8.

HRMS (ESI) m/z: $[M+Na]^+$ calcd. for $C_{24}H_{38}N_2O_4SNa$ 473.2445; found, 473.2449.

FT-IR (neat): 3408, 3292, 2974, 1705, 1604, 1496, 1455, 1435, 1392, 1367, 1160, 1078, 1047, 1024, 844, 700 cm^{-1}.

Methyl ((*R*)-2-((tert-butoxycarbonyl)amino)-4-phenylbutanethioyl)-*L*-phenylalanyl-*L*-leucinate (11a)

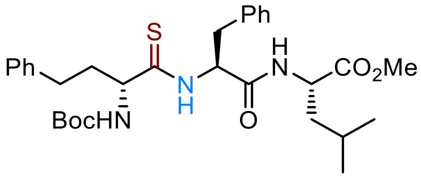

11a

Following procedure B: **11a** was isolated as yellow oil in 54% yield (30.7 mg; 0.054 mmol) which was purified by silica gel chromatography (PE:EA = 20:1~5:1, R_f= 0.4 (PE:EA = 5:1)).

$[\alpha]_{25}$ $_D$= -35.6 (*c* = 1.40, $CHCl_3$).

^{1}H NMR (400 MHz, $CDCl_3$) δ 8.36 (s, 1H), 7.28 (t, *J* = 6.3 Hz, 6H), 7.20 (d, *J* = 7.1 Hz, 3H), 7.10 (d, *J* = 7.7 Hz, 2H), 6.29 (s, 1H), 5.27 (dd, *J* = 20.9, 6.3 Hz, 2H), 4.59 – 4.45 (m, 1H), 4.26 (q, *J* = 7.8 Hz, 1H), 3.69 (s, 2H), 3.42 – 3.28 (m, 1H), 3.15 (s, 1H), 2.54 (d, *J* = 16.5 Hz, 2H), 2.09 (s, 1H), 2.02 – 1.87 (m, 1H), 1.42 (s, 9H), 1.25 (s, 3H), 0.86 (d, *J* = 4.4 Hz, 6H).

^{13}C NMR (101 MHz, $CDCl_3$) δ 204.2, 172.5, 169.3, 140.7, 136.0, 129.4, 128.9, 128.6, 128.4, 127.4, 126.3, 121.3, 80.4, 61.2, 59.3, 52.4, 51.2, 41.1, 37.2, 36.8, 31.9, 29.8, 28.4, 24.8, 22.8, 21.9.

HRMS (ESI) m/z: $[M+Na]^+$ calcd. for $C_{31}H_{43}N_3O_5SNa$ 592.2816; found, 592.2820.

FT-IR (neat): 3304, 2956, 1743, 1727, 1685, 1510, 1497, 1454, 1437, 1337, 1165, 699 cm^{-1}.

Supplementary Table S3. Optional solvents for polypeptide synthesis[a]

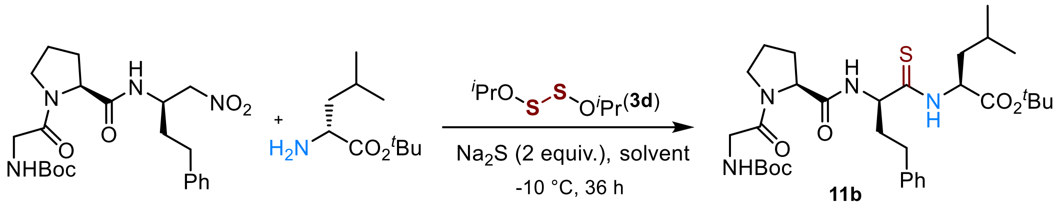

entry	solvent	Yield (%)[b]
1	THF	50
2	DMF	53
3	THF: DMF=1:1	51

[a]Unless noted otherwise, reactions were carried out with 0.1 mmol of **nitroalakne**, 0.2 mmol of **amine**, 0.2 mmol S source and 0.2 mmol of Na_2S in 1 mL of solvent. [b]Yield of isolated product.

For substrates less soluble in THF, it is thus possible to adopt DMF or DMF/THF mixtures in making polypeptides (**11b**-**11e**) (DMSO is also possible based on NMR studies).

tert-Butyl ((S)-2-((R)-1-((tert-butoxycarbonyl)glycyl)pyrrolidine-2-carboxamido)-4-phenylbutanethioyl)-L-leucinate (**11b**)

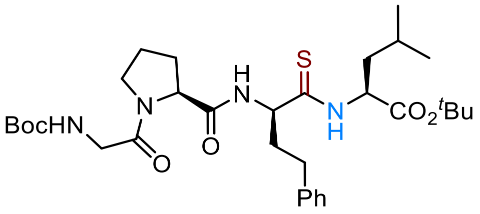

11b

Following procedure B: **11b** was isolated as yellow oil in 50% yield (30.9 mg; 0.078 mmol) which was purified by silica gel chromatography (DCM: MeOH = 50:1~10:1, R_f = 0.5 (DCM: MeOH = 10:1)).

$[\alpha]_{25\ D}$= -11.4 (*c* = 1.20, $CHCl_3$).

^{1}H NMR (400 MHz, $CDCl_3$) δ 8.39 (d, *J* = 7.7 Hz, 1H), 7.29 (d, *J* = 8.5 Hz, 2H), 7.20 (d, *J* = 7.7 Hz, 3H), 5.45 (s, 1H), 4.99 (q, *J* = 6.9 Hz, 1H), 4.70 (td, *J* = 8.4, 4.5 Hz, 1H), 4.45 (dd, *J* = 8.0, 3.3 Hz, 1H), 4.14 – 4.02 (m, 1H), 3.94 – 3.85 (m, 1H), 3.55 (td, *J* = 8.4, 3.7 Hz, 1H), 3.46 – 3.37 (m, 1H), 2.70 (t, *J* = 7.8 Hz, 2H), 2.47 (td, *J* = 13.6, 8.0 Hz, 1H), 2.32 (tt, *J* = 7.1, 3.4 Hz, 1H), 2.13 (dq, *J* = 14.8, 7.8 Hz, 2H), 2.02 – 1.89 (m, 2H), 1.80 – 1.57 (m, 3H), 1.47 (s, 9H), 1.44 (s, 9H), 0.92 (dd, *J* = 10.2, 6.3 Hz, 6H).

^{13}C NMR (101 MHz, $CDCl_3$) δ 202.6, 171.1, 169.1, 155.8, 141.0, 128.6, 126.2, 82.5, 79.9, 60.4, 59.5, 57.0, 46.4, 43.2, 40.3, 36.4, 32.2, 28.4, 28.1, 27.7, 25.0, 22.8, 22.5.

HRMS (ESI) m/z: $[M+Na]^+$ calcd. for $C_{32}H_{50}N_4O_6SNa$ 641.3343; found, 641.3366.

FT-IR (neat): 3347, 3289, 2976, 1739, 1718, 1653, 1512, 1456, 1367, 1157, 1135, 913, 702 cm^{-1}.

<u>*tert*-Butyl ((*R*)-2-((*S*)-2-((*S*)-2-(((benzyloxy)carbonyl)amino)-6-((tert-butoxy carbonyl) amino)hexanamido)propanamido)-4-phenylbutanethioyl)-*L*-leucinate</u> (11c)

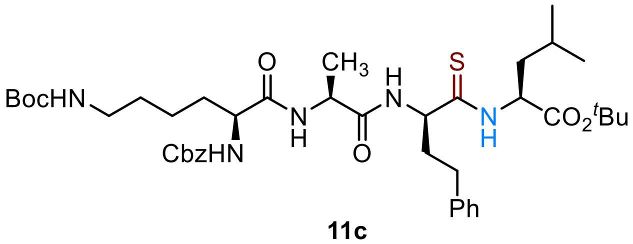

11c

Following procedure B: **11c** was isolated as yellow oil in 58% yield (46.3 mg; 0.058 mmol) which was purified by silica gel chromatography (DCM: MeOH = 50:1~10:1, R_f = 0.5 (DCM: MeOH = 10:1)).

$[\alpha]_{25\ D}$= -7.2 (*c* = 1.00, $CHCl_3$).

^{1}H NMR (400 MHz, $CDCl_3$) δ 7.32 (d, *J* = 3.0 Hz, 5H), 7.23 (d, *J* = 6.9 Hz, 2H), 7.16 (t, *J* = 6.9 Hz, 3H), 5.07 (s, 2H), 4.94 (q, *J* = 7.4 Hz, 2H), 4.56 (t, *J* = 7.0 Hz, 1H), 4.35 (s, 1H), 3.05 (dd, *J* = 12.0, 6.2 Hz, 2H), 2.76 – 2.50 (m, 2H), 2.28 – 1.97 (m, 2H), 1.89 – 1.57 (m, 5H), 1.44 (s, 9H), 1.42 – 1.23 (m, 16H), 0.95 (d, *J* = 6.0 Hz, 3H), 0.92 (d, *J* = 6.0 Hz, 3H).

^{13}C NMR (101 MHz, $CDCl_3$) δ 203.8, 172.0, 171.6, 170.6, 156.6, 141.1, 136.1, 128.6, 128.6, 128.5, 128.3, 128.2, 126.1, 82.3, 79.2, 77.3, 67.3, 58.0, 57.3, 54.9, 49.2, 40.4, 39.7, 37.7, 32.8, 31.9, 29.9, 28.5, 28.1, 25.2, 22.7, 22.4, 19.3.

HRMS (ESI) m/z: $[M+H]^+$ calcd. for $C_{42}H_{64}N_5O_8S$ 798.4470; found, 798.4498.

FT-IR (neat): 3282, 2976, 1733, 1699, 1684, 1637, 1519, 1454, 1392, 1366, 1155, 1135, 1050, 912, 844, 698 cm^{-1}.

N^6-((*S*)-2-((*R*)-2-((tert-butoxycarbonyl)amino)-3-phenylpropanamido)-4-phenyl butanethioyl)-N^2-palmitoylglycyl-*D*-histidyl-*L*-lysine (**11d**)

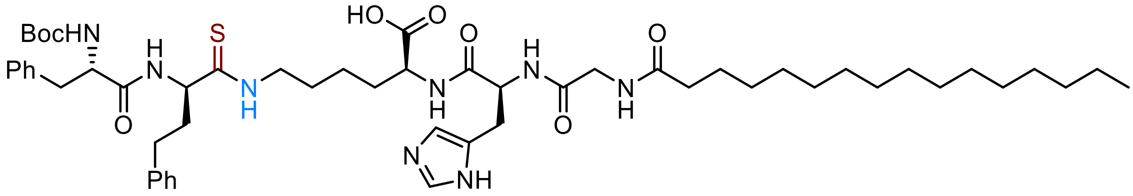

11d

Following procedure B: **11d** was isolated as yellow oil in 73% yield (36.6 mg; 0.0365 mmol) which was purified by silica gel chromatography (DCM:MeOH = 50:1~5:1, R_f = 0.3 (DCM:MeOH = 5:1)).

$[\alpha]_{25\ D}$= 29.4 (*c* = 1.00, $CHCl_3$).

^1H NMR (400 MHz, DMSO-d_6) δ 8.22 (s, 1H), 7.45 – 7.32 (m, 2H), 7.25 (q, *J* = 8.1 Hz, 7H), 7.21 – 7.02 (m, 5H), 6.64 (d, *J* = 25.8 Hz, 1H), 6.50 (s, 1H), 6.37 (s, 1H), 4.45 (s, 2H), 4.29 (d, *J* = 8.0 Hz, 1H), 4.25 – 4.01 (m, 2H), 3.87 (s, 1H), 3.69 (dd, *J* = 6.0, 2.5 Hz, 3H), 3.57 (s, 2H), 3.09 (td, *J* = 7.3, 4.3 Hz, 2H), 3.04 – 2.68 (m, 6H), 2.56 (d, *J* = 12.4 Hz, 1H), 2.35 (dd, *J* = 30.5, 9.6 Hz, 2H), 2.12 (t, *J* = 7.6 Hz, 3H), 1.99 (p, *J* = 6.9 Hz, 1H), 1.89 – 1.37 (m, 14H), 1.32 (s, 9H), 1.27 – 1.04 (m, 10H).

^{13}C NMR (101 MHz, DMSO-d_6) δ 202.5, 175.6, 173.1, 172.1, 169.6, 169.1, 163.3, 156.1, 149.7, 141.8, 138.4, 136.9, 130.2, 129.8, 128.8, 128.6, 127.0, 126.3, 78.8, 76.5, 76.5, 66.4, 61.5, 59.7, 57.0, 55.9, 54.8, 51.0, 50.1, 45.6, 42.7, 37.7, 35.5, 32.0, 31.8, 29.6, 29.6, 29.4, 29.3, 29.1, 28.7, 28.5, 27.3, 27.1, 25.7, 25.2, 22.6, 18.2, 14.5.

HRMS (ESI) m/z: $[M+NH_4]^+$ calcd. for $C_{54}H_{86}N_9O_8S$ 1020.6315; found, 1020.6313.

FT-IR (neat): 3277, 2977, 1709, 1674, 1668, 1586, 1555, 1496, 1454, 1417, 1369, 1153, 1087, 1045, 699 cm^{-1}.

Methyl ((*R*)-2-((*R*)-2-((tert-butoxycarbonyl)amino)-3-phenylpropanamido)-4-phenylbutanethioyl)-*L*-phenylalanyl-*L*-alanyl-*L*-tyrosylvalinate (11e)

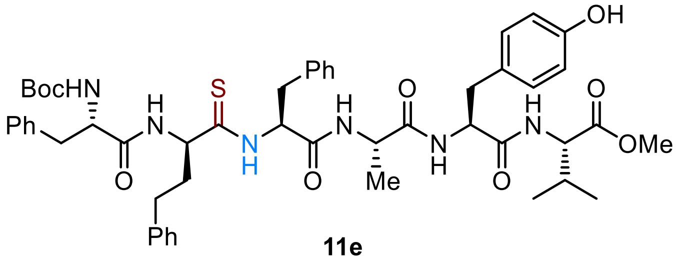

11e

Following procedure B: **11e** was isolated as yellow oil in 51% yield (47.7 mg; 0.051 mmol) which was purified by silica gel chromatography (DCM:MeOH = 50:1~10:1, R_f = 0.5 (DCM:MeOH = 10:1)).

$[\alpha]_{25\ D}$= 15.6 (*c* = 1.40, $CHCl_3$).

^{1}H NMR (400 MHz, MeOD-d_4) δ 7.34 – 7.04 (m, 15H), 7.01 (d, *J* = 8.1 Hz, 2H), 6.93 (d, *J* = 7.1 Hz, 2H), 6.67 (dd, *J* = 8.6, 4.9 Hz, 2H), 5.38 (dd, *J* = 10.9, 4.2 Hz, 1H), 4.65 – 4.48 (m, 1H), 4.47 – 4.08 (m, 4H), 3.71 – 3.57 (m, 3H), 3.39 (dd, *J* = 14.4, 4.1 Hz, 1H), 3.15 – 2.93 (m, 3H), 2.92 – 2.74 (m, 2H), 2.22 – 1.97 (m, 3H), 1.90 – 1.59 (m, 2H), 1.39 (d, *J* = 6.4 Hz, 2H), 1.36 (s, 9H), 1.31 – 1.19 (m, 1H), 0.92 (dt, *J* = 6.9, 5.4 Hz, 6H).

^{13}C NMR (101 MHz, MeOD-d_4) δ 206.0, 173.0, 172.5, 171.9, 171.7, 170.8, 155.9, 141.1, 137.3, 136.9, 130.1, 130.0, 129.0, 128.8, 128.2, 128.2, 128.1, 128.0, 127.3, 126.6, 126.4, 125.6, 114.9, 79.5, 60.0, 59.3, 57.9, 56.3, 54.9, 51.2, 49.5, 37.8, 36.7, 36.3, 31.1, 30.7, 27.4, 18.1, 17.4, 16.5.

HRMS (ESI) m/z: $[M+H]^+$ calcd. for $C_{51}H_{65}N_6O_9S$ 937.4528; found, 937.4526.

FT-IR (neat): 3281, 3064, 3028, 2969, 2931, 1728, 1633, 1516, 1496, 1451, 1436, 1367, 1168, 916, 699 cm^{-1}.

Di-*tert*-butyl ((propane-1,3-diylbis(azanediyl))bis(3-thioxopropane-3,1-diyl)) dicarbamate (13)

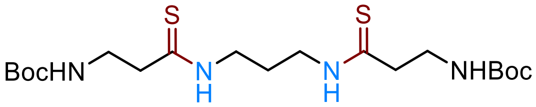

13

Following procedure A: the *bis*-thioamidated product **13** was isolated as yellow solid in 94% yield (1.265 g; 2.82 mmol) which was purified by silica gel chromatography (DCM: MeOH= 100:1~20:1, R_f = 0.5 (DCM: MeOH = 20:1)).

^{1}H NMR (400 MHz, $CDCl_3$) δ 9.02 (s, 1H), 5.36 (s, 1H), 3.75 (d, *J* = 5.7 Hz, 2H), 3.54 (q, *J* = 6.2 Hz, 2H), 2.87 (s, 2H), 1.98 (p, *J* = 6.1 Hz, 1H), 1.43 (s, 9H).

^{13}C NMR (101 MHz, $CDCl_3$) δ 202.6, 156.4, 79.8, 46.2, 42.6, 39.4, 28.5, 26.5.

HRMS (ESI) m/z: $[M+H]^+$ calcd. for $C_{19}H_{37}N_4O_4S_2$ 449.2251; found, 449.2253.

FT-IR (neat): 3267, 2979, 1688, 1521, 1458, 1410, 1365, 1170, 1094, 1046, 859, 706 cm^{-1}.

Di-*tert*-butyl (3,7,13,17-tetrathioxo-4,8,12,16-tetraazanonadecane-1,19-diyl)dicarbamate (14)

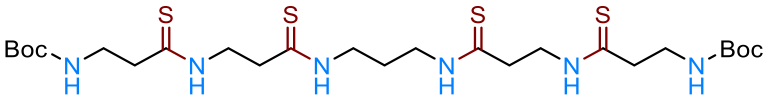

14

Following procedure A after *bis*-*N*-boc deptrotection of **13** with 4M HCl in 1,4-dioxane solution: the tetrakis-thioamide **14** was isolated as yellow solid in 91% yield (113.2 mg; 0.182 mmol) which was purified by silica gel chromatography (DCM: MeOH= 100:1~20:1, R_f= 0.45 (DCM: MeOH = 20:1)).

^{1}H NMR (400 MHz, $CDCl_3$) δ 9.24 (s, 1H), 9.00 (s, 1H), 5.39 (t, *J* = 6.0 Hz, 1H), 4.16 – 4.02 (m, 2H), 3.75 (s, 2H), 3.51 (s, 2H), 3.02 (s, 2H), 2.85 (s, 2H), 2.03 (s, 1H), 1.43 (s, 9H).

^{13}C NMR (101 MHz, $CDCl_3$) δ 202.3, 156.5, 79.9, 46.5, 44.7, 43.5, 42.9, 39.8, 28.5, 26.3.

HRMS (ESI) m/z: $[M+Na]^+$ calcd. for $C_{25}H_{46}N_6O_4S_4Na$ 645.2356; found, 645.2355.

FT-IR (neat): 3265, 2977, 1685, 1520, 1506, 1457, 1364, 1165, 1087, 1043, 867 cm^{-1}.

***N,N'*-(3,7,13,17-tetrathioxo-4,8,12,16-tetraazanonadecane-1,19-diyl)bis(4-methoxybenzothioamide) (16)**

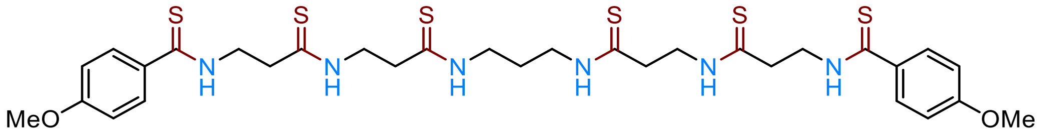

16

Following procedure A after *bis*-*N*-Boc deprotection of **14** with 4M HCl in 1,4-dioxane solution: the hexakis-thioamide **16** was isolated as yellow oil in 55% yield (79.4 mg; 0.11 mmol) which was purified by silica gel chromatography (DCM: MeOH= 100:1~20:1, R_f = 0.5(DCM: MeOH = 20:1)).

^{1}H NMR (400 MHz, $CDCl_3$) δ 9.02 (s, 1H), 8.74 (s, 1H), 8.43 (s, 1H), 7.84 (d, *J* = 8.8 Hz, 2H), 6.89 (d, *J* = 8.8 Hz, 2H), 4.16 – 4.09 (m, 2H), 4.07 – 3.99 (m, 2H), 3.84 (s, 3H), 3.57 (d, *J* = 5.4 Hz, 2H), 3.14 – 3.03 (m, 2H), 2.99 – 2.89 (m, 2H), 1.81 (dd, *J* = 16.1, 9.9 Hz, 2H).

^{13}C NMR (101 MHz, $CDCl_3$) δ 202.1, 202.0, 196.8, 162.6, 133.2, 129.0, 113.9, 55.7, 45.1, 44.4, 43.3, 42.7, 26.5.

HRMS (ESI) m/z: $[M+H]^+$ calcd. for $C_{31}H_{43}N_6O_2S_6$ 723.1766; found, 723.1768.

FT-IR (neat): 3205, 2935, 1604, 1527, 1502, 1439, 1403, 1326, 1169, 1087, 917, 713 cm^{-1}.

_N,N'_-(3,7,13,17-tetrathioxo-4,8,12,16-tetraazanonadecane-1,19-diyl)bis(4-hydroxybenzothioamide) (**Closthioamide**)

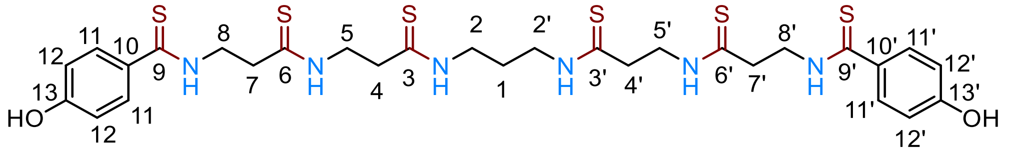

Closthioamide

Following reported BBr_3 demethylation of **16** with in DCM at -78 °C to 0 °C[20], **Closthioamide** was isolated as yellow oil in 62% yield (21.7 mg; 0.031 mmol) which was purified by silica gel chromatography (DCM: MeOH = 100:1~20:1, R_f = 0.5 (DCM: MeOH = 20:1)).

^1H NMR (400 MHz, CD_3OD) δ 9.73 (d, *J* = 30.2 Hz, 1H), 7.72 (d, *J* = 8.7 Hz, 2H), 6.76 (d, *J* = 8.7 Hz, 2H), 4.11 (t, *J* = 6.8 Hz, 2H), 4.01 (t, *J* = 6.7 Hz, 2H), 3.62 (t, *J* = 6.9 Hz, 2H), 3.03 (t, *J* = 6.8 Hz, 2H), 2.93 (t, *J* = 6.7 Hz, 2H), 1.95 (p, *J* = 6.9 Hz, 1H).

^{13}C NMR (101 MHz, CD_3OD) δ 203.3, 202.9, 199.1, 161.8, 134.0, 130.3, 115.7, 46.5, 45.8, 44.1, 44.1, 43.8, 27.1.

HRMS (ESI) m/z: $[M+H]^+$ calcd. for $C_{29}H_{39}N_6O_2S_6$ 695.1453; found, 695.1448.

FT-IR (neat): 3208, 2936, 1527, 1502, 1438, 1402, 1325, 1169, 1086, 916, 714 cm^{-1}.

Supplementary Table S4 : NMR data of naturally isolated Closthioamide and synthetic Closthioamide

	**Natural Closthioamide**[21]		**Synthetic Closthioamide**	
Position	δ_H (*J*[Hz], m)	δ_C	δ_H (*J*[Hz], m)	δ_C
**1**	1.94 (6.9, p)	27.1	1.95 (6.9, p)	27.1
**2,2’**	3.62 (6.9, t)	44.1	3.62 (6.9, t)	44.1
**3,3’**	-	202.9	-	202.9
**4,4’**	2.93 (6.8, t)	43.8	2.93 (6.7, t)	43.8
**5,5’**	4.01 (6.8, t)	45.8	4.01 (6.7, t)	45.8
**6,6’**	-	203.3	-	203.3
**7,7’**	3.02 (6.8, t)	44.1	3.03 (6.8, t)	44.1
**8,8’**	4.11 (6.8, t)	46.5	4.11 (6.8, t)	46.5
**9,9’**	-	199.1	-	199.1
**10,10’**	-	133.9	-	134.0
**11,11’**	7.71 (8.9, d)	130.3	7.72 (8.7, d)	130.3
**12,12’**	6.75 (8.9, d)	115.7	6.76 (8.7, d)	115.7
**13,13’**	-	161.8	-	161.8

2 Supplementary Discussion

2.1 Possible mechanistic pathways and control experiments

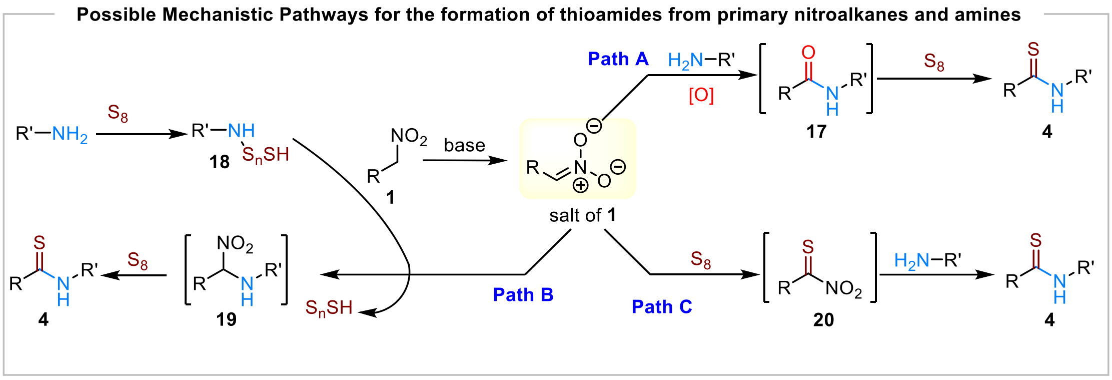

Supplementary Fig. S2 Three possible mechanistic pathways

2.1.1 Control reactions to interrogate path A.

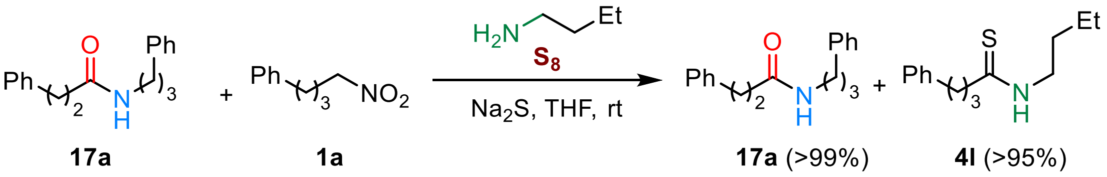

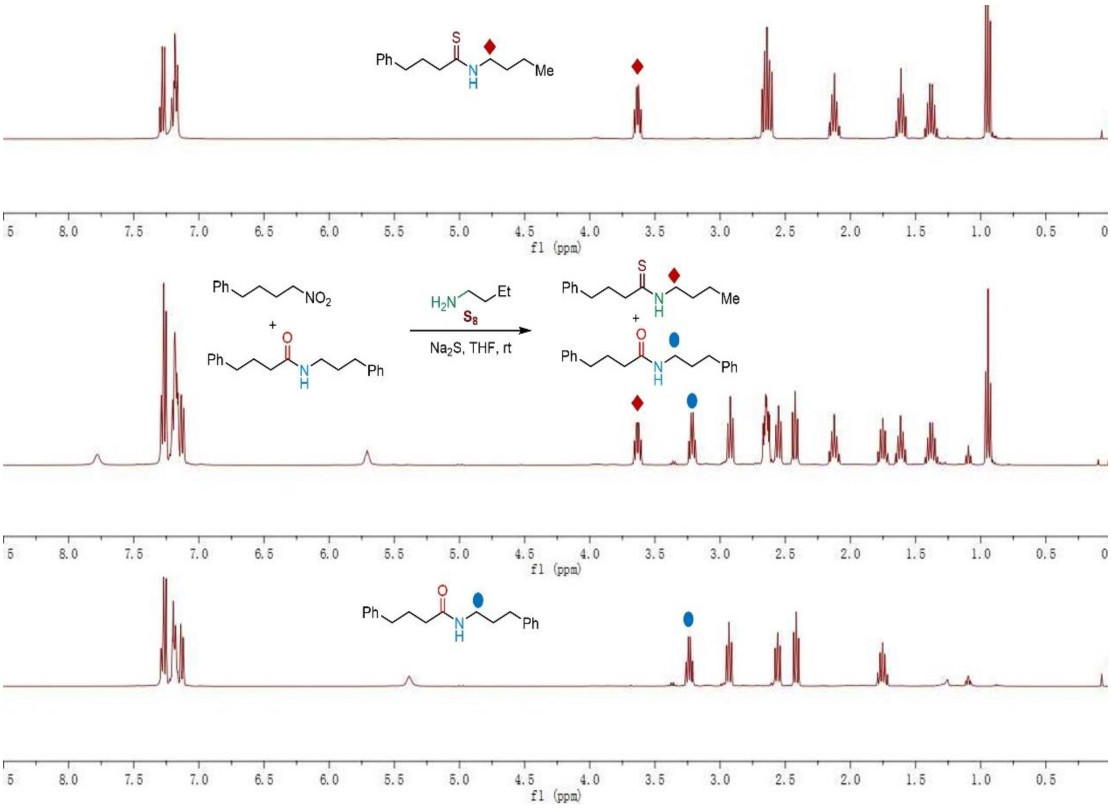

Supplementary Fig. S3 Comparison of the possibility of oxamide to thioamide.

We synthesize oxamide **17a** via reported procedure[22], then treated with our standard conditions, however, all the oxamide was completely recovered and no thioamide was observed at all (Eq. 1 in main manuscript).

<u>*N*-butyl-4-phenylbutanethioamide</u> (**4l**)

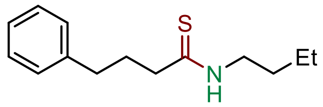

4l

Following procedure A: **4l** was isolated as yellow oil in 98% yield (46.1 mg; 0.196 mmol) which was purified by silica gel chromatography (PE:EA = 20:1~5:1, R_f = 0.5 (PE:EA = 5:1)).

^{1}H NMR (400 MHz, $CDCl_3$) δ 7.29 (d, *J* = 6.9 Hz, 2H), 7.22 – 7.15 (m, 3H), 3.71 – 3.57 (m, 2H), 2.64 (dt, *J* = 15.1, 7.7 Hz, 4H), 2.19 – 2.05 (m, 2H), 1.68 – 1.56 (m, 2H), 1.38 (h, *J* = 7.3 Hz, 2H), 0.95 (t, *J* = 7.4 Hz, 3H).

^{13}C NMR (101 MHz, $CDCl_3$) δ 205.0, 141.4, 128.6, 128.5, 126.1, 46.4, 46.0, 34.9, 30.8, 30.2, 20.3, 13.9.

HRMS (ESI) m/z: $[M+H]^+$ calcd. for $C_{14}H_{22}NS$ 236.1473; found, 236.1476.

FT-IR (neat): 3353, 2931, 2856, 1530, 1495, 1453, 1406, 1340, 1123, 1085, 1030, 909, 699 cm^{-1}.

Conclusion: Based on all control reactions in **Section 4.1**, thioamide formation via oxamide in *Figure S2* (**Path A**) was ruled out.

2.1.2 Control reactions to interrogate path B.

(1) Sulfenamide 18a was synthesize via reported procedure[23].

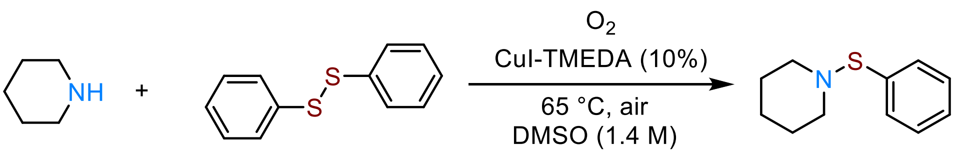

18a (88%)

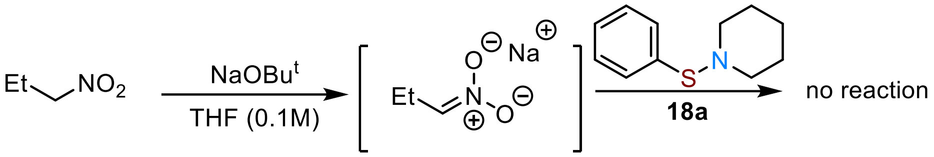

1b sodium salt of **1b**

(2) Synthesis of Diethyl 1-(nitro(phenyl)methyl)hydrazine-1,2-dicarboxylate (19a)

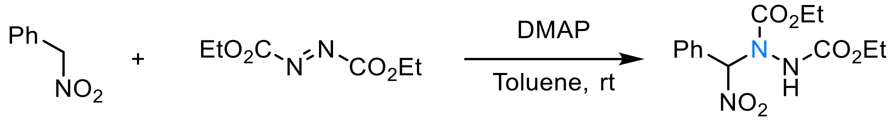

19a (45%)

19a was prepared from (nitromethyl)benzene in one step according to a reported procedure[24], which was isolated as colorless oil in 45% yield (140.0 mg; 0.45 mmol) after purification by silica gel chromatography (PE:EA = 20:1~5:1, R_f = 0.4 (PE:EA = 10:1)).

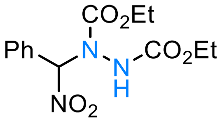

19a

^{1}H NMR (400 MHz, $CDCl_3$) δ 7.90 (s, 1H), 7.70 (s, 1H), 7.57 – 7.42 (m, 4H), 4.24 (q, *J* = 7.1 Hz, 4H), 4.01 (d, *J* = 7.1 Hz, 1H), 1.34 – 1.25 (m, 6H).

^{13}C NMR (101 MHz, $CDCl_3$) δ 155.9, 153.9, 130.7, 129.5, 128.3(2C), 63.2, 62.5, 14.6, 14.5, 13.9.

HRMS (ESI) m/z: $[M+NH_4]^+$ calcd. for $C_{13}H_{21}N_4O_6$ 318.1272; found, 318.1273.

FT-IR (neat): 3371, 2986, 1732, 1503, 1375, 1323, 1218, 1096, 1056, 1031, 702 cm^{-1}.

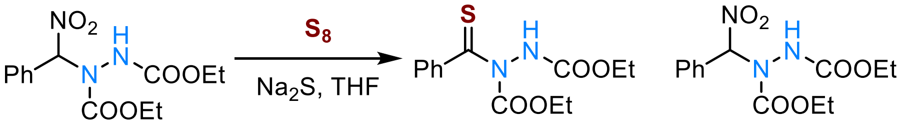

19a **21** (not observed) **19a** (>97%)

19a was treated with S_8 in our standard condition, we try to see it is any possible to generate thioamide, but only starting materials was generated.

Conclusion: Based on all control reaction in **Section 4.2**, thioamide formation via α-amino nitroalkane in *Figure S2* (**Path B**) was ruled out.

2.1.3 Control reactions to trap proposed thioacyl intermediate 20 (Path C).

(1) NMR and HRMS studies of **nitroalkane 22** was mixed with Na_2S and S_8 in **d^6-DMSO**

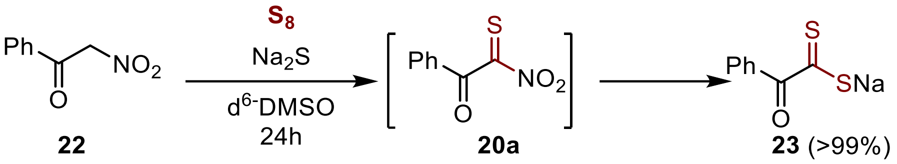

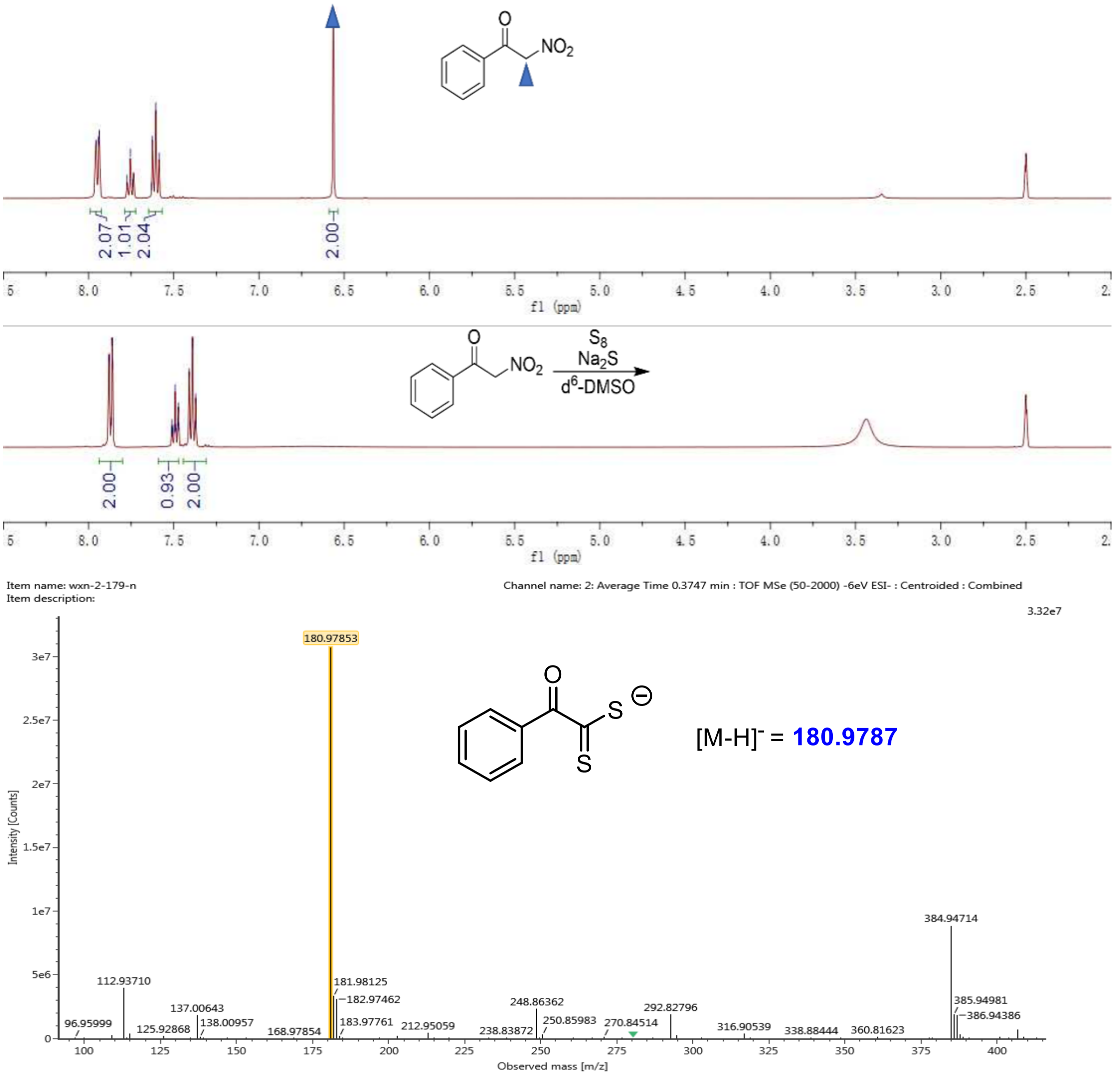

Supplementary Fig. S4 In-situ NMR studies of nitroalkane reacts with S_8 and Na_2S and HRMS study by negative ion mode

Characterization of **Sodium 2-oxo-2-phenylethanedithioate** (**23**)

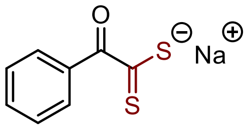

23

The yield determined by ^{1}H NMR, >99%.

^{1}H NMR (400 MHz, DMSO-d_6) δ 7.94 – 7.80 (m, 2H), 7.59 – 7.47 (m, 1H), 7.44 – 7.31 (m, 2H).

^{13}C NMR (101 MHz, DMSO-d_6) δ 254.6, 191.5, 134.8, 132.5, 130.2, 128.5.

HRMS (ESI) m/z: [M-H]$^-$ calcd. for $C_8H_4OS_2Na$ 180.9785; found, 180.9787.

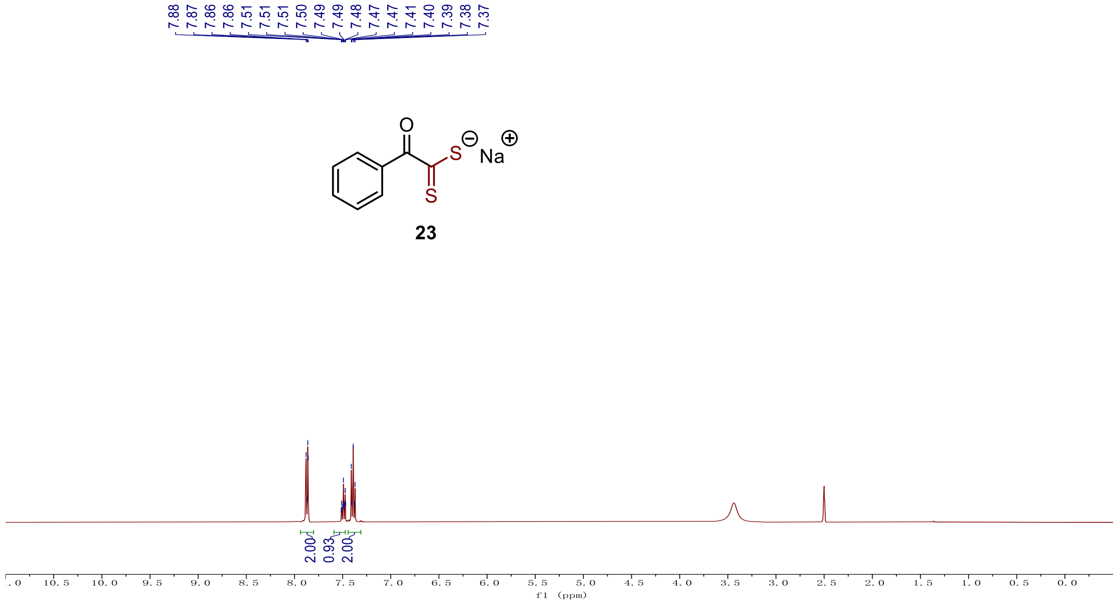

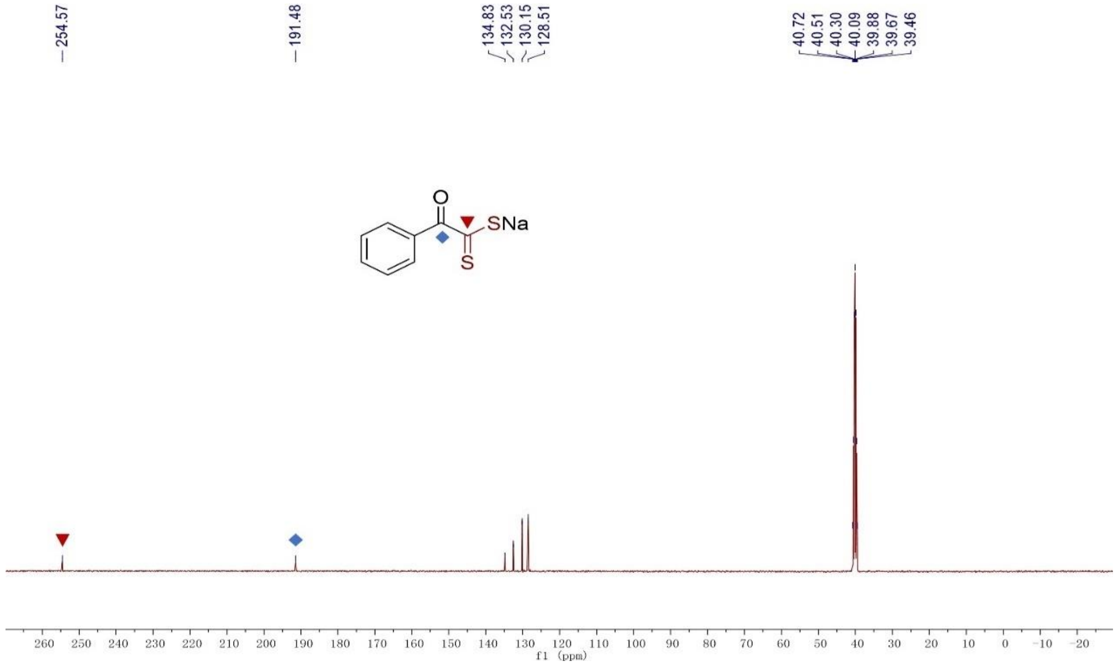

(2) Intramolecular trapping thioacyl nitrate with alcohol nucleaphiles

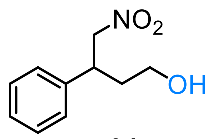

24

The **4-Nitro-3-phenylbutan-1-ol** (**24**) was prepared in 83% yield (5 mmol) from 3-Phenylpropanal in two steps according to a reported procedure[25].

a. Intramolecular experiment to trap acyl nitrate with alcohol:

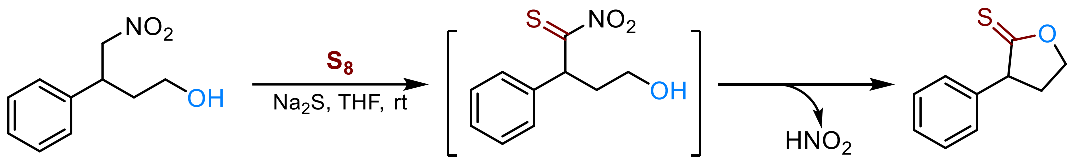

b. Intermolecular and intramolecular competing experiment to trap acyl nitrate with alcohol:

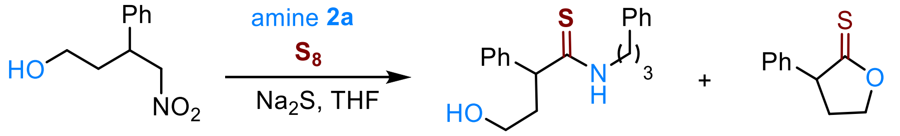

Supplementary Fig. S5 Control reaction to trap acyl nitrate

3-Phenyldihydrofuran-2(3*H*)-thione (**25**)

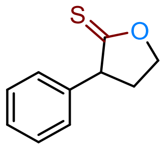

25

Following procedure A: **25** was isolated as yellow oil in 61% yield (21.7 mg; 0.122 mmol) which was purified by silica gel chromatography (PE:EA = 20:1~5:1, R_f = 0.5 (PE:EA = 7:1)).

^{1}H NMR (400 MHz, $CDCl_3$) δ 7.42 – 7.26 (m, 5H), 4.82 (ddd, *J* = 9.2, 8.2, 4.1 Hz, 1H), 4.68 (td, *J* = 9.1, 6.9 Hz, 1H), 4.14 (t, J = 9.0 Hz, 1H), 2.78 (dddd, *J* = 12.7, 8.5, 6.8, 4.0 Hz, 1H), 2.45 (dq, *J* = 12.8, 8.8 Hz, 1H).

^{13}C NMR (101 MHz, $CDCl_3$) δ 223.8, 139.3, 129.0, 128.4, 127.8, 75.0, 60.2, 33.5.

HRMS (ESI) m/z: $[M+Na]^+$ calcd. for $C_{10}H_{10}OSNa$ 201.0345; found, 201.0341.

FT-IR (neat): 2963, 1494, 1473, 1454, 1370, 1149, 699 cm^{-1}.

4-Hydroxy-2-phenyl-*N*-(3-phenylpropyl)butanethioamide (26)

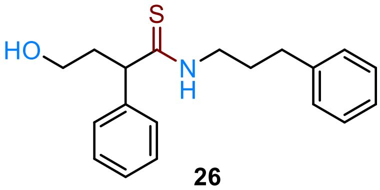

26

Following procedure A: **26** was isolated as yellow oil in 55% yield (17.2 mg; 0.055 mmol) which was purified by silica gel chromatography (PE:EA = 20:1~2:1, R_f = 0.5 (PE:EA = 1:1)).

^{1}H NMR (400 MHz, $CDCl_3$) δ 7.38 – 7.27 (m, 6H), 7.25 – 7.16 (m, 3H), 7.07 (d, *J* = 7.1 Hz, 2H), 4.07 (t, *J* = 7.6 Hz, 1H), 3.70 (ddd, *J* = 12.1, 7.1, 5.2 Hz, 1H), 3.66 – 3.57 (m, 3H), 2.58 – 2.51 (m, 3H), 2.15 (dtd, *J* = 14.0, 7.0, 5.1 Hz, 1H), 1.99 – 1.84 (m, 3H).

^{13}C NMR (101 MHz, $CDCl_3$) δ 206.0, 141.0, 139.8, 129.2, 128.7, 128.4, 128.1, 127.9, 126.3, 60.5, 57.0, 45.7, 37.8, 33.3, 29.4.

HRMS (ESI) m/z: $[M+H]^+$ calcd. for $C_{19}H_{24}NOS$ 314.1573; found, 314.1573.

FT-IR (neat): 3343, 3240, 2930, 2880, 1550, 1533, 1495, 1453, 1378, 1132, 1051, 1029, 913, 699 cm^{-1}.

c. Ion chromatography experiment to detect the formation of NO_2^-

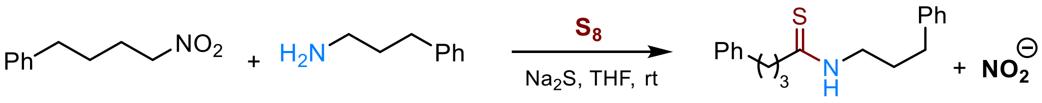

1a **2a** **4a** (98%) 91%

The nitro compound **1a** (0.2 mmol) and Na_2S (2.0 equiv.) were added to a 10 mL reaction tube, followed by THF (2 mL). After stirring for 10 minutes, S_8 (2.0 equiv.) was added. Next, the reaction was stirred at rt for another 10 minutes and the amine **2a** (2.0 equiv.) was added. The reaction was monitored by TLC until the nitroalkane was consumed. The mixture was then concentrated under reduced pressure and distilled water (5 mL) was added to the crude residue. Ultrasonication at 45 °C for one hour and centrifugation for 10 minutes then gave a clear liquid. This set of operations was repeated four times to give four water samples, which were combined, filtered and analyzed by ion chromatography in triplicate. In this way, the amount of $\mathbf{NO_2^-}$ via ion chromatographic analysis was performed 3 times to give an average concentration.

Theoretical concentration:

NO_2^- = 0.0133 mmol/mL = 13.3 mmol/L

Analysis results:

NO_2^- = (9.473 × 20 × 2.94÷ 46) mmol/L = 12.1 mmol/L

Average value	9.473
SD	0.216ppm
RSD	2.277%

Test conditions:

Test column	MetrosepA Supp 5 (250 mmH×4.0 mm IC)
Guard column type	Metrosep A Supp 5 Guard/ 4.0
Sample volume	200ul
Eluent	3.2 mmol/L Na_2CO_3 - 1.0 mmol/L $NaHCO_3$
Temperature	40 °C
Flow	0.700 mL/min
Suppressor	MSM-HC (Metrohm, regeneration fluid: 0.5% H_2SO_4)
Suppressor	MCS (Metrohm)
Detector	Conductivity detector (Metrohm)
Software	MagIC Net™

MagIC Net

2022-06-02 18:08:33

Sample data

Ident	-1-3 20
Sample type	
Determination start	2022-05-02 20:17:51 UTC+8
Method	20min
Operator	HPIC

Anions

1 (940 Professional IC Vario 1)

16.9 min

Metrosep A Supp 5 - 250/4.0

0.700 mL/min

12.09 MPa

40.0 °C

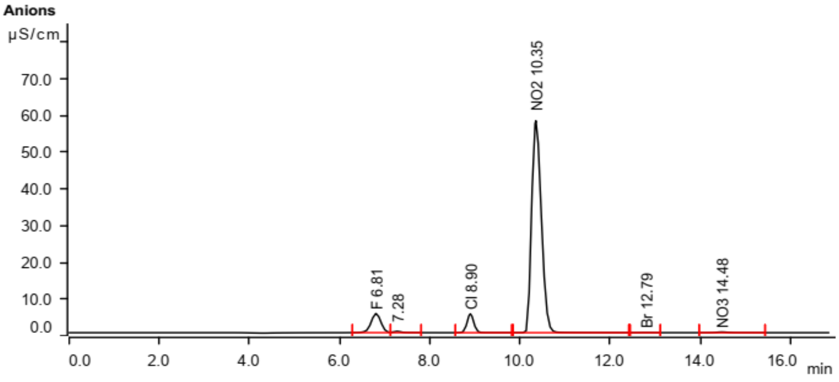

峰序列号	保留时间	面积	高度	浓度	组分名称	RSD Y
	min	(µS/cm)x min	µS/cm	ppm		
1	6.805	1.3234	5.214	-0.431	F	81.154
3	8.897	0.8909	5.142	0.893	Cl	87.975
4	10.350	14.3636	57.740	9.099	NO2	122.703
5	12.792	0.0072	0.028	0.158	Br	3.036
6	14.475	0.0556	0.192	0.648	NO3	80.932

Supplementary Fig. S6 Ion chromatographic analysis

Conclusion: Based on all control reaction in **Section 4.3**, thioamide formation via thioacyl nitrate as a key intermediate in *Figure S2* (**Path B**).

2.2 Control reaction to determine the thioacyl nitrate formation.

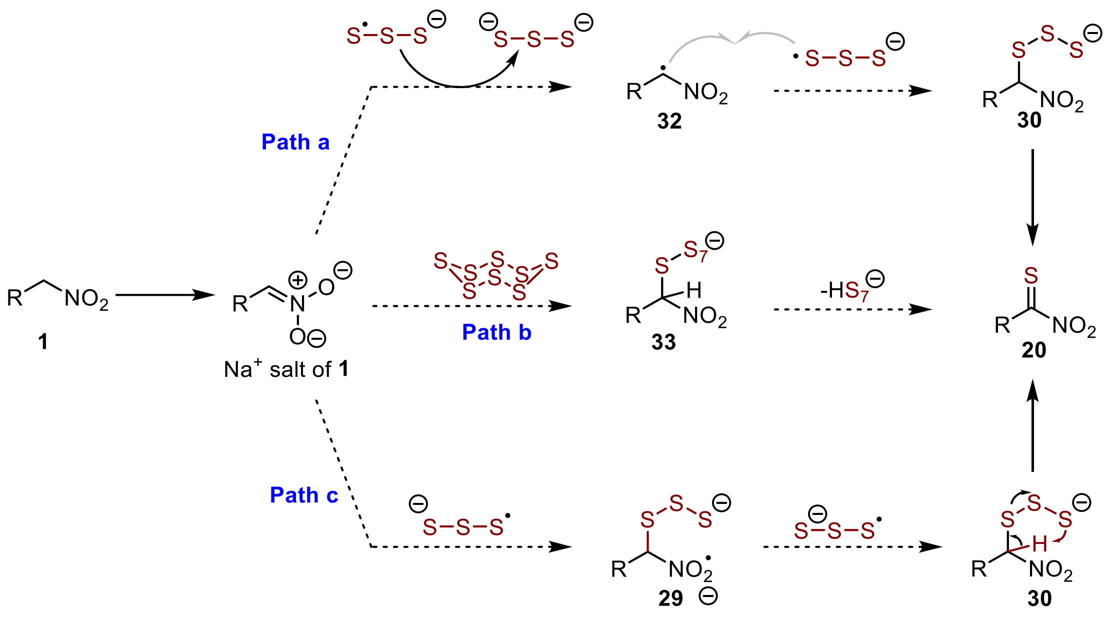

Supplementary Fig. S7 Three possible mechanistic pathways to thioamide bond formation

Based on relevant literature and results herein, there are three possible mechanistic pathways to form thioamides from nitroalkanes. These are proposed in ***Figure S7***. Below, we present evidence to distinguish the proposed mechanistic pathways.

2.2.1 Control reactions to rule out Path a and Path b

We designed a nitroalkanes bearing a *cis*-cycopropane; if a radical intermediate like **32** is formed, we would observe the *trans*-thioamide ***trans*-28** via radical ring opening and closing. Indeed, we only observed and isolated thioamide ***cis*-28**.

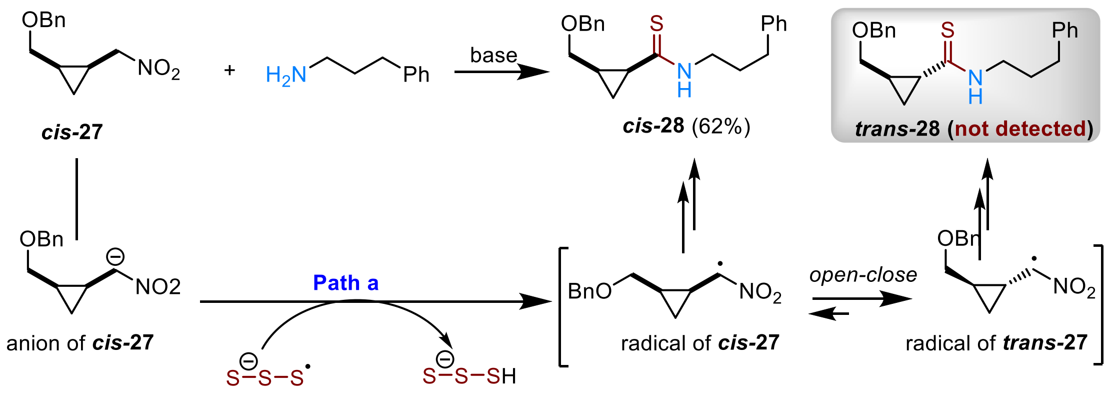

Supplementary Fig. S8 Radical clock reaction

Conclusion: Based on this reaction, **Path a** in Figure **S7/S8** was ruled out.

2.2.2 Control reaction to distinguish Path b and Path c

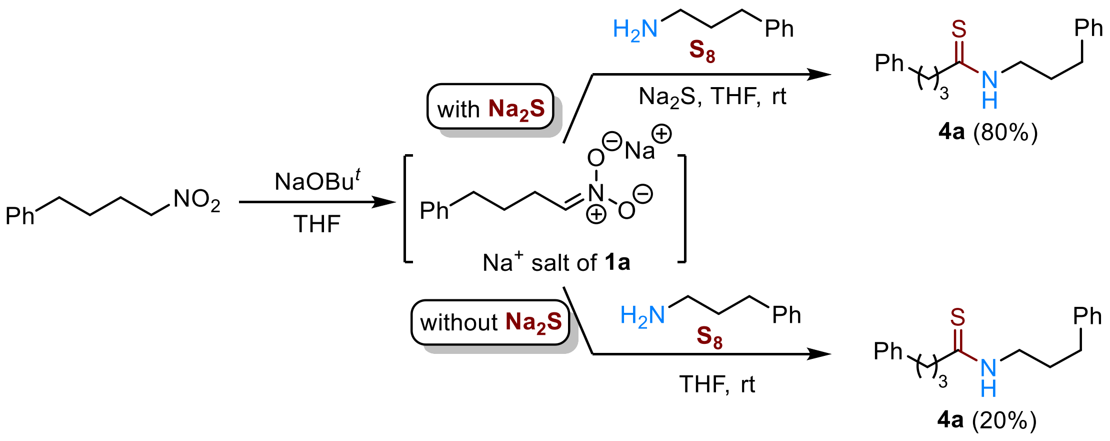

Supplementary Fig. S9 Na^+ salt of **1a** reacts with S_8 and S_3 radical anion

Conclusion: Based on above and ref. 36, 37 of paper**, Path b** (Fig. **S7**) was ruled out**.**

(((*(1R,2S)*-2-(Nitromethyl)cyclopropyl)methoxy)methyl)benzene (***cis*-27**)

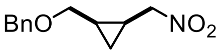

***cis*-27**

The nitro compound was prepared in 43% yield (2 mmol) through two steps according to a reported procedure[26].

^{1}H NMR (400 MHz, $CDCl_3$) δ 7.38 – 7.26 (m, 5H), 4.57 – 4.41 (m, 3H), 4.26 (dd, *J* = 13.8, 8.2 Hz, 1H), 3.74 (dd, *J* = 10.5, 5.4 Hz, 1H), 3.29 (dd, *J* = 10.5, 8.3 Hz, 1H), 1.62 (pd, *J* = 8.3, 5.6 Hz, 1H), 1.46 (qt, *J* = 8.5, 5.7 Hz, 1H), 0.99 (td, *J* = 8.4, 5.4 Hz, 1H), 0.50 (q, *J* = 5.6 Hz, 1H).

^{13}C NMR (101 MHz, $CDCl_3$) δ 138.0, 128.6(2C), 127.9, 76.1, 73.1, 69.2, 16.2, 13.3, 8.9.

***(1S,2R)*-2-((Benzyloxy)methyl)-*N*-(3-phenylpropyl)cyclopropane-1-carbothio-amide (28)**

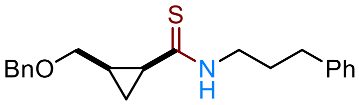

***cis*-28**

Following procedure B: In crude ^{1}H NMR, we only observed one isomer, which was isolated as yellow oil in 62% yield (42.1 mg; 0.124 mmol) by silica gel chromatography

(PE:EA = 20:1~5:1, R_f = 0.5 (PE:EA = 7:1)), and further conformed by 2D NMR as *cis*-isomer **28**.

^{1}H NMR (400 MHz, $CDCl_3$) δ 7.86 (s, 1H), 7.37 – 7.26 (m, 7H), 7.24 – 7.17 (m, 1H), 7.16 – 7.11 (m, 2H), 4.49 – 4.40 (m, 2H), 3.84 (dd, *J* = 10.3, 5.0 Hz, 1H), 3.74 – 3.56 (m, 2H), 3.54 – 3.47 (m, 1H), 2.64 (t, *J* = 7.6 Hz, 2H), 2.09 (td, *J* = 8.5, 6.0 Hz, 1H), 1.90 (tt, *J* = 14.7, 7.1 Hz, 2H), 1.55 (qdd, *J* = 8.8, 6.6, 5.0 Hz, 1H), 1.23 (dd, *J* = 11.8, 5.9 Hz, 2H), 1.15 (td, *J* = 8.5, 5.2 Hz, 1H).

^{13}C NMR (101 MHz, $CDCl_3$) δ 201.2, 141.2, 138.0, 128.6, 128.6, 128.4, 127.9, 126.2, 73.2, 69.2, 46.0, 33.4, 30.0, 29.5, 21.1, 12.1.

HRMS (ESI) m/z: $[M+H]^+$ calcd. for $C_{21}H_{26}NOS$ 340.1730; found, 340.1731.

FT-IR (neat): 3265, 2928, 2855, 1602, 1541, 1496, 1452, 1404, 1329, 1163, 1108, 1071, 912, 699 cm^{-1}.

2D NMR spectra was used to confirm the structure of **28** was *cis* isomer.

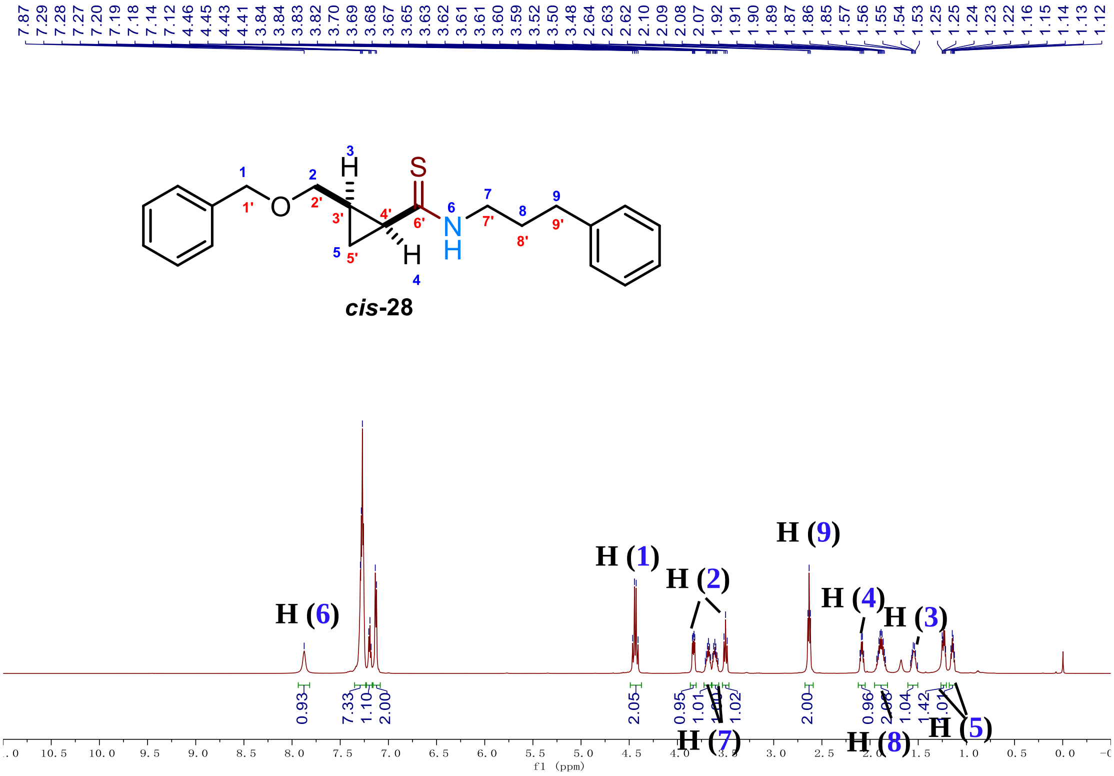

^{1}H NMR (400 MHz, Chloroform-*d*) (**28**):

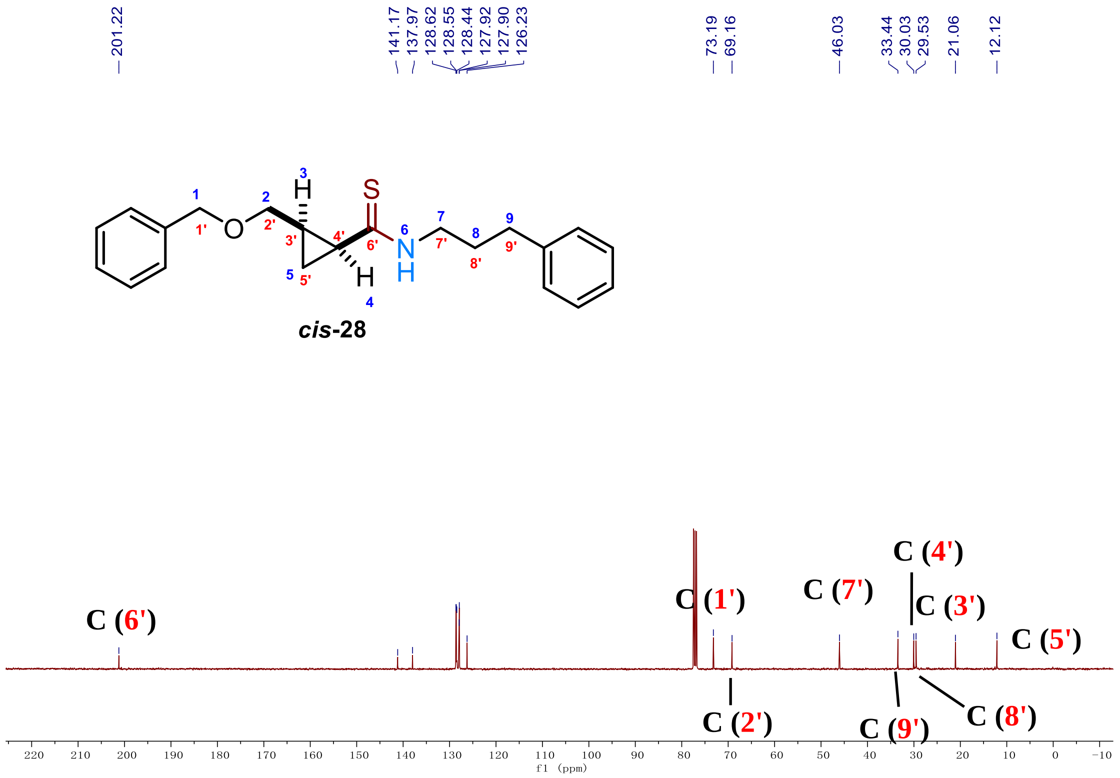

^{13}C NMR (101 MHz, Chloroform-*d*) (**28**):

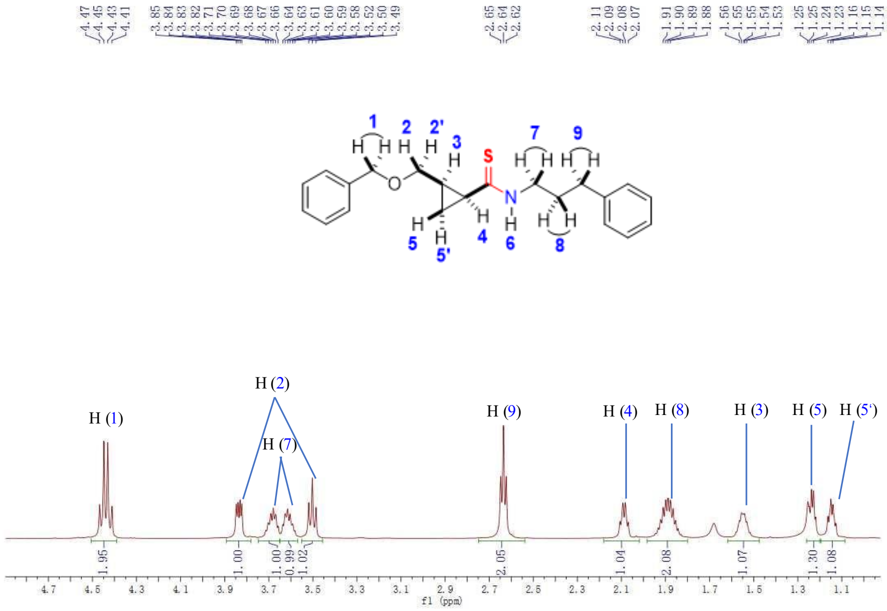

2D Noesy spectrum of 28:

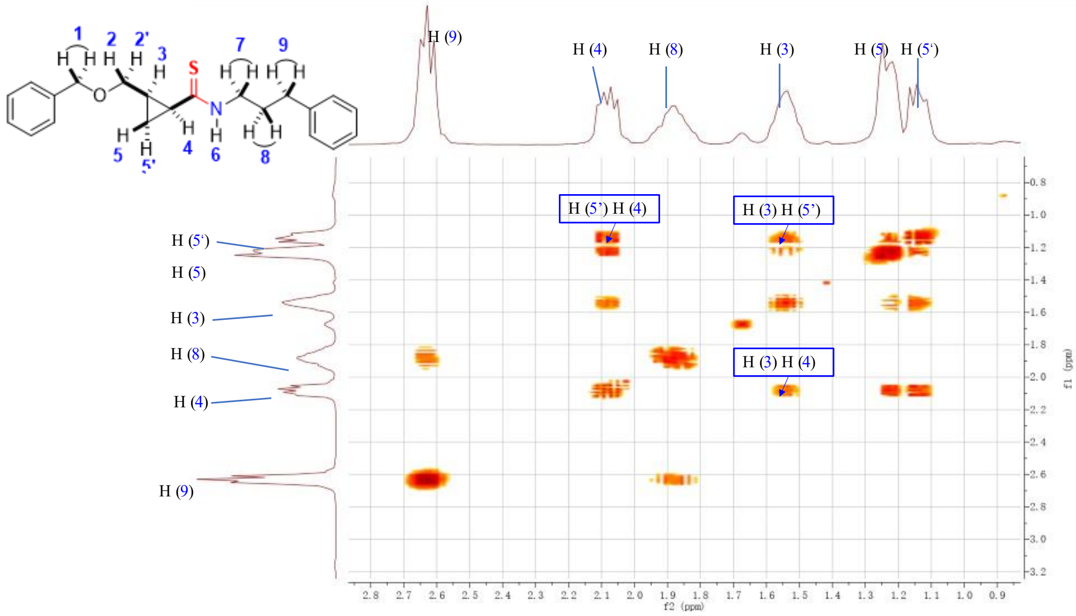

2D COSY spectrum of **28**:

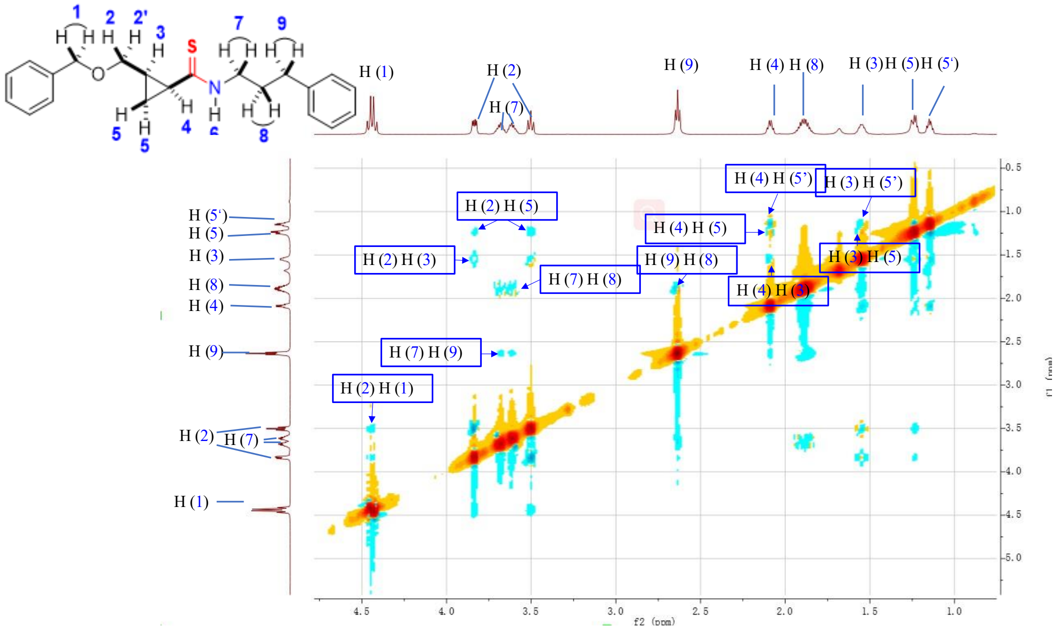

2D HSQC spectrum of **28:**

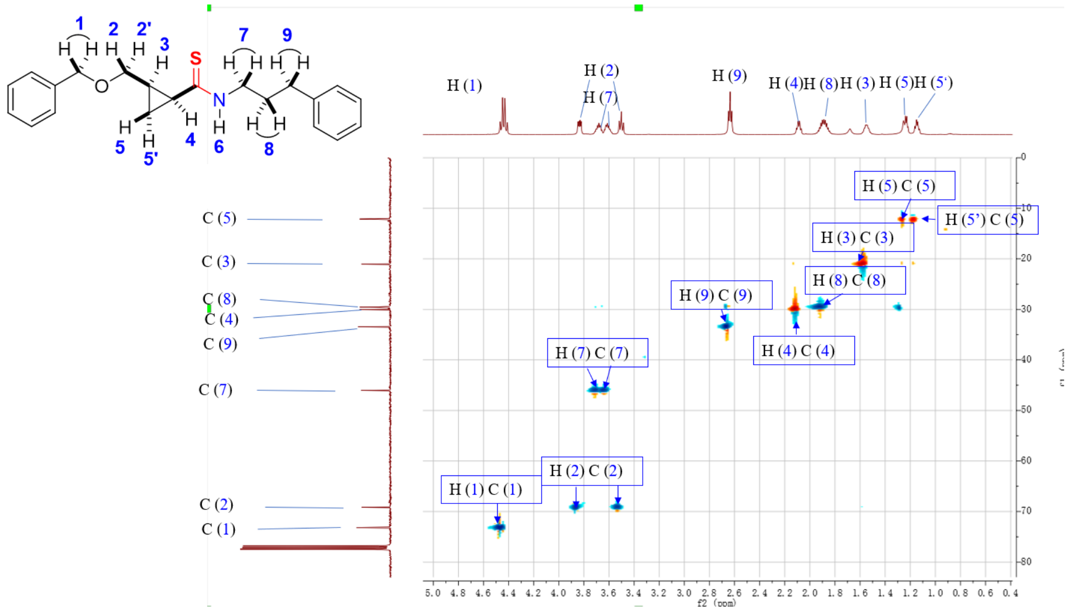

2D HMBC spectrum of **28:**

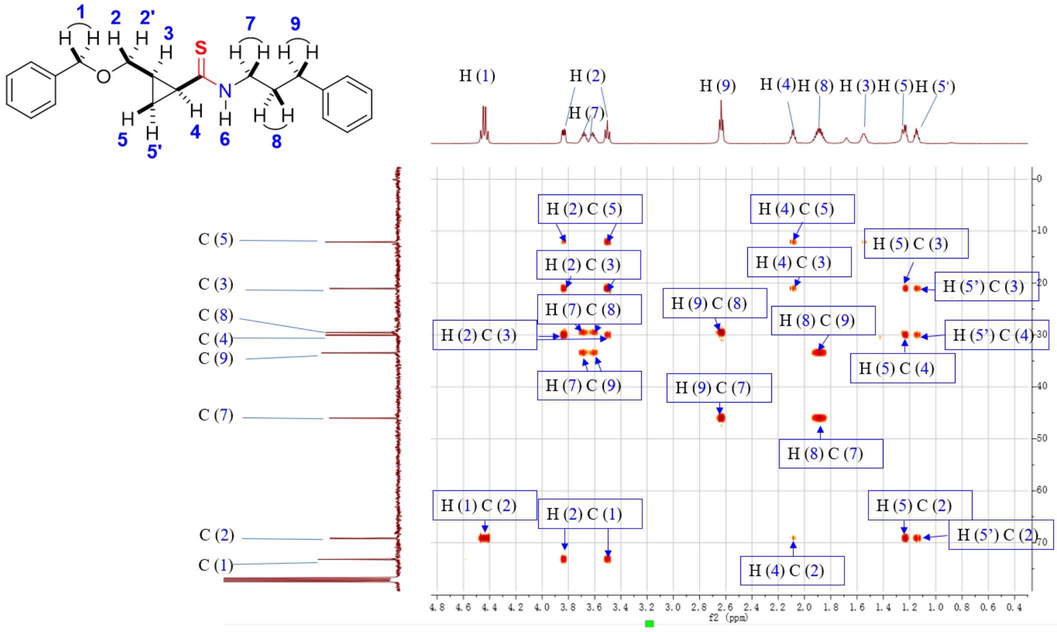

2.2.3 Control reaction to detect the formation of S_3 radical anion.

It has been reported that elemental sulfur is activated in the presence of bases such as Na_2S or NaO^{t}Bu[27] to produce S_3 radical anions which are shown to react with 1,3-diyne **S1** to afford thiophene **S2** (eq 1). We thus used this reaction to provide evidence for the formation of S_3 radical anions.

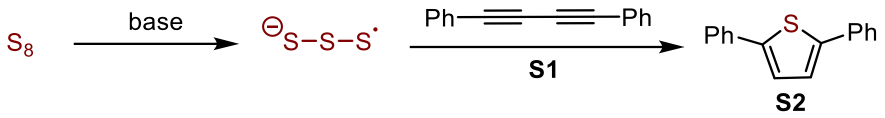

(1)

The results of Na_2S reacts with $\mathbf{S_8}$ and **3d** are summarized in Table S3. In both cases, the thiophene **S2** was formed, which indicated the likely existence of reactive S_3 radical anion species, with **3d**/Na_2S being more effective than $\mathbf{S_8}$/Na_2S at low temperatures.

Supplementary Table S5. Control reactions to detect the formation of S_3 radical anion.[a]

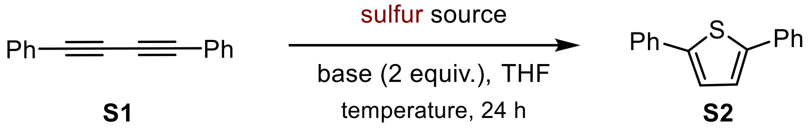

(2)

entry	sulfur source	base	temperature	NMR yield
1	S_8	NaO^{t}Bu	rt	69%
2	S_8	Na_2S	rt	68%
3	S_8	Na_2S	-10 °C	5%
4	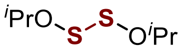 (**3d**)	Na_2S	rt	70%
5	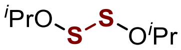 (**3d**)	Na_2S	-10 °C	20%

[a]Unless noted otherwise, reactions were carried out with 0.1 mmol of **S1**, 0.2 mmol of base, and 0.2mmol **S** source in 1 mL of DMF.

In addition, we performed the electron paramagnetic resonance (EPR) experiments (see next page). This demonstrates a single EPR signal assignable to the trisulfur radical

anion observed in the DMF solution of Na_2S with **S_8** and **3d**, respectively, at room temperature. (Ref. 27: *Org. Lett.* **2014**, *16*, 6156.)

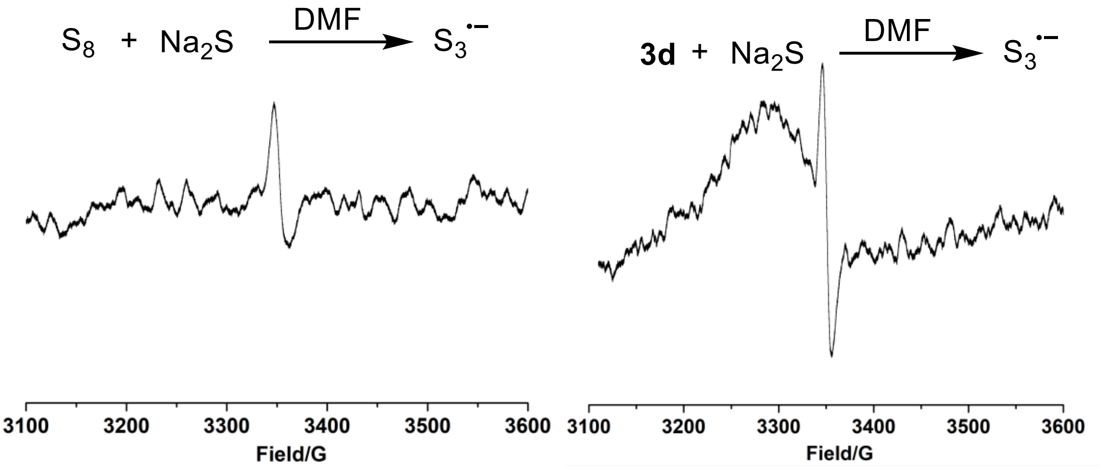

Supplementary Fig. S10 EPR experiment to detect the formation of S_3 radical anion.

Conclusion: Based on the evidence above and manuscript references 48, 49, both **S_8** and the dissulfide **3d** (RSSR, where R = leaving group) can react with Na_2S to form S_3 radical anions. Thus, a similar mechanism to Fig. 5f in the manuscript is proposed to explain thiopeptide formation when using **3d.**

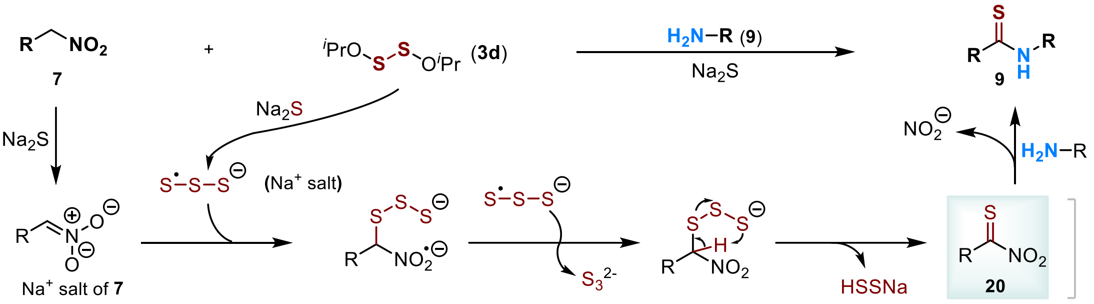

Supplementary Fig. S11 Proposed reaction pathway in the conversion of primary nitroalkanes to thioapeptides via thioacylating species **20** using **3d**.

3. Supplementary Notes

3.1 NMR spectra

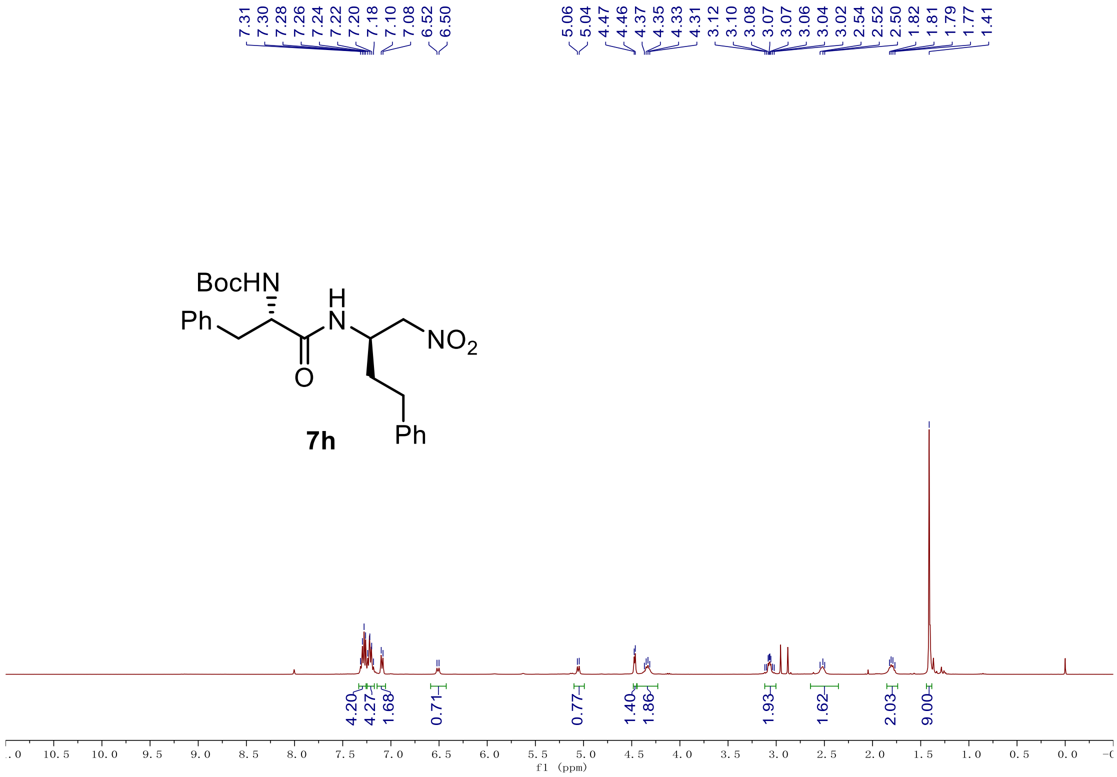

Supplementary Fig. S12 ^{1}H NMR spectrum of compound **7h** (400 MHz, $CDCl_3$)

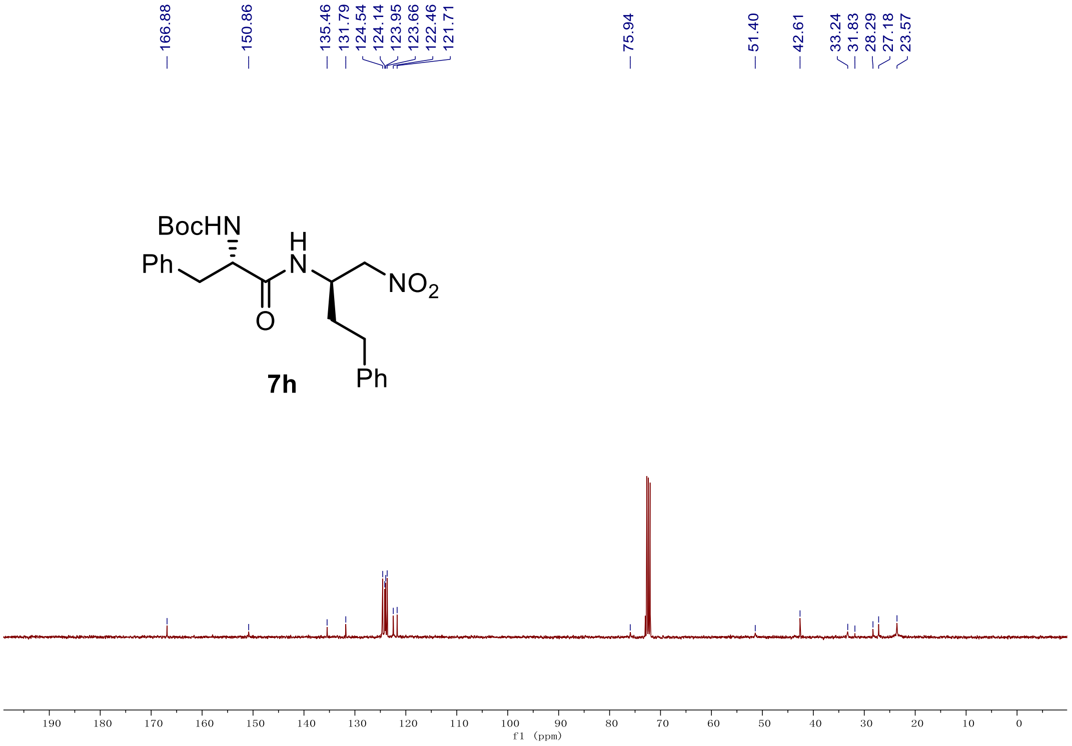

Supplementary Fig. S13 ^{13}C NMR spectrum of compound **7h** (101 MHz, $CDCl_3$)

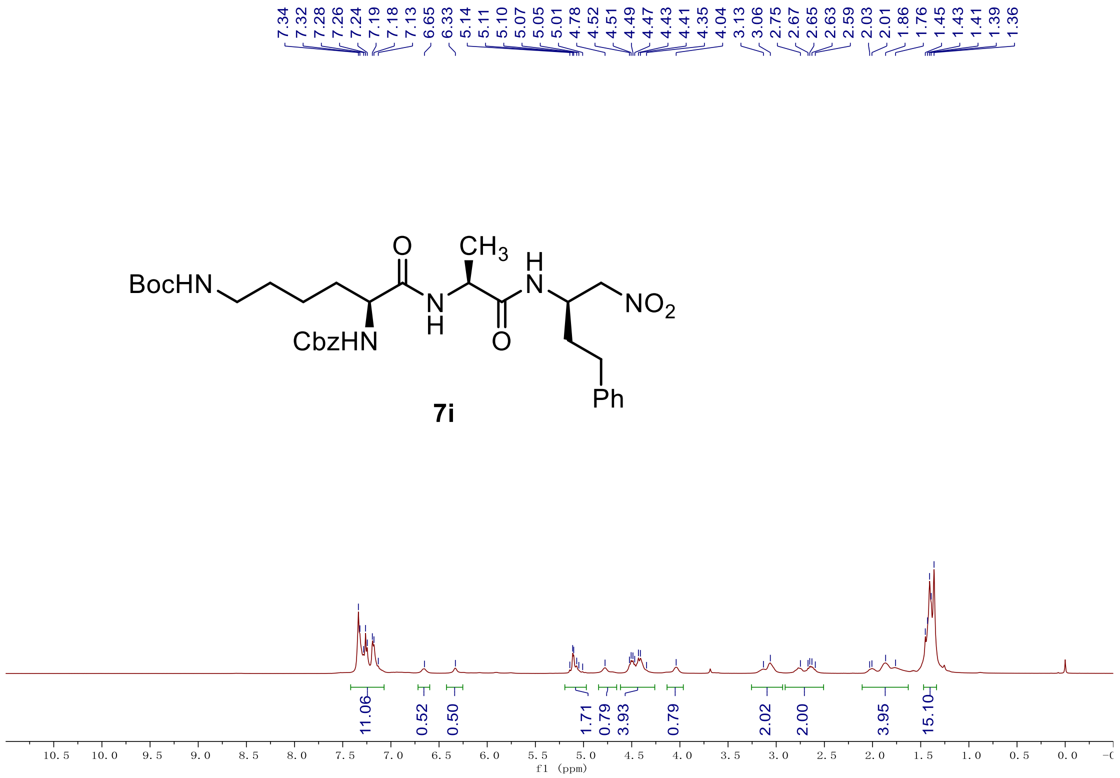

Supplementary Fig. S14 ^{1}H NMR spectrum of compound **7i** (400 MHz, $CDCl_3$)

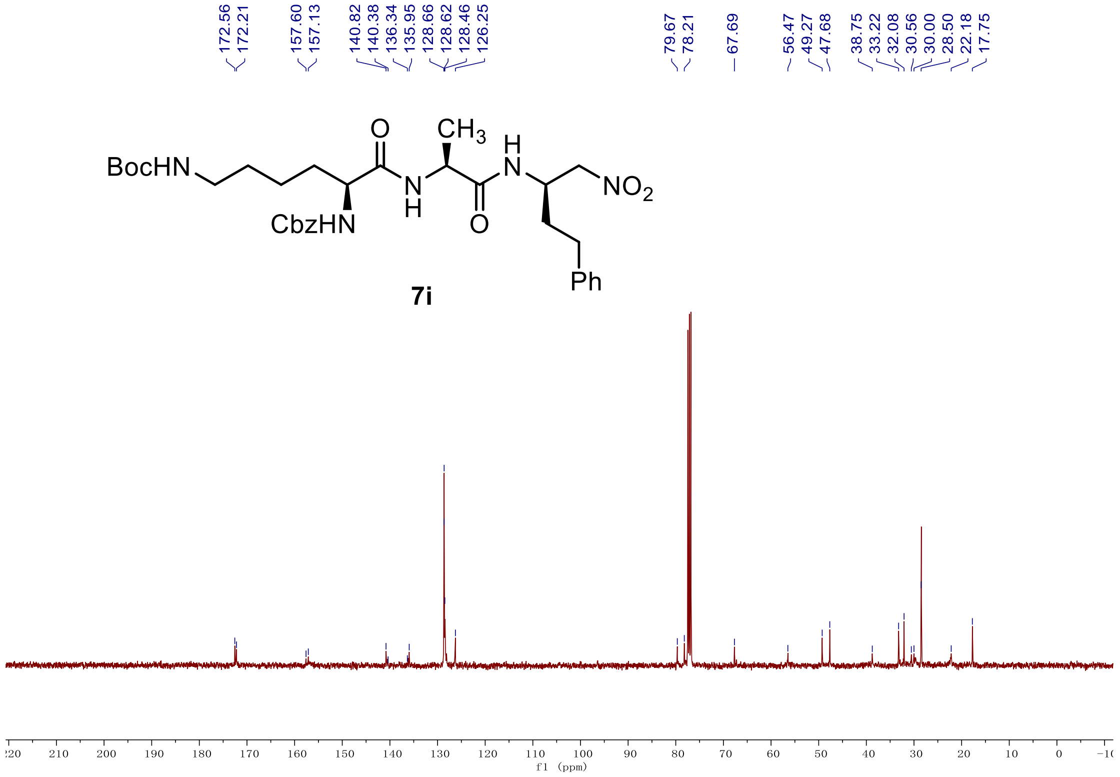

Supplementary Fig. S15 ^{13}C NMR spectrum of compound **7i** (101 MHz, $CDCl_3$)

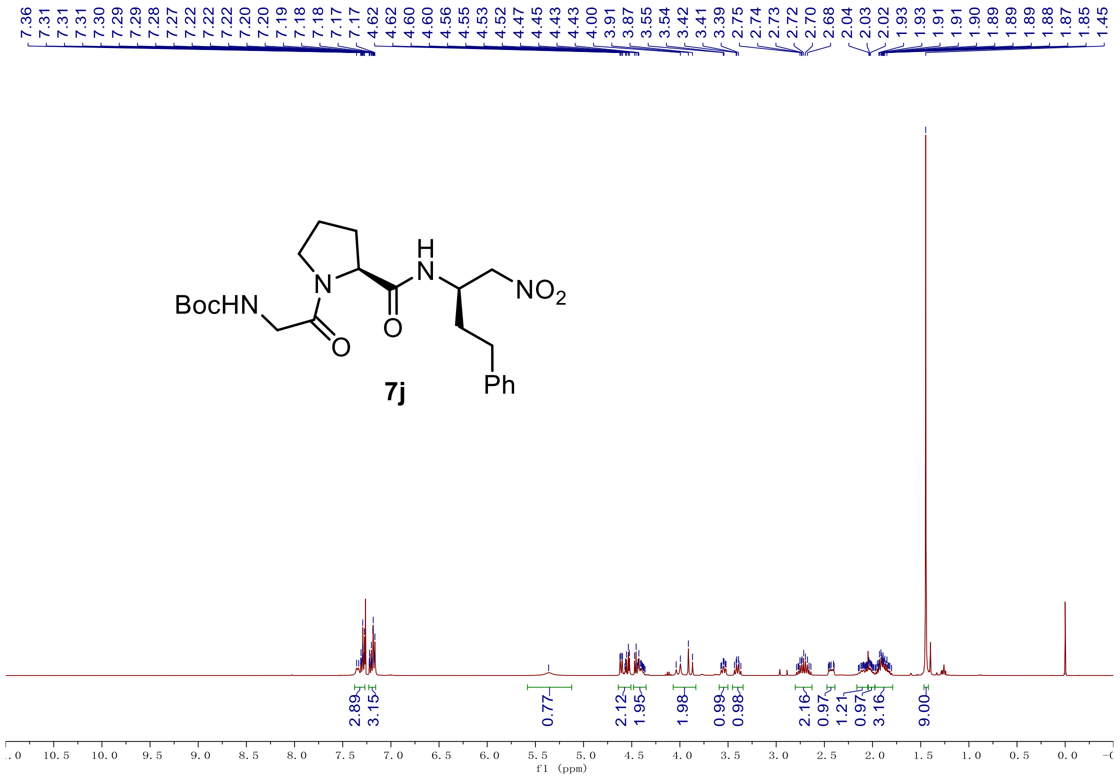

Supplementary Fig. S16 ^{1}H NMR spectrum of compound **7j** (400 MHz, $CDCl_3$)

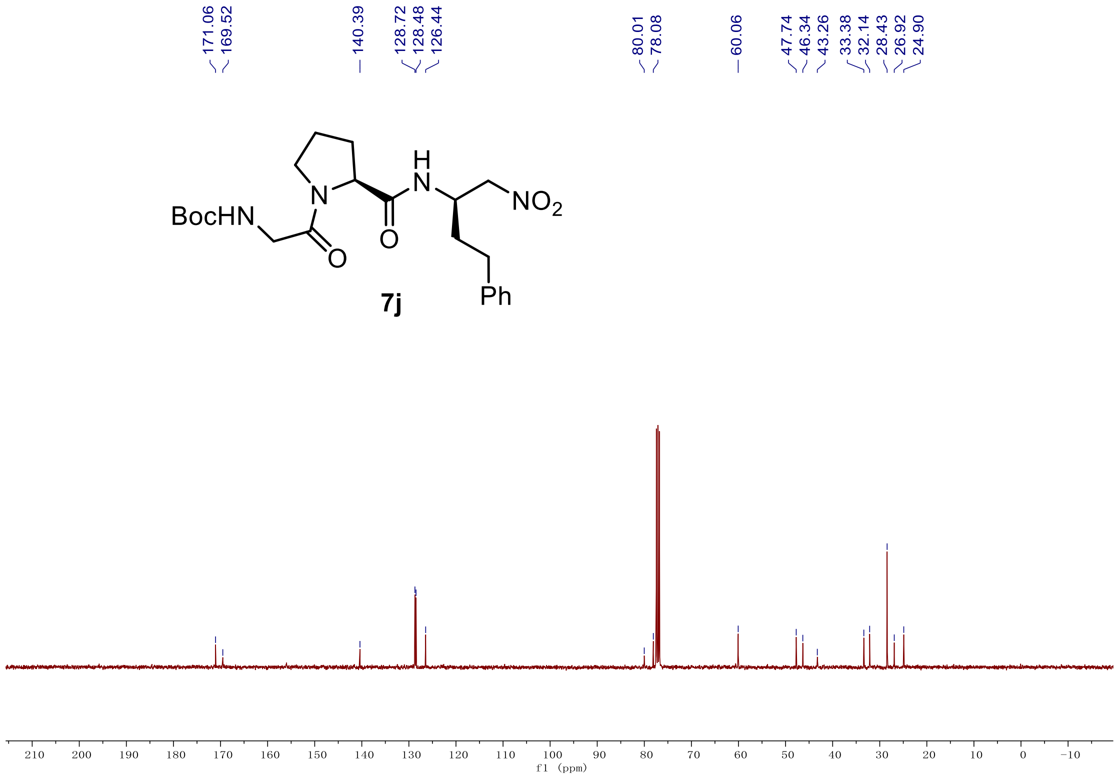

Supplementary Fig. S17 ^{13}C NMR spectrum of compound **7j** (101 MHz, $CDCl_3$)

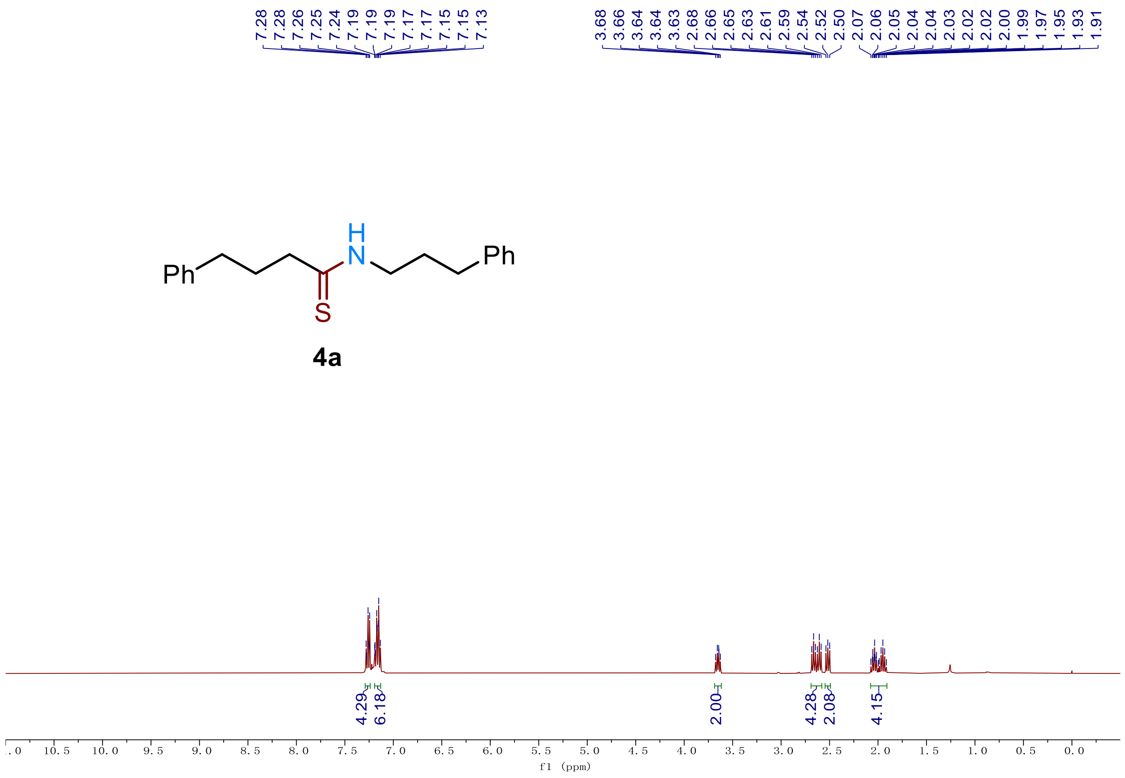

Supplementary Fig. S18 ^{1}H NMR spectrum of compound **4a** (400 MHz, $CDCl_3$)

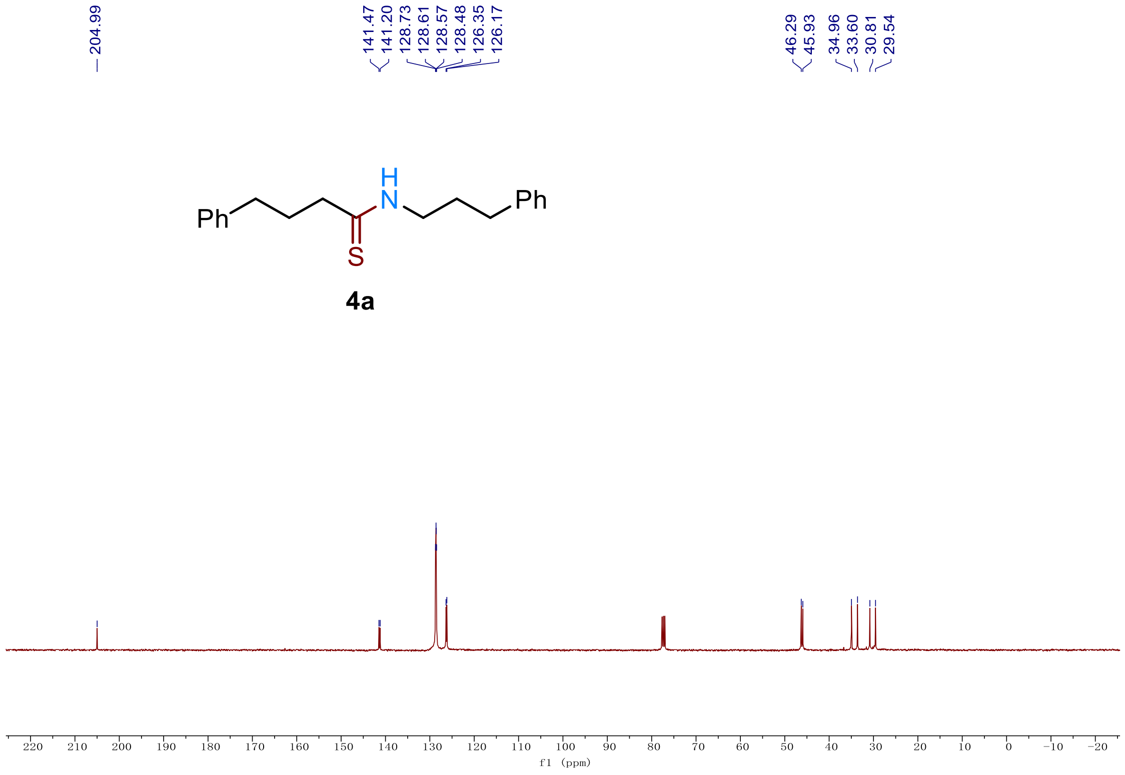

Supplementary Fig. S19 ^{13}C NMR spectrum of compound **4a** (101 MHz, $CDCl_3$)

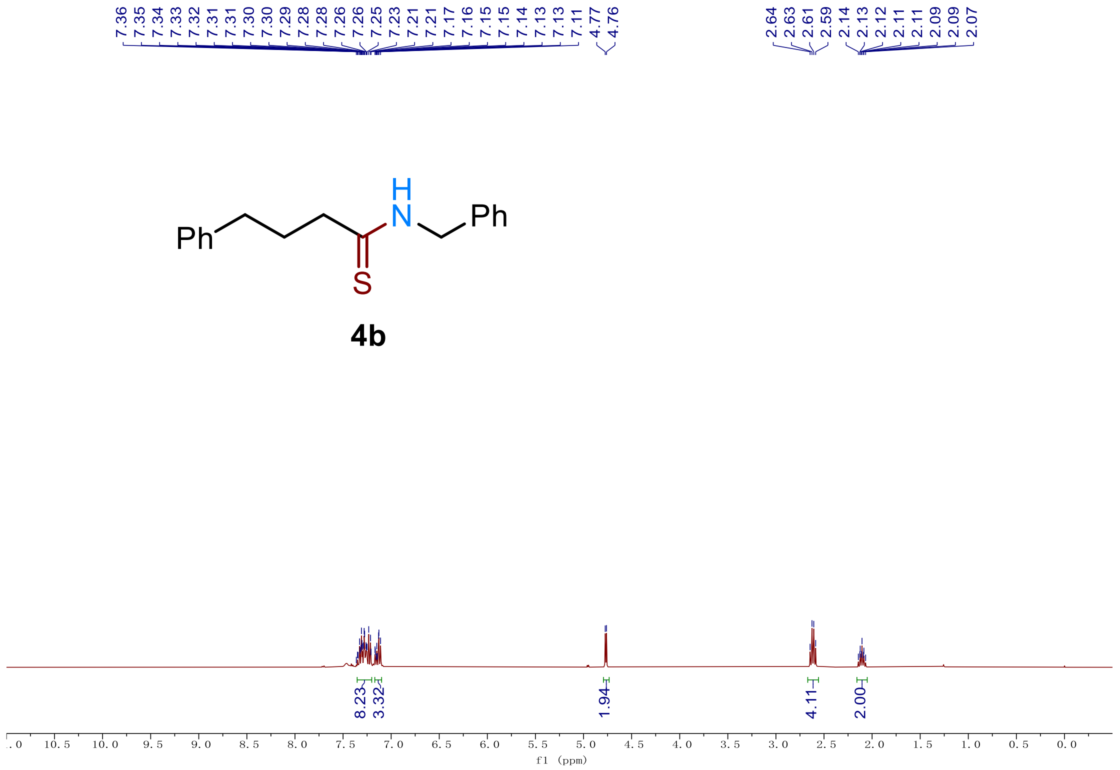

Supplementary Fig. S20 ^{1}H NMR spectrum of compound **4b** (400 MHz, $CDCl_3$)

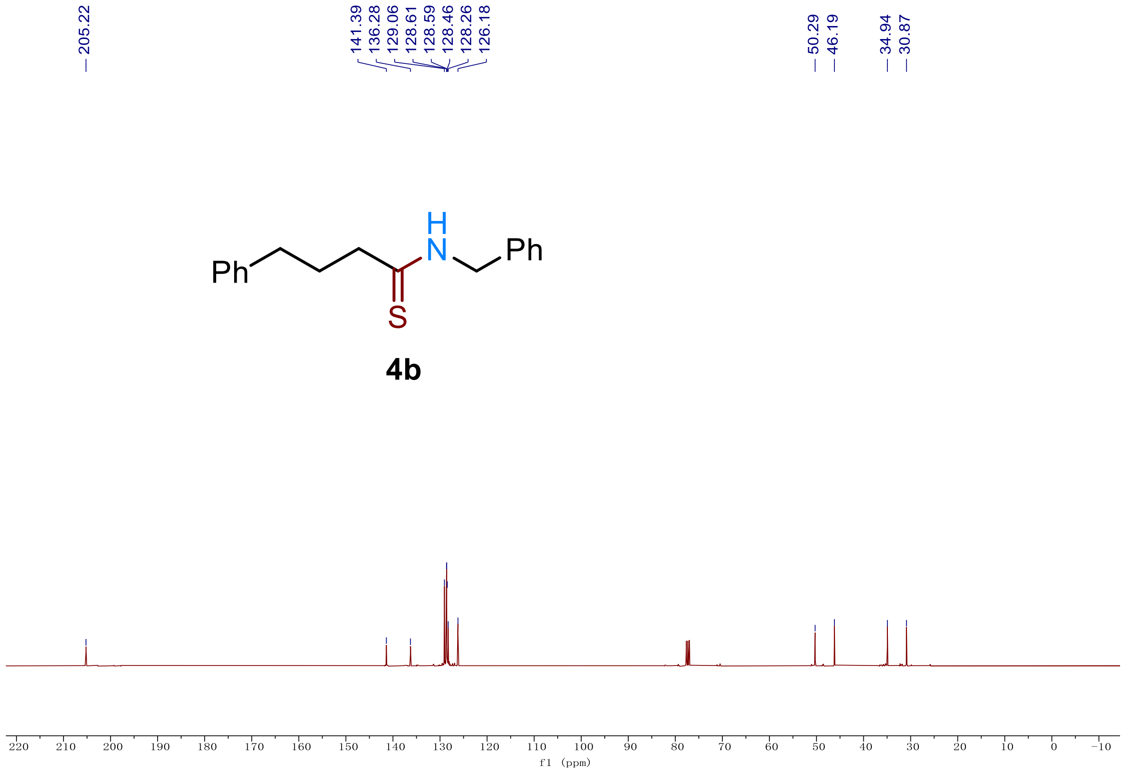

Supplementary Fig. S21 ^{13}C NMR spectrum of compound **4b** (101 MHz, $CDCl_3$)

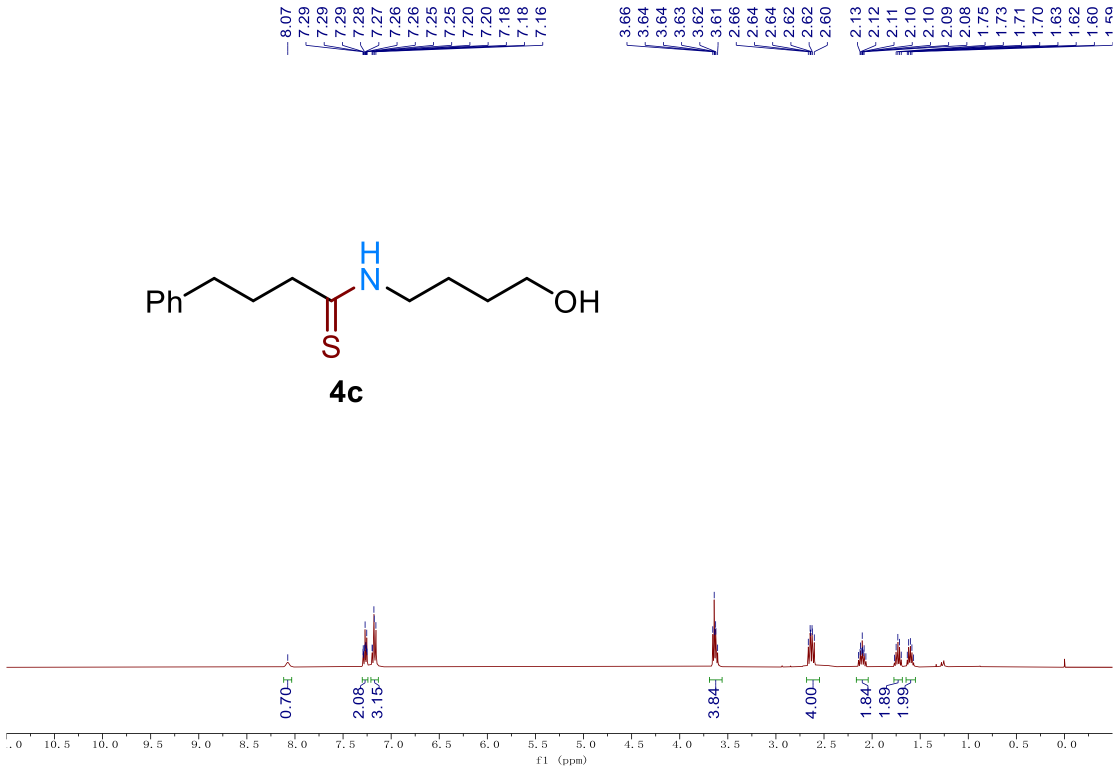

Supplementary Fig. S22 ^{1}H NMR spectrum of compound **4c** (400 MHz, $CDCl_3$)

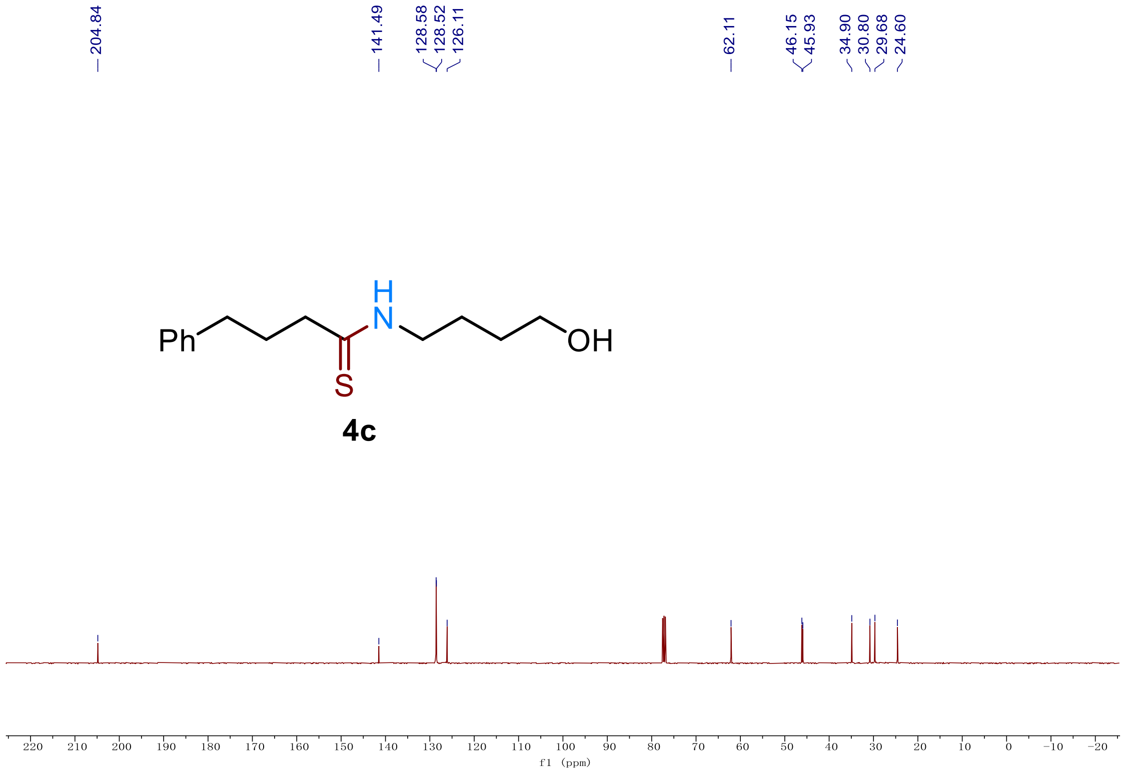

Supplementary Fig. S23 ^{13}C NMR spectrum of compound **4c** (101 MHz, $CDCl_3$)

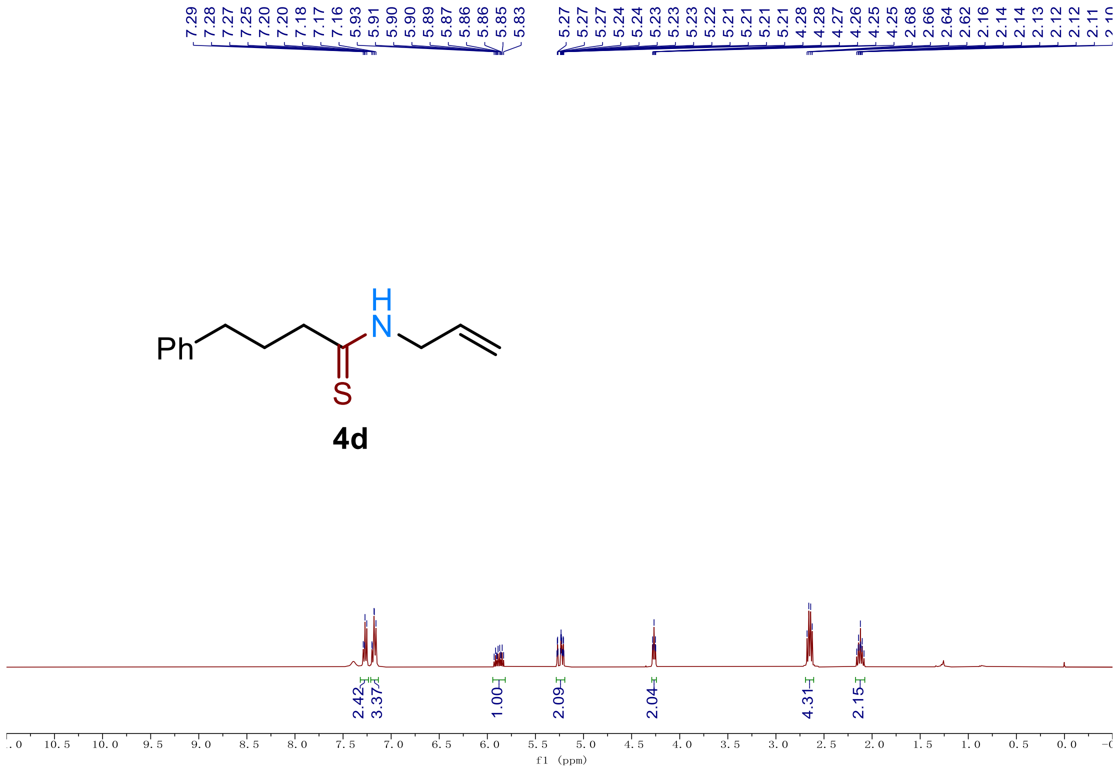

Supplementary Fig. S24 ^{1}H NMR spectrum of compound **4d** (400 MHz, $CDCl_3$)

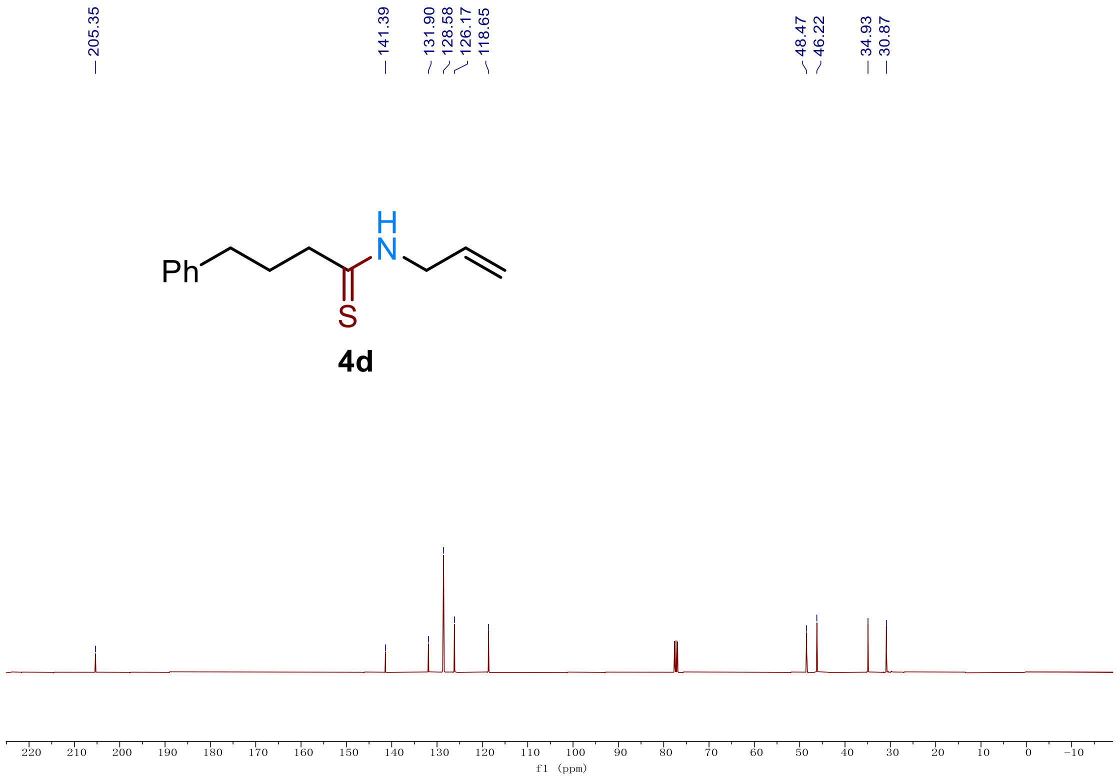

Supplementary Fig. S25 ^{13}C NMR spectrum of compound **4d** (101 MHz, $CDCl_3$)

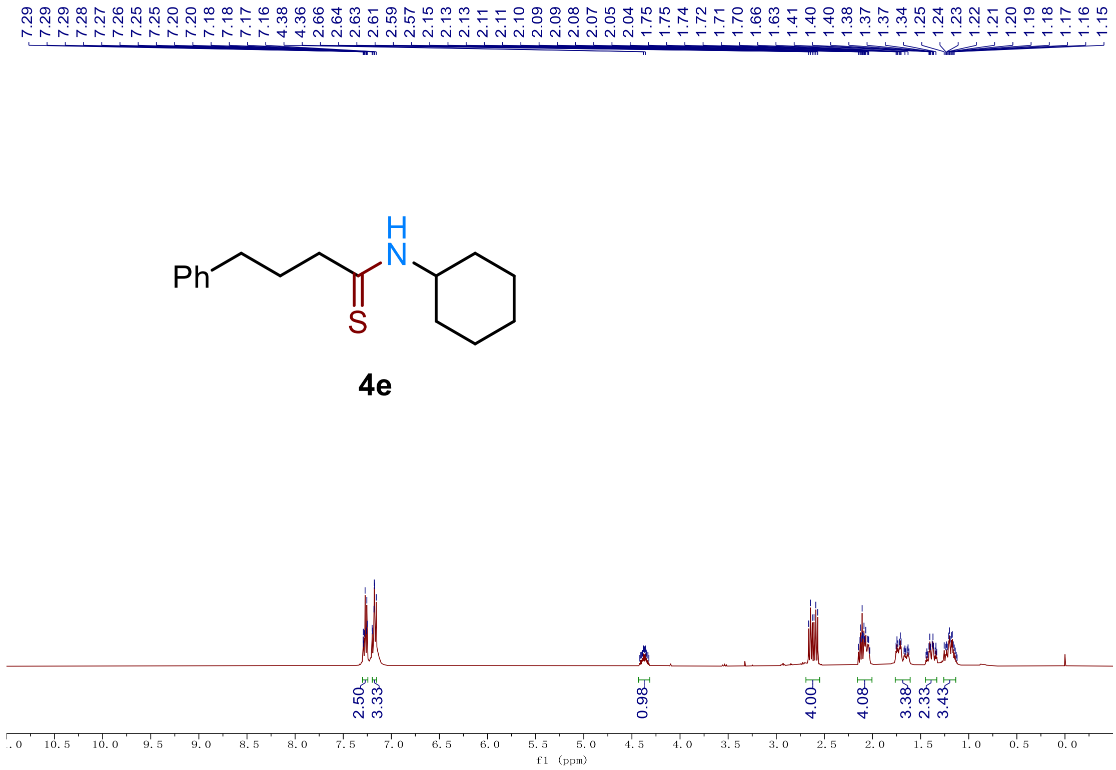

Supplementary Fig. S26 ^{1}H NMR spectrum of compound **4e** (400 MHz, $CDCl_3$)

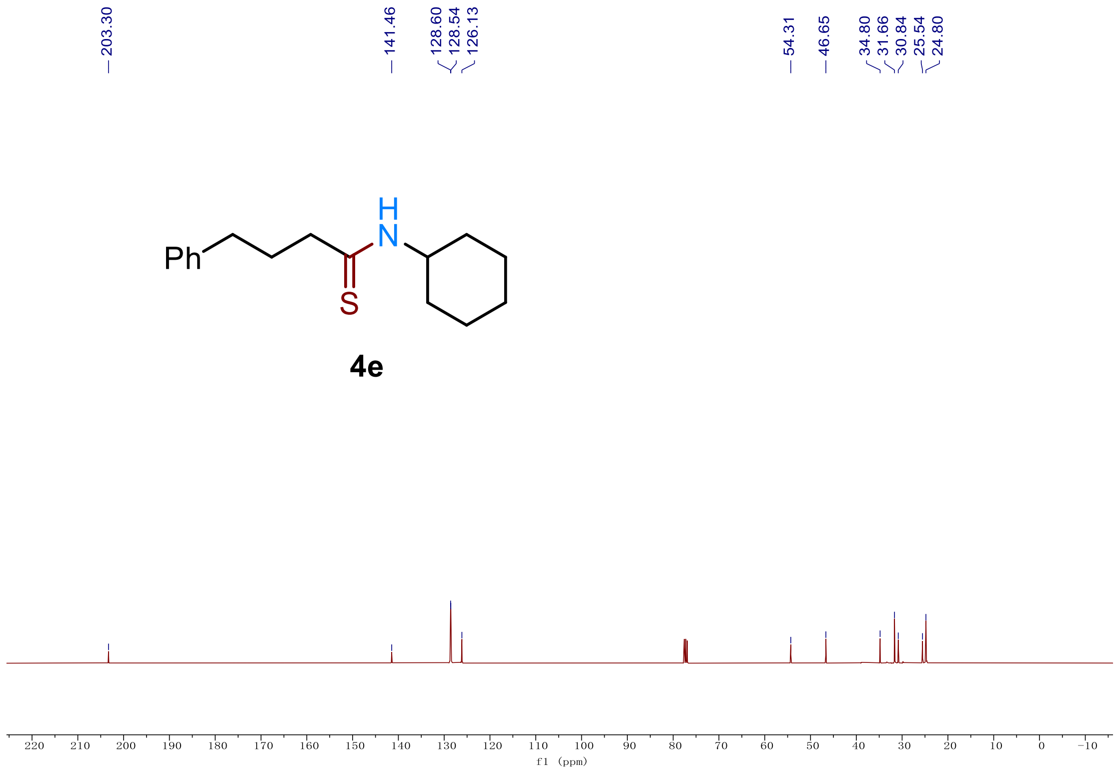

Supplementary Fig. S27 ^{13}C NMR spectrum of compound **4e** (101 MHz, $CDCl_3$)

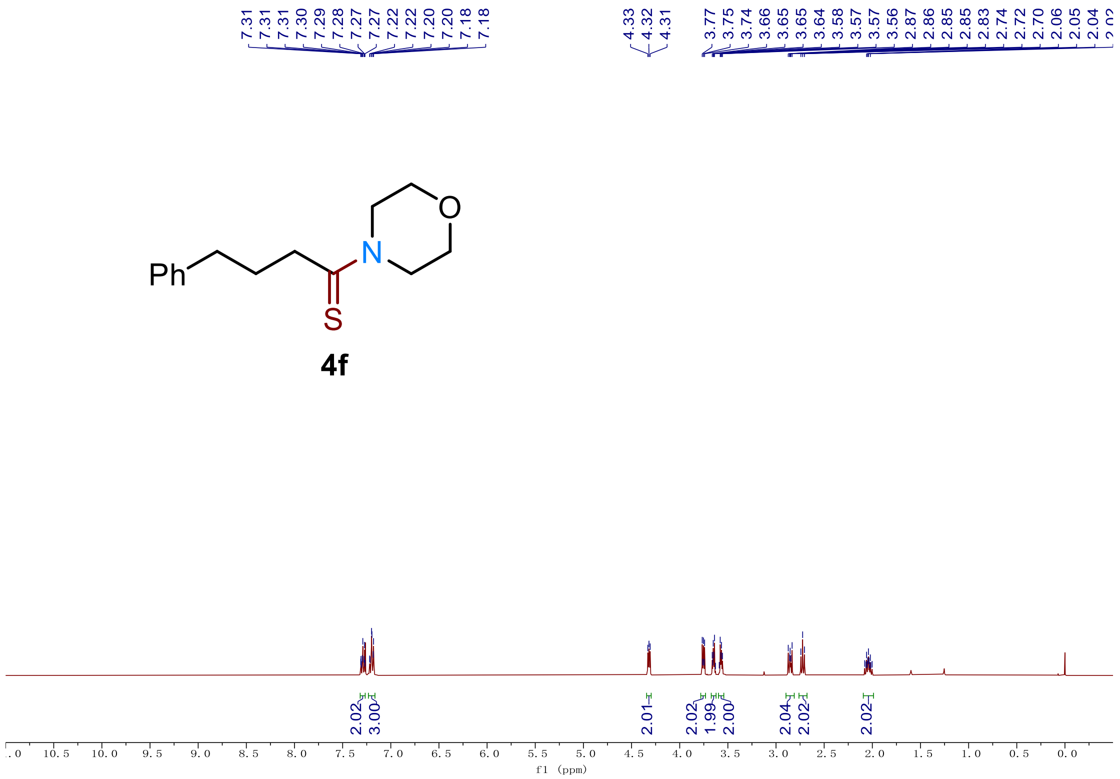

Supplementary Fig. S28 ^{1}H NMR spectrum of compound **4f** (400 MHz, $CDCl_3$)

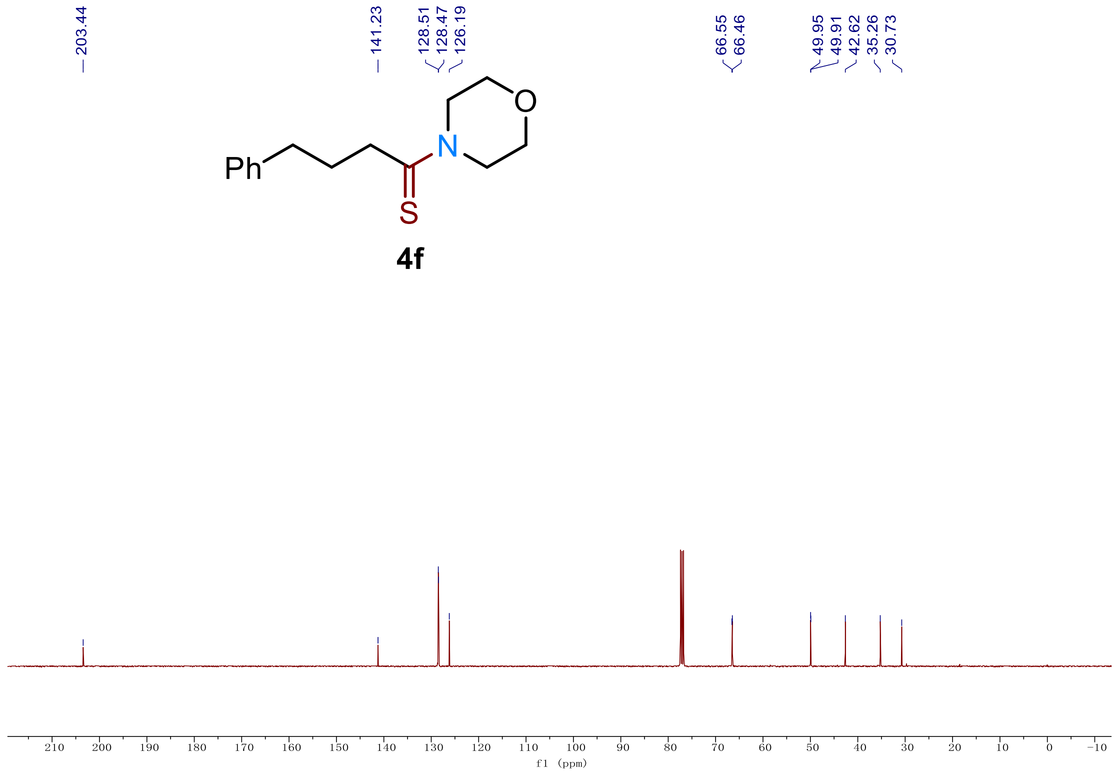

Supplementary Fig. S29 ^{13}C NMR spectrum of compound **4f** (101 MHz, $CDCl_3$)

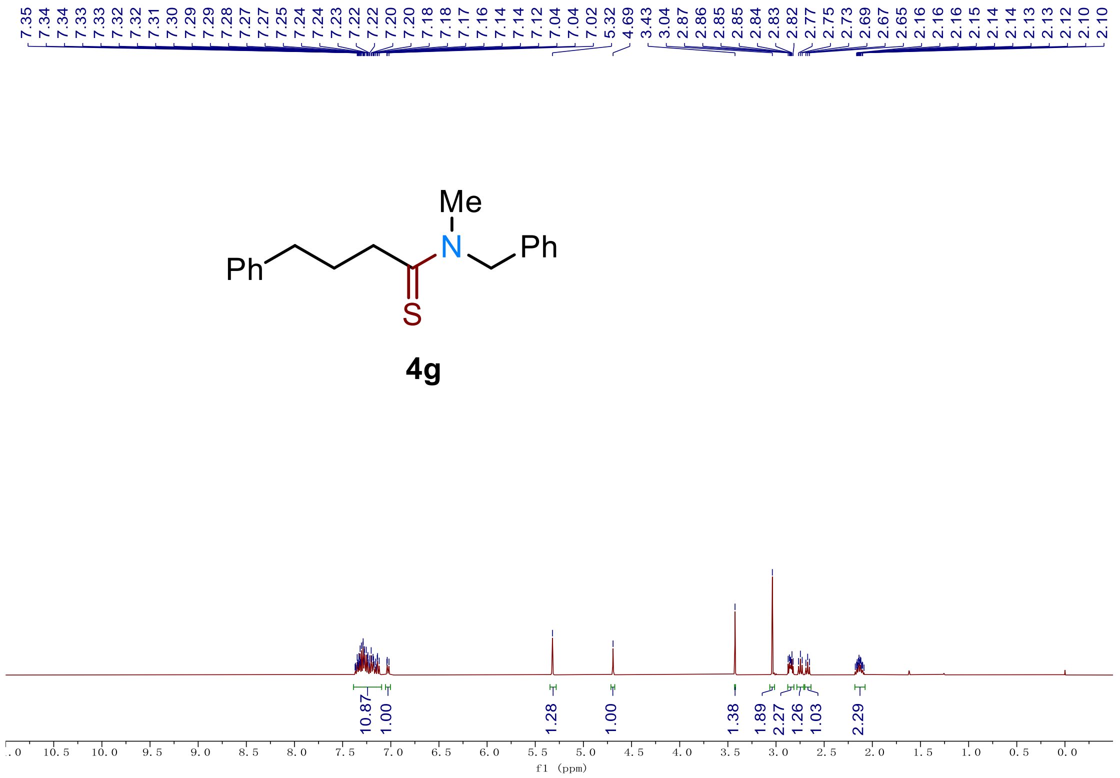

Supplementary Fig. S30 ^{1}H NMR spectrum of compound **4g** (400 MHz, $CDCl_3$)

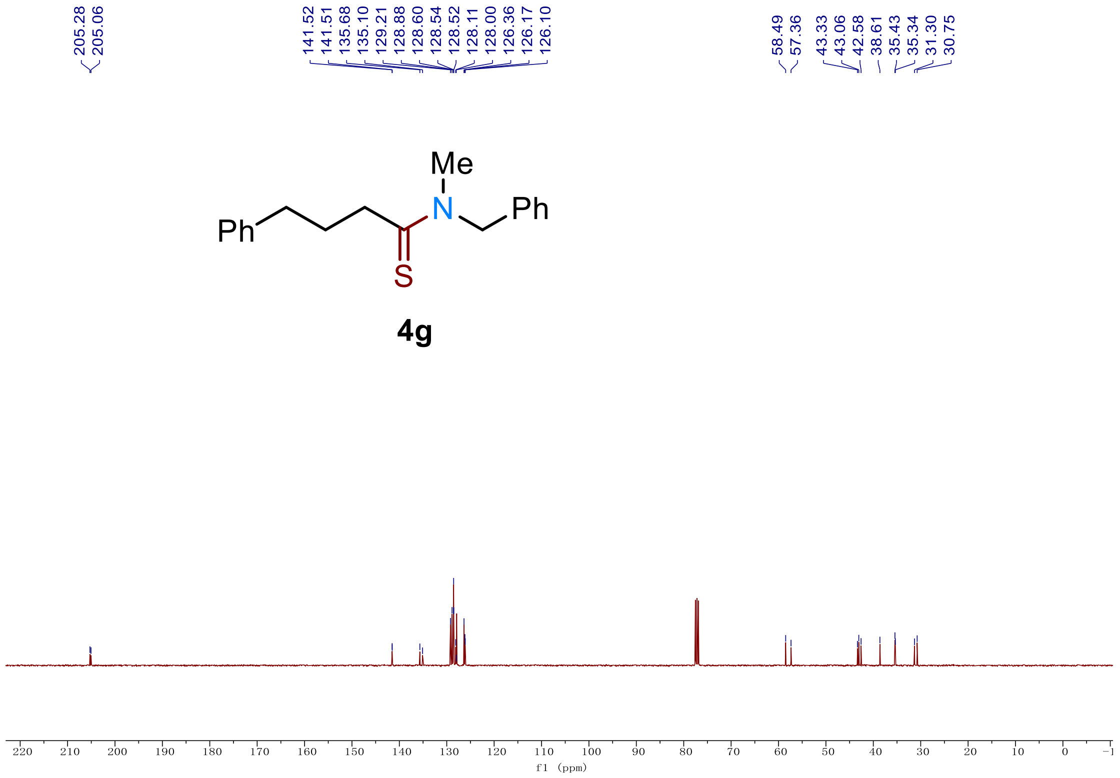

Supplementary Fig. S31 ^{13}C NMR spectrum of compound **4g** (101 MHz, $CDCl_3$)

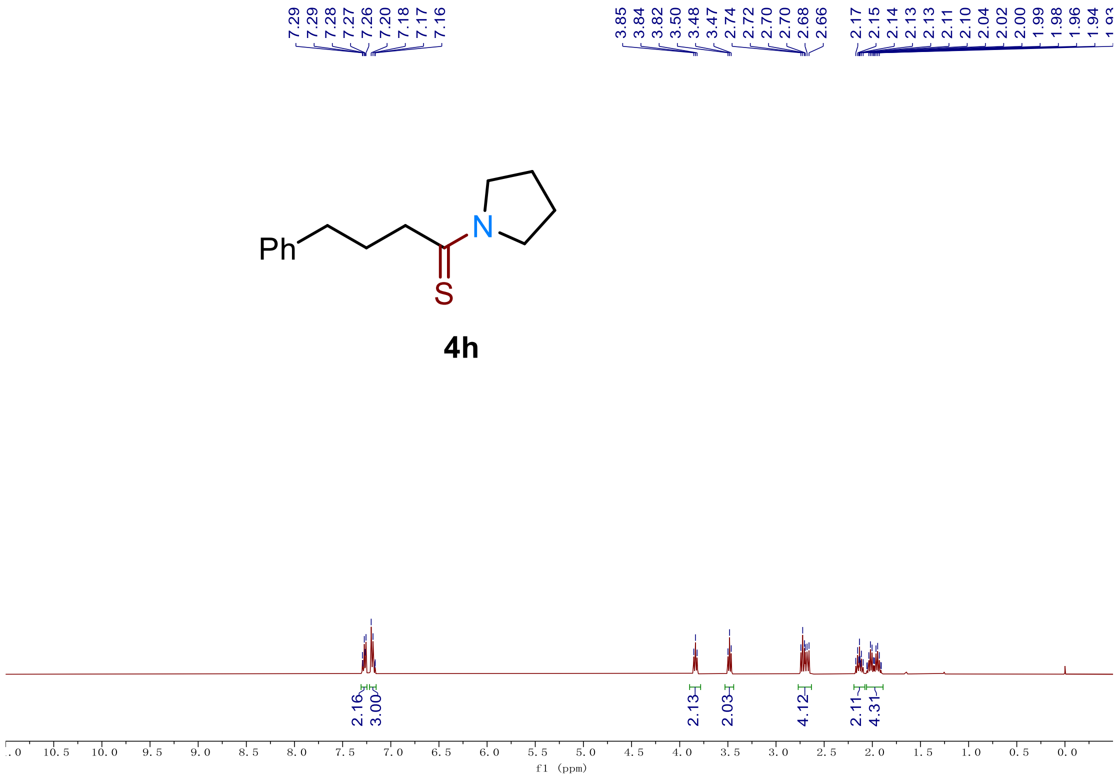

Supplementary Fig. S32 ^{1}H NMR spectrum of compound **4h** (400 MHz, $CDCl_3$)

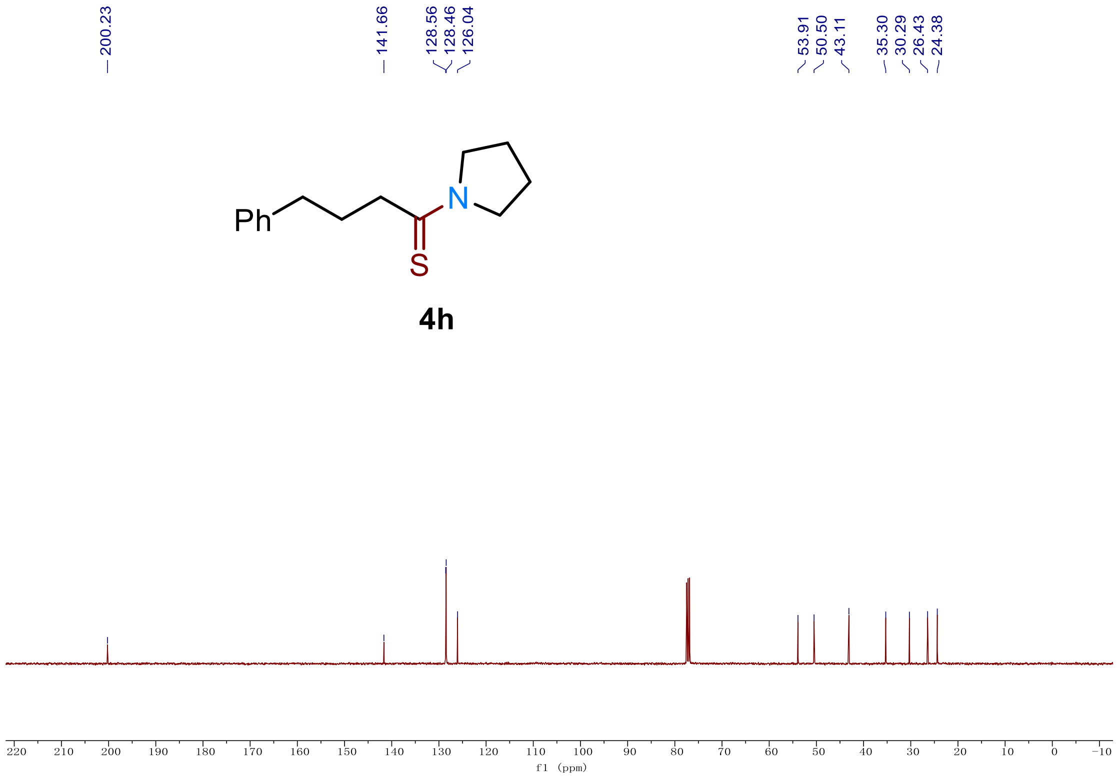

Supplementary Fig. S33 ^{13}C NMR spectrum of compound **4h** (101 MHz, $CDCl_3$)

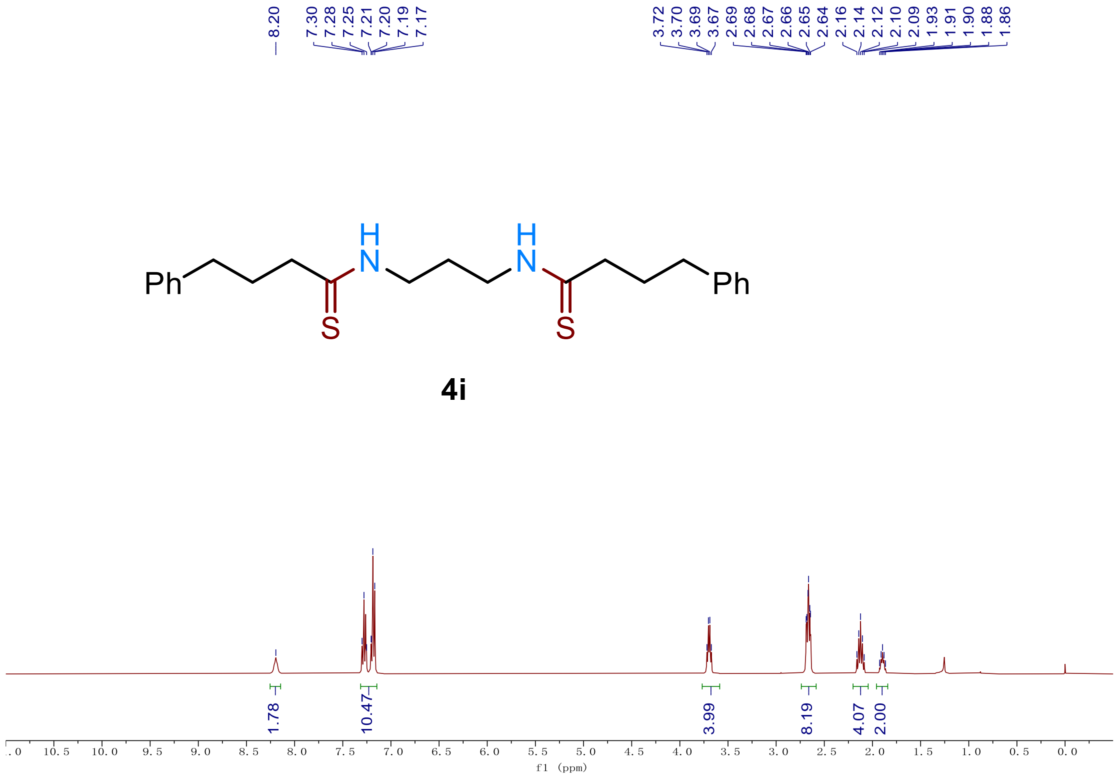

Supplementary Fig. S34 ^{1}H NMR spectrum of compound **4i** (400 MHz, $CDCl_3$)

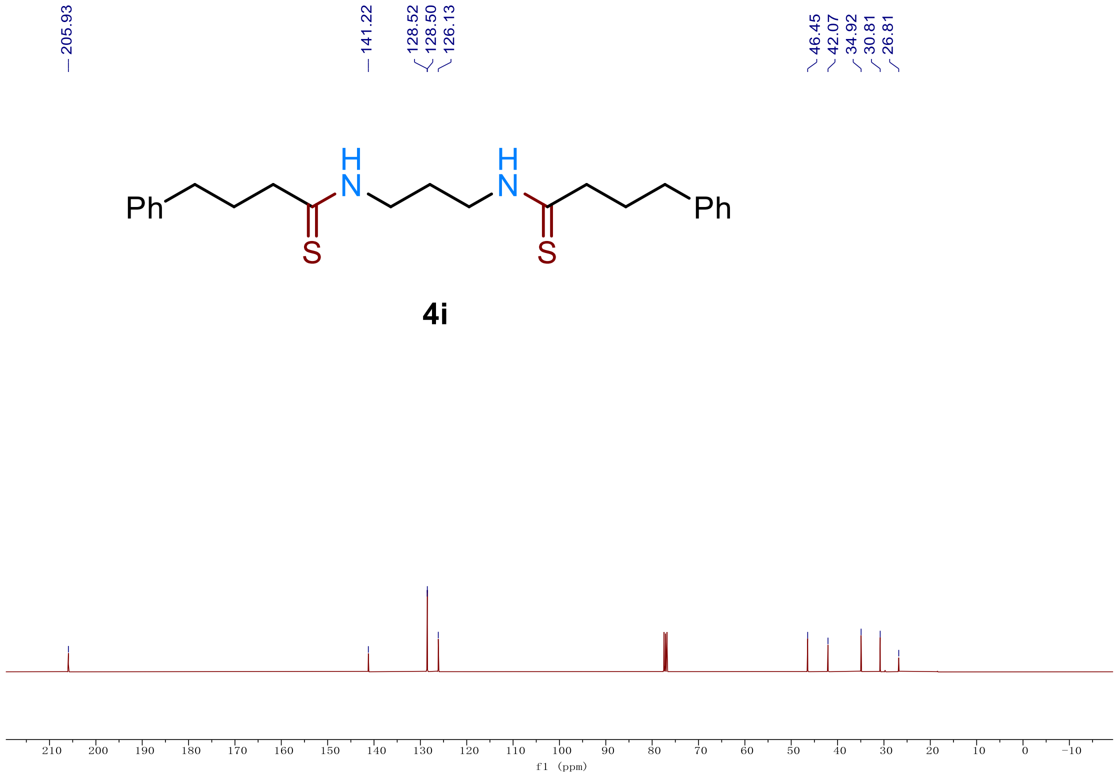

Supplementary Fig. S35 ^{13}C NMR spectrum of compound **4i** (101 MHz, $CDCl_3$)

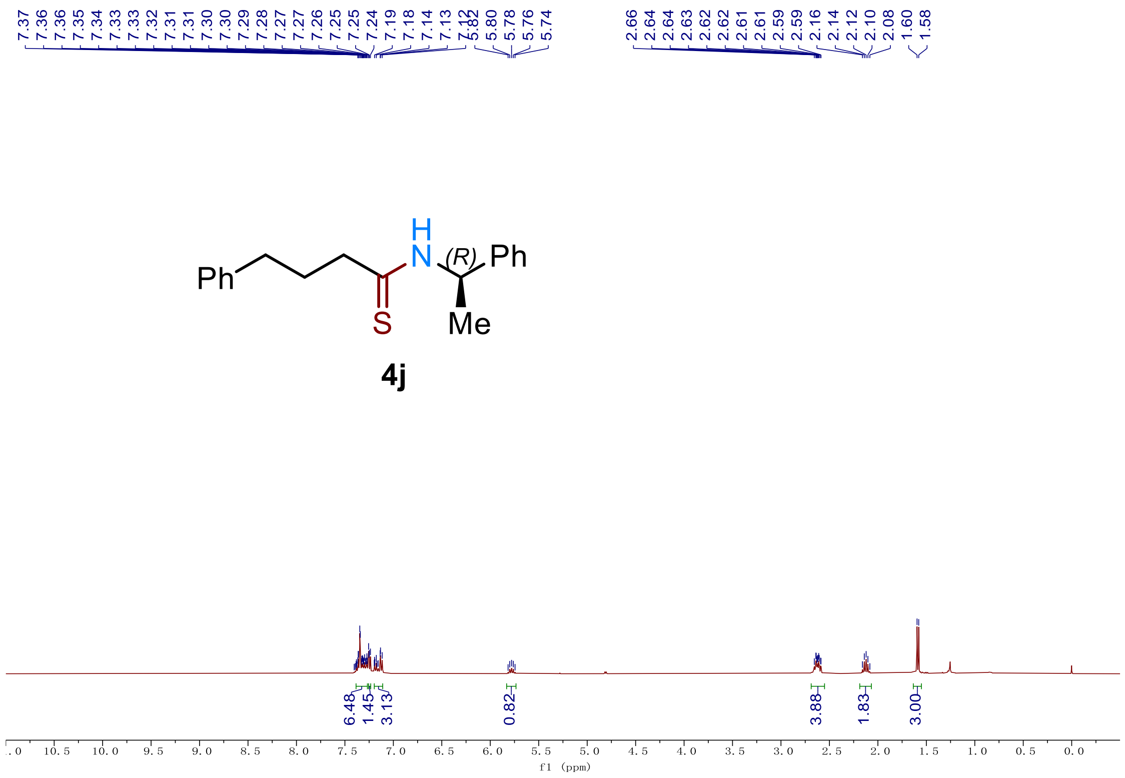

Supplementary Fig. S36 ^{1}H NMR spectrum of compound **4j** (400 MHz, $CDCl_3$)

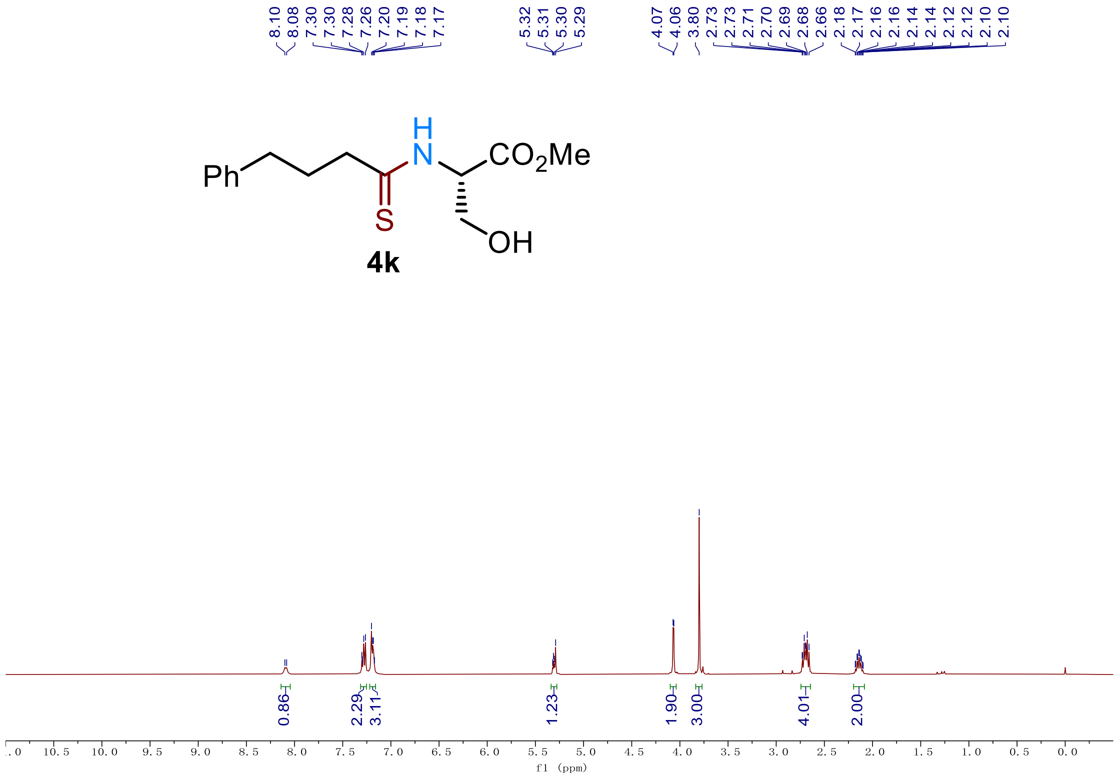

Supplementary Fig. S37 ^{1}H NMR spectrum of compound **4k** (400 MHz, $CDCl_3$)

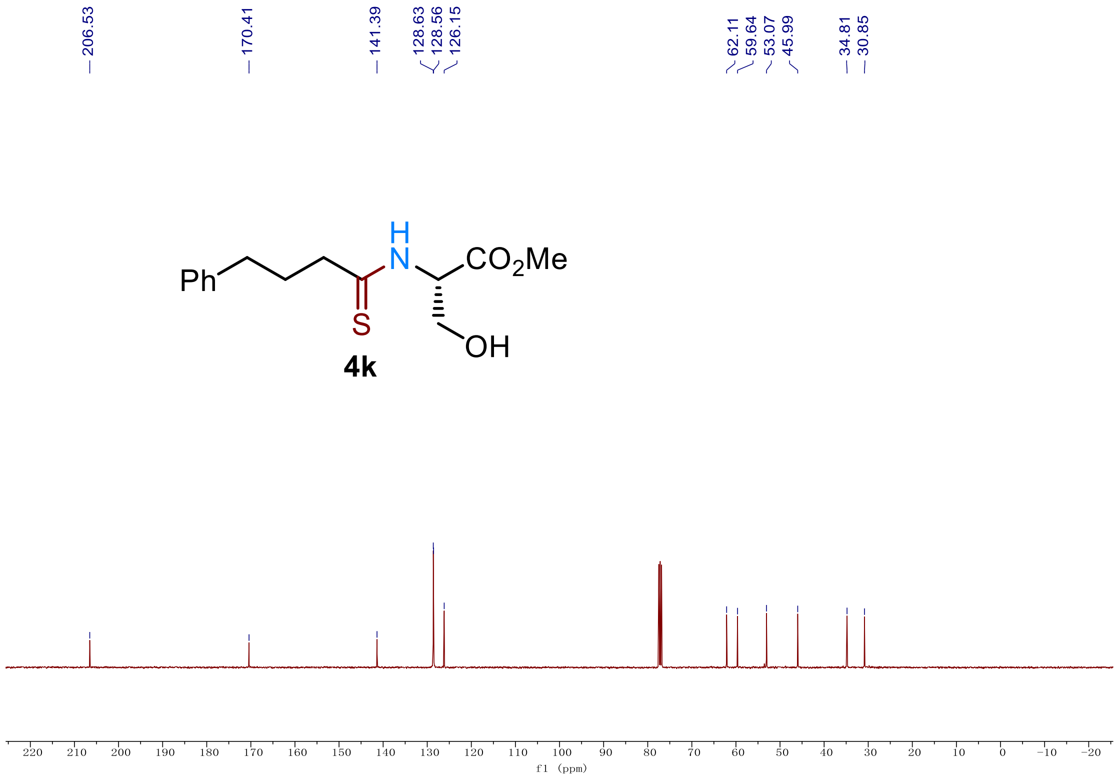

Supplementary Fig. S38 ^{13}C NMR spectrum of compound **4k** (101 MHz, $CDCl_3$)

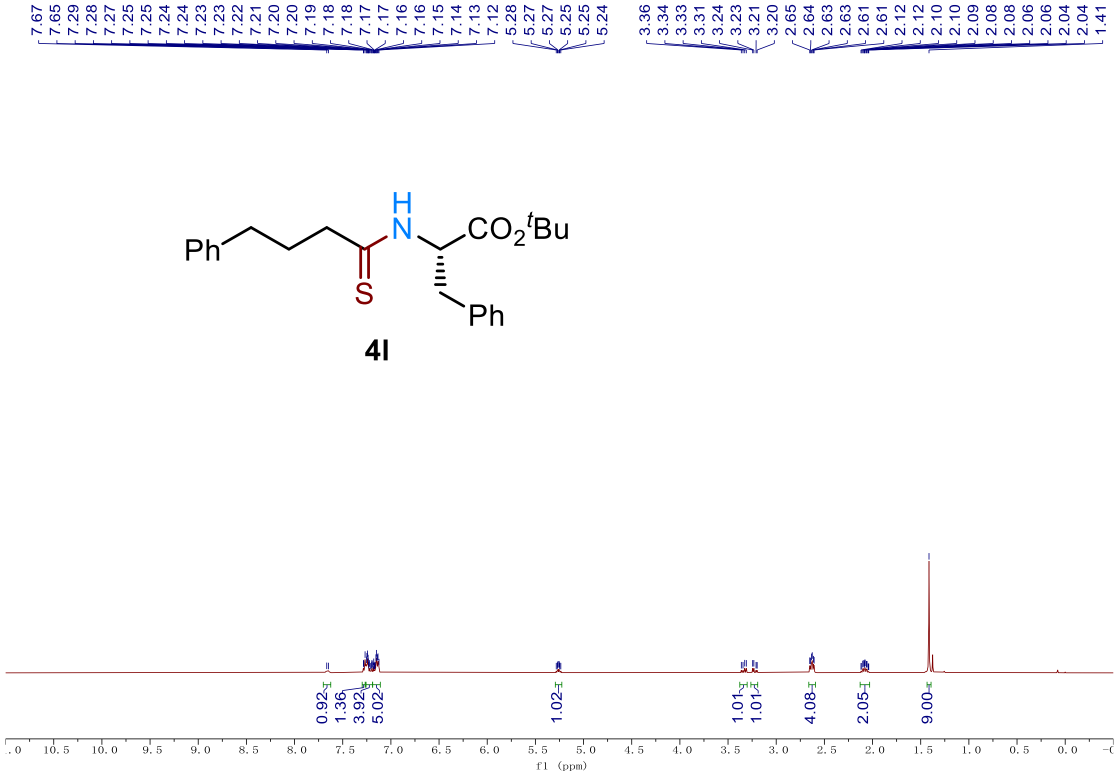

Supplementary Fig. S39 ^{1}H NMR spectrum of compound **4l** (400 MHz, $CDCl_3$)

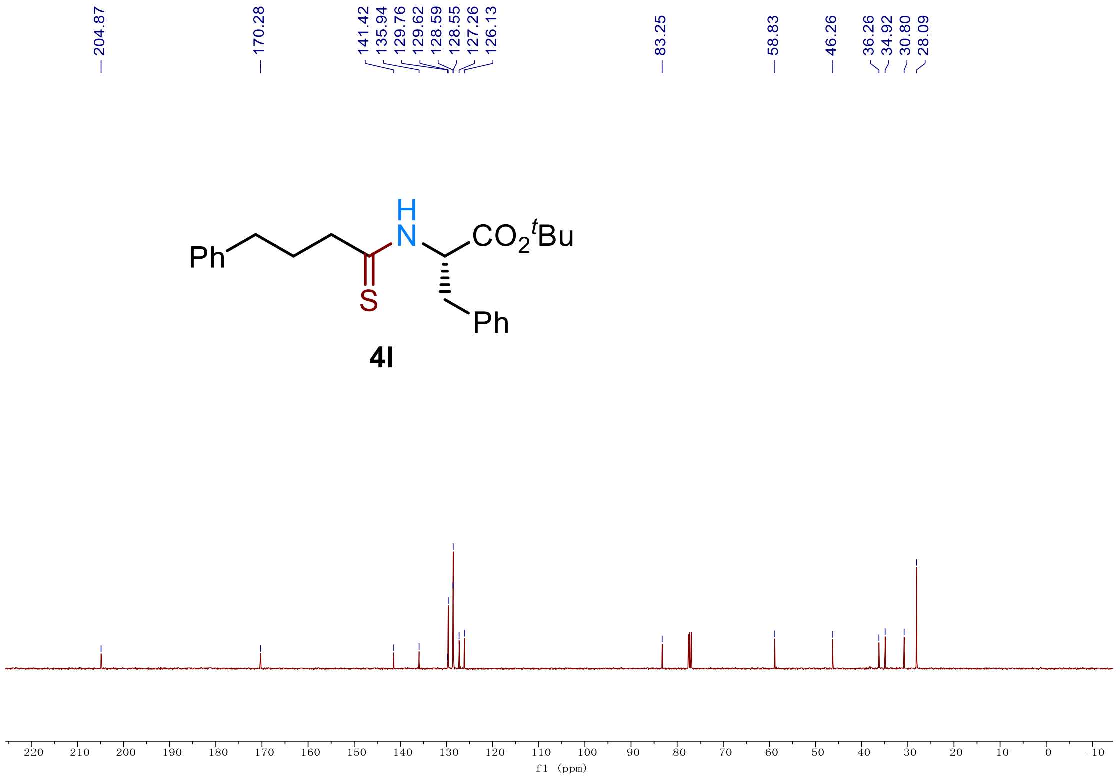

Supplementary Fig. S40 ^{13}C NMR spectrum of compound **4l** (101 MHz, $CDCl_3$)

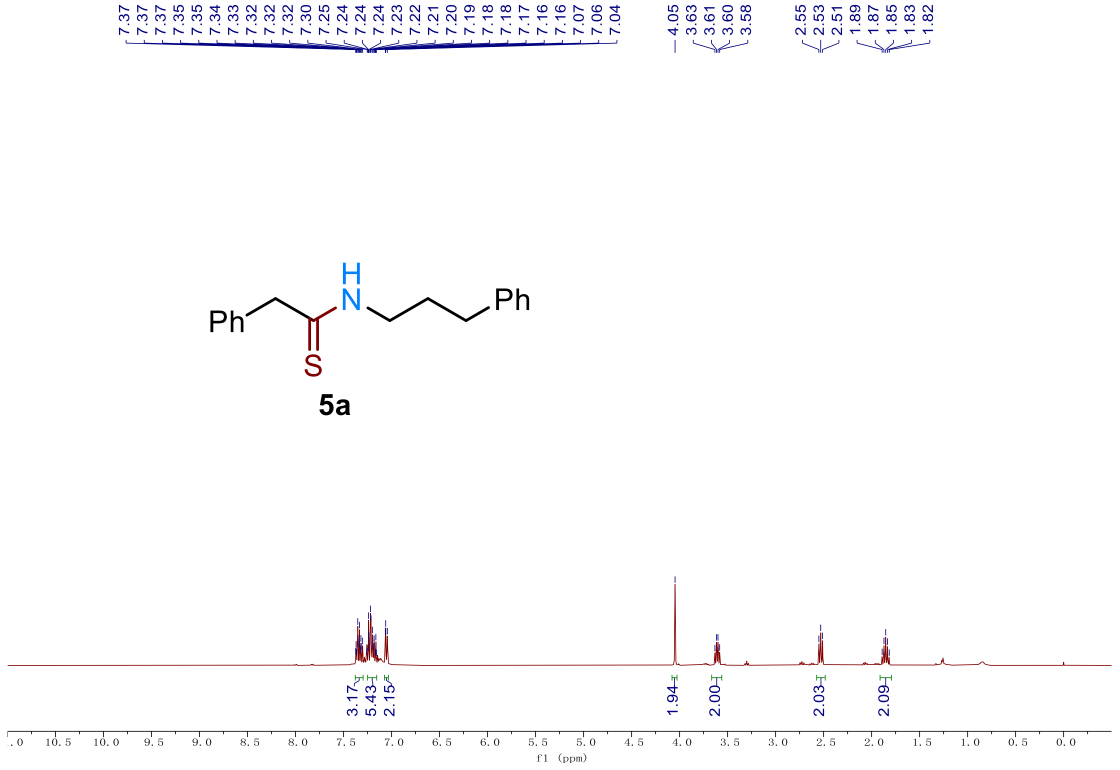

Supplementary Fig. S41 ^{1}H NMR spectrum of compound **5a** (400 MHz, $CDCl_3$)

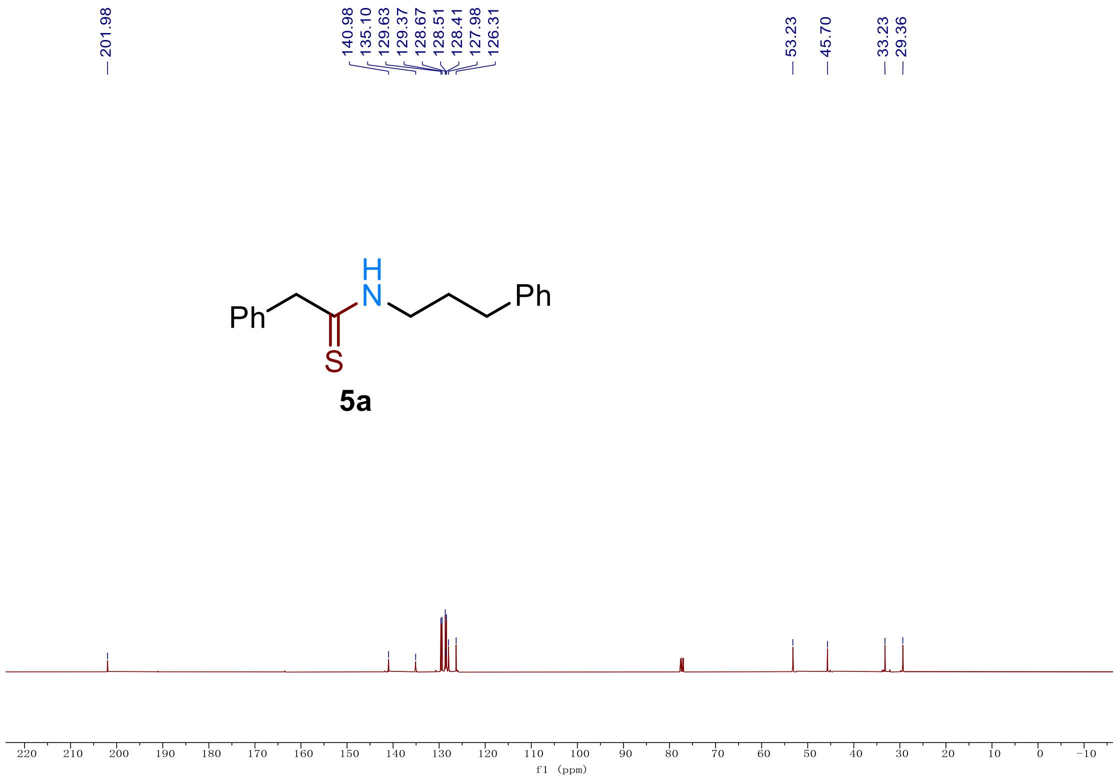

Supplementary Fig. S42 ^{13}C NMR spectrum of compound **5a** (101 MHz, $CDCl_3$)

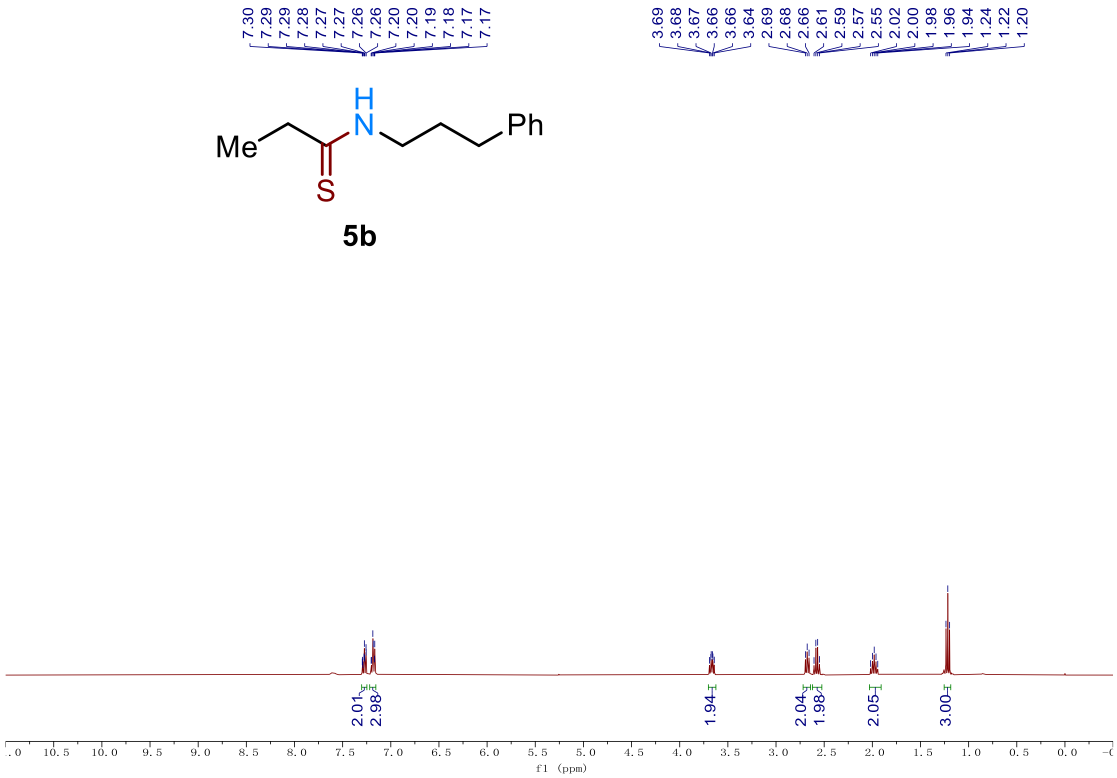

Supplementary Fig. S43 ^{1}H NMR spectrum of compound **5b** (400 MHz, $CDCl_3$)

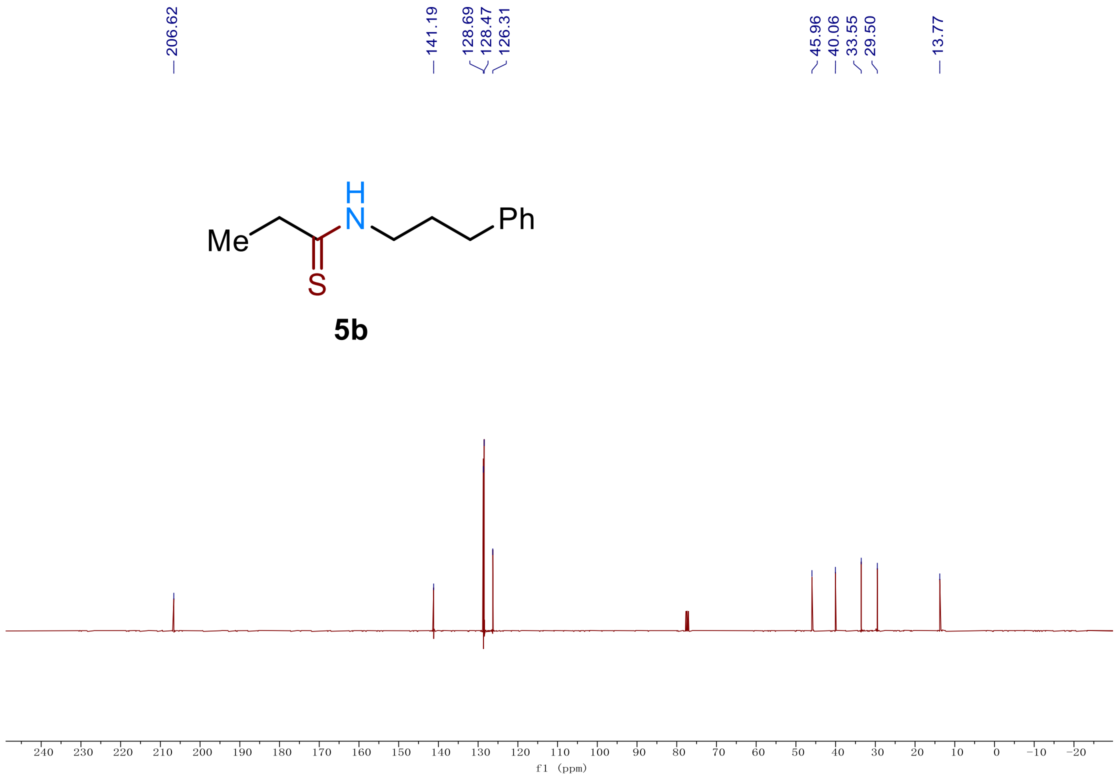

Supplementary Fig. S44 ^{13}C NMR spectrum of compound **5b** (101 MHz, $CDCl_3$)

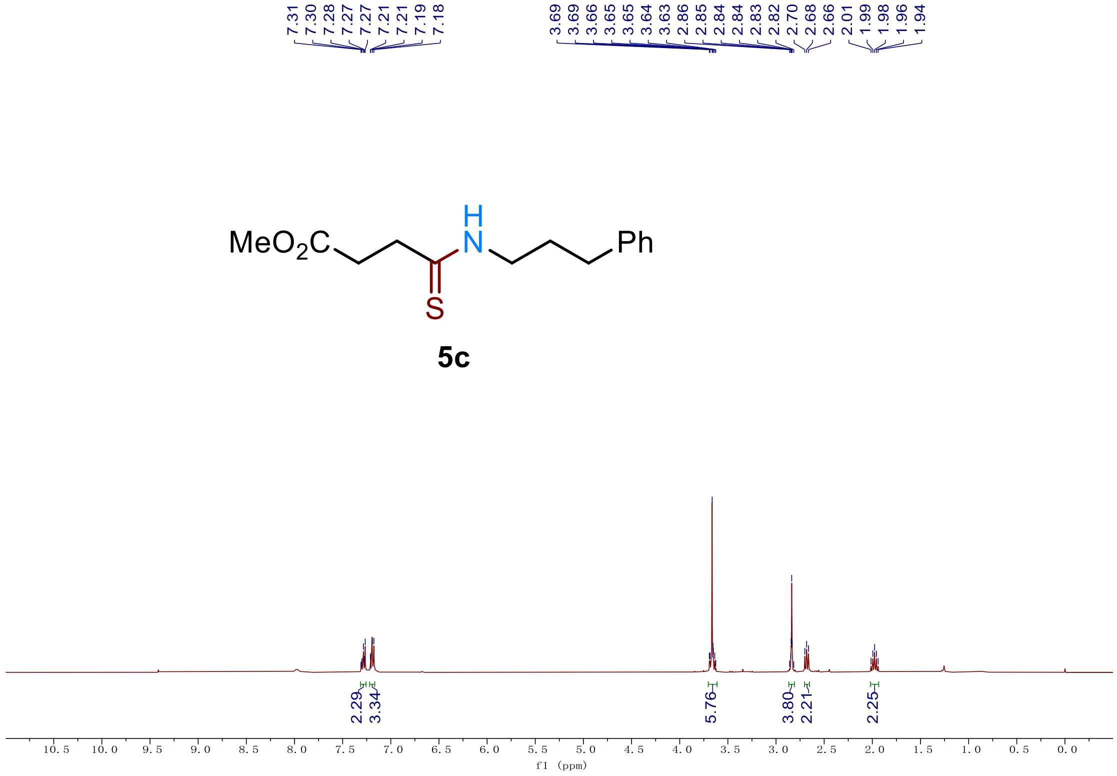

Supplementary Fig. S45 ^{1}H NMR spectrum of compound **5c** (400 MHz, $CDCl_3$)

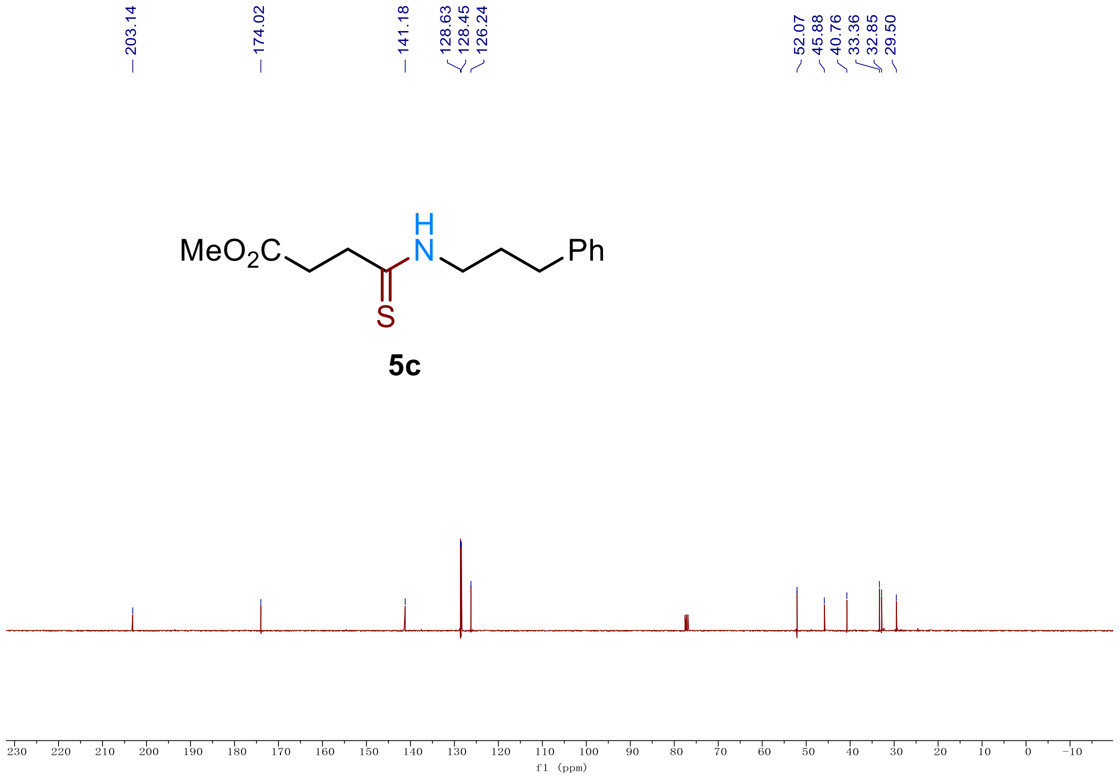

Supplementary Fig. S46 ^{13}C NMR spectrum of compound **5c** (101 MHz, $CDCl_3$)

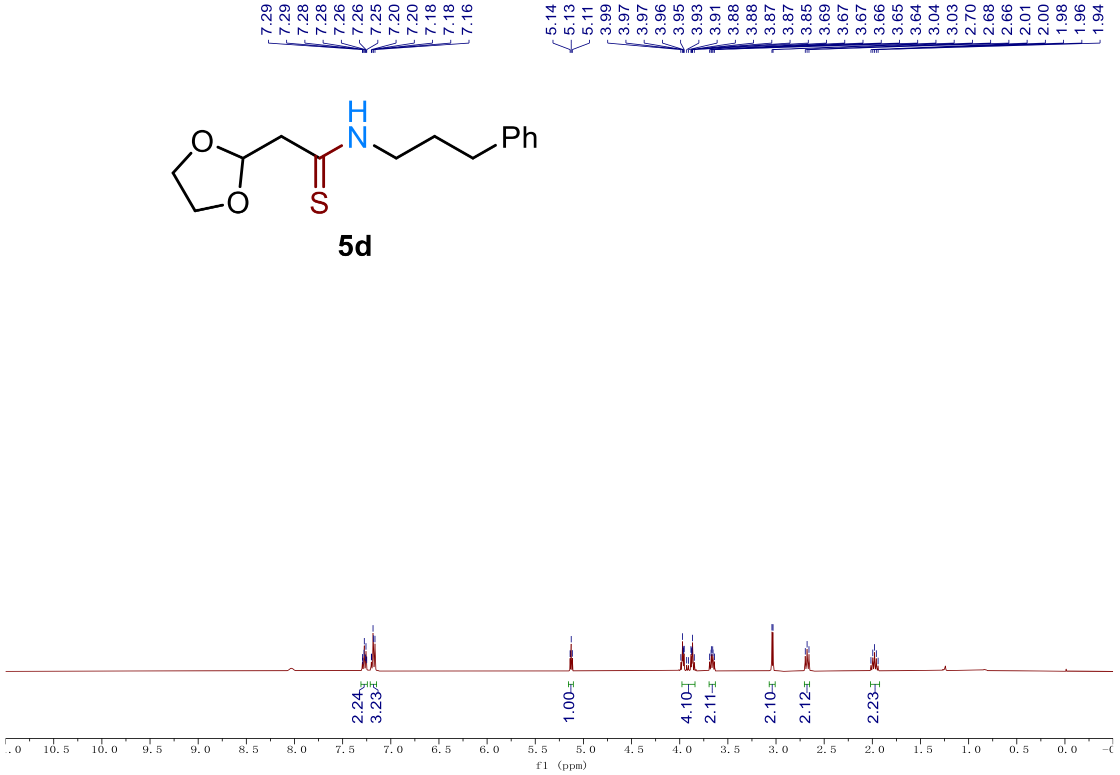

Supplementary Fig. S47 ^{1}H NMR spectrum of compound **5d** (400 MHz, $CDCl_3$)

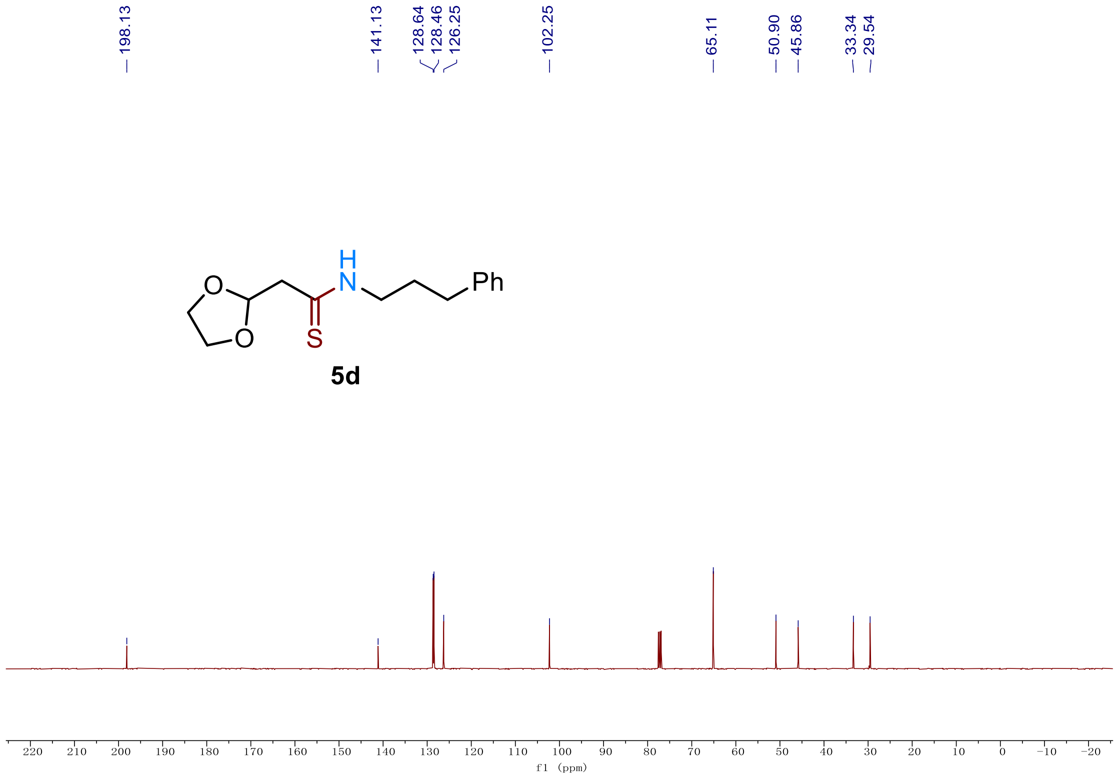

Supplementary Fig. S48 ^{13}C NMR spectrum of compound **5d** (101 MHz, $CDCl_3$)

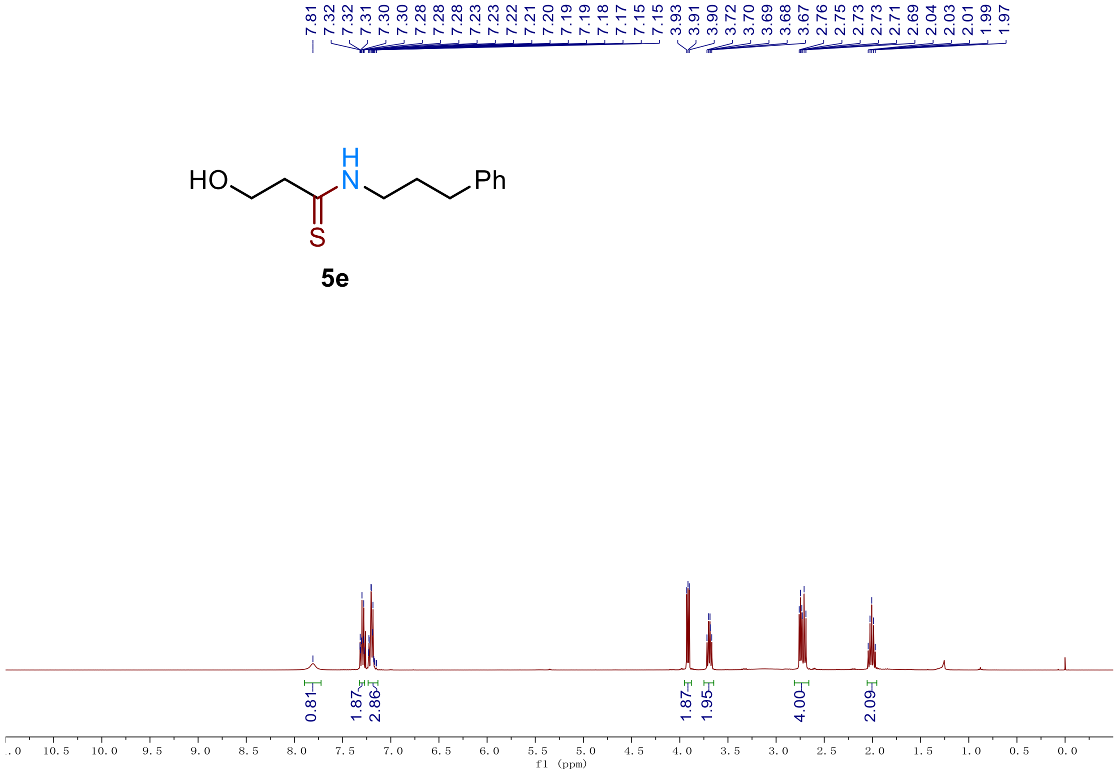

Supplementary Fig. S49 ^{1}H NMR spectrum of compound **5e** (400 MHz, $CDCl_3$)

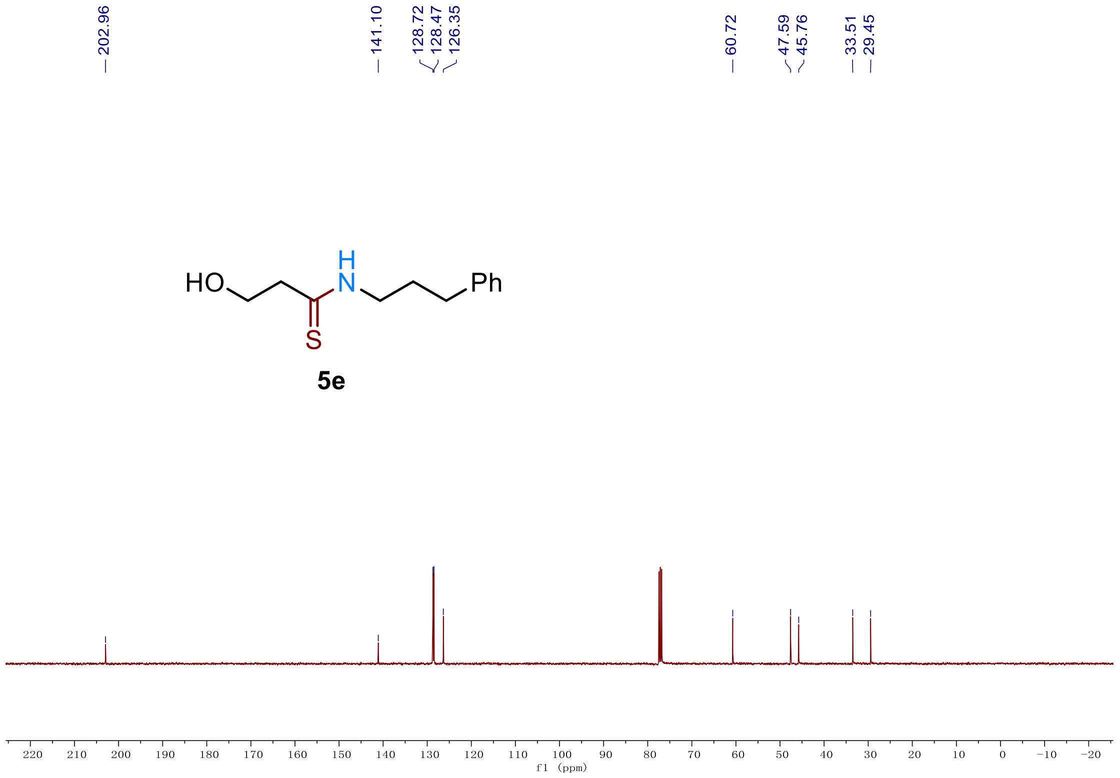

Supplementary Fig. S50 ^{13}C NMR spectrum of compound **5e** (101 MHz, $CDCl_3$)

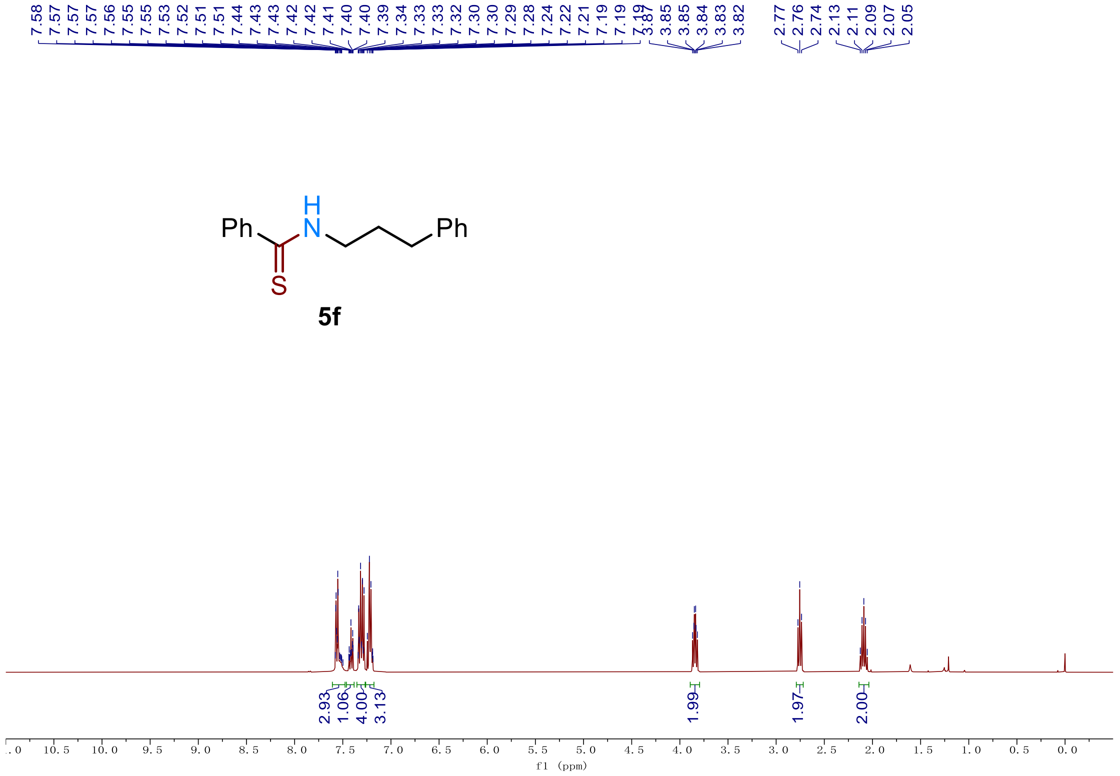

Supplementary Fig. S51 ^{1}H NMR spectrum of compound **5f** (400 MHz, $CDCl_3$)

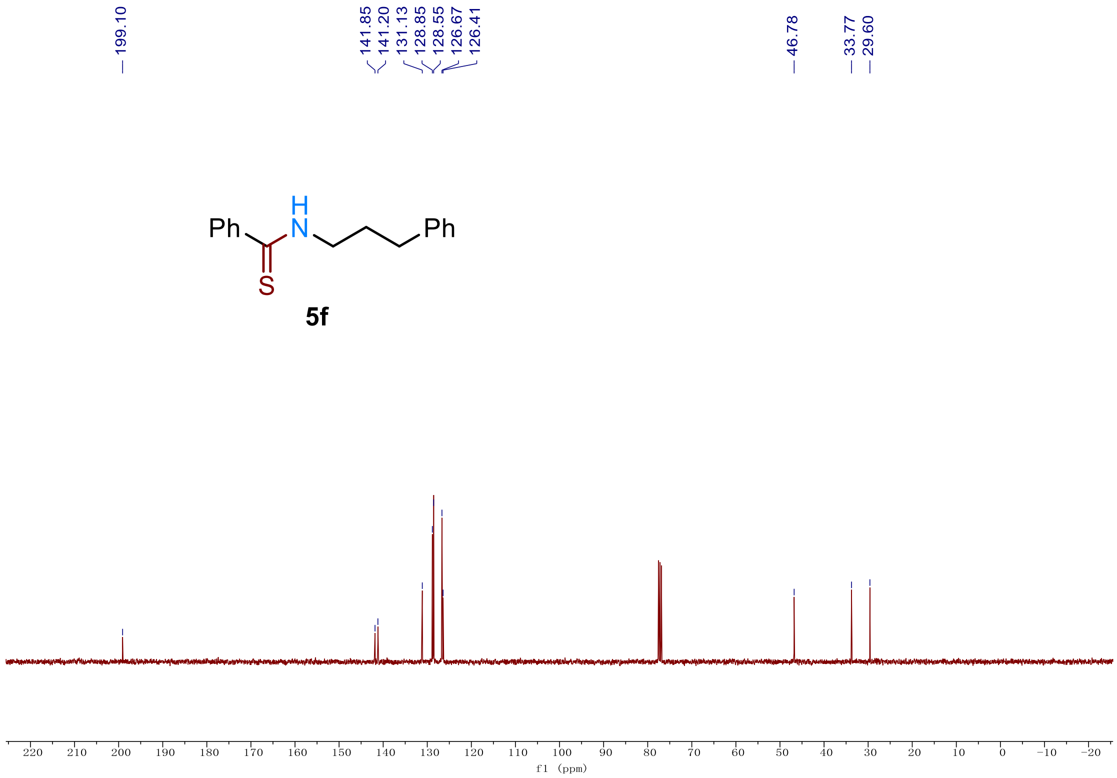

Supplementary Fig. S52 ^{13}C NMR spectrum of compound **5f** (101 MHz, $CDCl_3$)

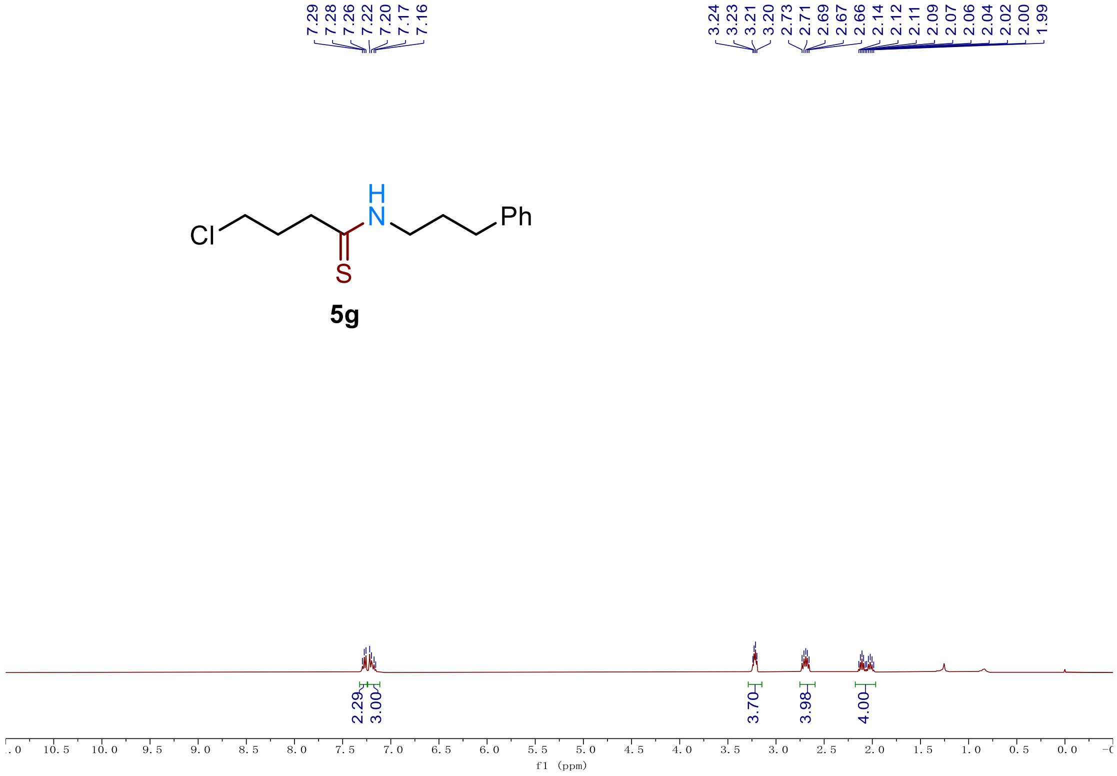

Supplementary Fig. S53 ^{1}H NMR spectrum of compound **5g** (400 MHz, $CDCl_3$)

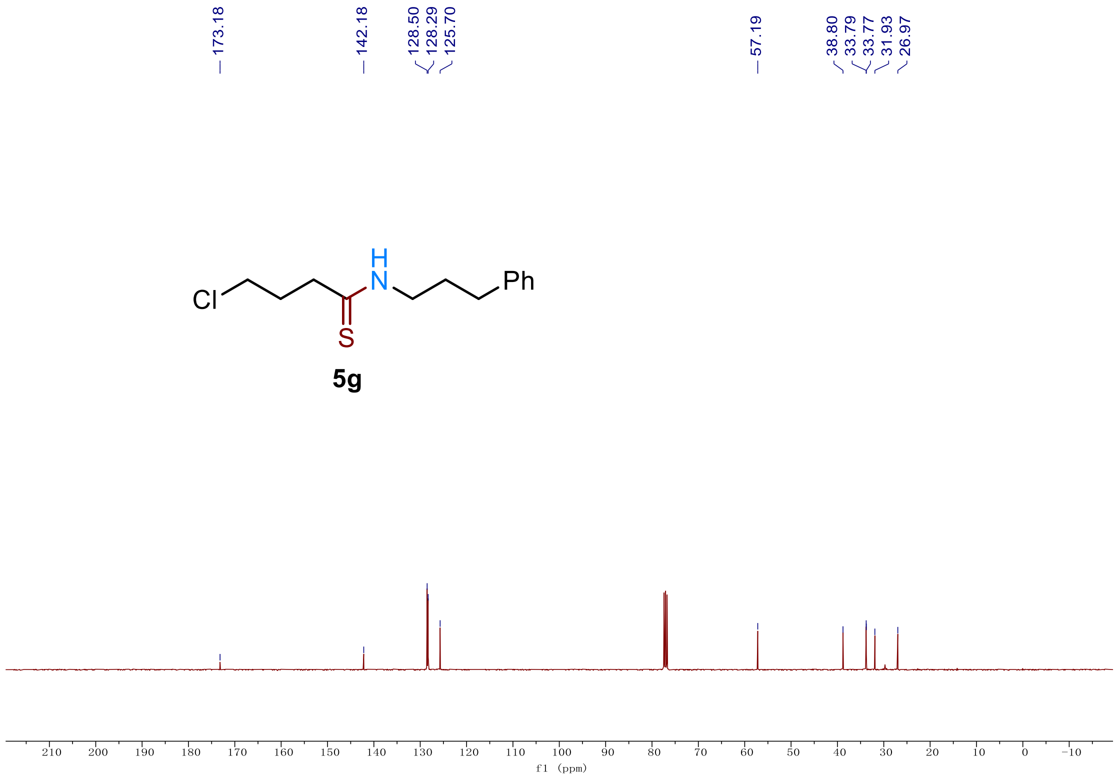

Supplementary Fig. S54 ^{13}C NMR spectrum of compound **5g** (101 MHz, $CDCl_3$)

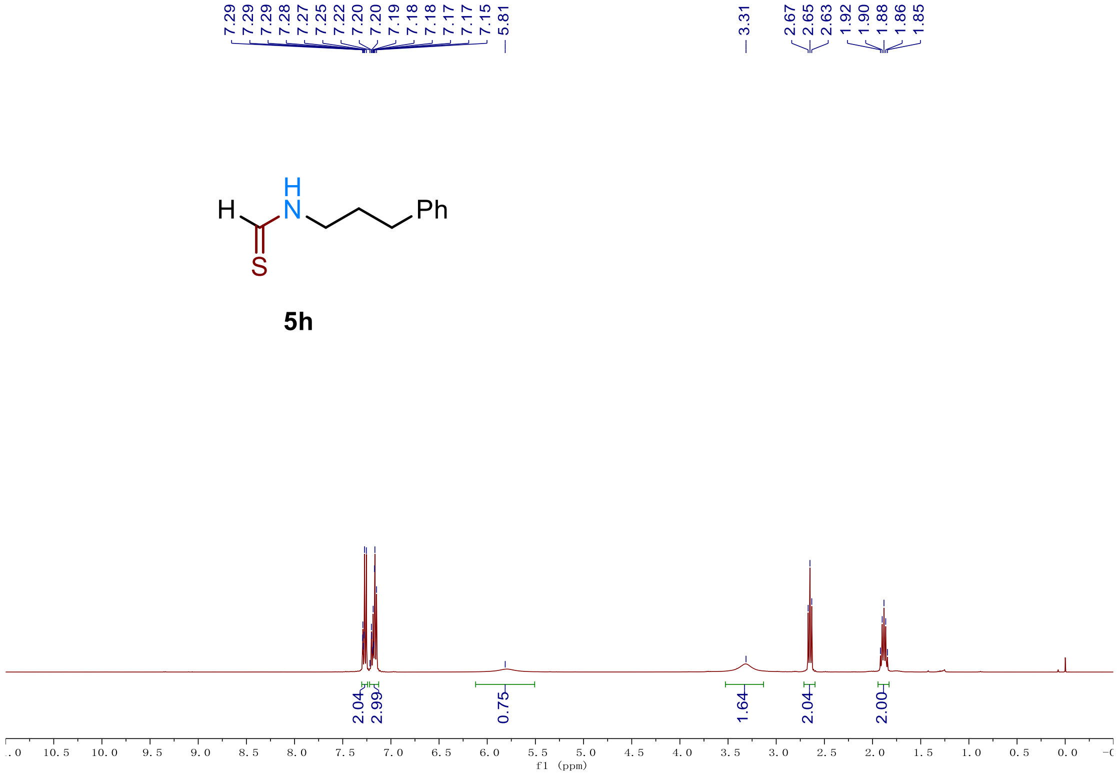

Supplementary Fig. S55 ^{1}H NMR spectrum of compound **5h** (400 MHz, $CDCl_3$)

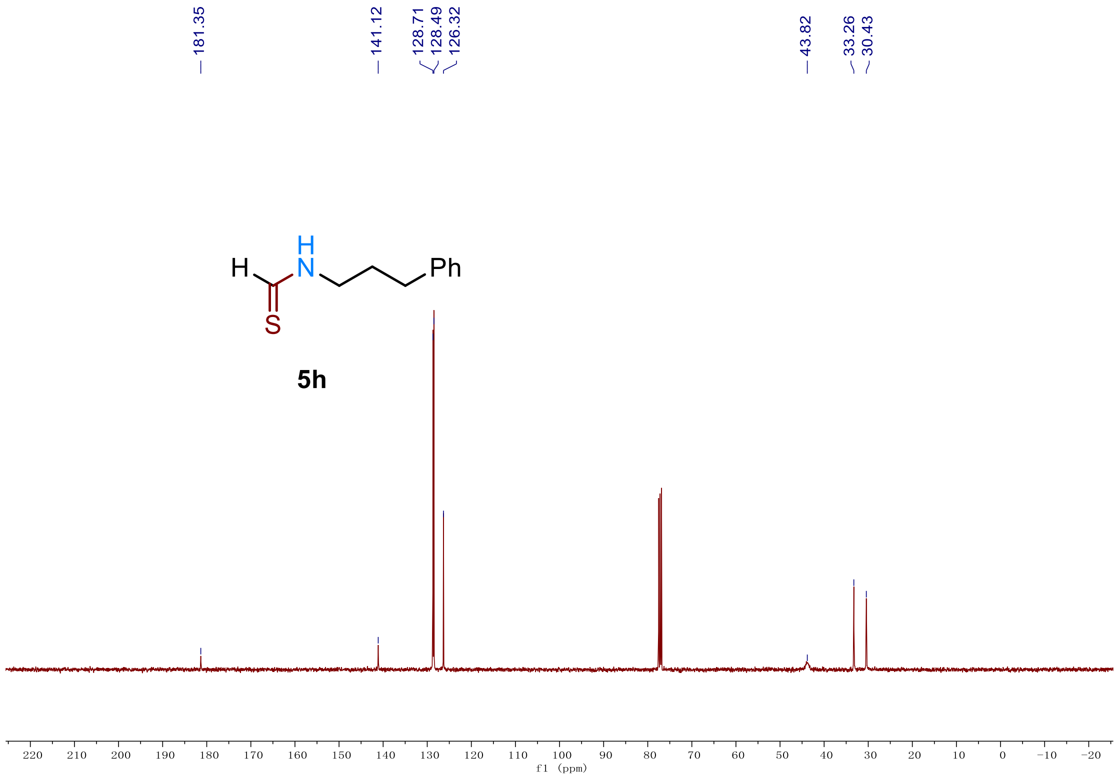

Supplementary Fig. S56 ^{13}C NMR spectrum of compound **5h** (101 MHz, $CDCl_3$)

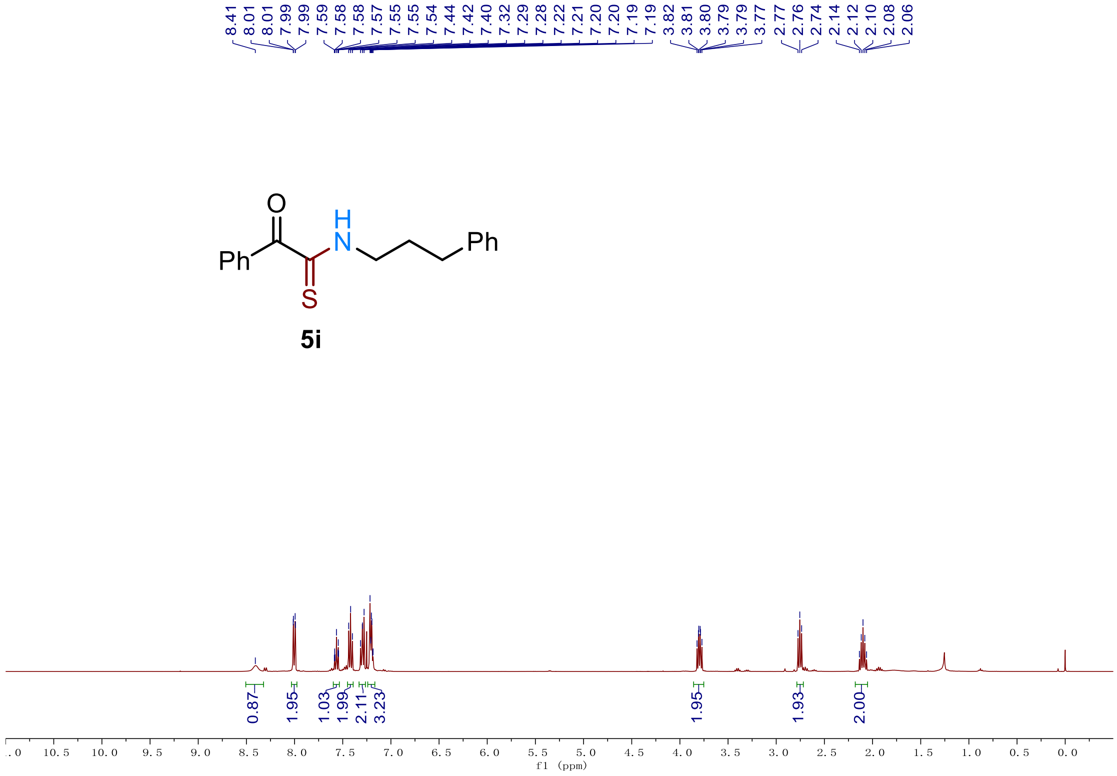

Supplementary Fig. S57 ^{1}H NMR spectrum of compound **5i** (400 MHz, $CDCl_3$)

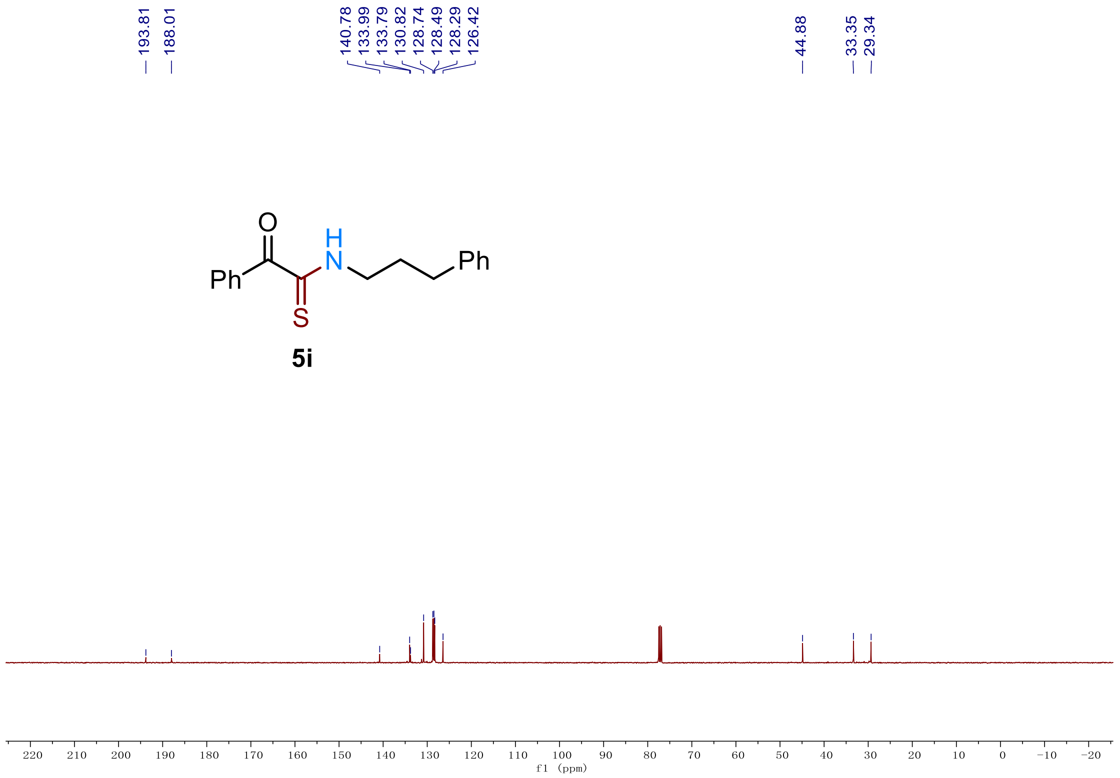

Supplementary Fig. S58 ^{13}C NMR spectrum of compound **5i** (101 MHz, $CDCl_3$)

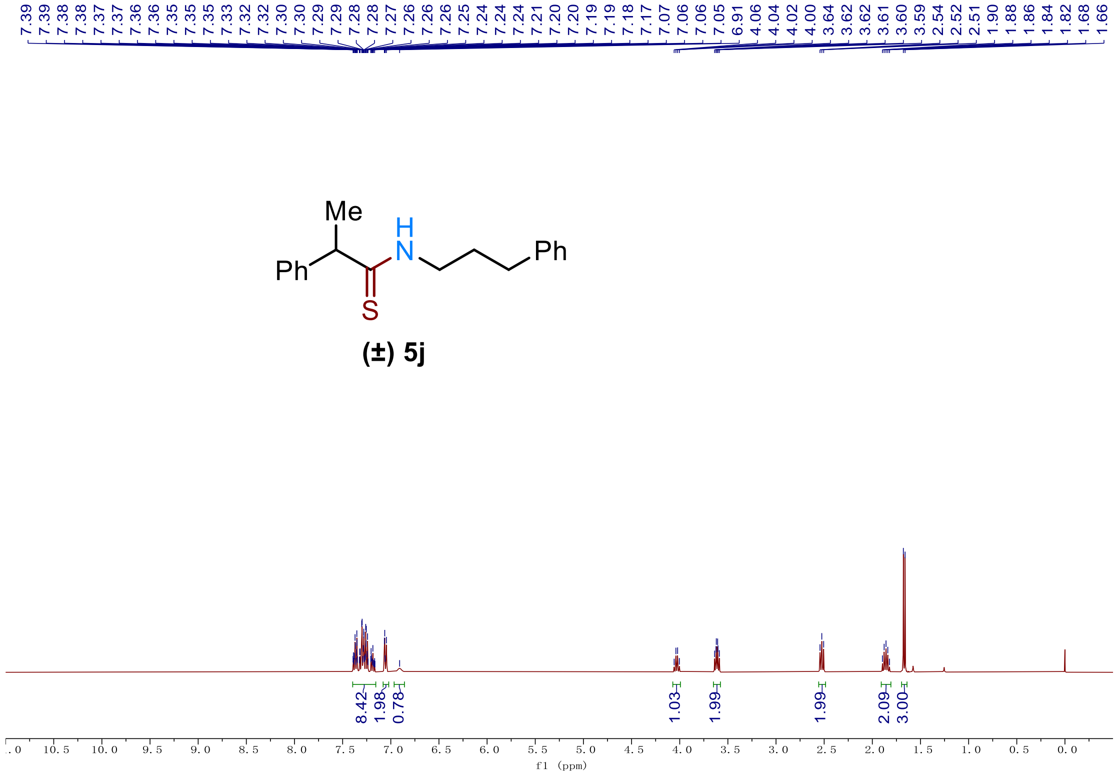

Supplementary Fig. S59 ^{1}H NMR spectrum of compound **(±) 5j** (400 MHz, $CDCl_3$)

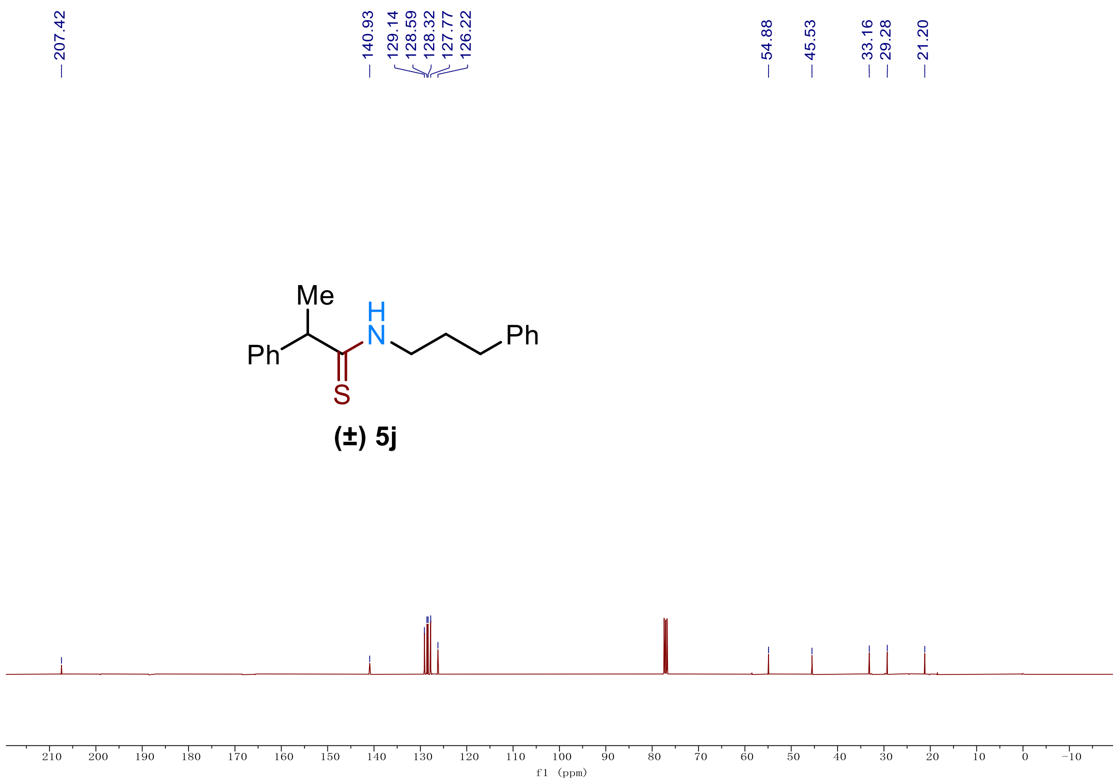

Supplementary Fig. S60 ^{13}C NMR spectrum of compound **(±) 5j** (101 MHz, $CDCl_3$)

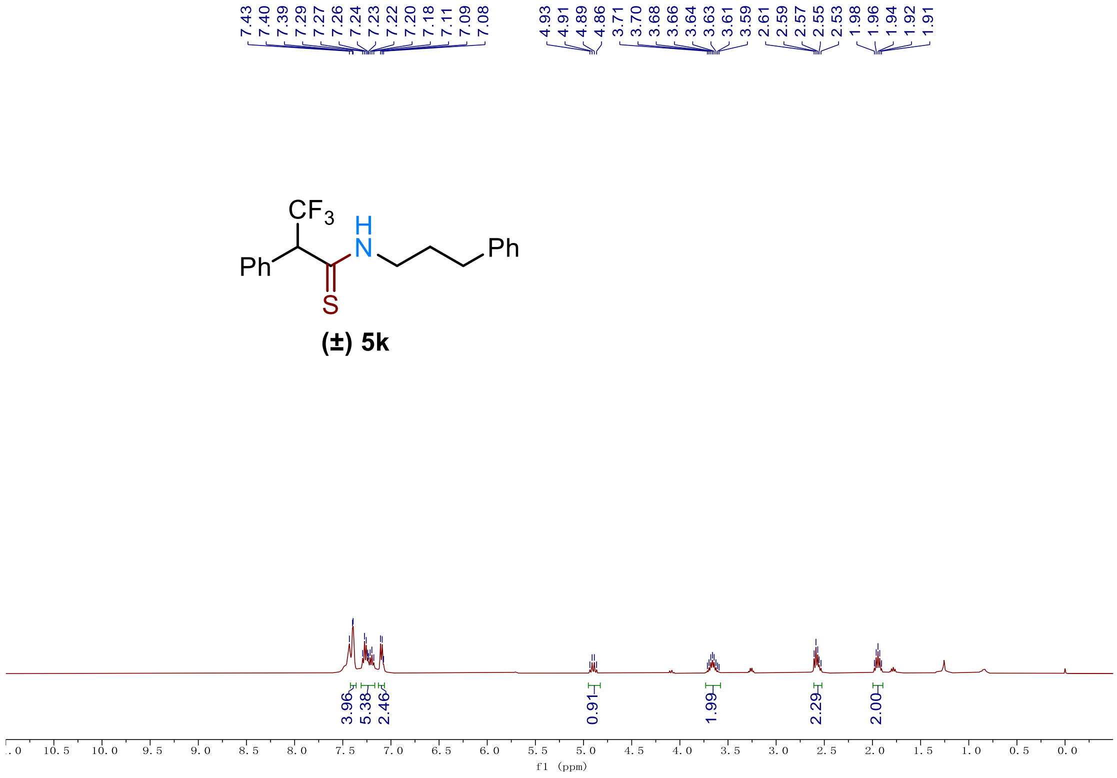

Supplementary Fig. S61 ^{1}H NMR spectrum of compound **(±) 5k** (400 MHz, $CDCl_3$)

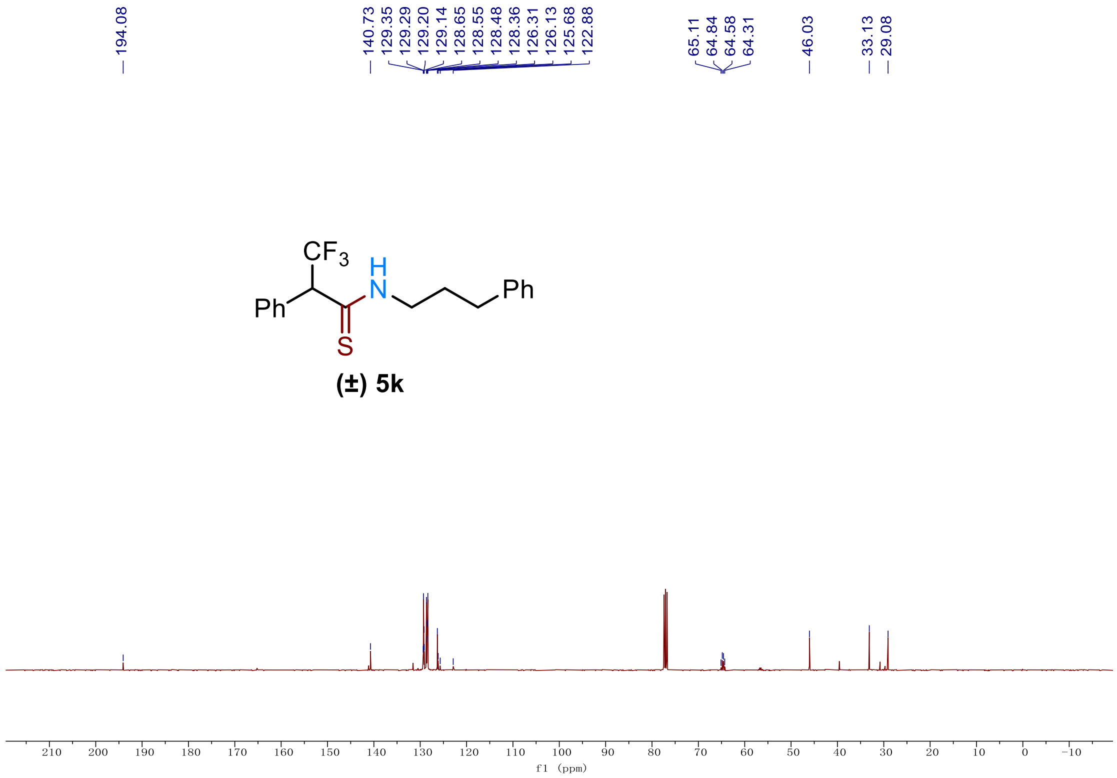

Supplementary Fig. S62 ^{13}C NMR spectrum of compound **(±) 5k** (101 MHz, $CDCl_3$)

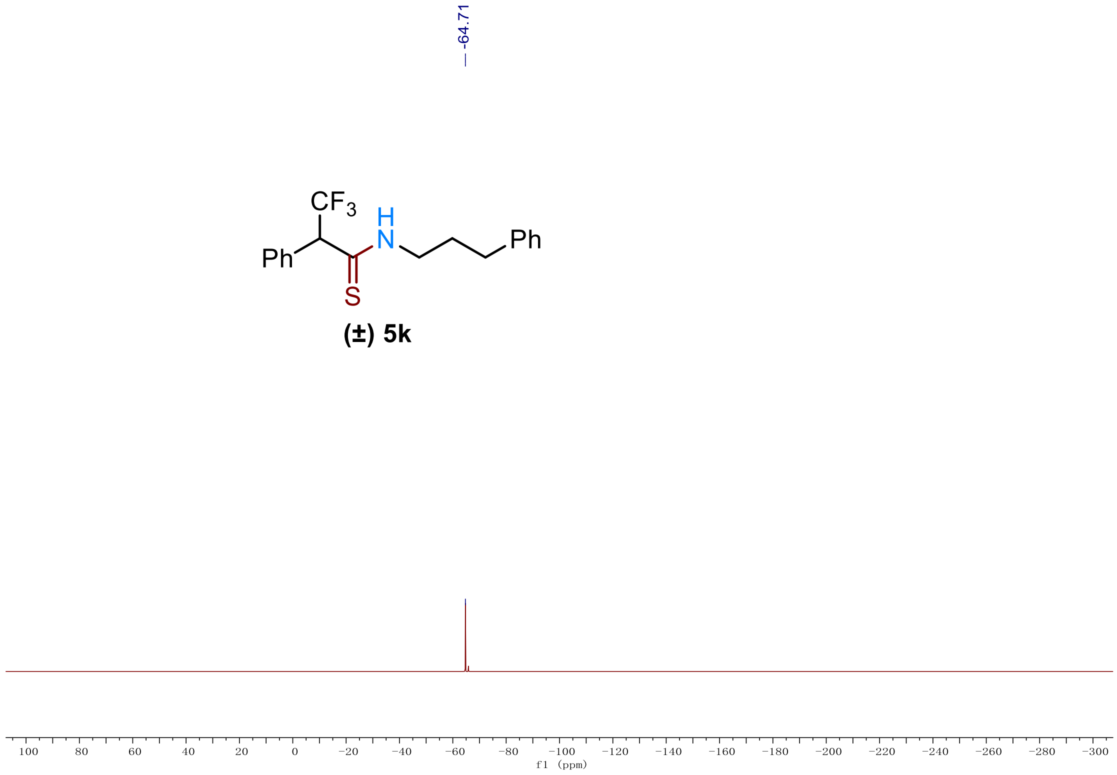

Supplementary Fig. S63 ^{19}F NMR spectrum of compound **(±) 5k** (377 MHz, $CDCl_3$)

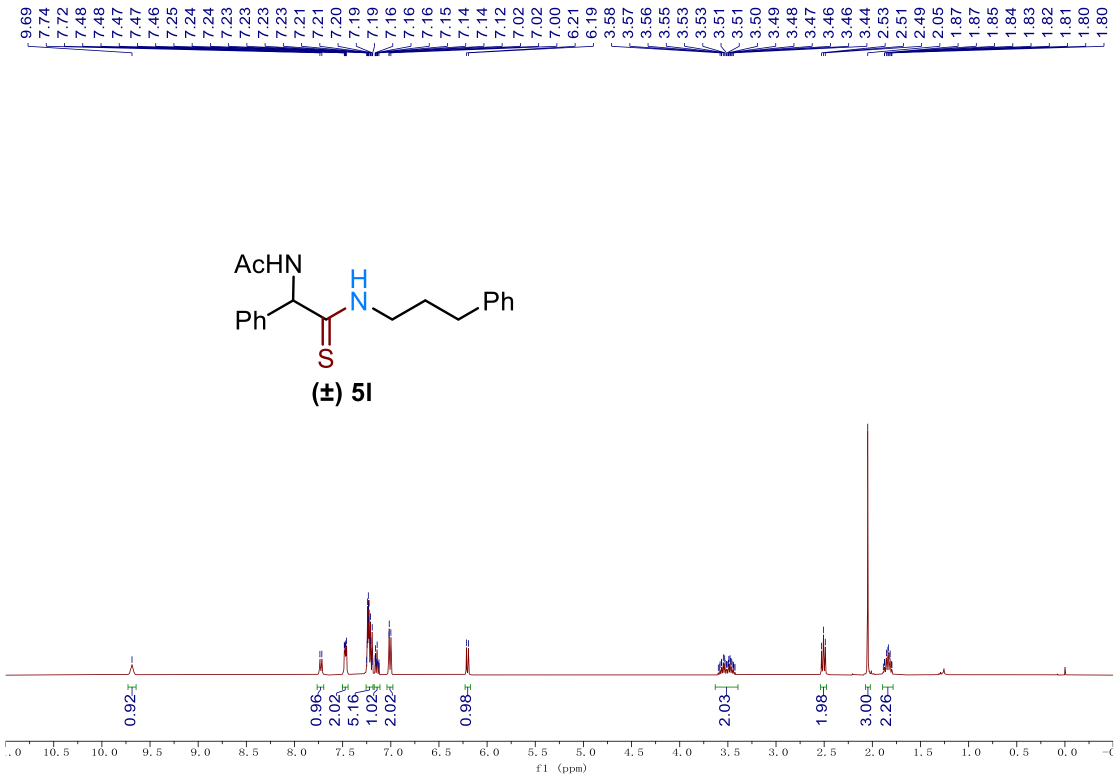

Supplementary Fig. S64 ^{1}H NMR spectrum of compound **(±) 5l** (400 MHz, $CDCl_3$)

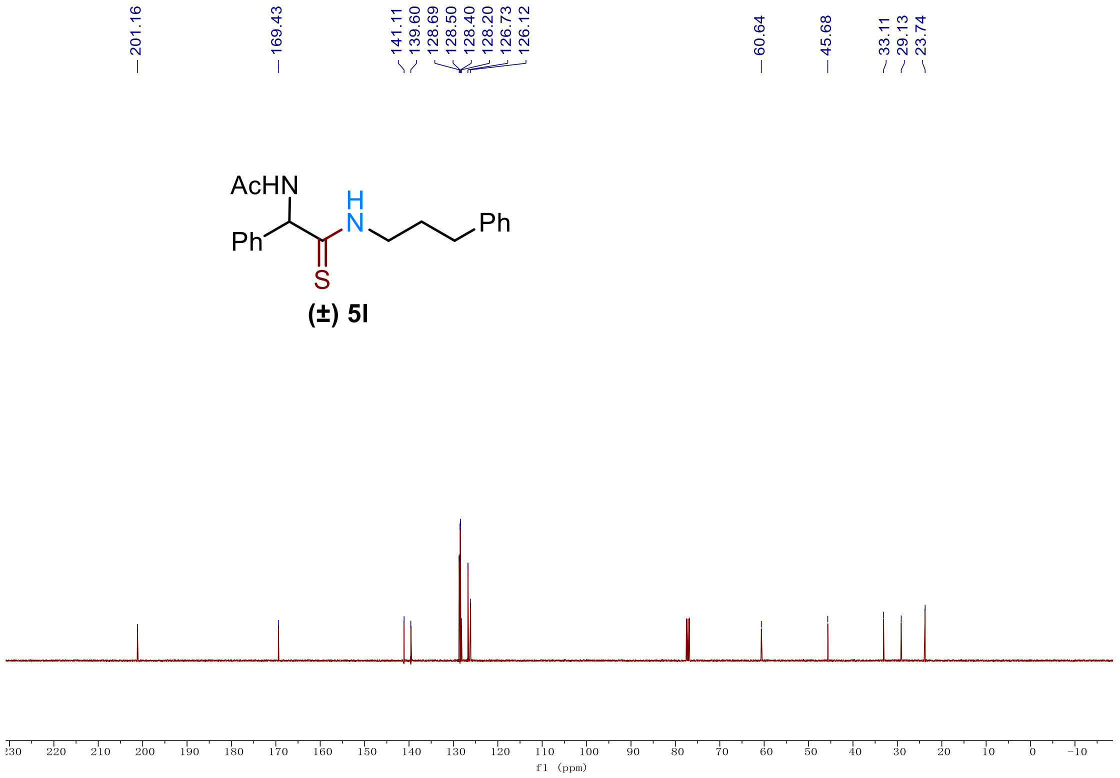

Supplementary Fig. S65 ^{13}C NMR spectrum of compound **(±) 5l** (101 MHz, $CDCl_3$)

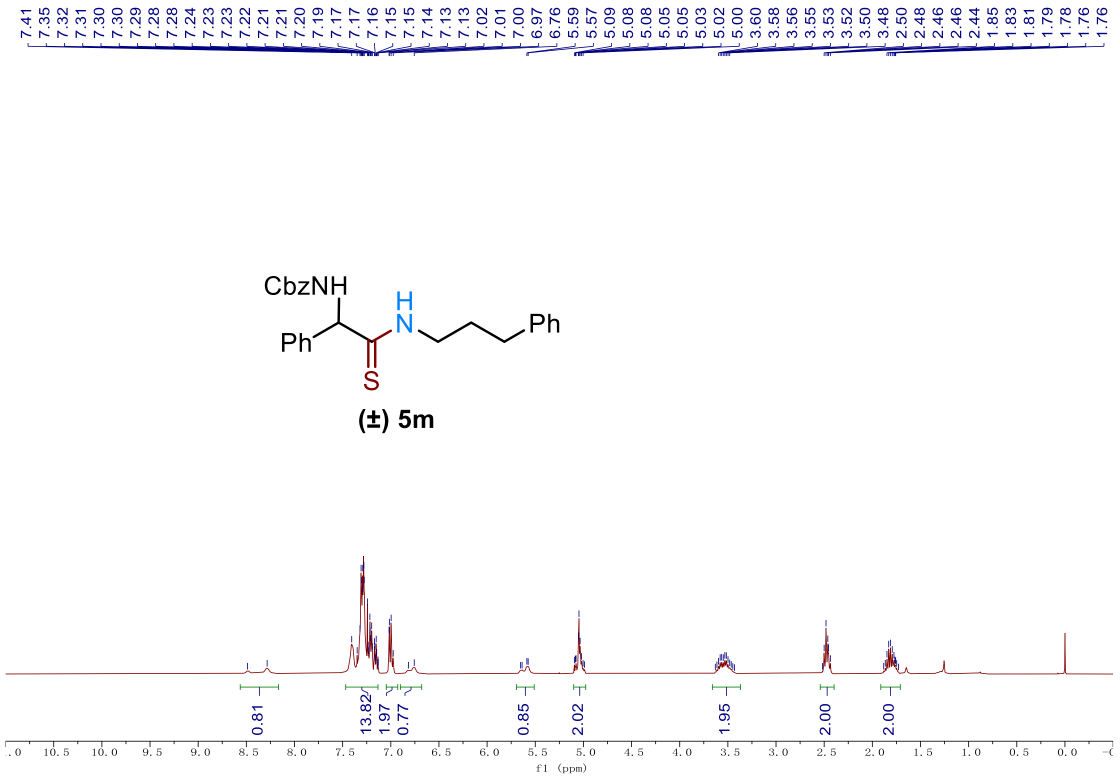

Supplementary Fig. S66 ^{1}H NMR spectrum of compound **(±) 5m** (400 MHz, $CDCl_3$)

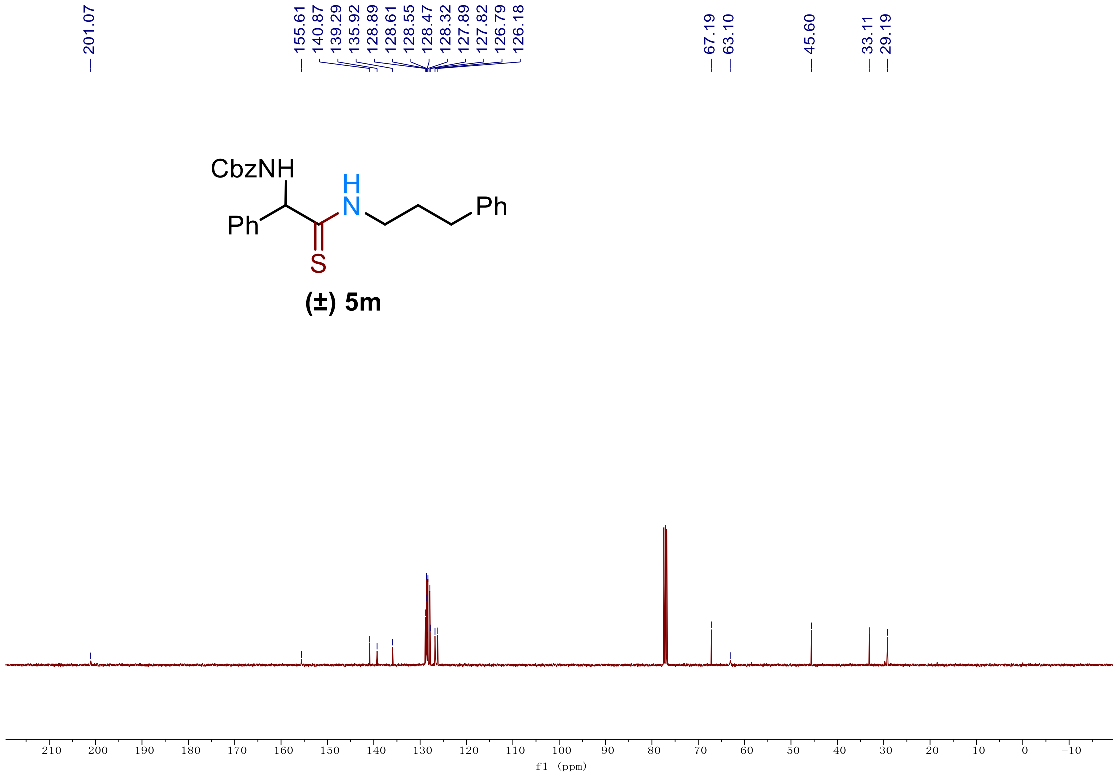

Supplementary Fig. S67 ^{13}C NMR spectrum of compound **(±) 5m** (101 MHz, $CDCl_3$)

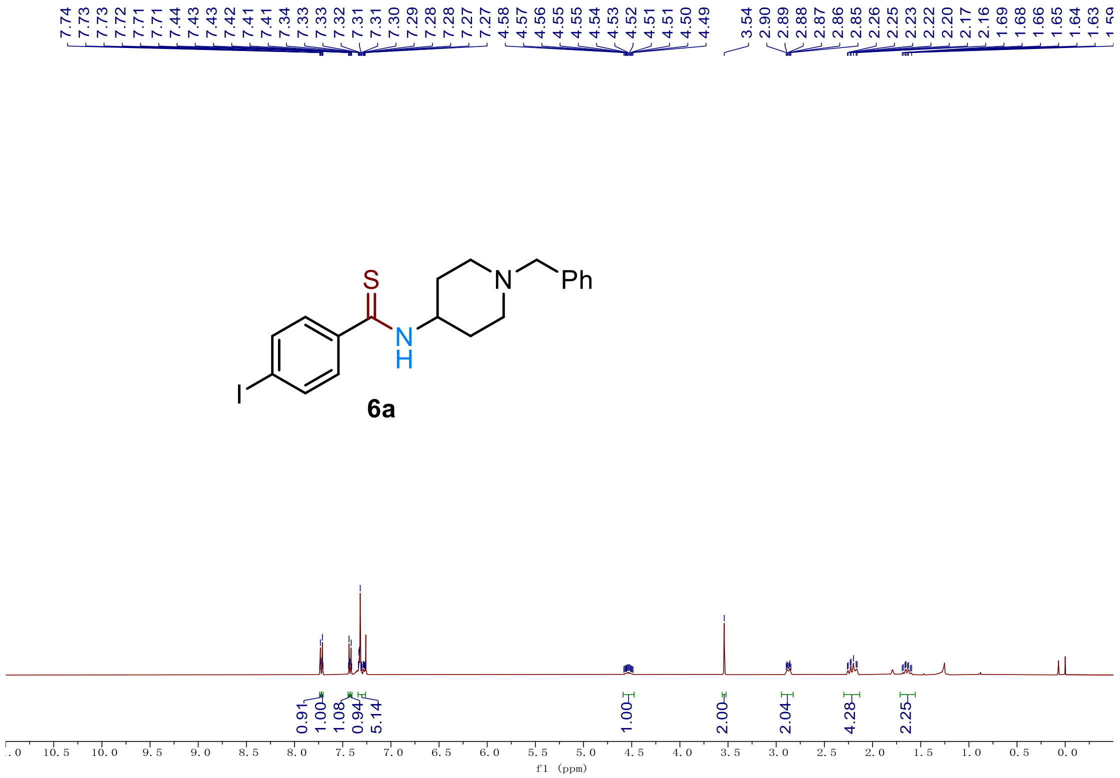

Supplementary Fig. S68 ^{1}H NMR spectrum of compound **6a** (400 MHz, $CDCl_3$)

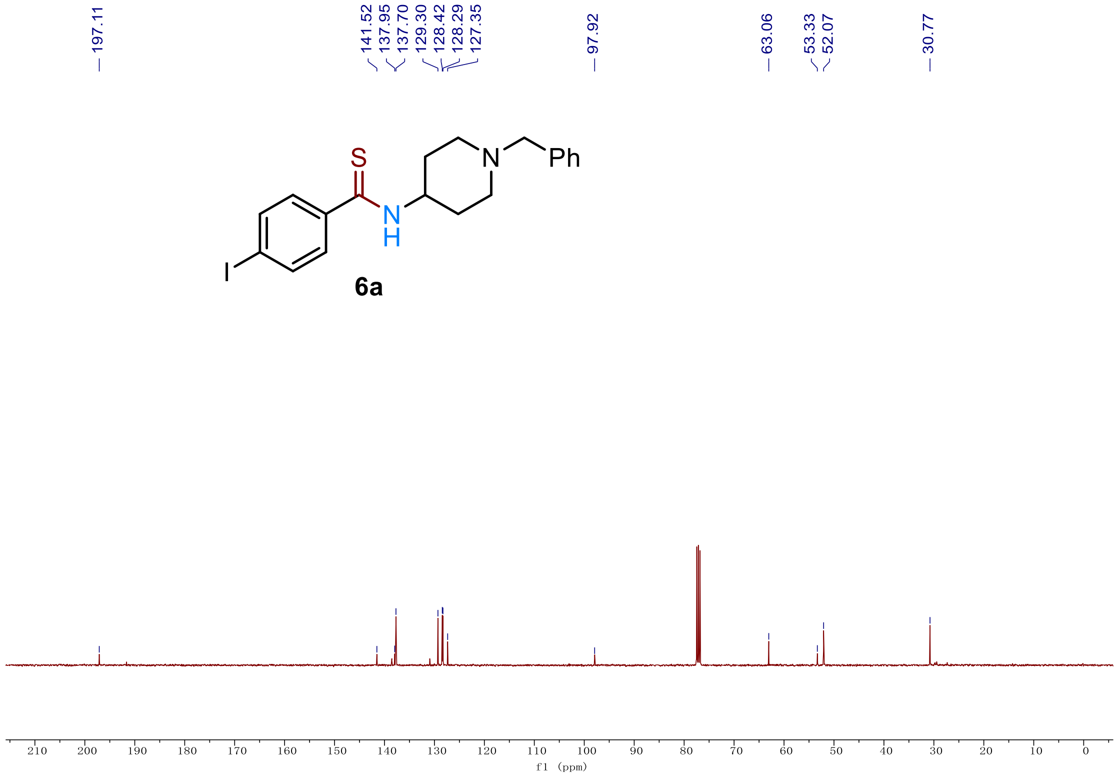

Supplementary Fig. S69 ^{13}C NMR spectrum of compound **6a** (101 MHz, $CDCl_3$)

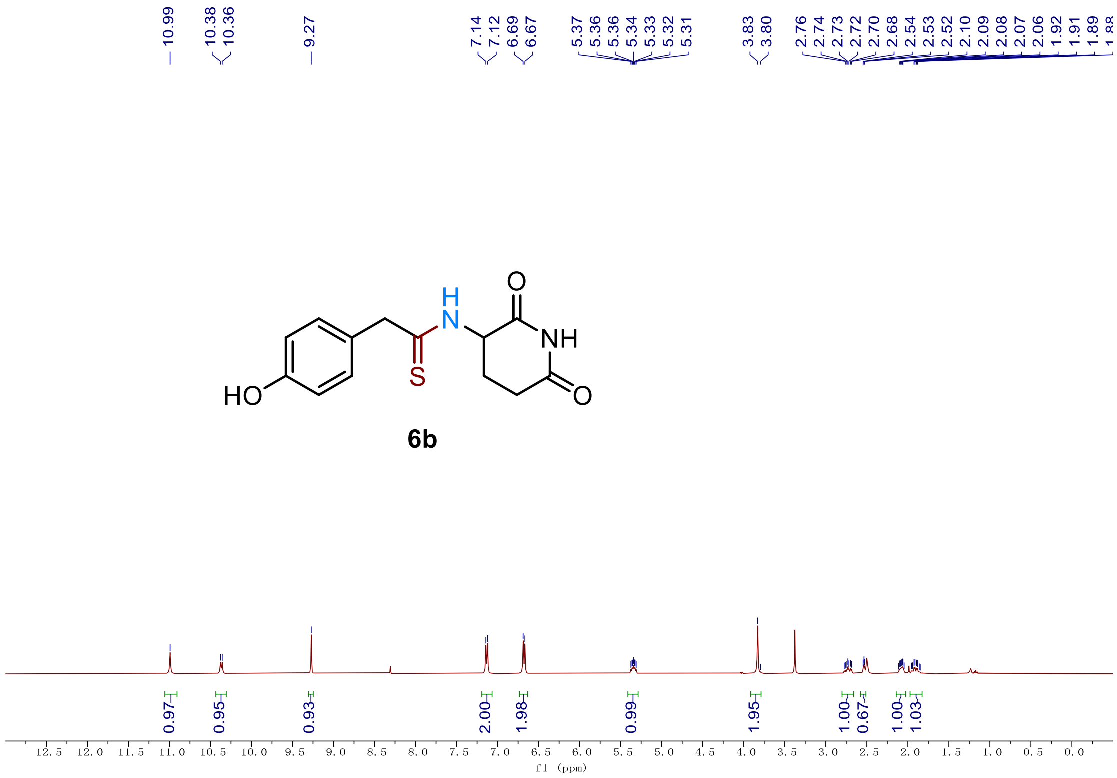

Supplementary Fig. S70 ^{1}H NMR spectrum of compound **6b** (400 MHz, DMSO-d_6)

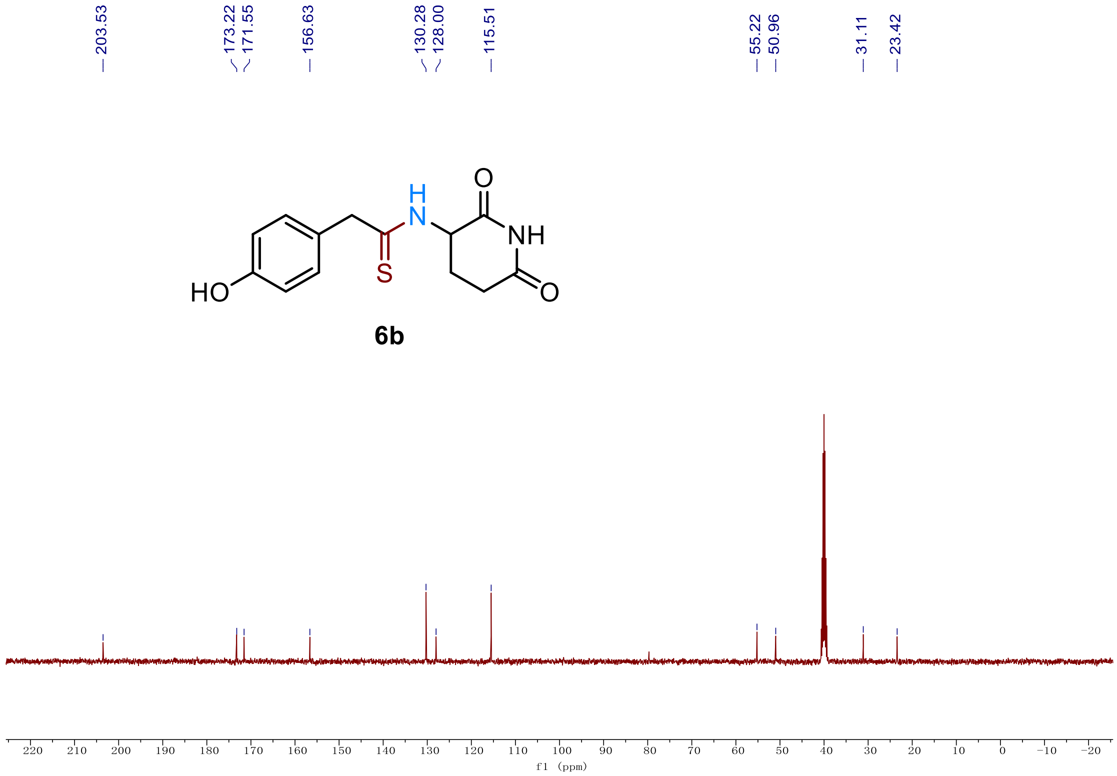

Supplementary Fig. S71 ^{13}C NMR spectrum of compound **6b** (101 MHz, DMSO-d_6)

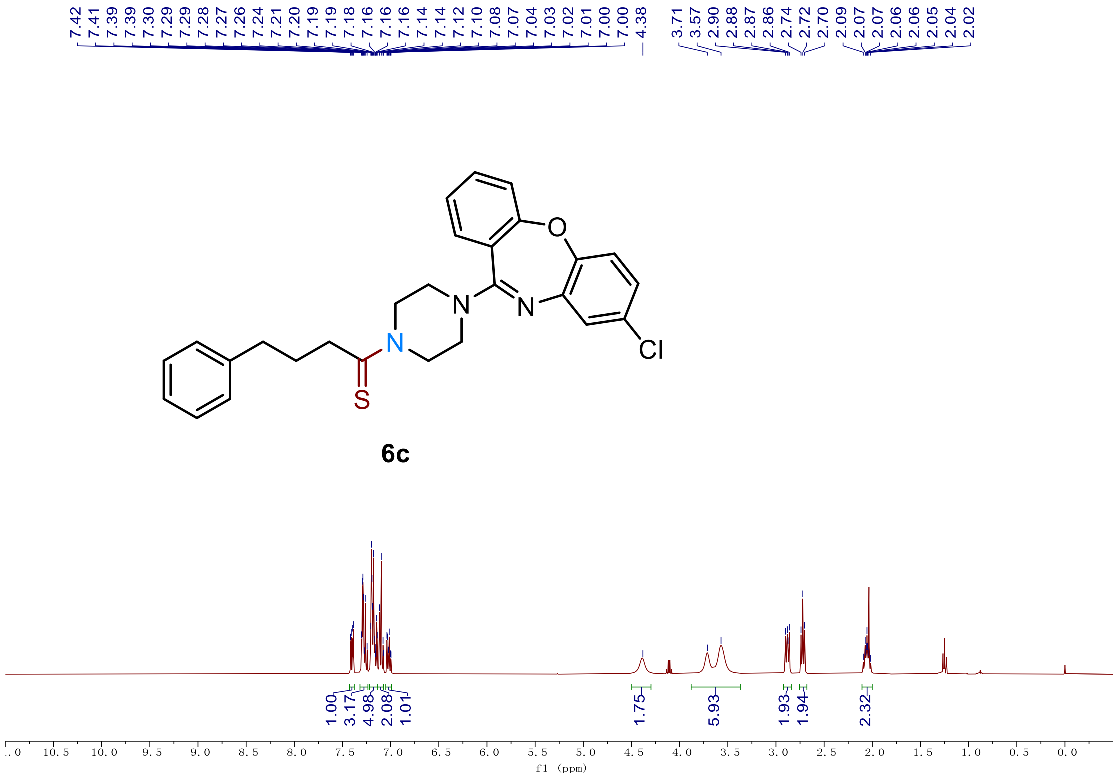

Supplementary Fig. S72 ^{1}H NMR spectrum of compound **6c** (400 MHz, $CDCl_3$)

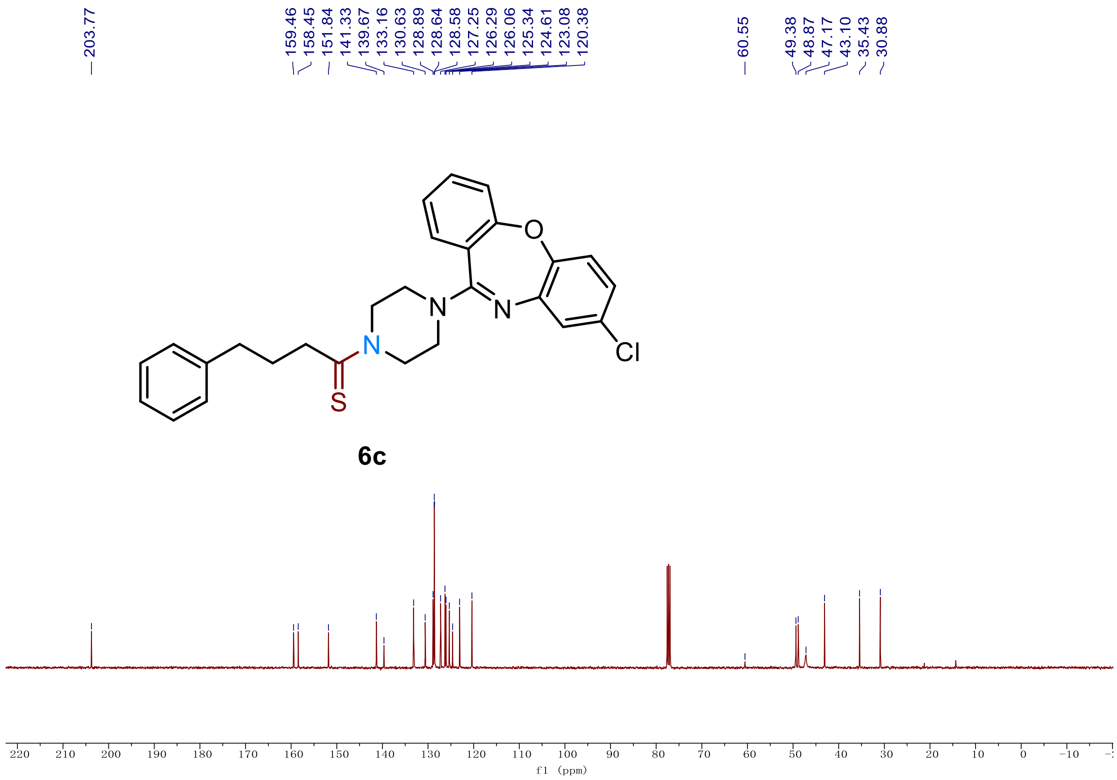

Supplementary Fig. S73 ^{13}C NMR spectrum of compound **6c** (101 MHz, $CDCl_3$)

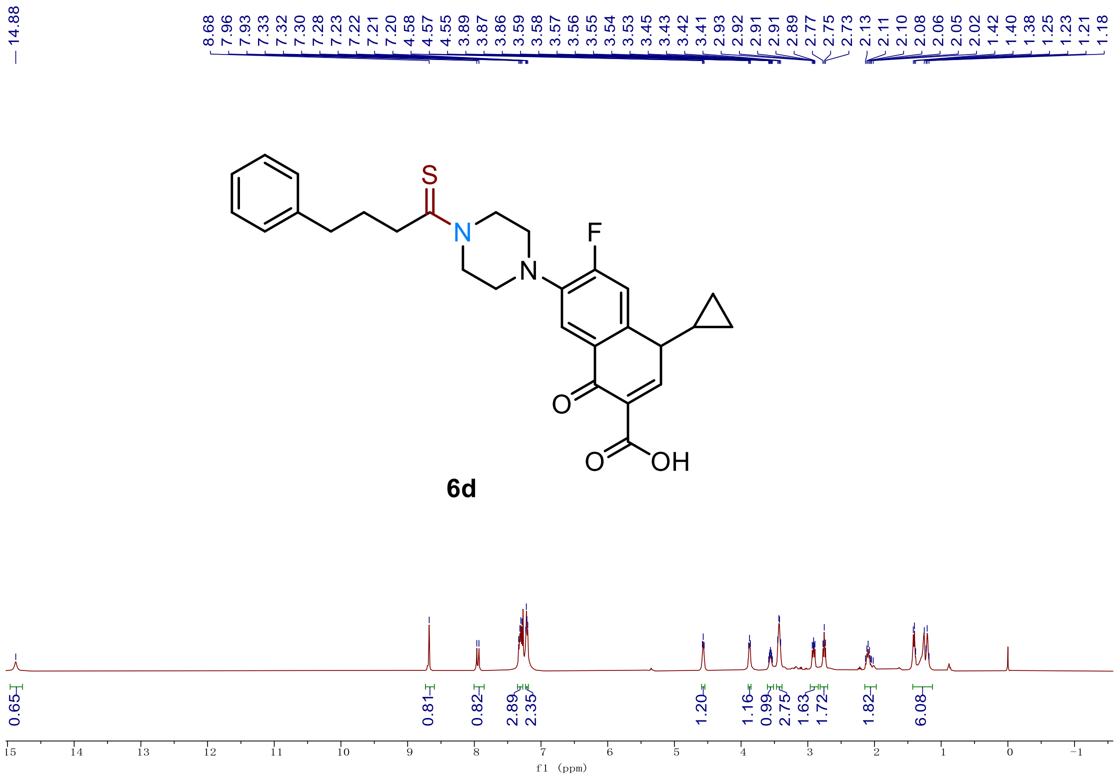

Supplementary Fig. S74 ^{1}H NMR spectrum of compound **6d** (400 MHz, $CDCl_3$)

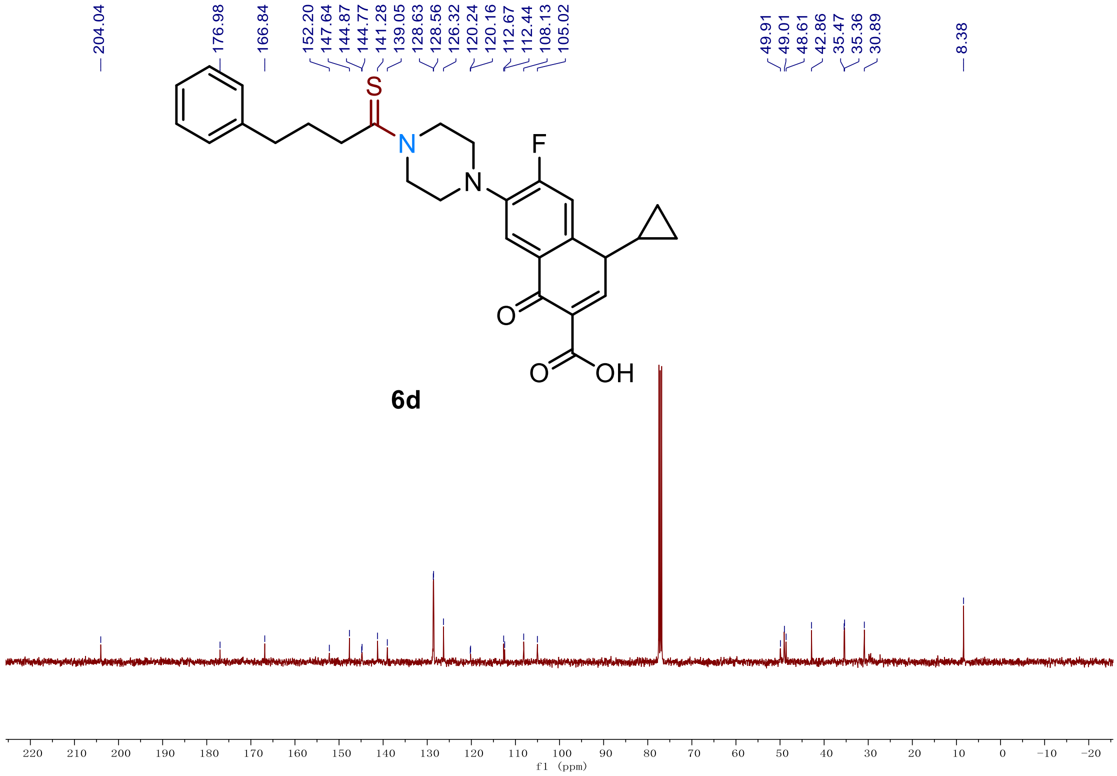

Supplementary Fig. S75 ^{13}C NMR spectrum of compound **6d** (101 MHz, $CDCl_3$)

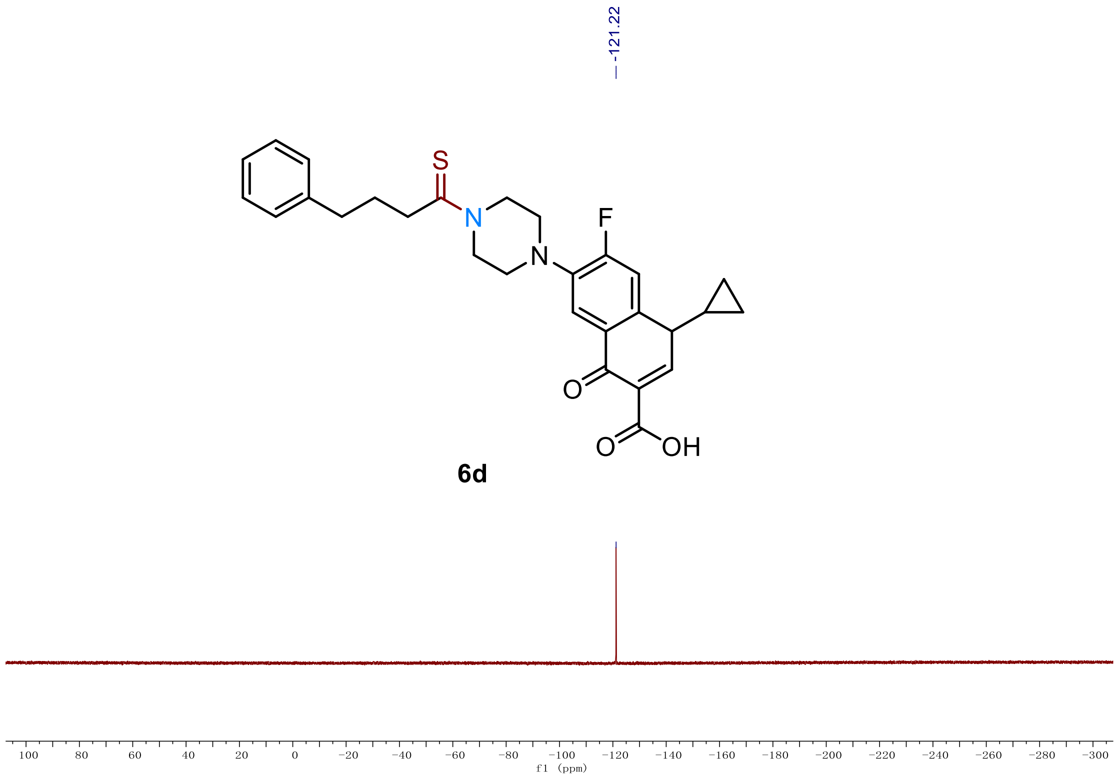

Supplementary Fig. S76 ^{19}F NMR spectrum of compound **6d** (377 MHz, $CDCl_3$)

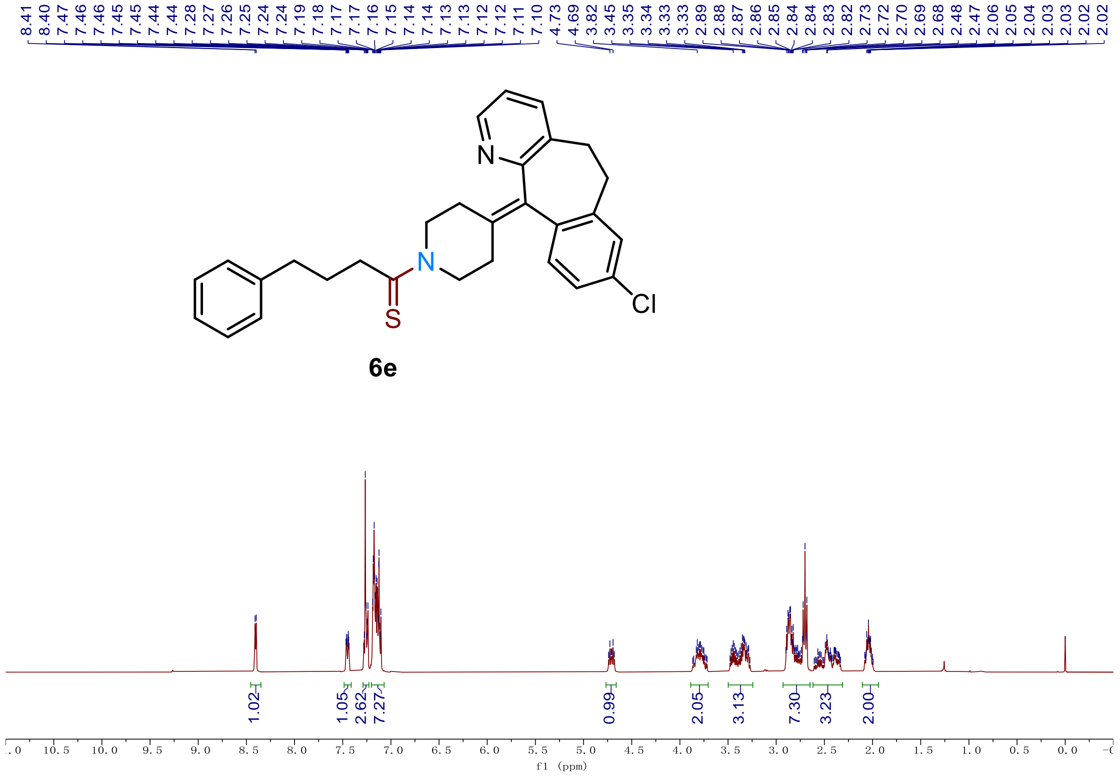

Supplementary Fig. S77 ^{1}H NMR spectrum of compound **6e** (400 MHz, $CDCl_3$)

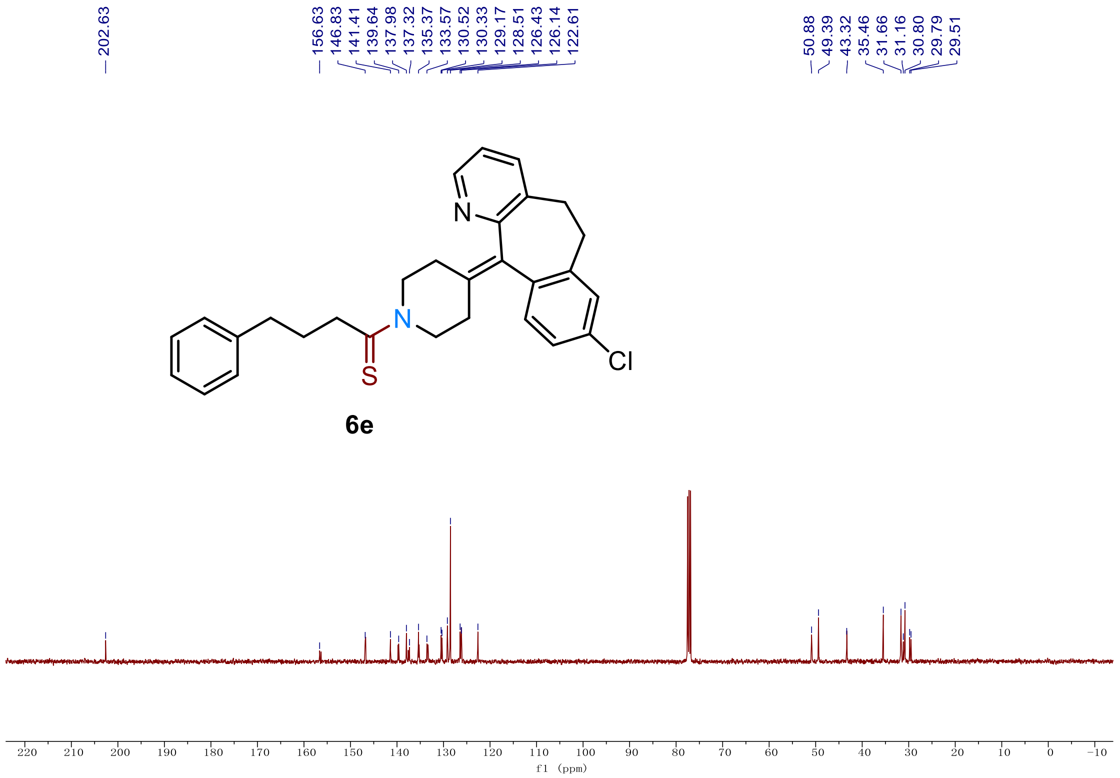

Supplementary Fig. S78 ^{13}C NMR spectrum of compound **6e** (101 MHz, $CDCl_3$)

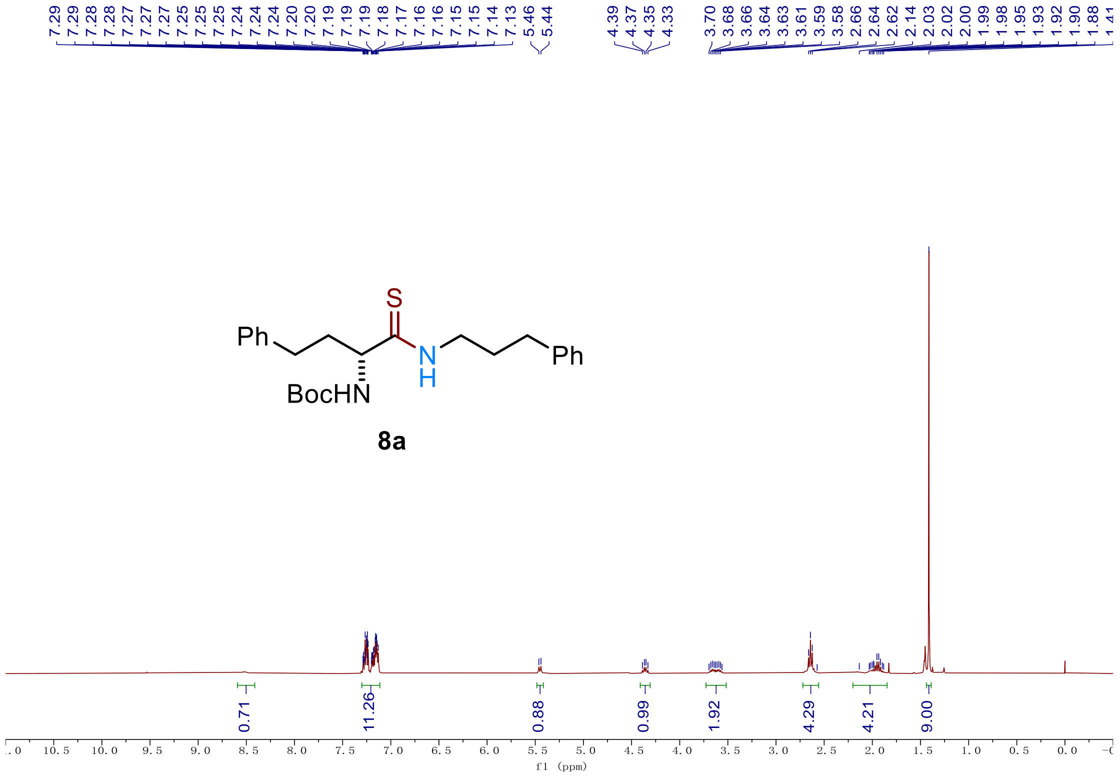

Supplementary Fig. S79 ^{1}H NMR spectrum of compound **8a** (400 MHz, $CDCl_3$)

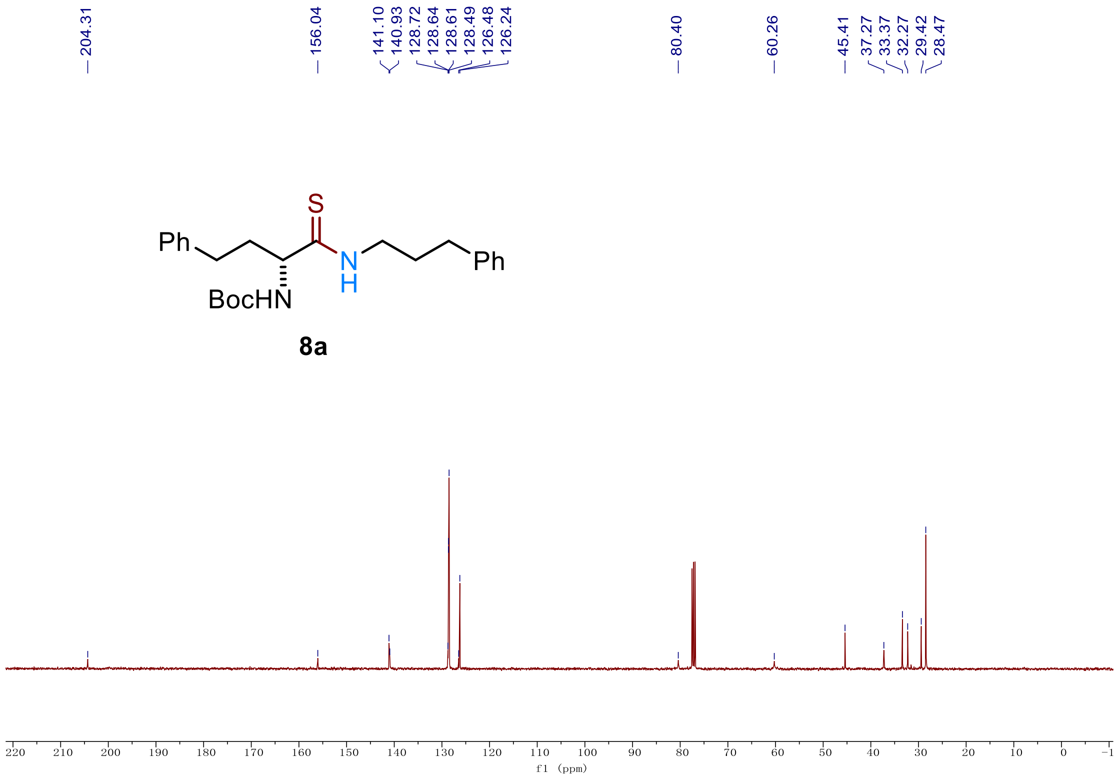

Supplementary Fig. S80 ^{13}C NMR spectrum of compound **8a** (101 MHz, $CDCl_3$)

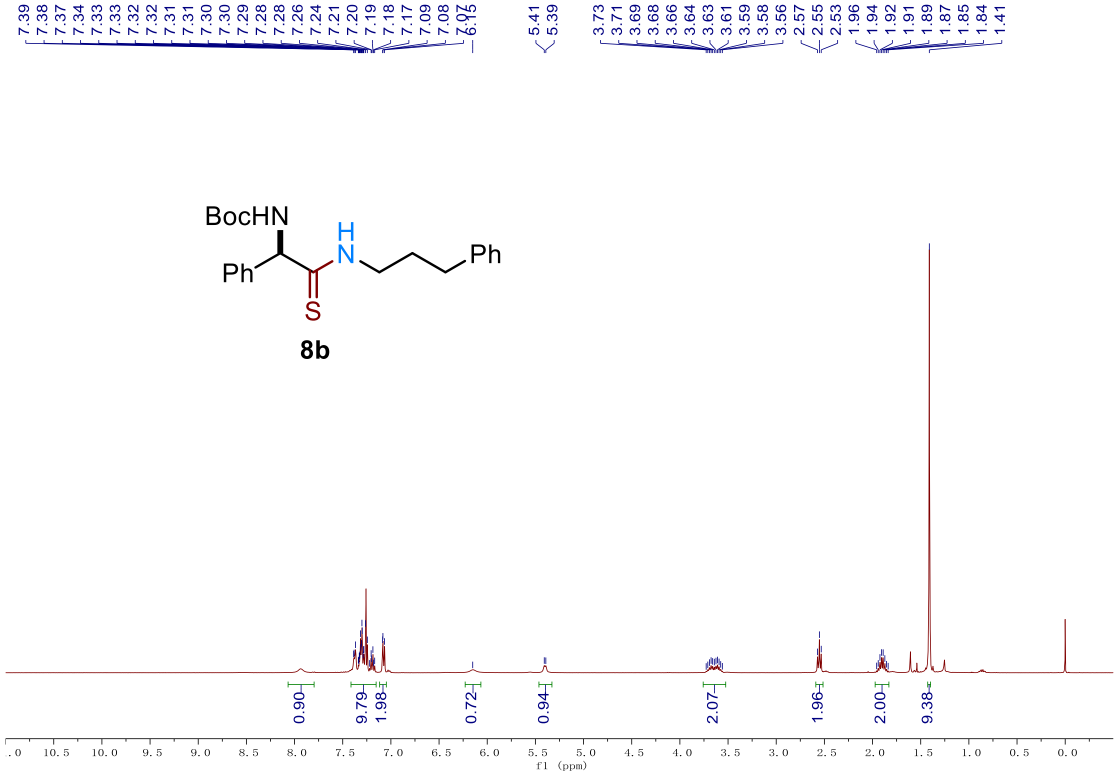

Supplementary Fig. S81 ^{1}H NMR spectrum of compound **8b** (400 MHz, $CDCl_3$)

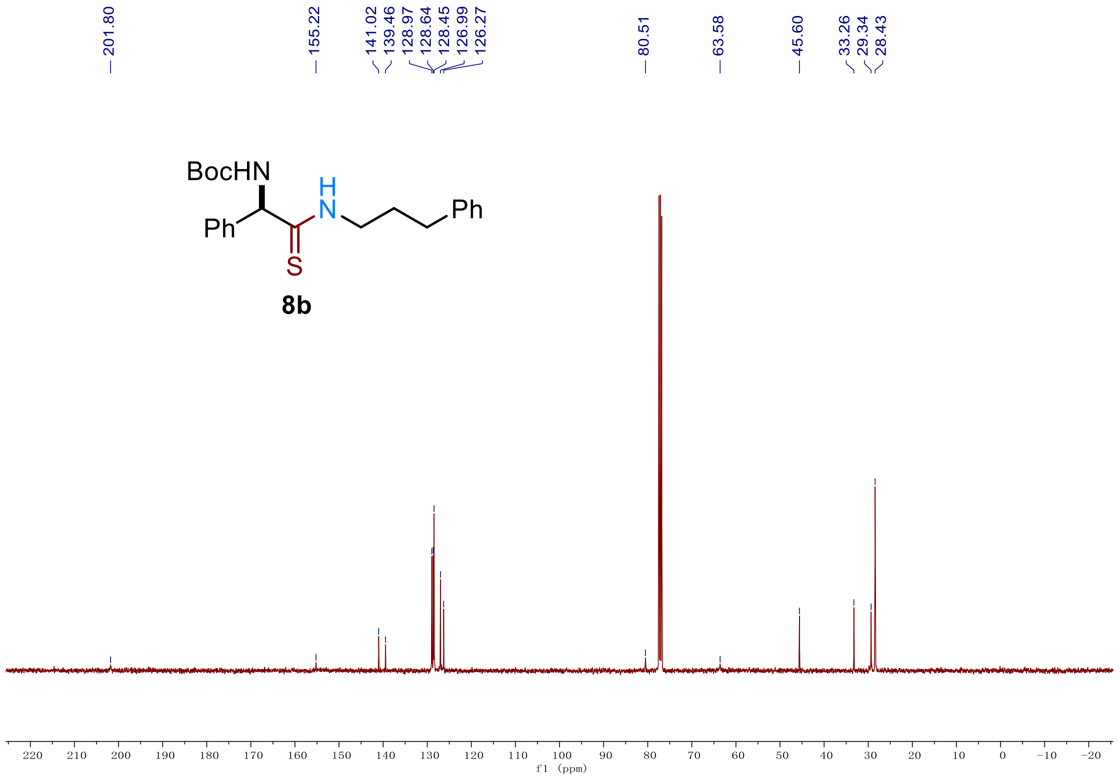

Supplementary Fig. S82 ^{13}C NMR spectrum of compound **8b** (101 MHz, $CDCl_3$)

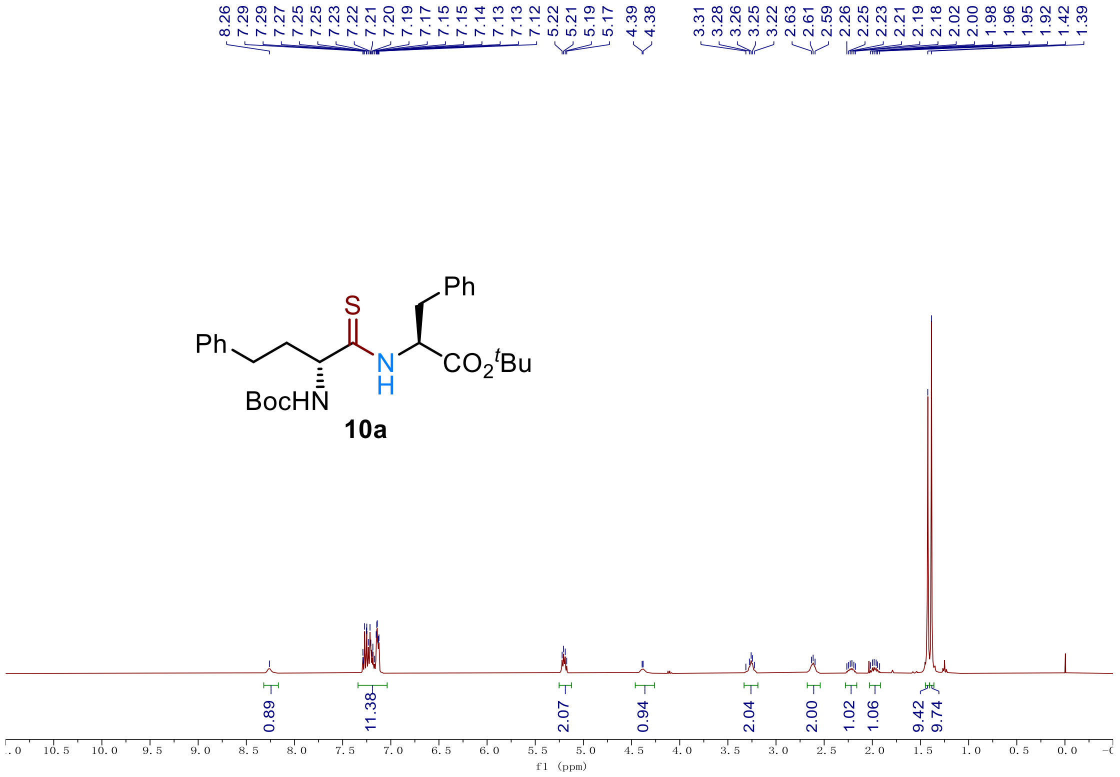

Supplementary Fig. S83 ^{1}H NMR spectrum of compound **10a** (400 MHz, $CDCl_3$)

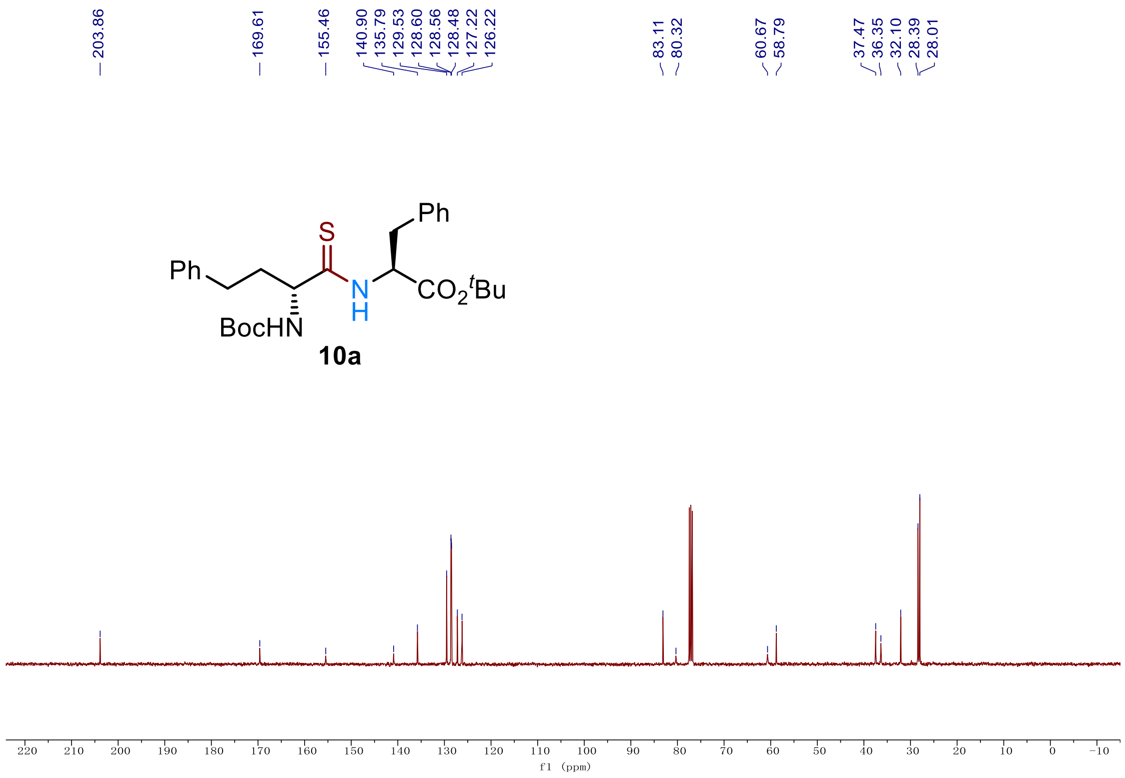

Supplementary Fig. S84 ^{13}C NMR spectrum of compound **10a** (101 MHz, $CDCl_3$)

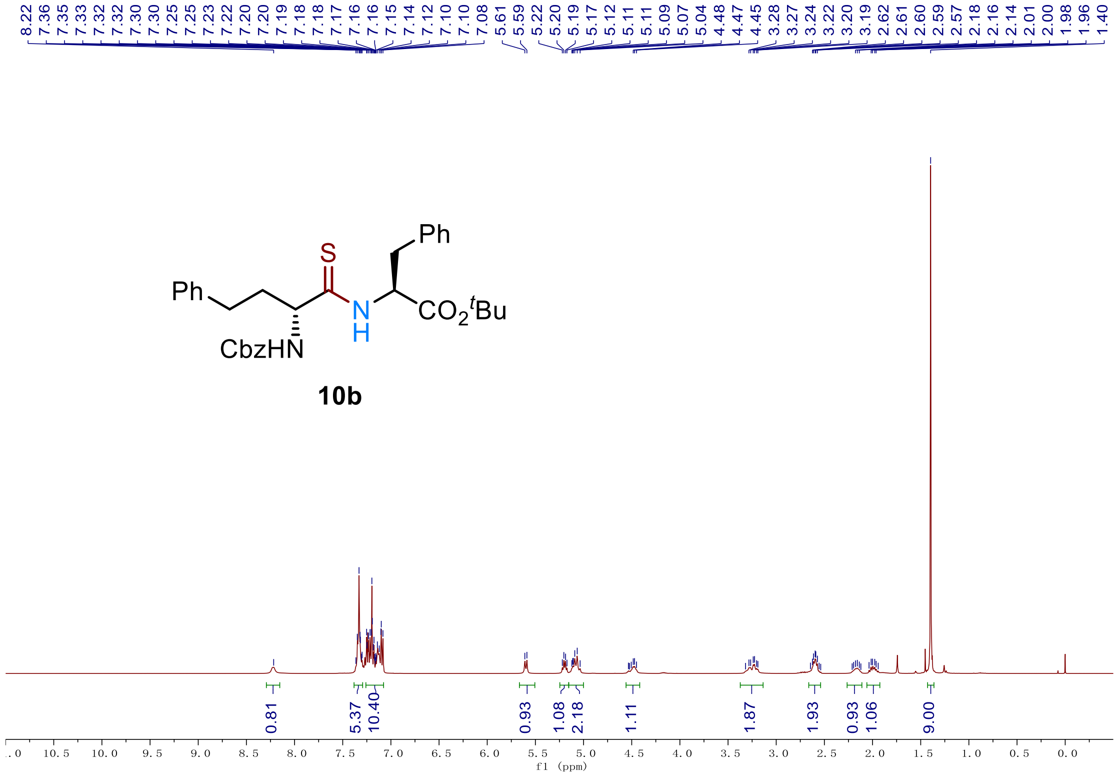

Supplementary Fig. S85 ^{1}H NMR spectrum of compound **10b** (400 MHz, $CDCl_3$)

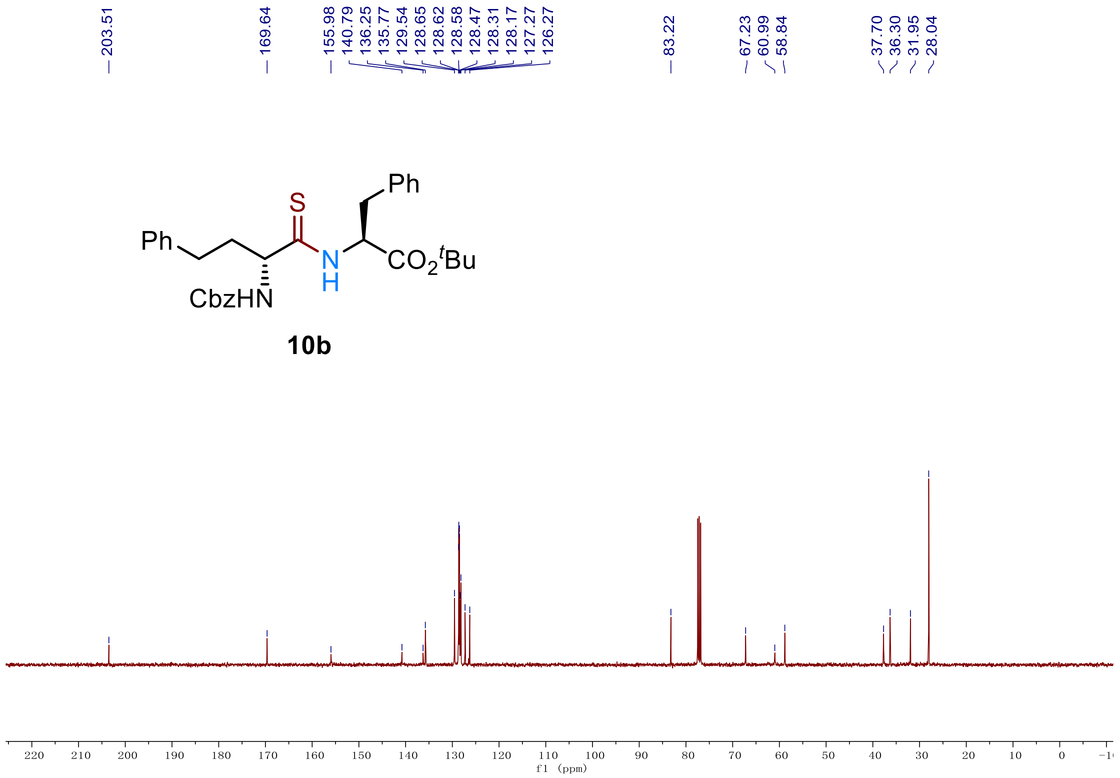

Supplementary Fig. S86 ^{13}C NMR spectrum of compound **10b** (101 MHz, $CDCl_3$)

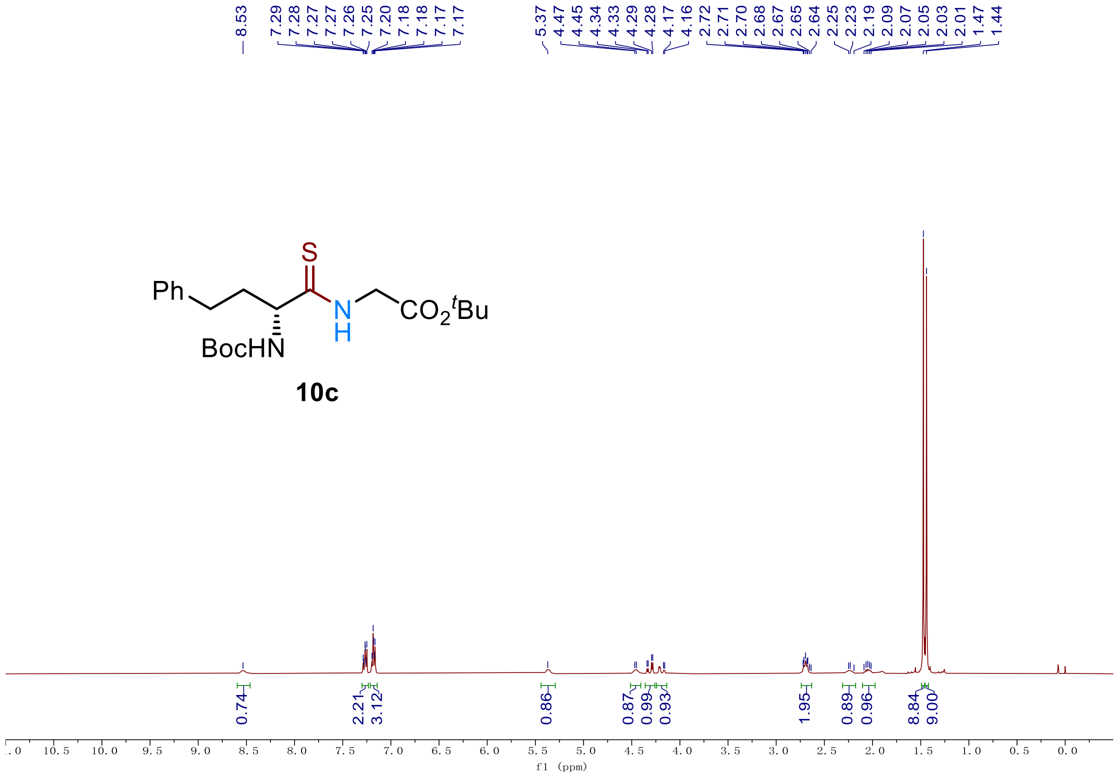

Supplementary Fig. S87 ^{1}H NMR spectrum of compound **10c** (400 MHz, $CDCl_3$)

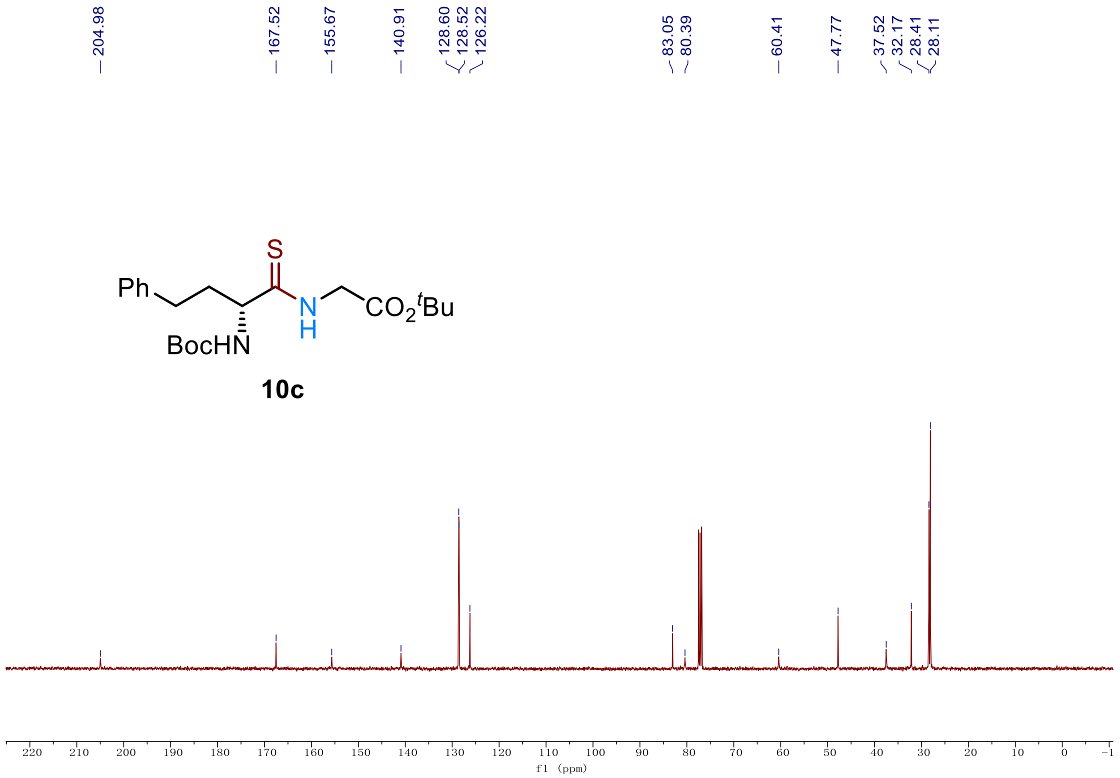

Supplementary Fig. S88 ^{13}C NMR spectrum of compound **10c** (101 MHz, $CDCl_3$)

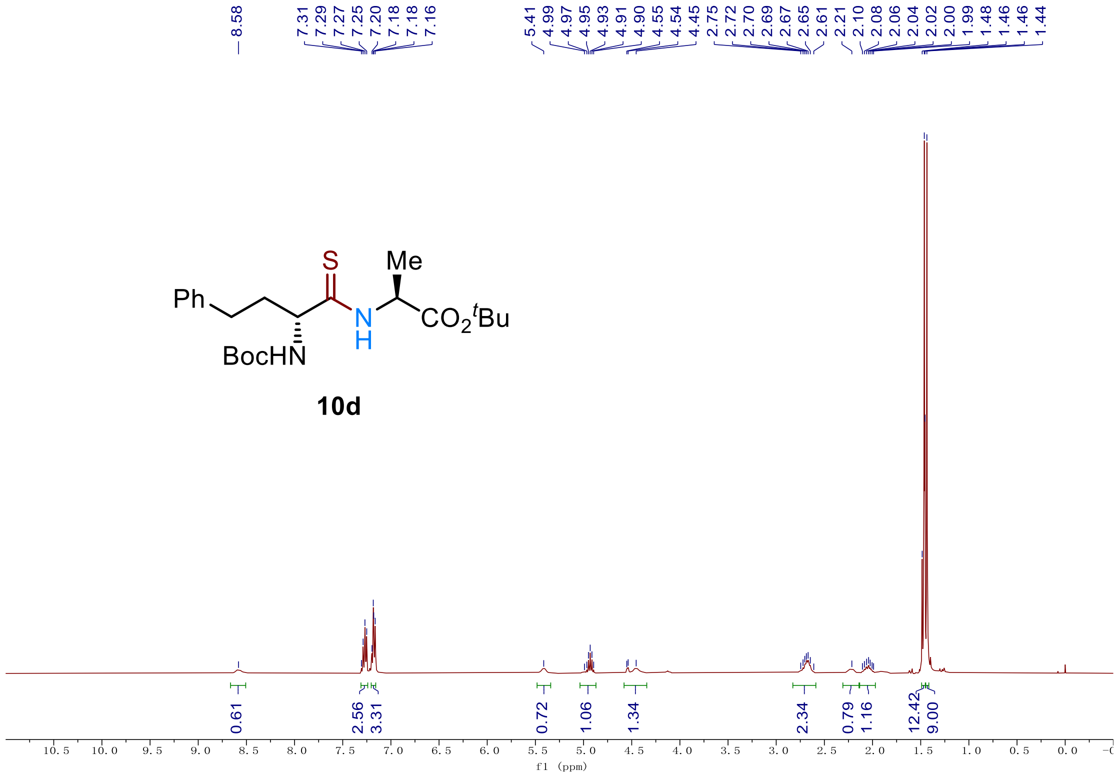

Supplementary Fig. S89 ^{1}H NMR spectrum of compound **10d** (400 MHz, $CDCl_3$)

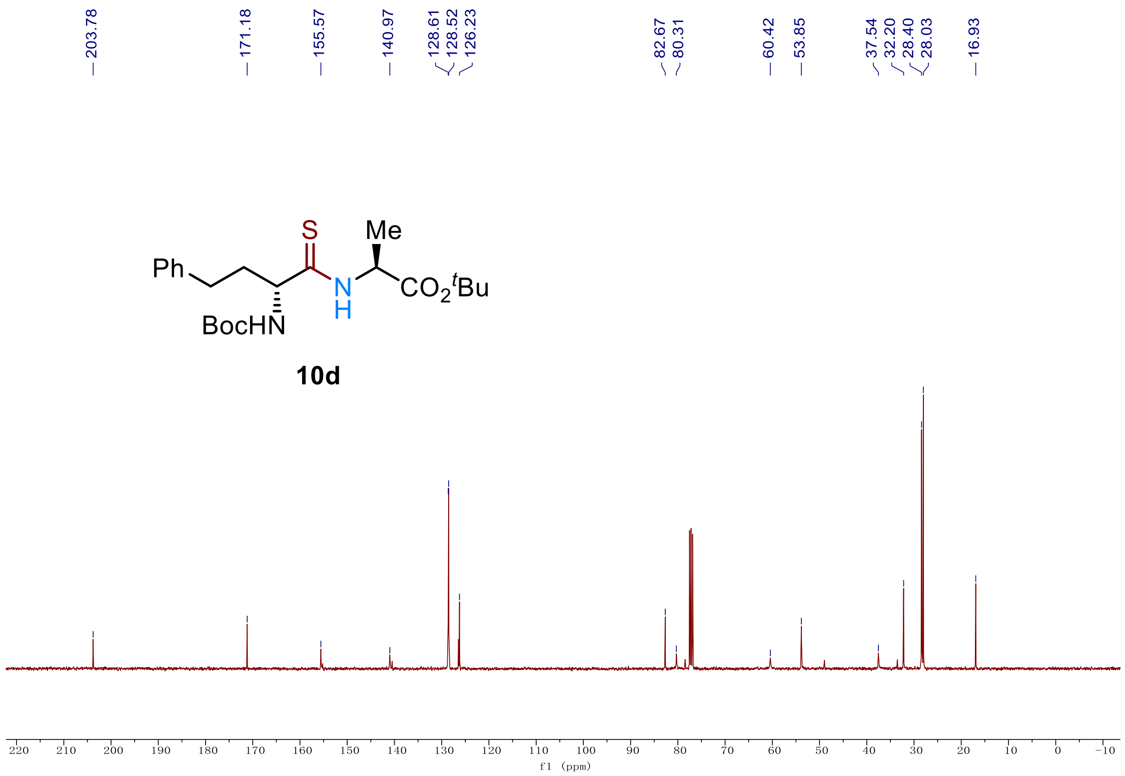

Supplementary Fig. S90 ^{13}C NMR spectrum of compound **10d** (101 MHz, $CDCl_3$)

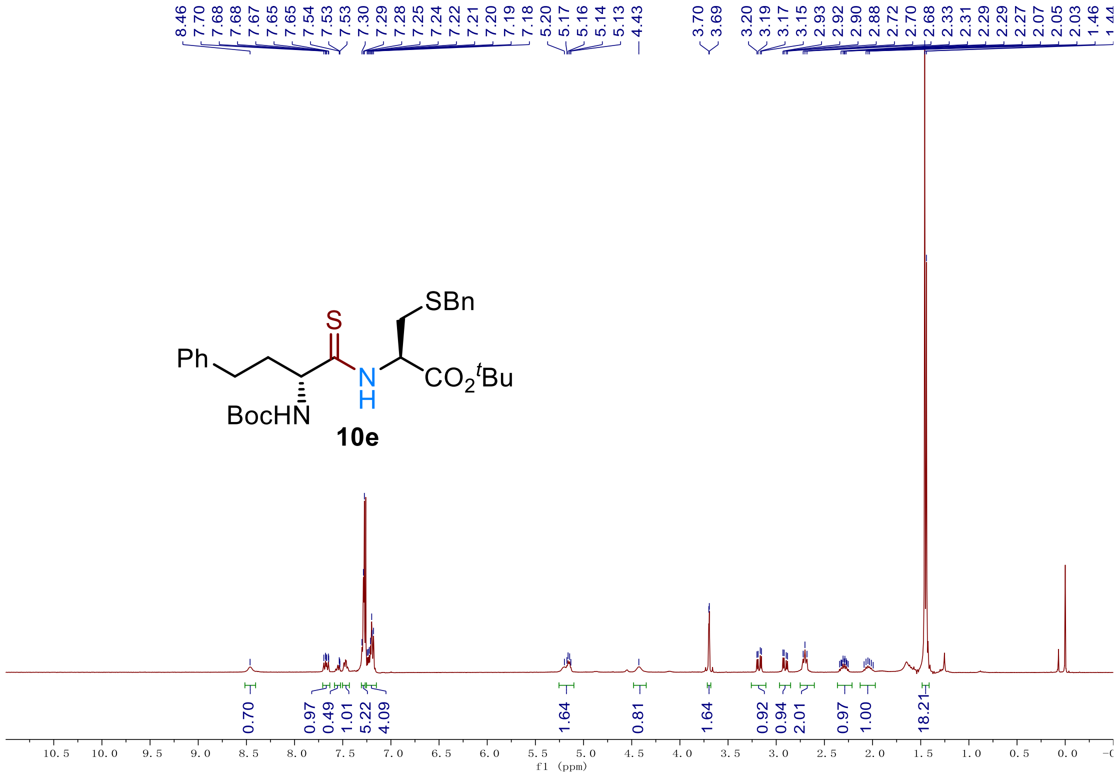

Supplementary Fig. S91 ^1H NMR spectrum of compound **10e** (400 MHz, $CDCl_3$)

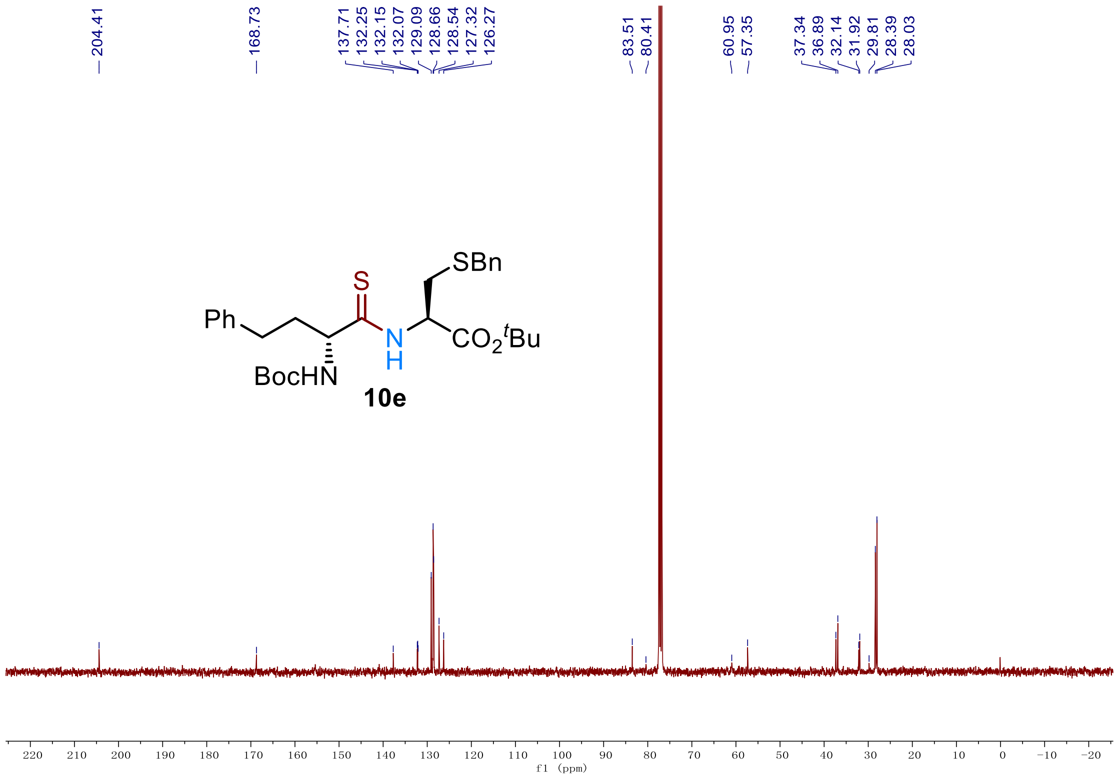

Supplementary Fig. S92 ^{13}C NMR spectrum of compound **10e** (101 MHz, $CDCl_3$)

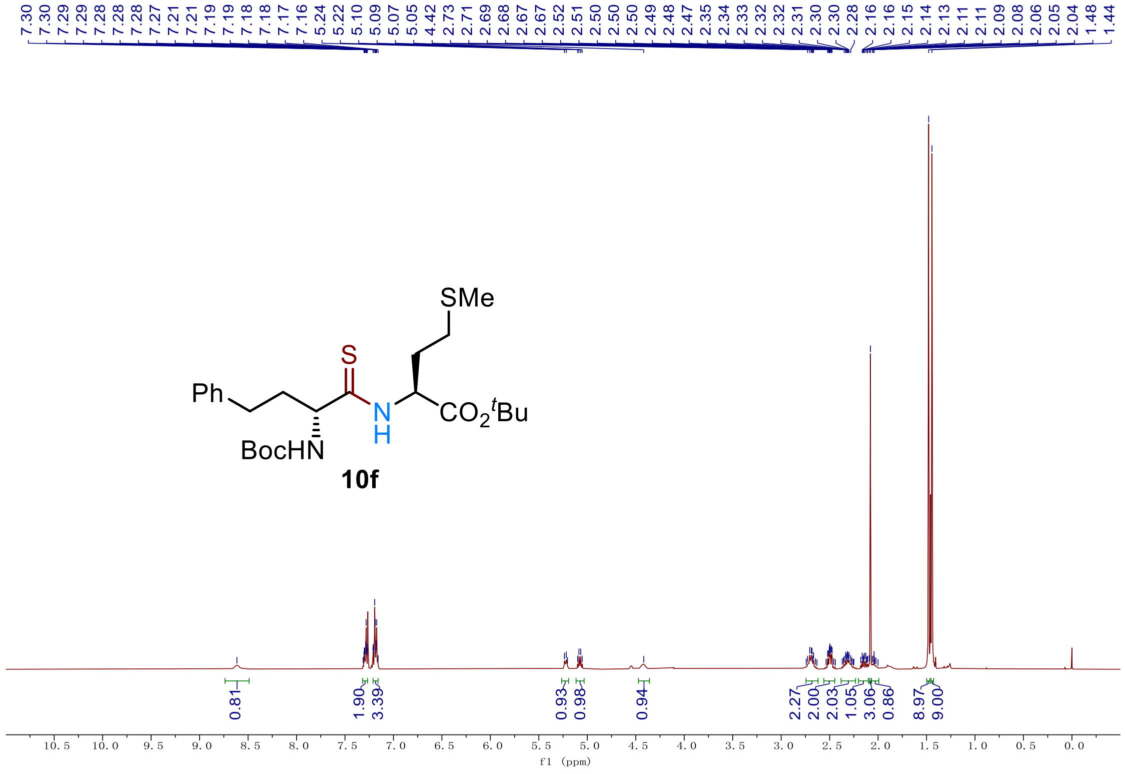

Supplementary Fig. S93 ^{1}H NMR spectrum of compound **10f** (400 MHz, $CDCl_3$)

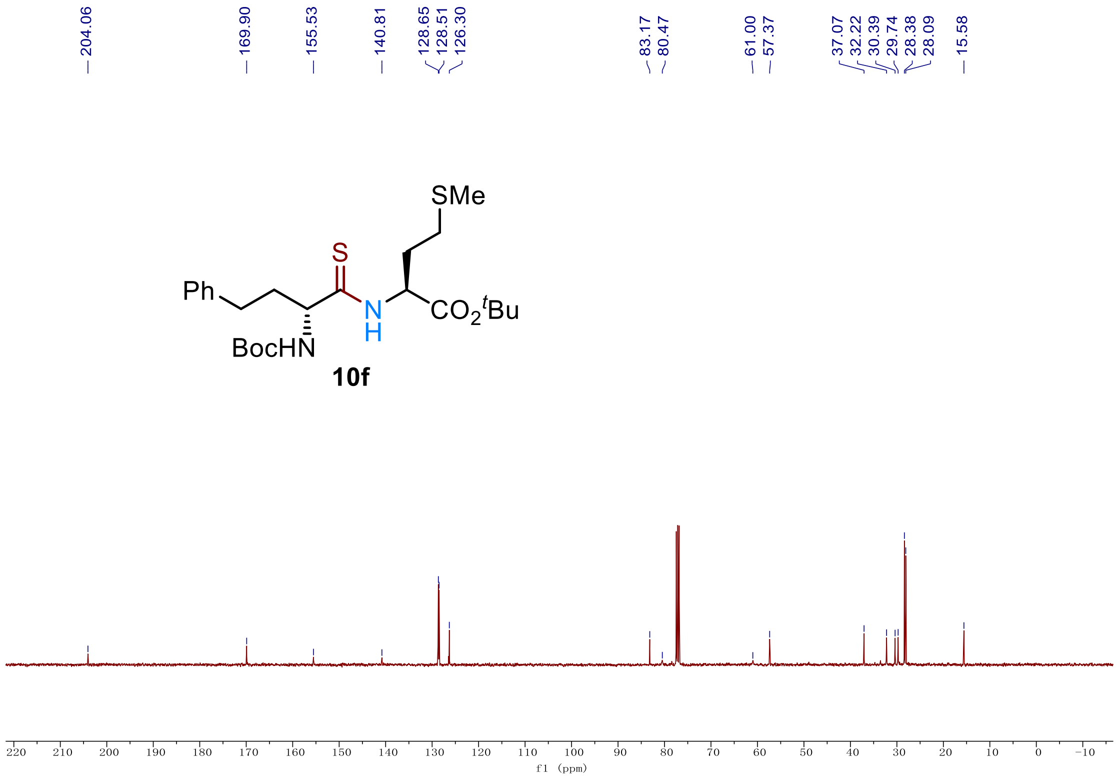

Supplementary Fig. S94 ^{13}C NMR spectrum of compound **10f** (101 MHz, $CDCl_3$)

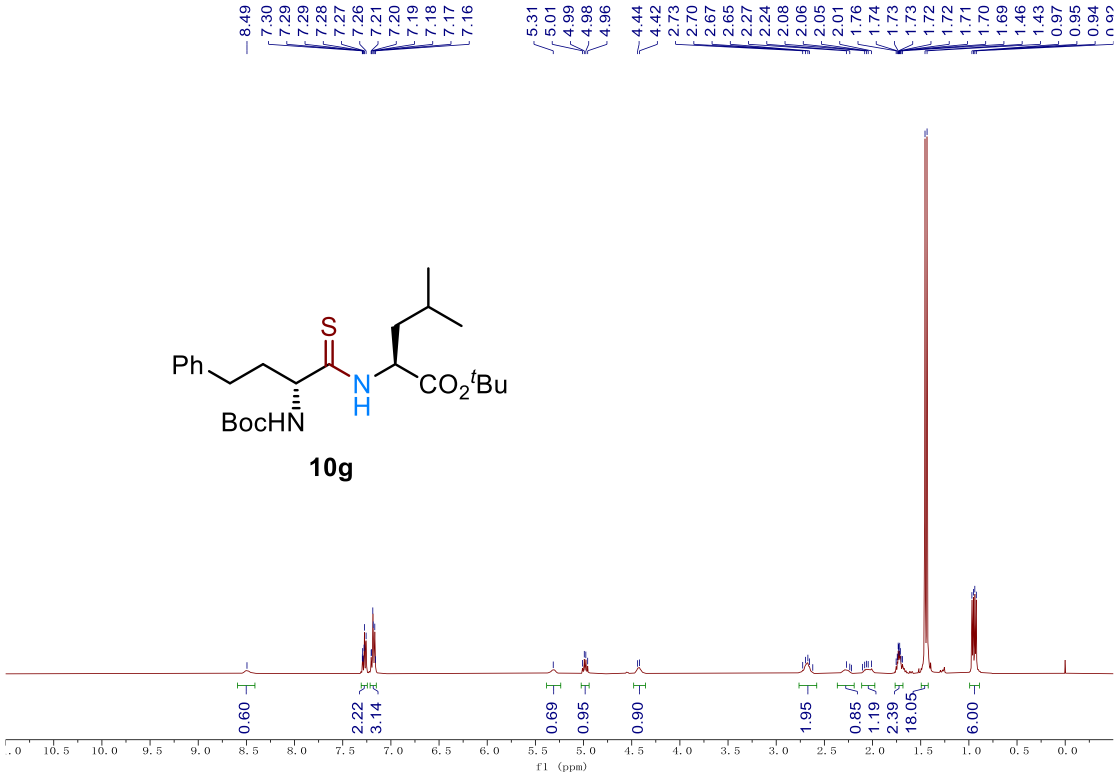

Supplementary Fig. S95 ^{1}H NMR spectrum of compound **10g** (400 MHz, $CDCl_3$)

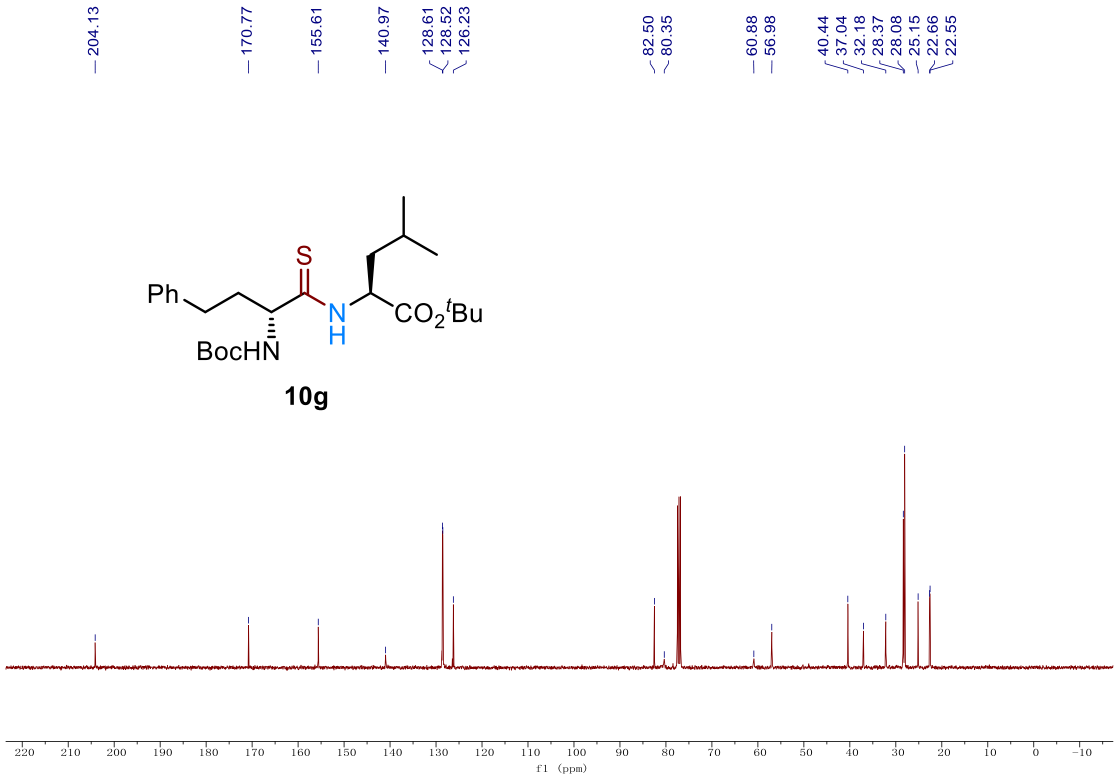

Supplementary Fig. S96 ^{13}C NMR spectrum of compound **10g** (101 MHz, $CDCl_3$)

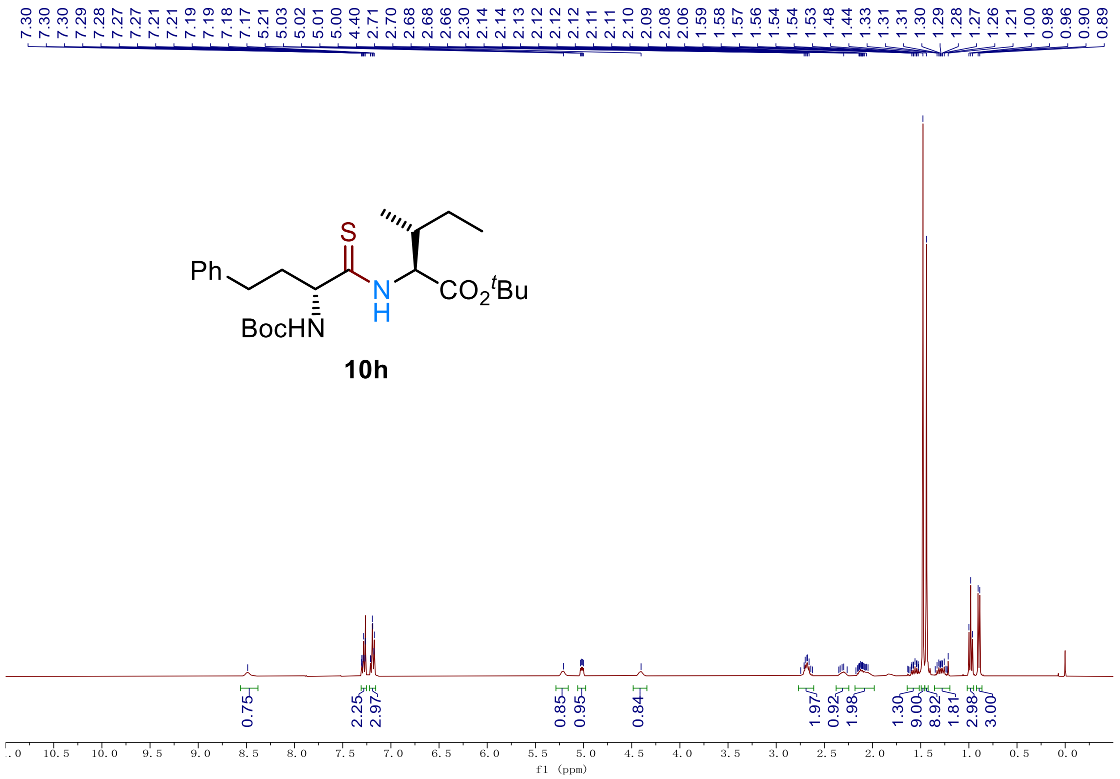

Supplementary Fig. S97 ^{1}H NMR spectrum of compound **10h** (400 MHz, $CDCl_3$)

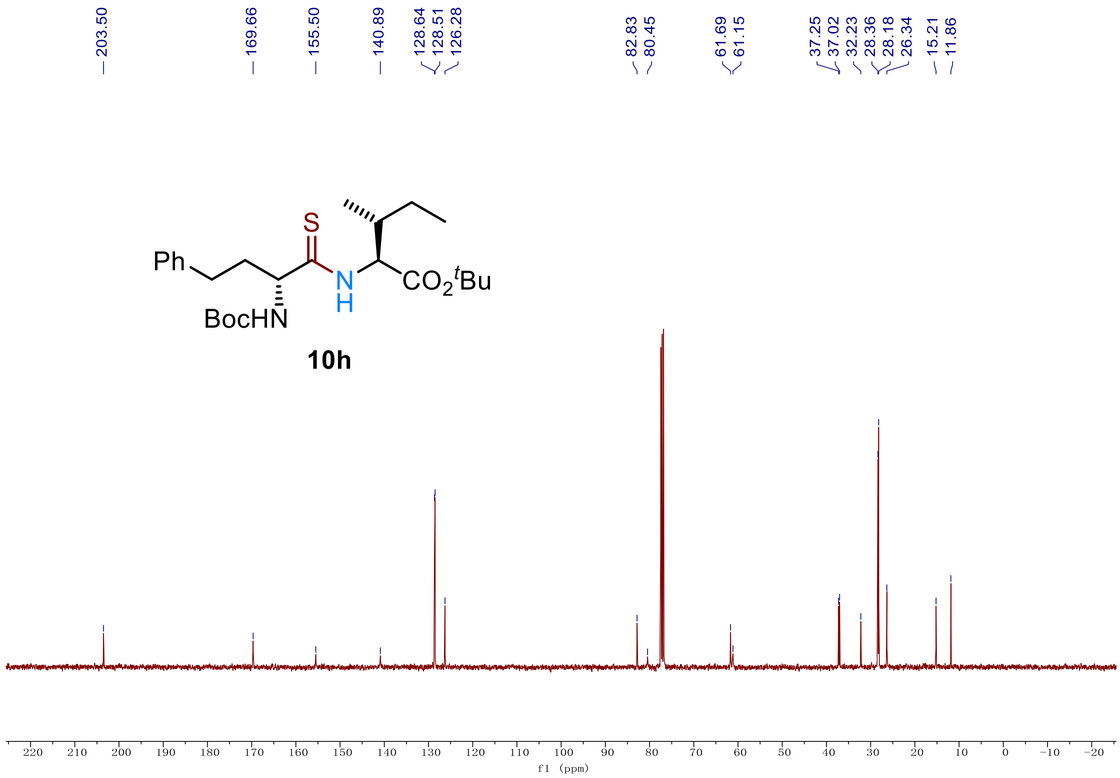

Supplementary Fig. S98 ^{13}C NMR spectrum of compound **10h** (101 MHz, $CDCl_3$)

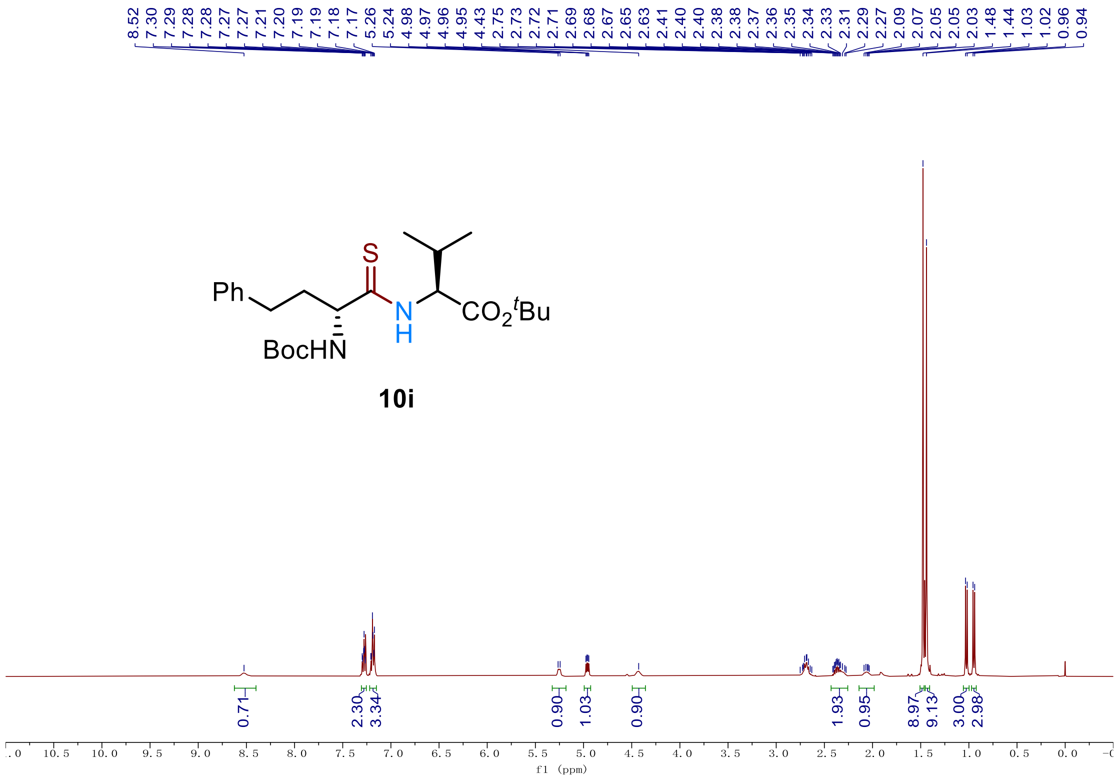

Supplementary Fig. S99 ^{1}H NMR spectrum of compound **10i** (400 MHz, $CDCl_3$)

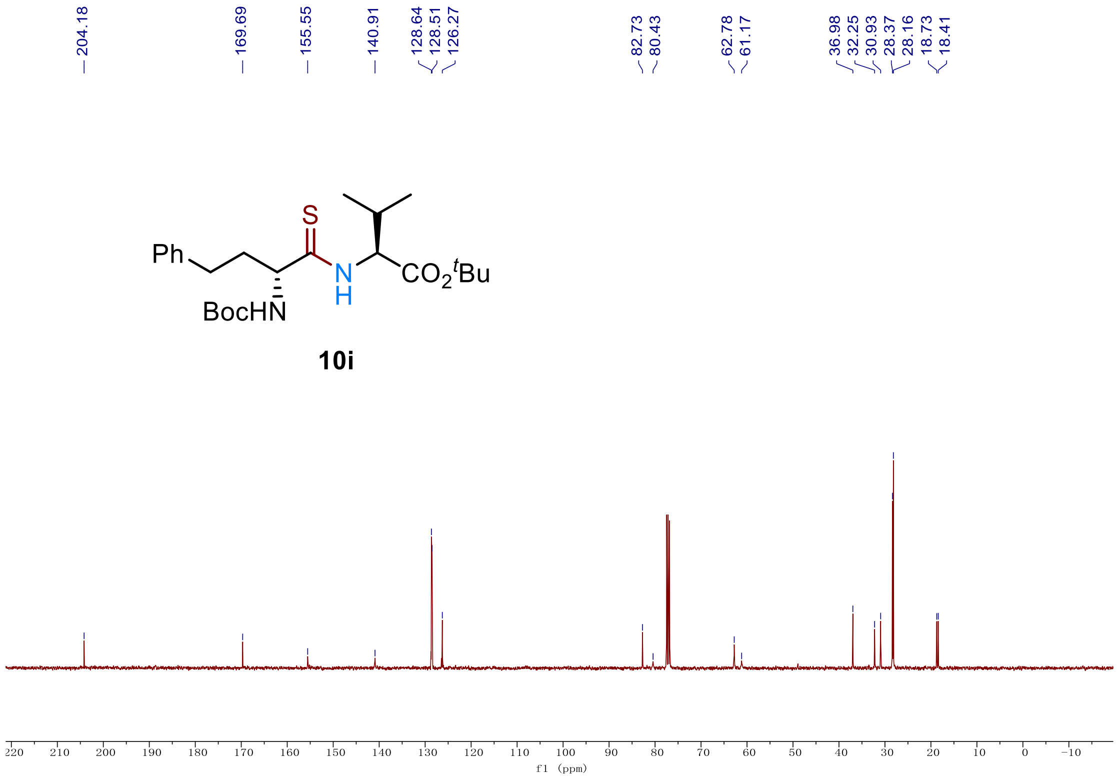

Supplementary Fig. S100 ^{13}C NMR spectrum of compound **10i** (101 MHz, $CDCl_3$)

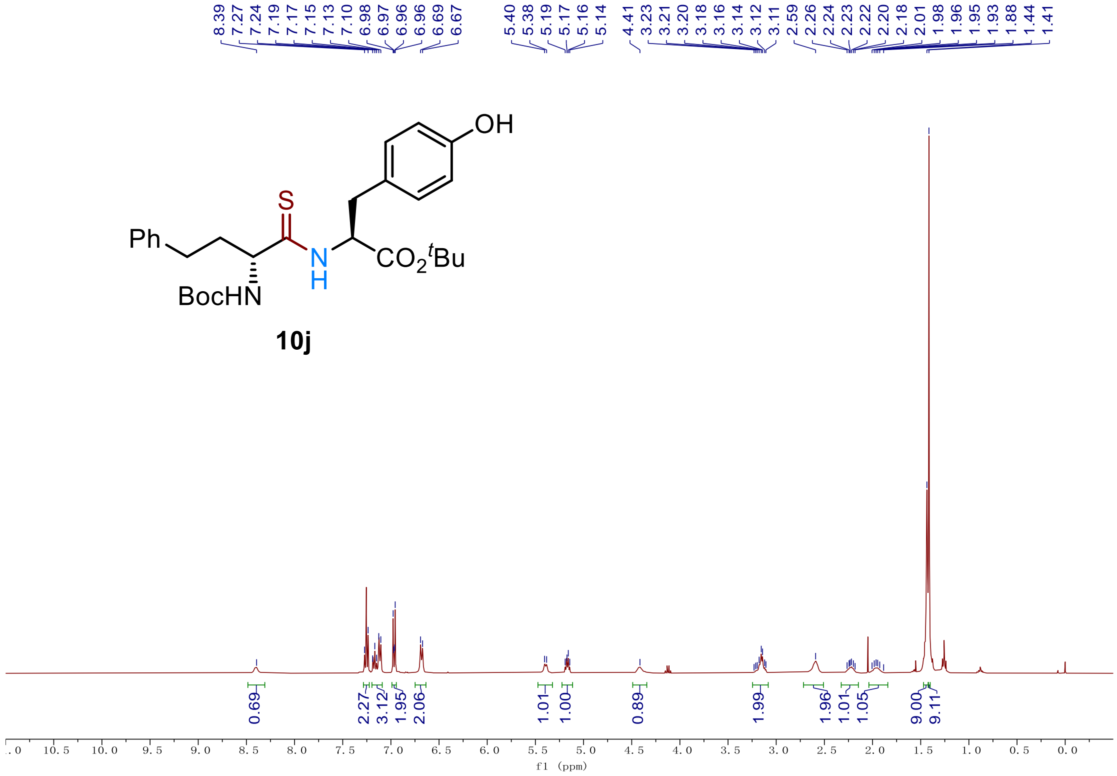

Supplementary Fig. S101 ^{1}H NMR spectrum of compound **10j** (400 MHz, $CDCl_3$)

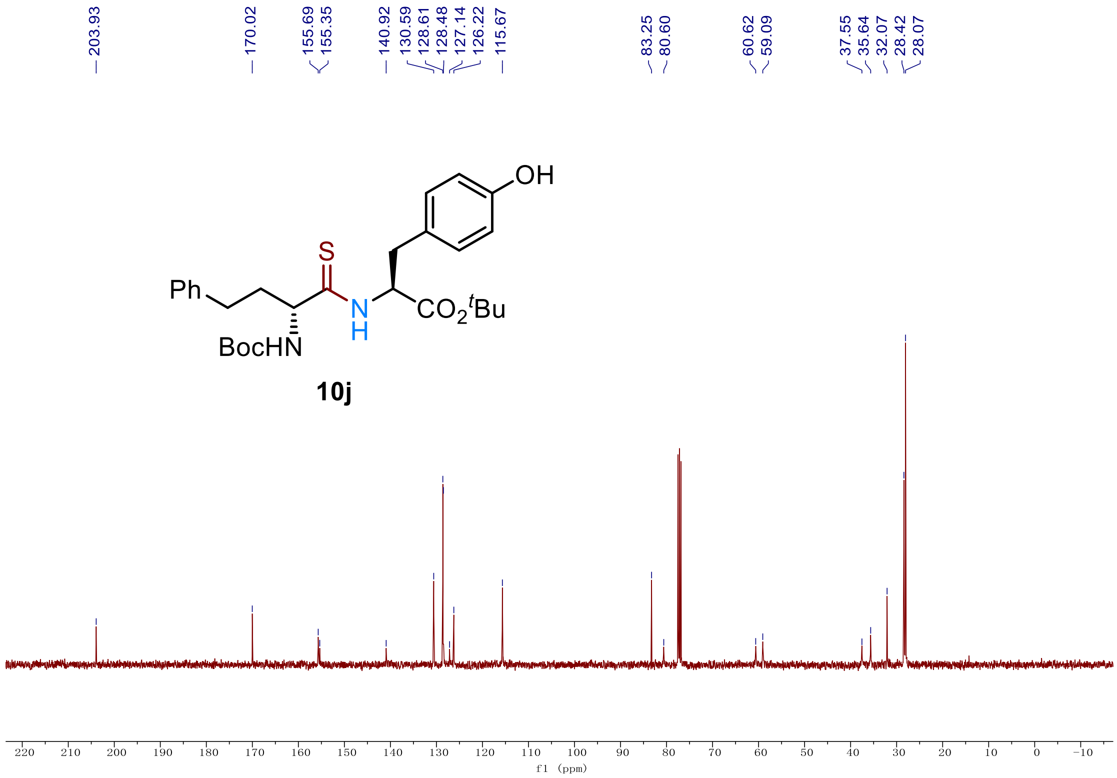

Supplementary Fig. S102 ^{13}C NMR spectrum of compound **10j** (101 MHz, $CDCl_3$)

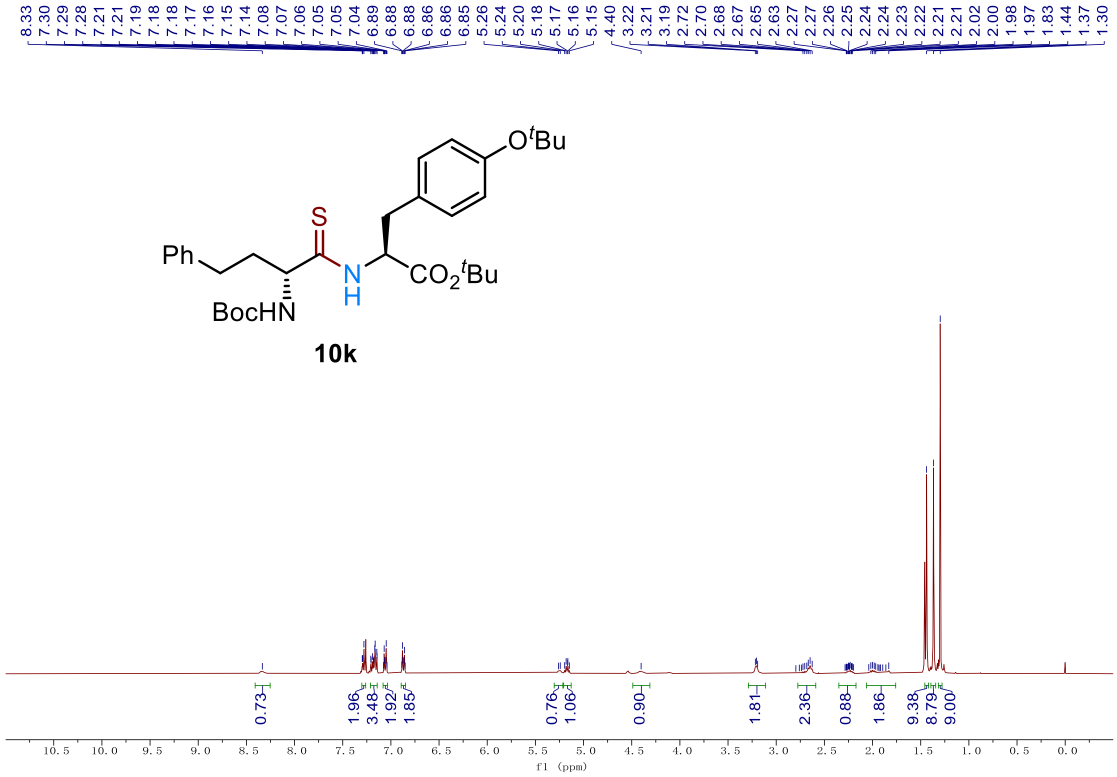

Supplementary Fig. S103 ^{1}H NMR spectrum of compound **10k** (400 MHz, $CDCl_3$)

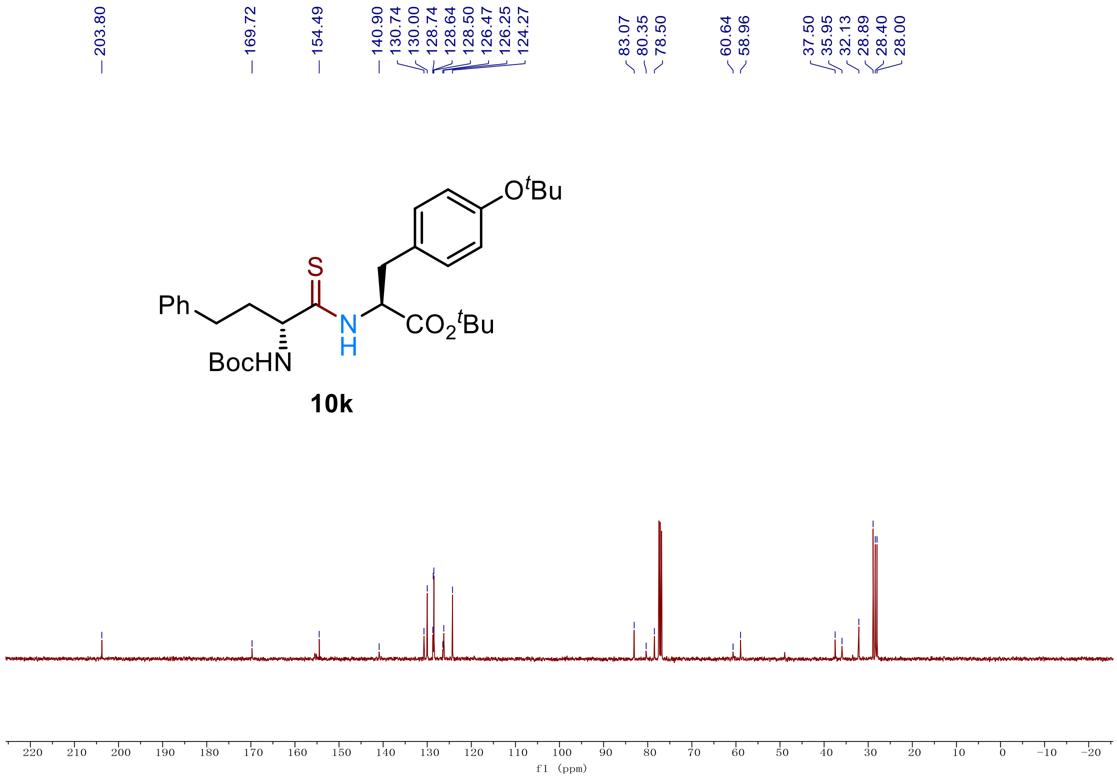

Supplementary Fig. S104 ^{13}C NMR spectrum of compound **10k** (101 MHz, $CDCl_3$)

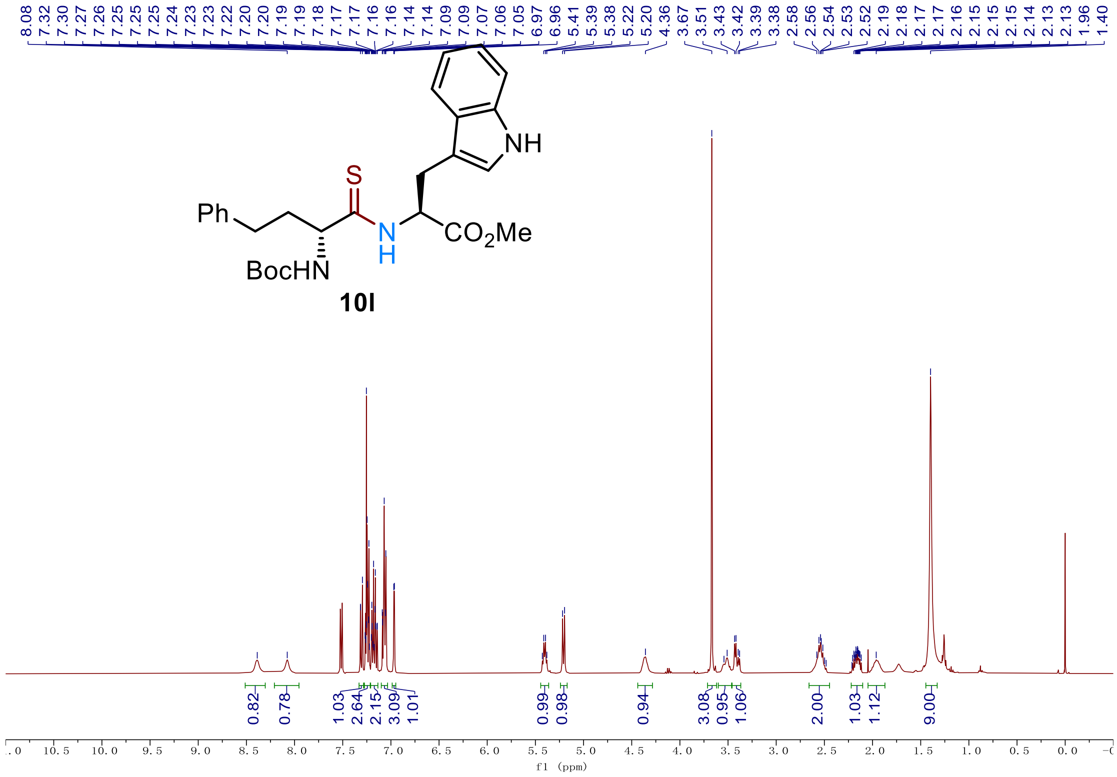

Supplementary Fig. S105 ^{1}H NMR spectrum of compound **10l** (400 MHz, $CDCl_3$)

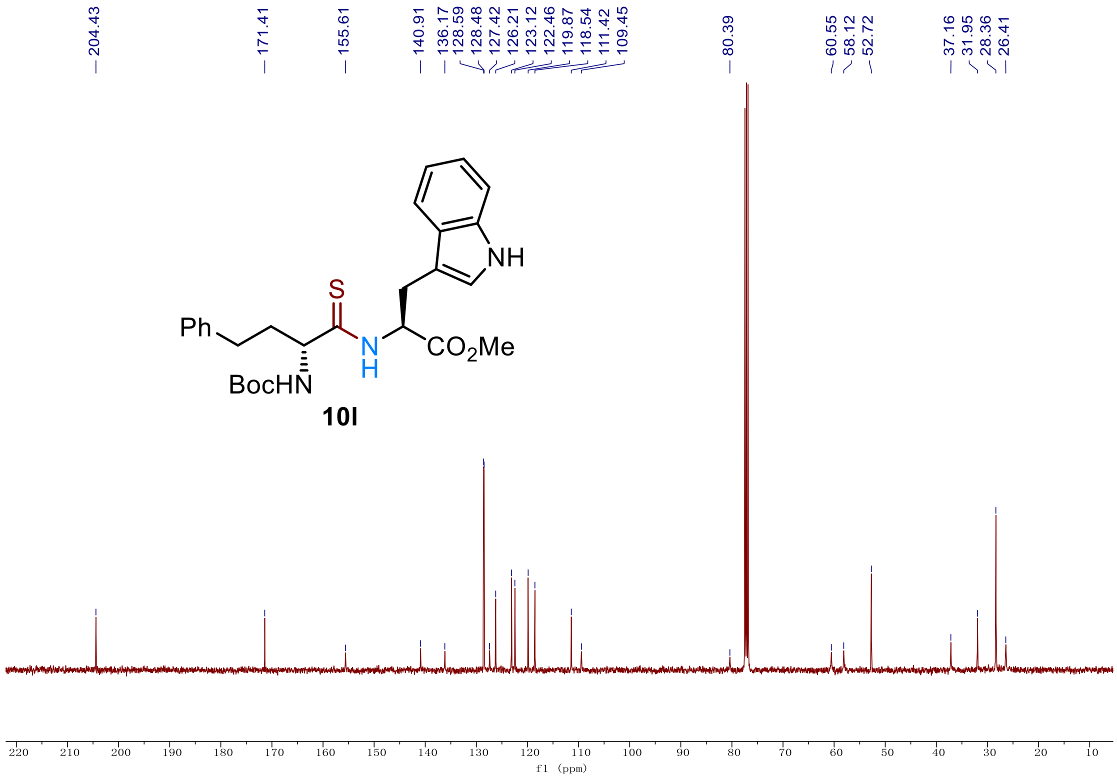

Supplementary Fig. S106 ^{13}C NMR spectrum of compound **10l** (101 MHz, $CDCl_3$)

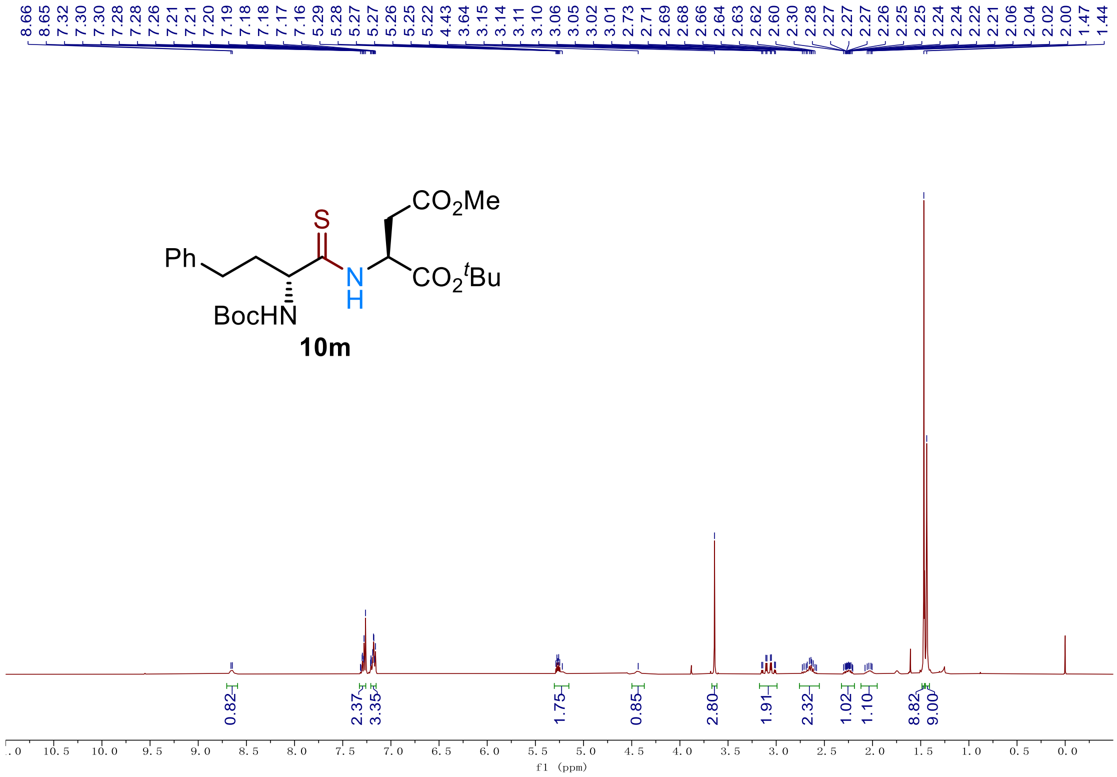

Supplementary Fig. S107 ^{1}H NMR spectrum of compound **10m** (400 MHz, $CDCl_3$)

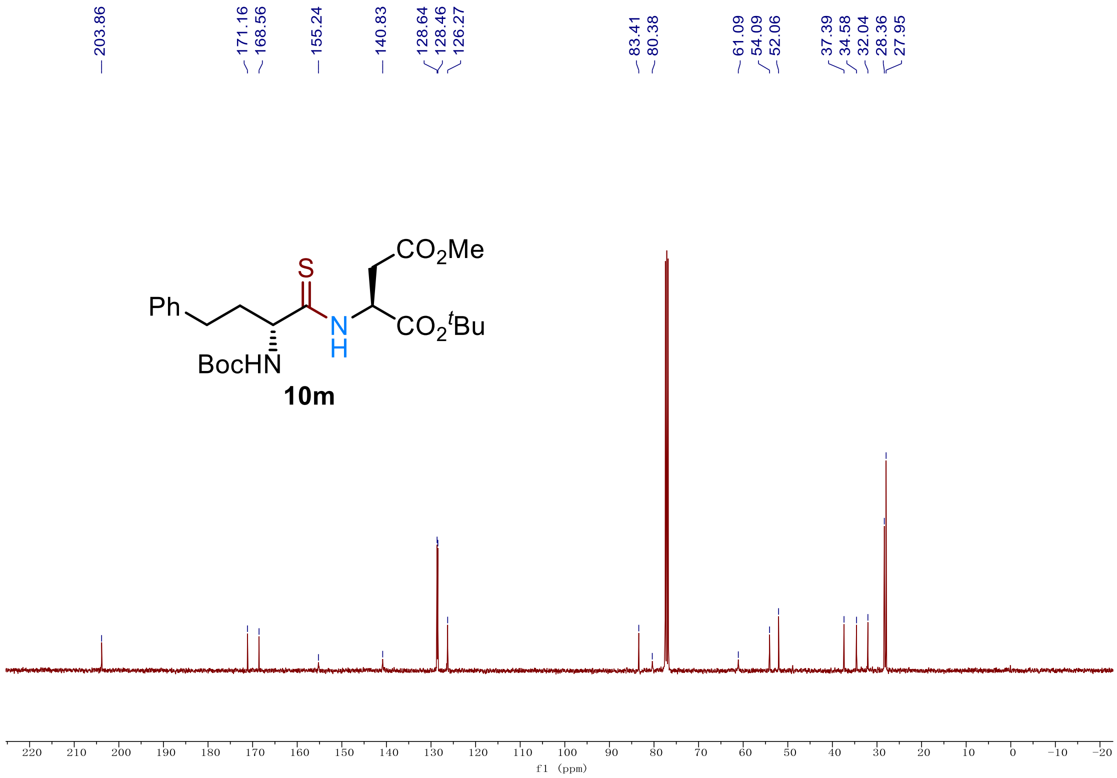

Supplementary Fig. S108 ^{13}C NMR spectrum of compound **10m** (101 MHz, $CDCl_3$)

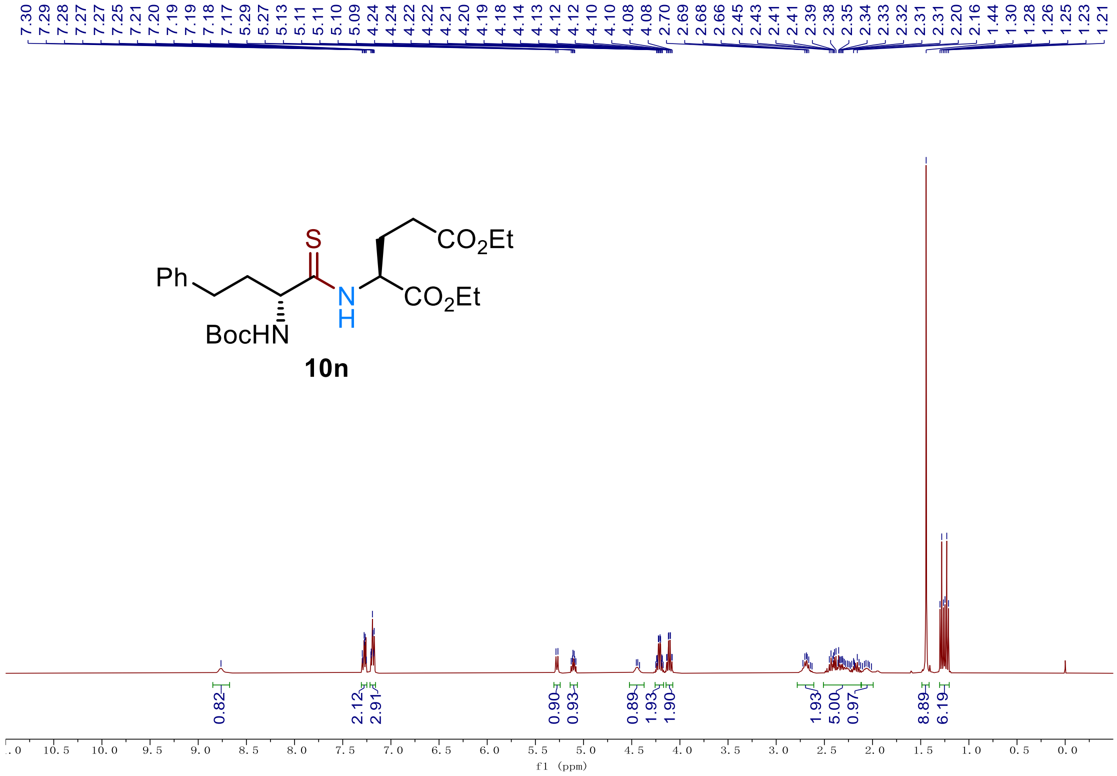

Supplementary Fig. S109 ^{1}H NMR spectrum of compound **10n** (400 MHz, $CDCl_3$)

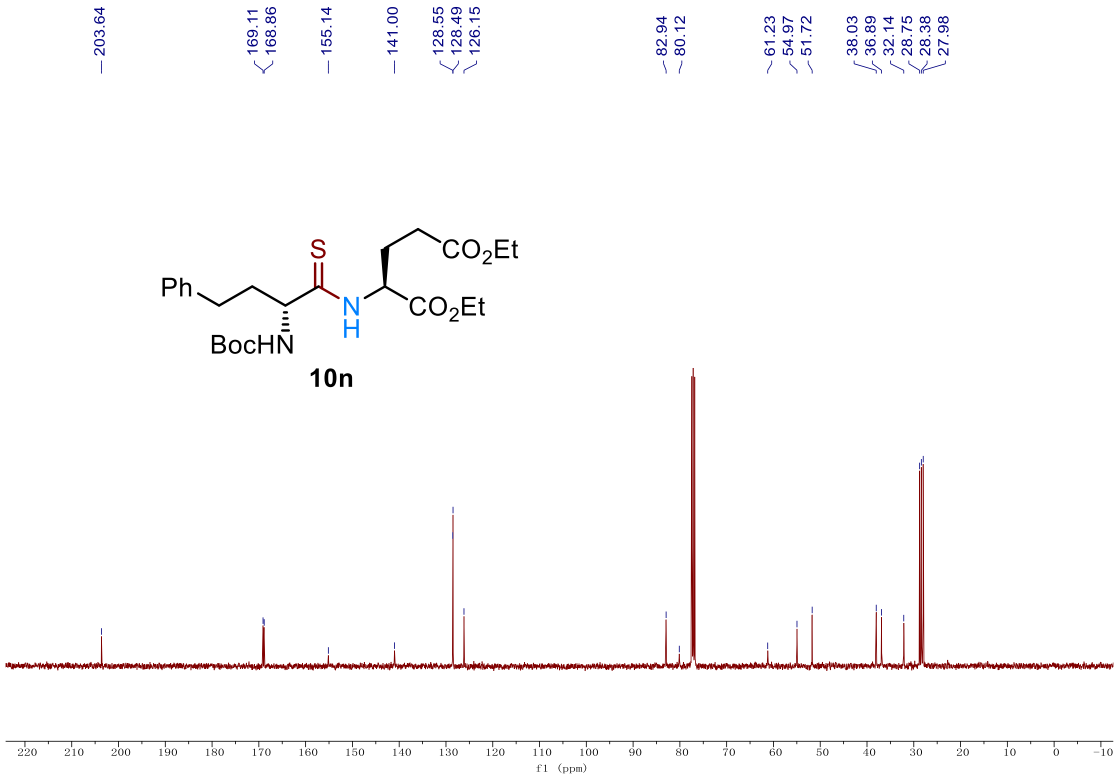

Supplementary Fig. S110 ^{13}C NMR spectrum of compound **10n** (101 MHz, $CDCl_3$)

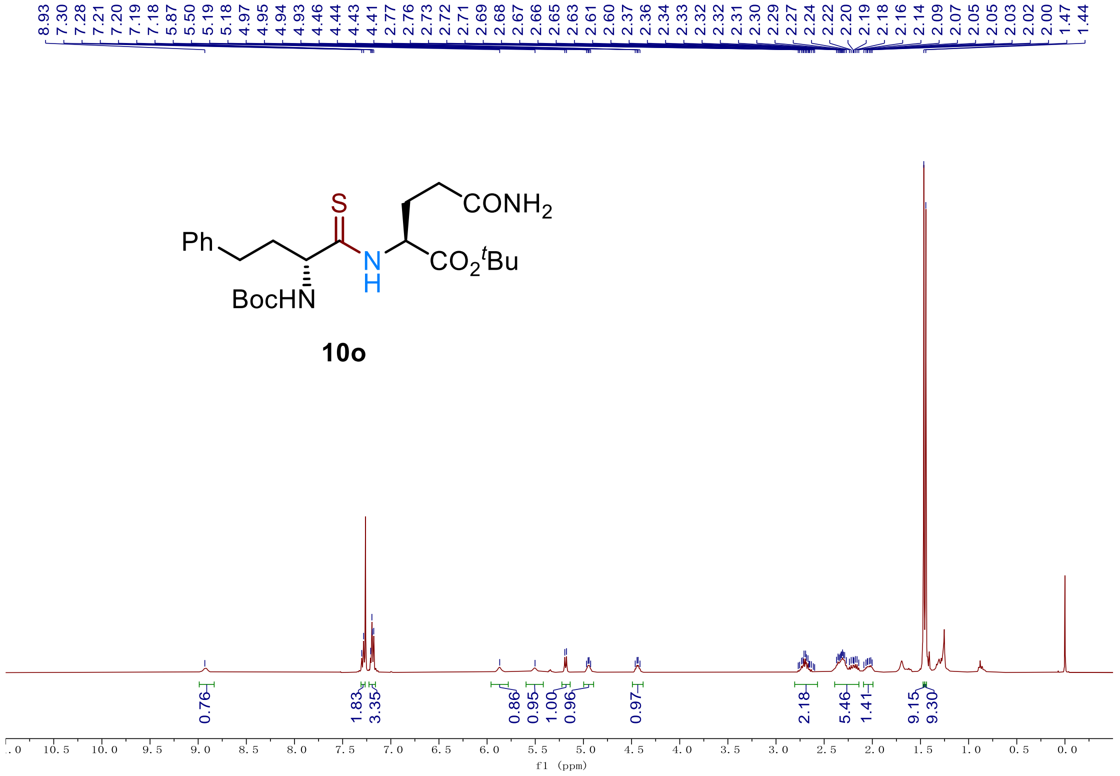

Supplementary Fig. S111 ^{1}H NMR spectrum of compound **10o** (400 MHz, $CDCl_3$)

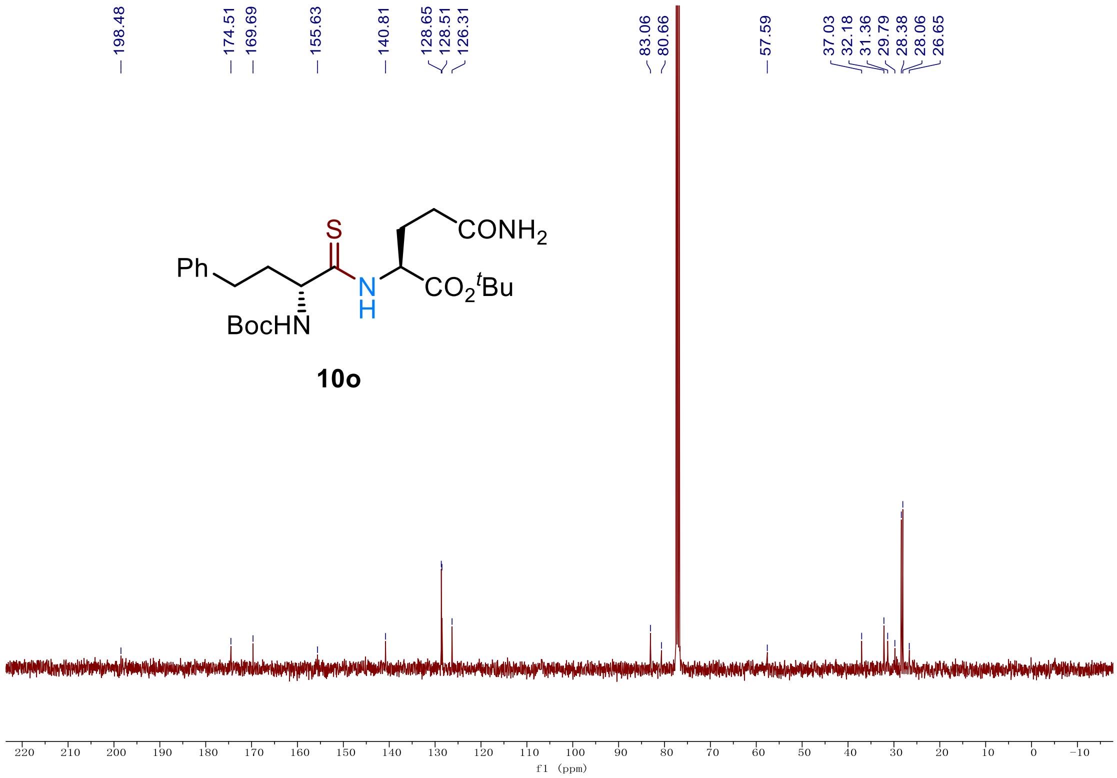

Supplementary Fig. S112 ^{13}C NMR spectrum of compound **10o** (101 MHz, $CDCl_3$)

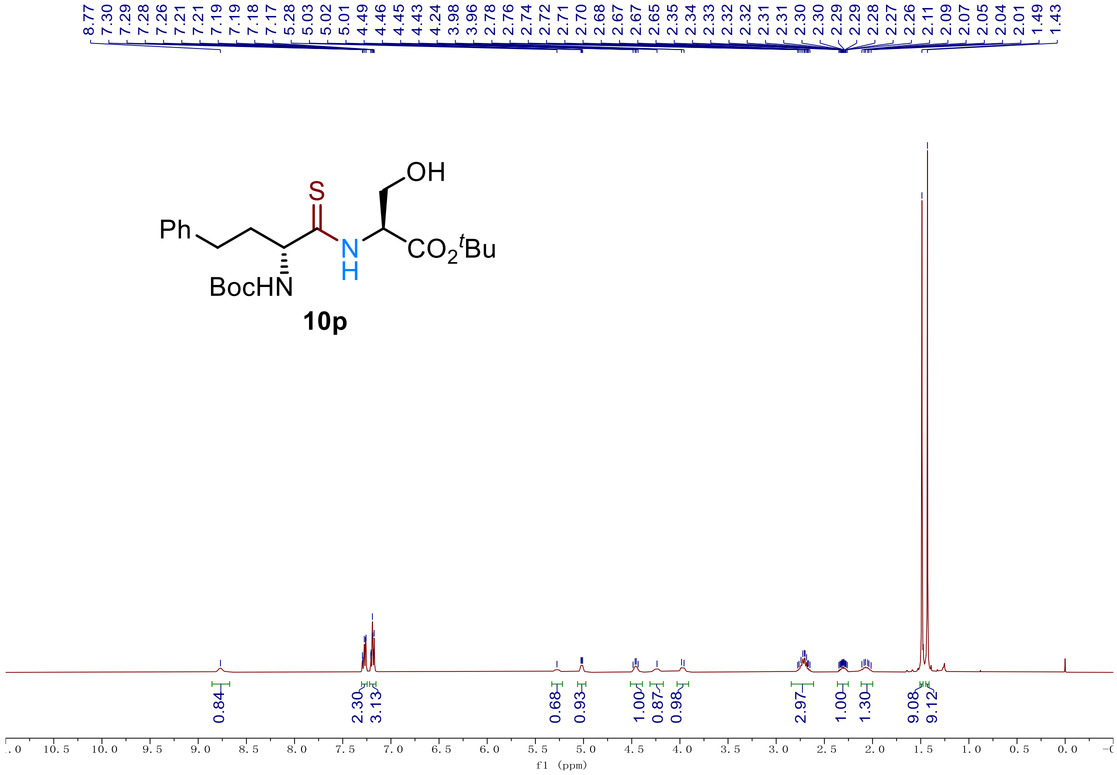

Supplementary Fig. S113 ^{1}H NMR spectrum of compound **10p** (400 MHz, $CDCl_3$)

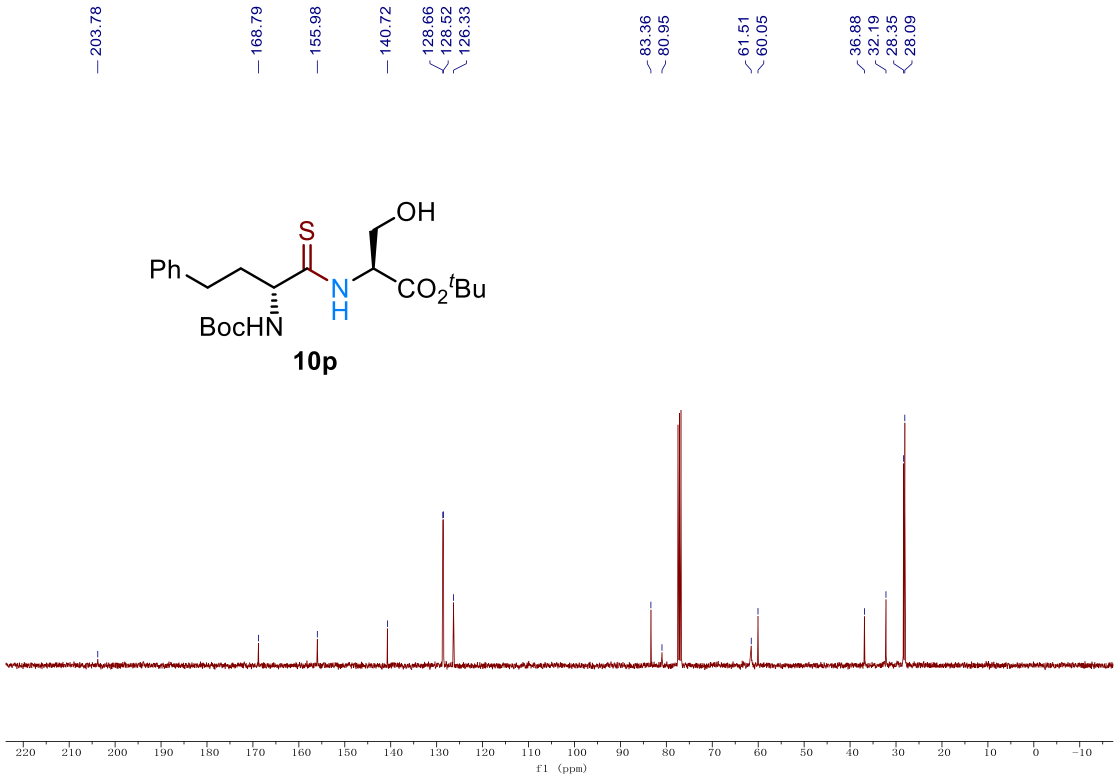

Supplementary Fig. S114 ^{13}C NMR spectrum of compound **10p** (101 MHz, $CDCl_3$)

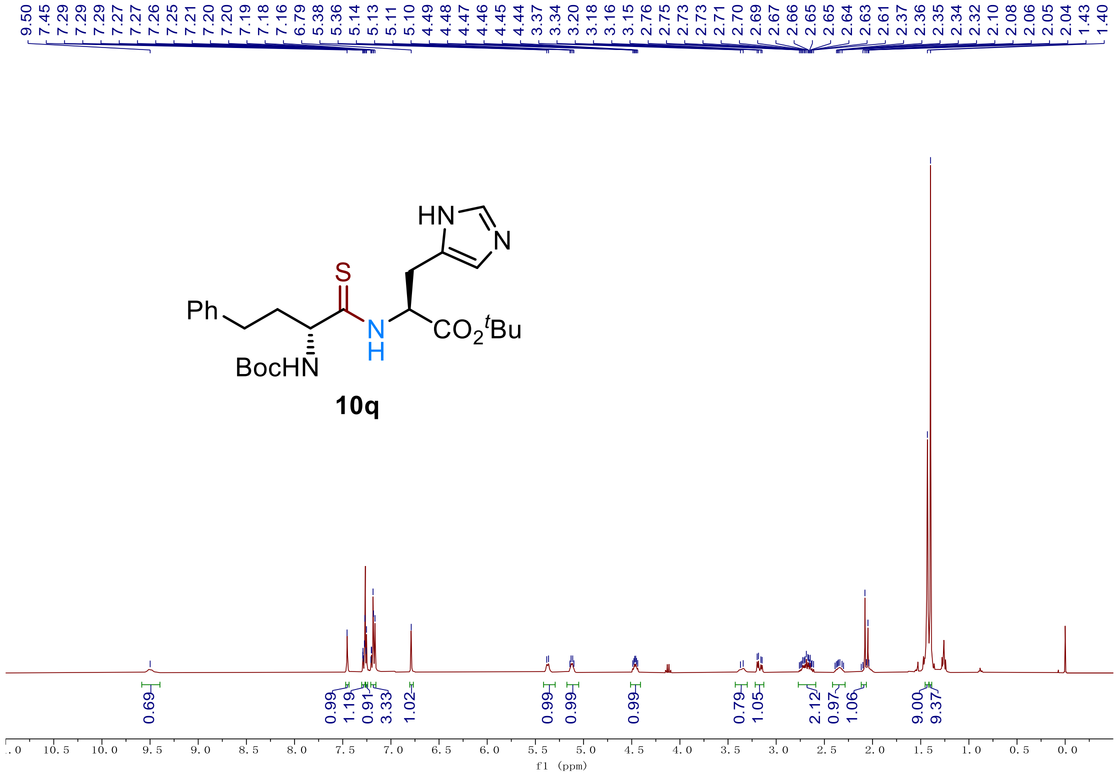

Supplementary Fig. S115 ^{1}H NMR spectrum of compound **10q** (400 MHz, $CDCl_3$)

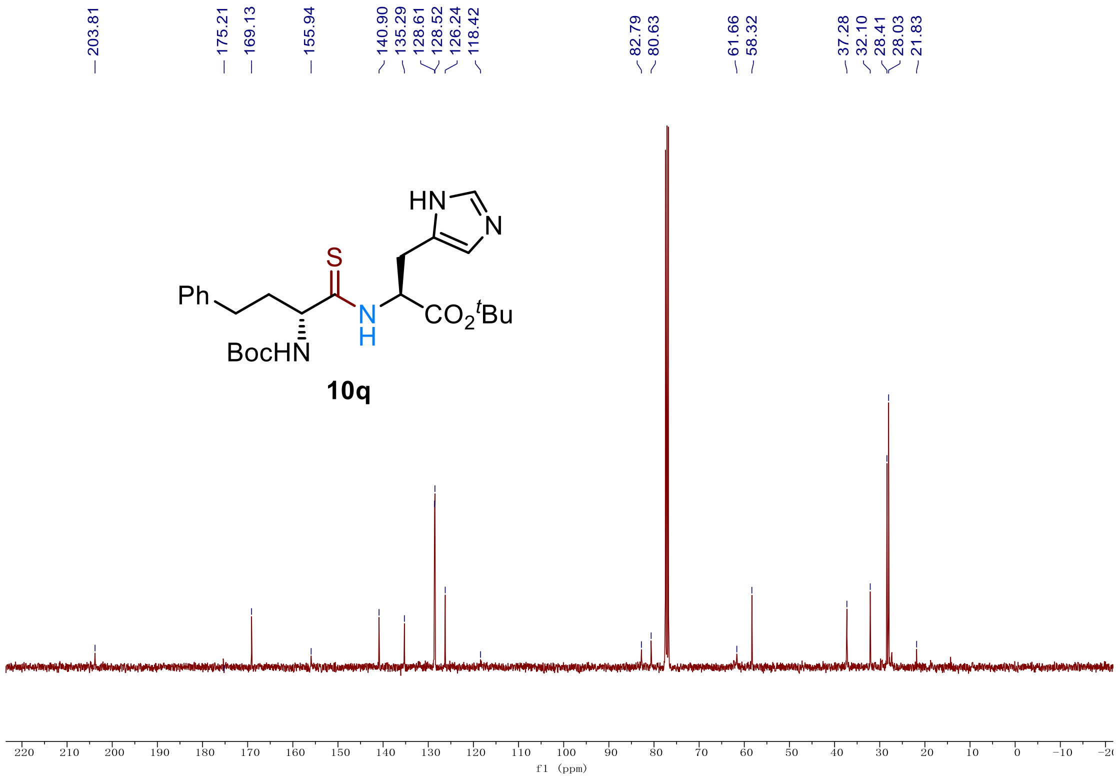

Supplementary Fig. S116 ^{13}C NMR spectrum of compound **10q** (101 MHz, $CDCl_3$)

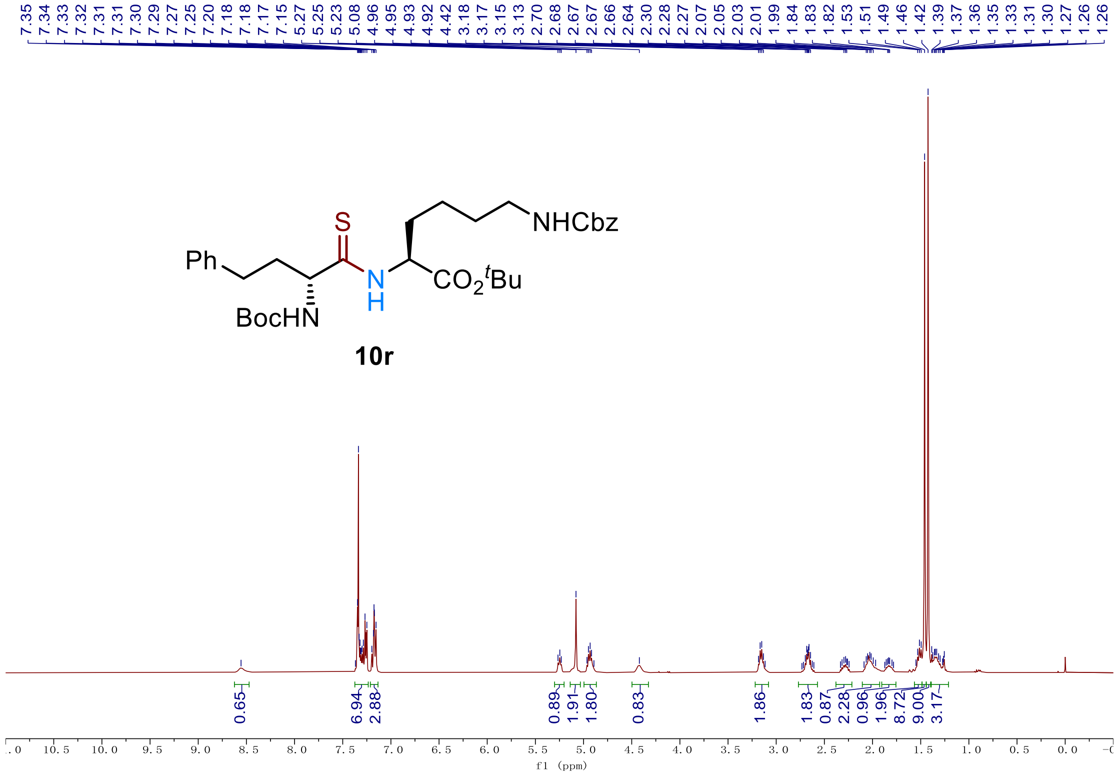

Supplementary Fig. S117 ^{1}H NMR spectrum of compound **10r** (400 MHz, $CDCl_3$)

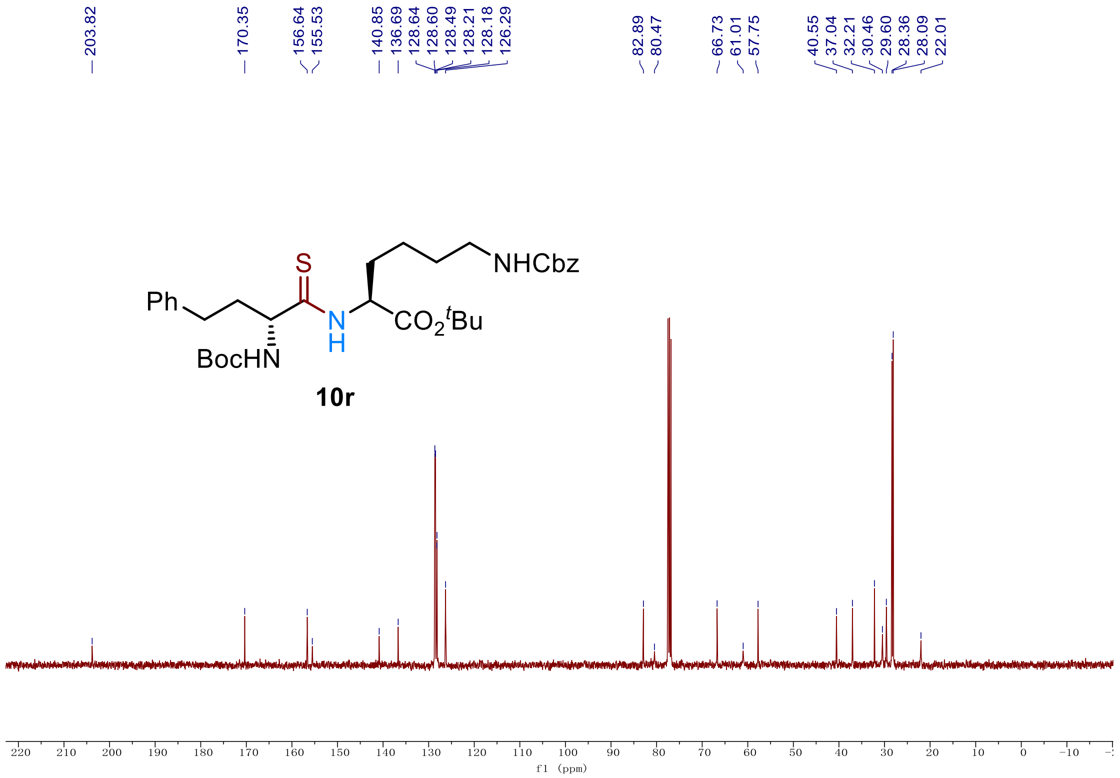

Supplementary Fig. S118 ^{13}C NMR spectrum of compound **10r** (101 MHz, $CDCl_3$)

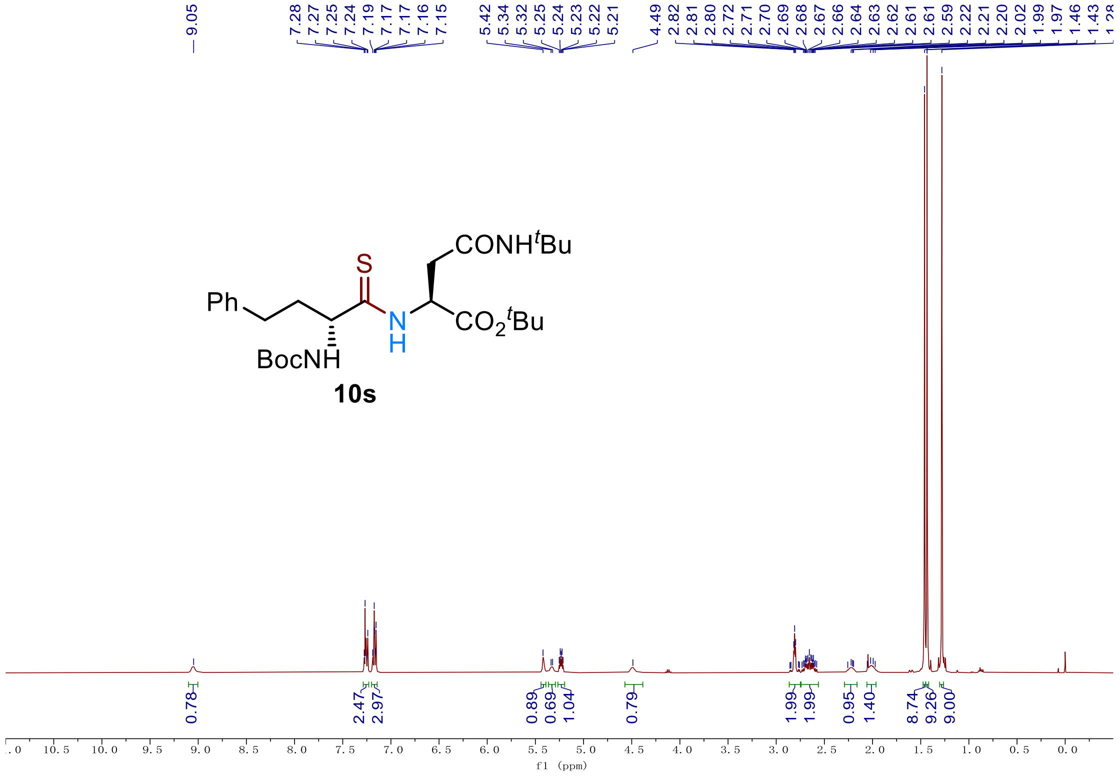

Supplementary Fig. S119 ^{1}H NMR spectrum of compound **10s** (400 MHz, $CDCl_3$)

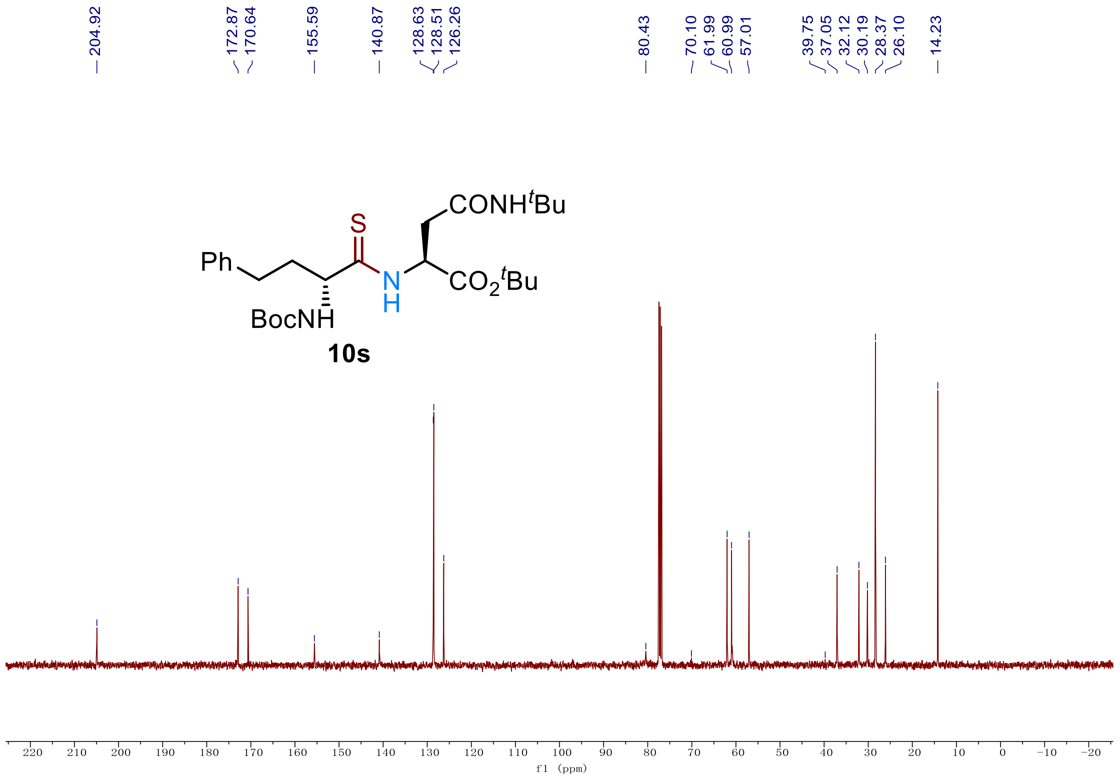

Supplementary Fig. S120 ^{13}C NMR spectrum of compound **10s** (101 MHz, $CDCl_3$)

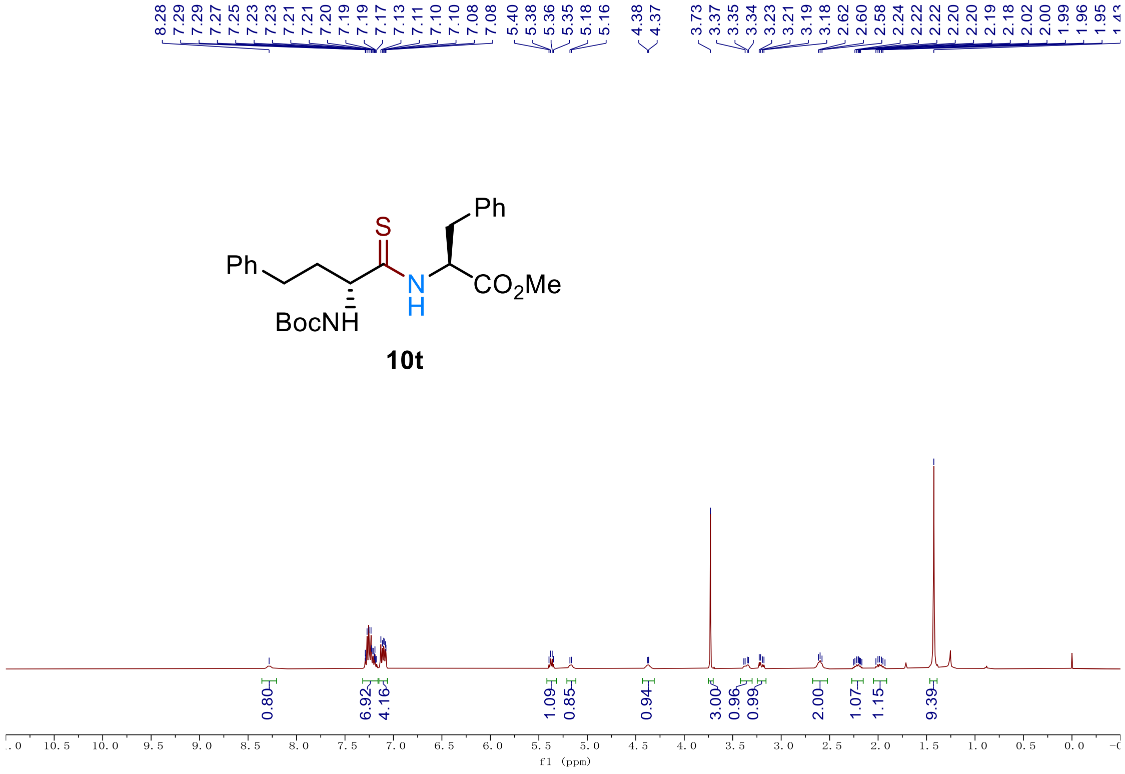

Supplementary Fig. S121 ^{1}H NMR spectrum of compound **10t** (400 MHz, $CDCl_3$)

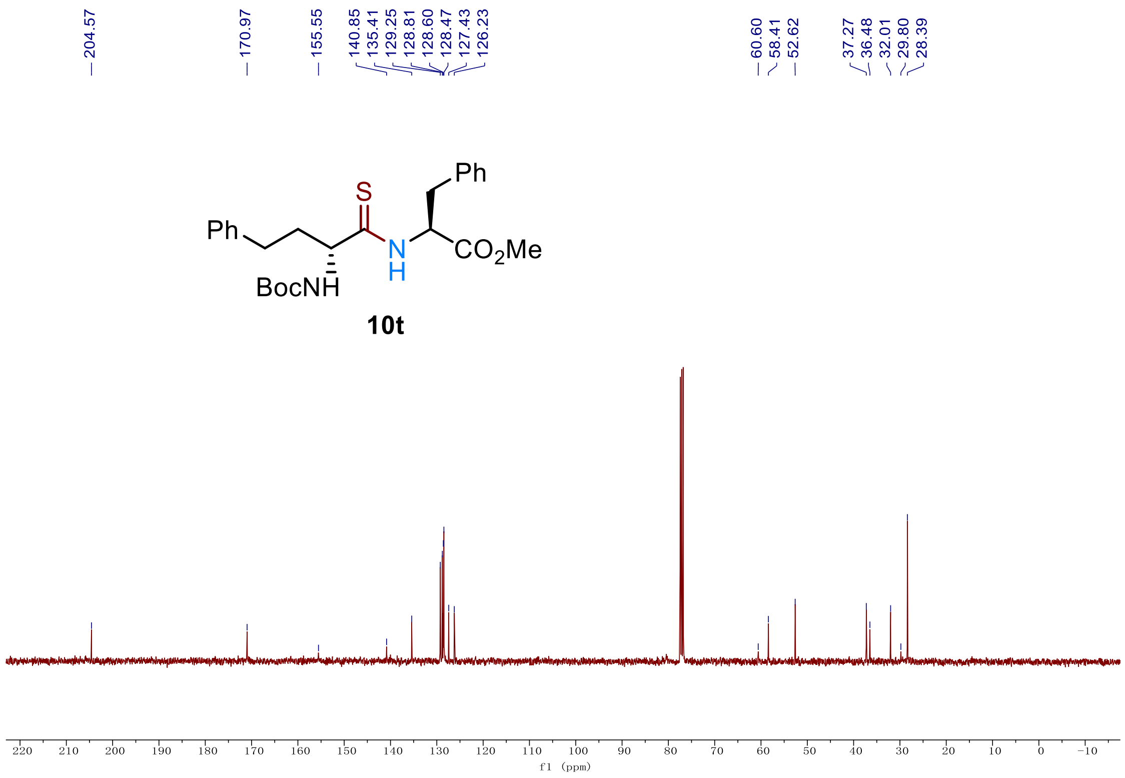

Supplementary Fig. S122 ^{13}C NMR spectrum of compound **10t** (101 MHz, $CDCl_3$)

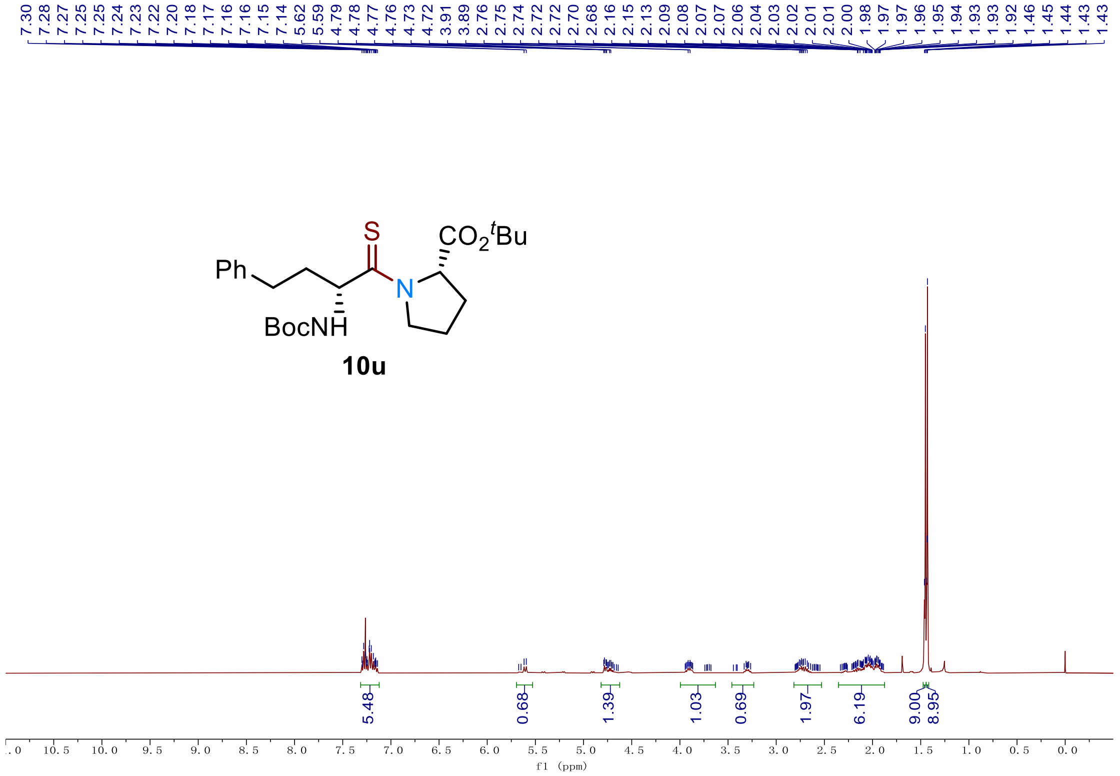

Supplementary Fig. S123 ^{1}H NMR spectrum of compound **10u** (400 MHz, $CDCl_3$)

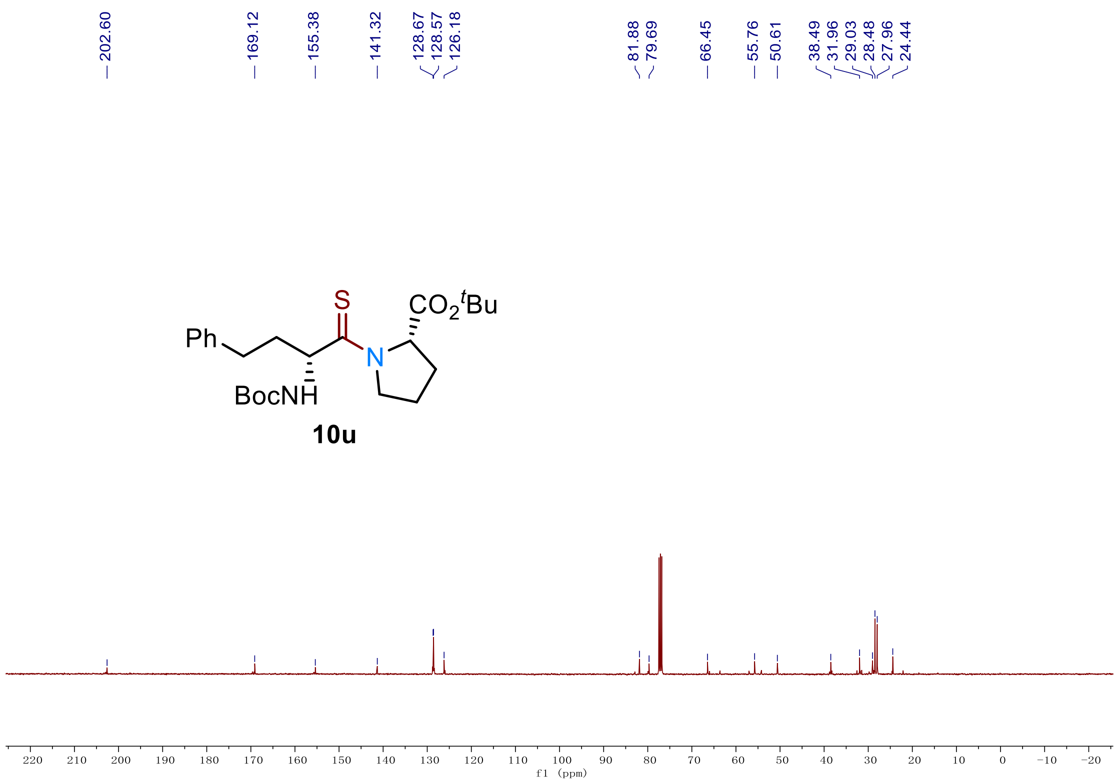

Supplementary Fig. S124 ^{13}C NMR spectrum of compound **10u** (101 MHz, $CDCl_3$)

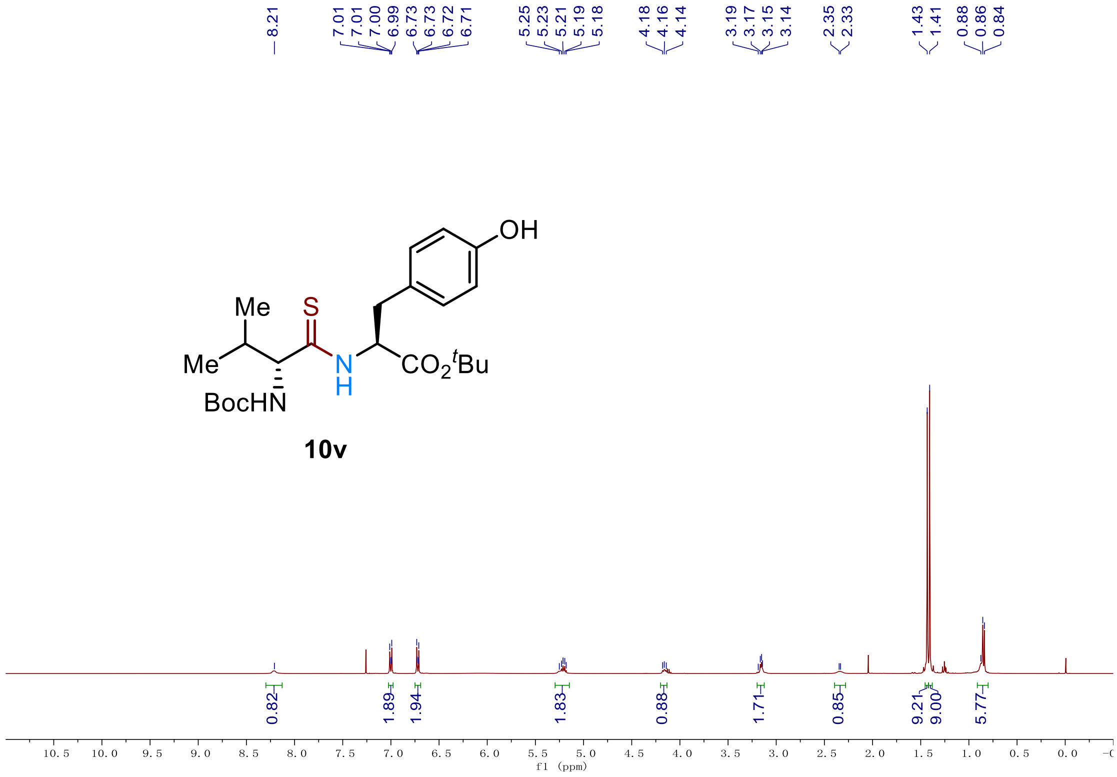

Supplementary Fig. S125 ^{1}H NMR spectrum of compound **10v** (400 MHz, $CDCl_3$)

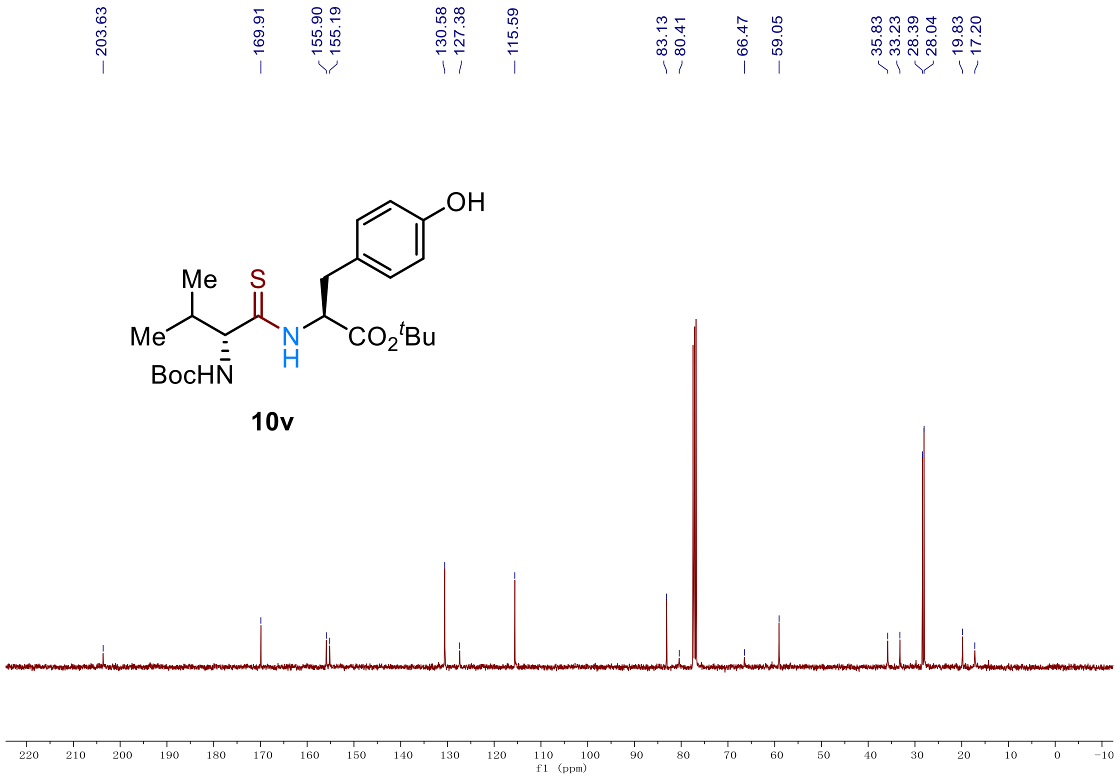

Supplementary Fig. S126 ^{13}C NMR spectrum of compound **10v** (101 MHz, $CDCl_3$)

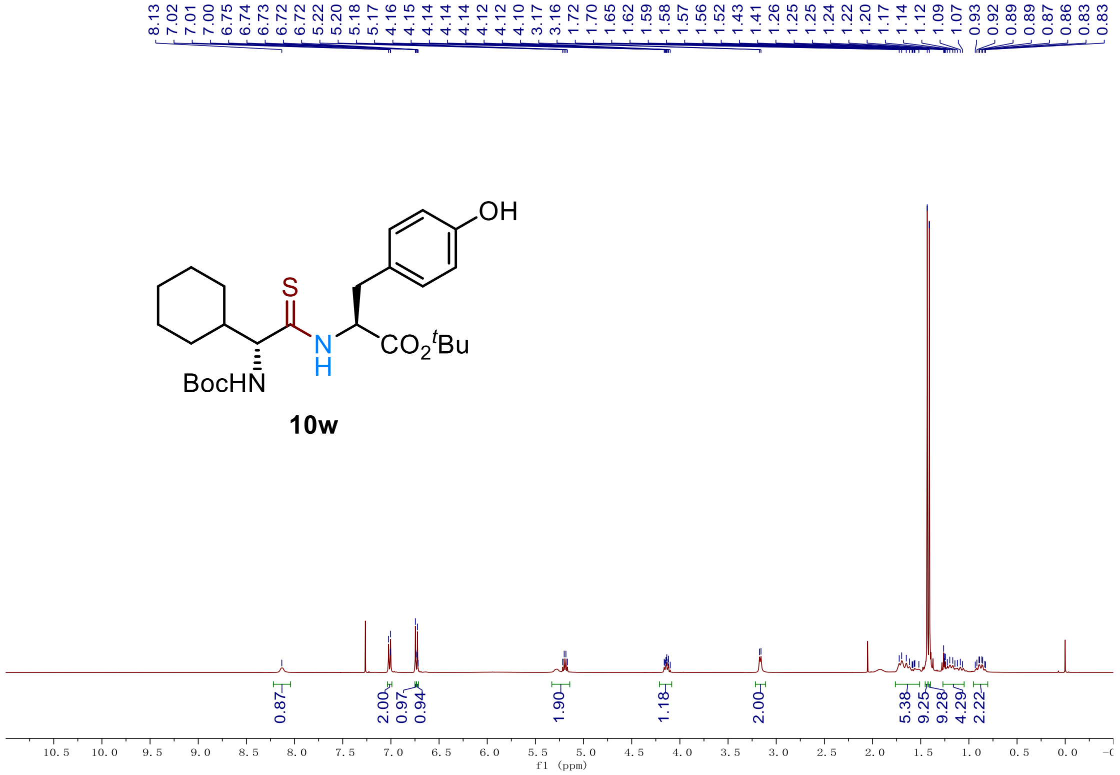

Supplementary Fig. S127 ^{1}H NMR spectrum of compound **10w** (400 MHz, $CDCl_3$)

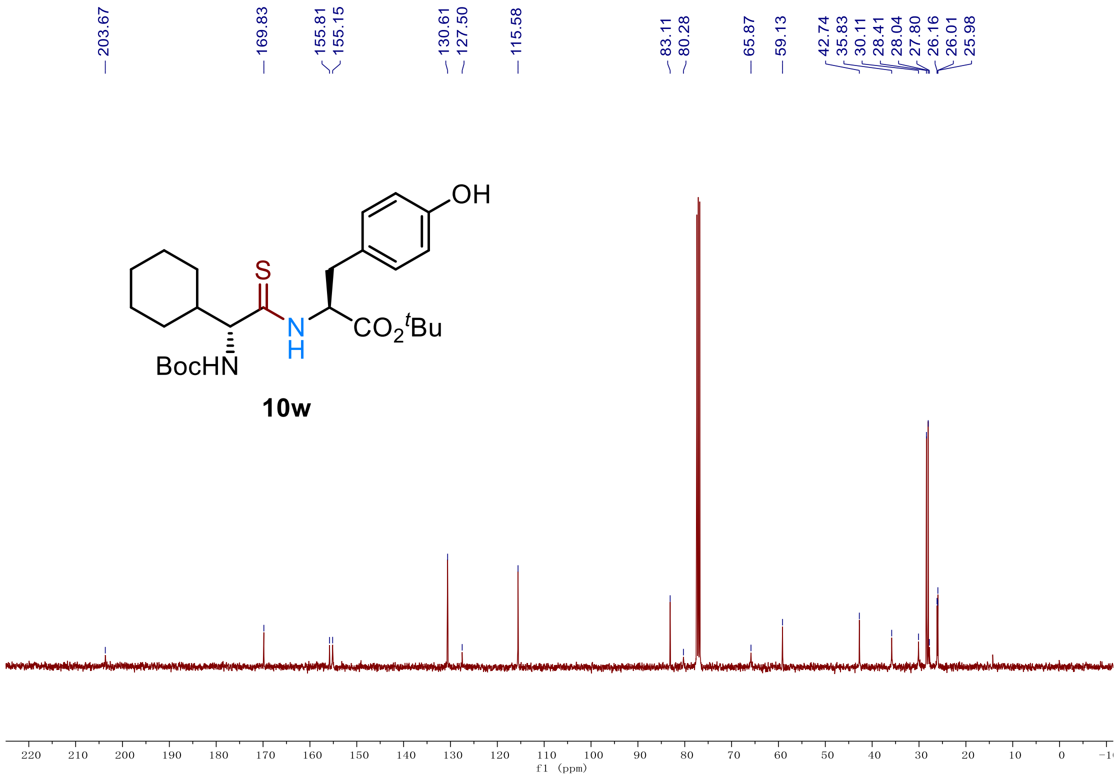

Supplementary Fig. S128 ^{13}C NMR spectrum of compound **10w** (101 MHz, $CDCl_3$)

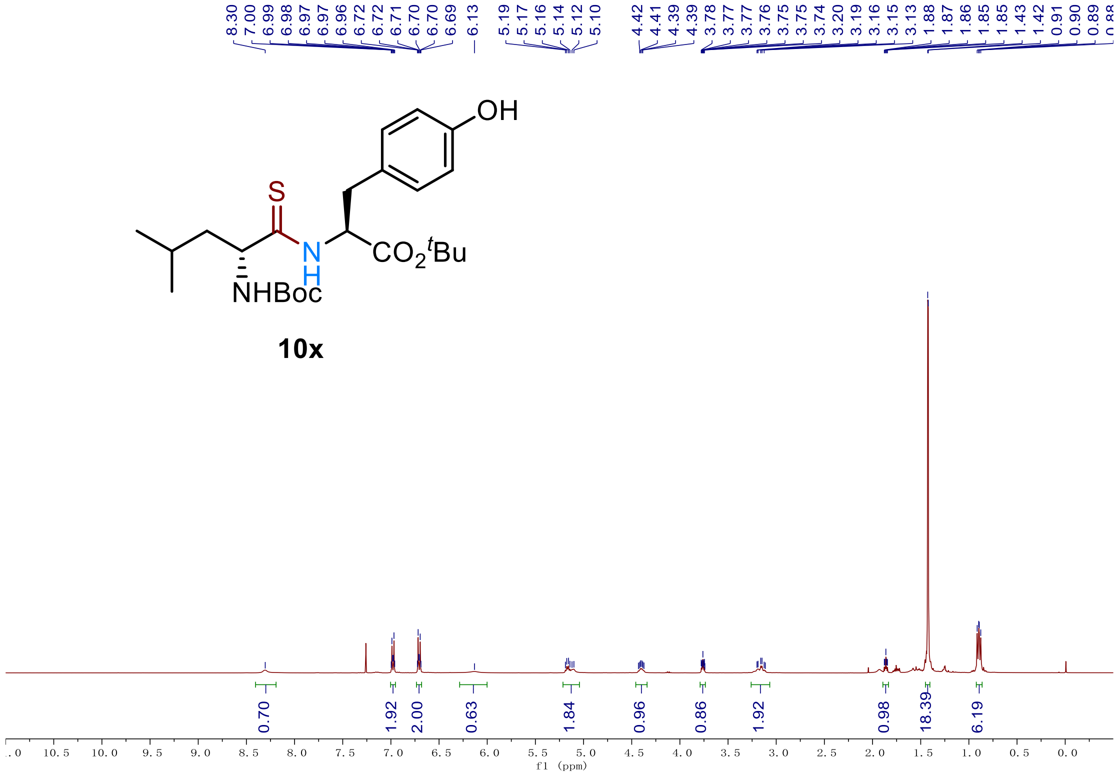

Supplementary Fig. S129 ^{1}H NMR spectrum of compound **10x** (400 MHz, $CDCl_3$)

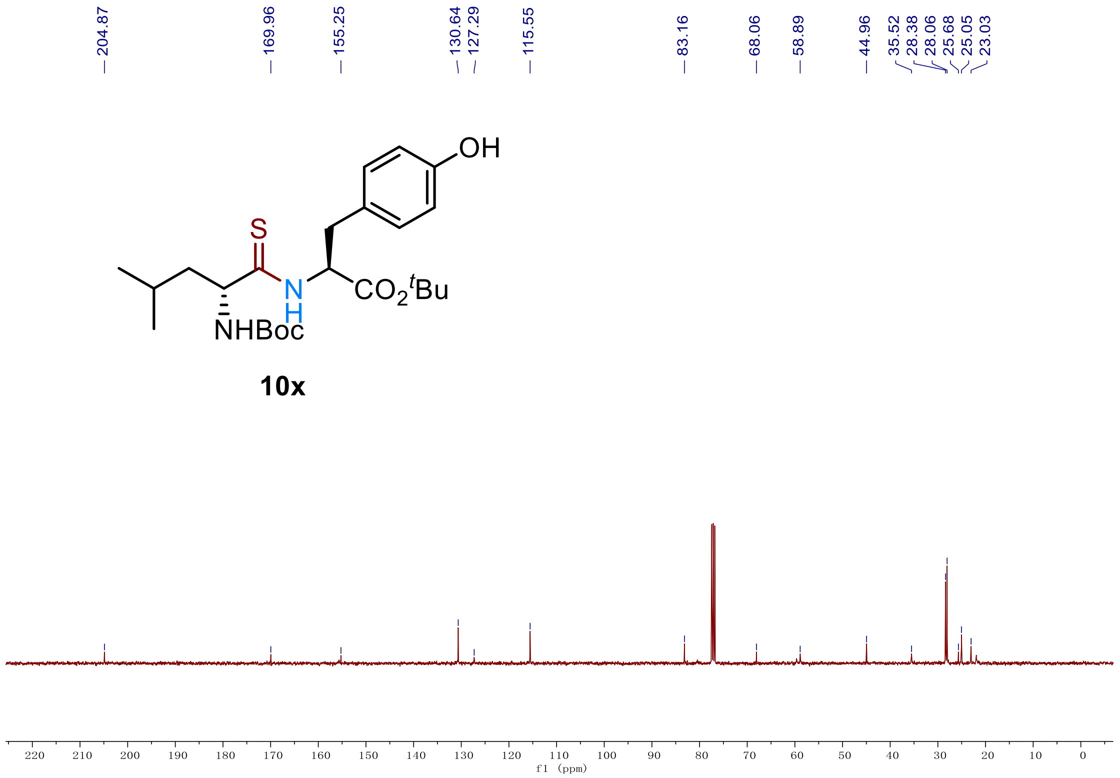

Supplementary Fig. S130 ^{13}C NMR spectrum of compound **10x** (101 MHz, $CDCl_3$)

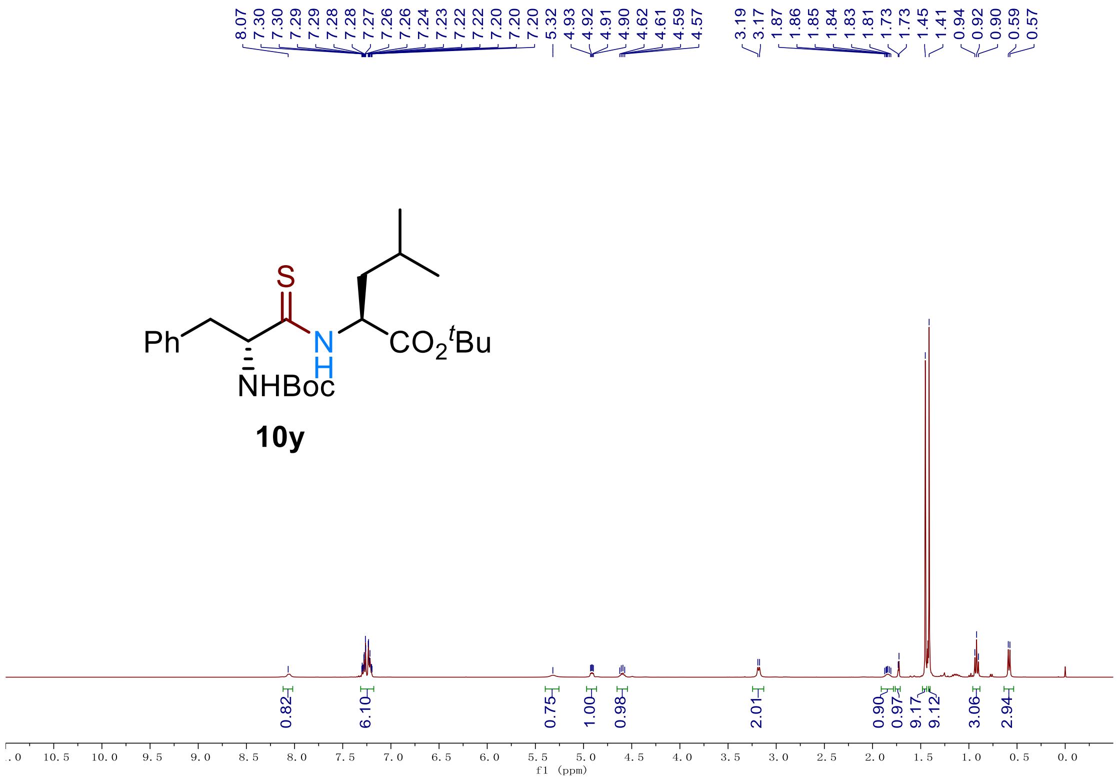

Supplementary Fig. S131 ^{1}H NMR spectrum of compound **10y** (400 MHz, $CDCl_3$)

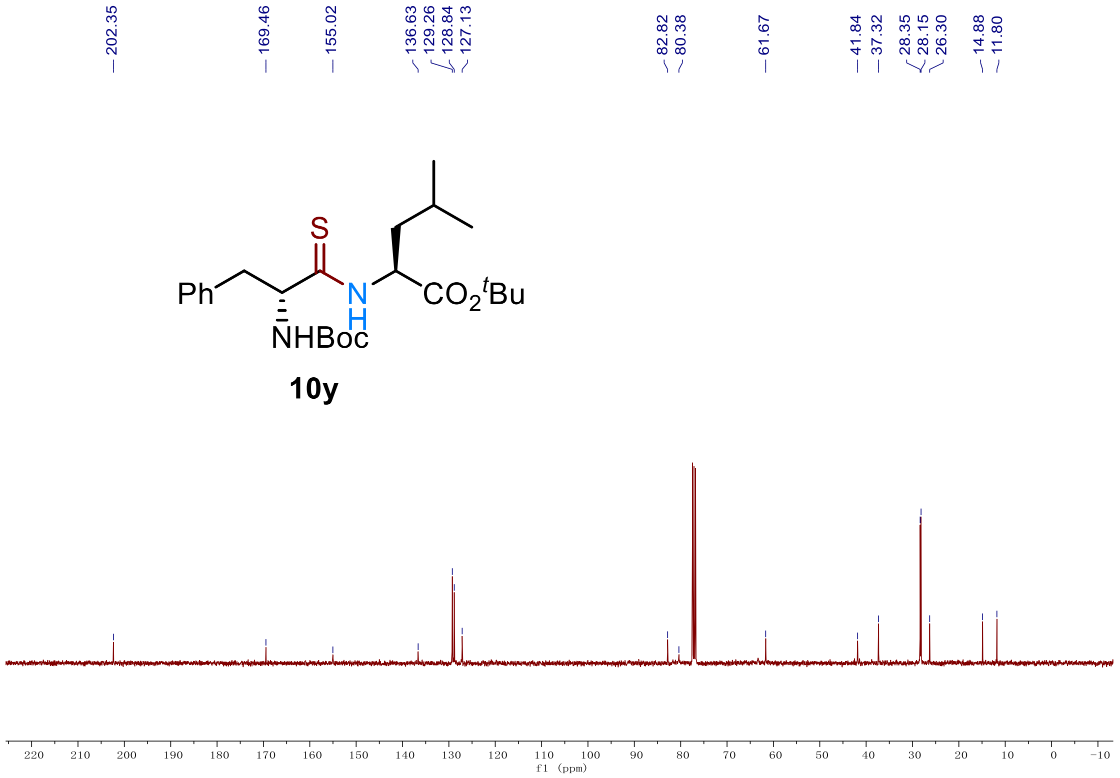

Supplementary Fig. S132 ^{13}C NMR spectrum of compound **10y** (101 MHz, $CDCl_3$)

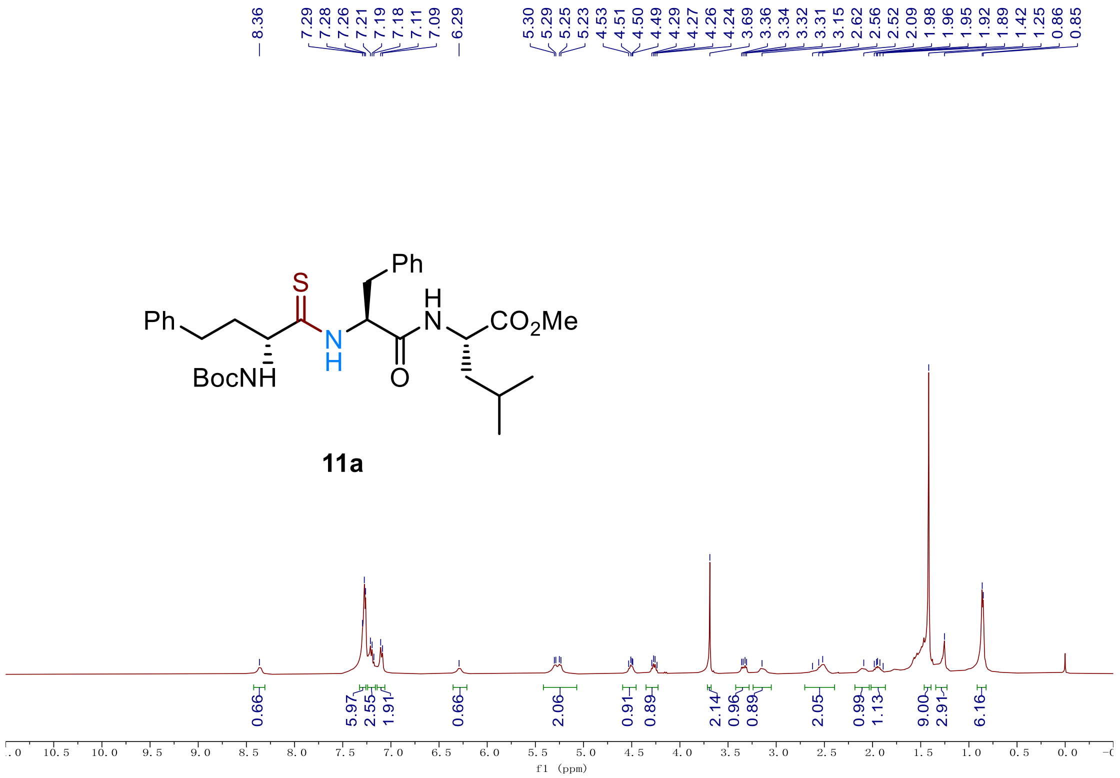

Supplementary Fig. S133 ^{1}H NMR spectrum of compound **11a** (400 MHz, $CDCl_3$)

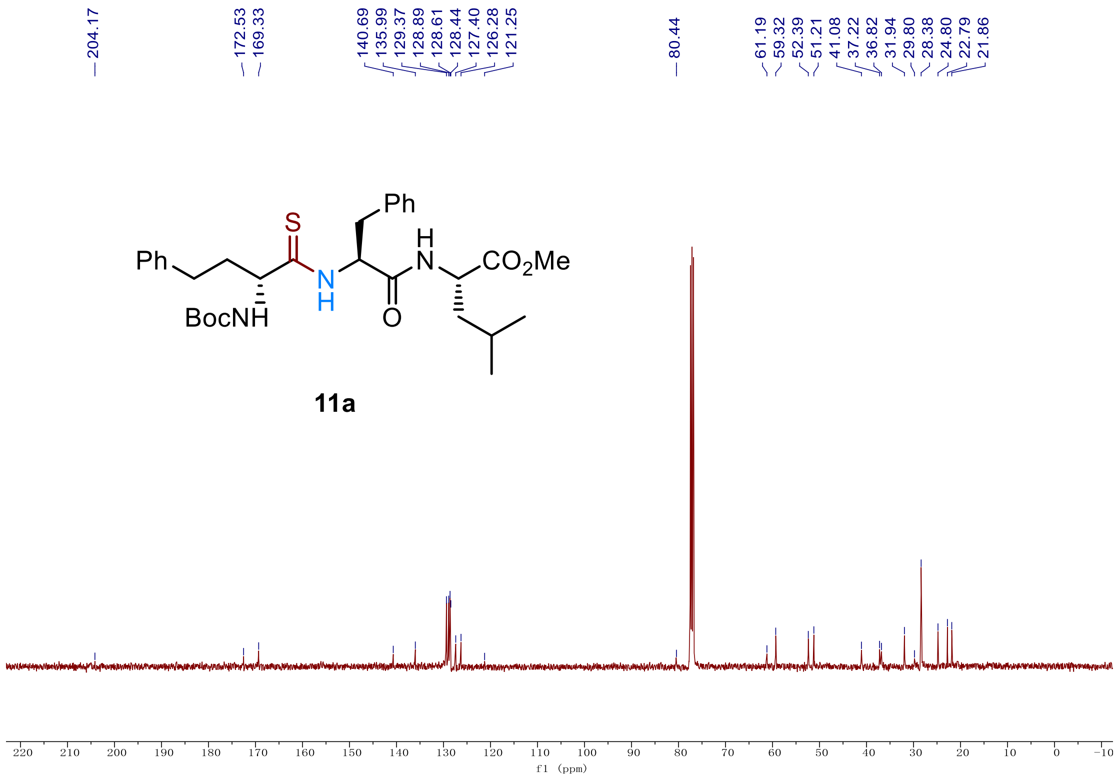

Supplementary Fig. S134 ^{13}C NMR spectrum of compound **11a** (101 MHz, $CDCl_3$)

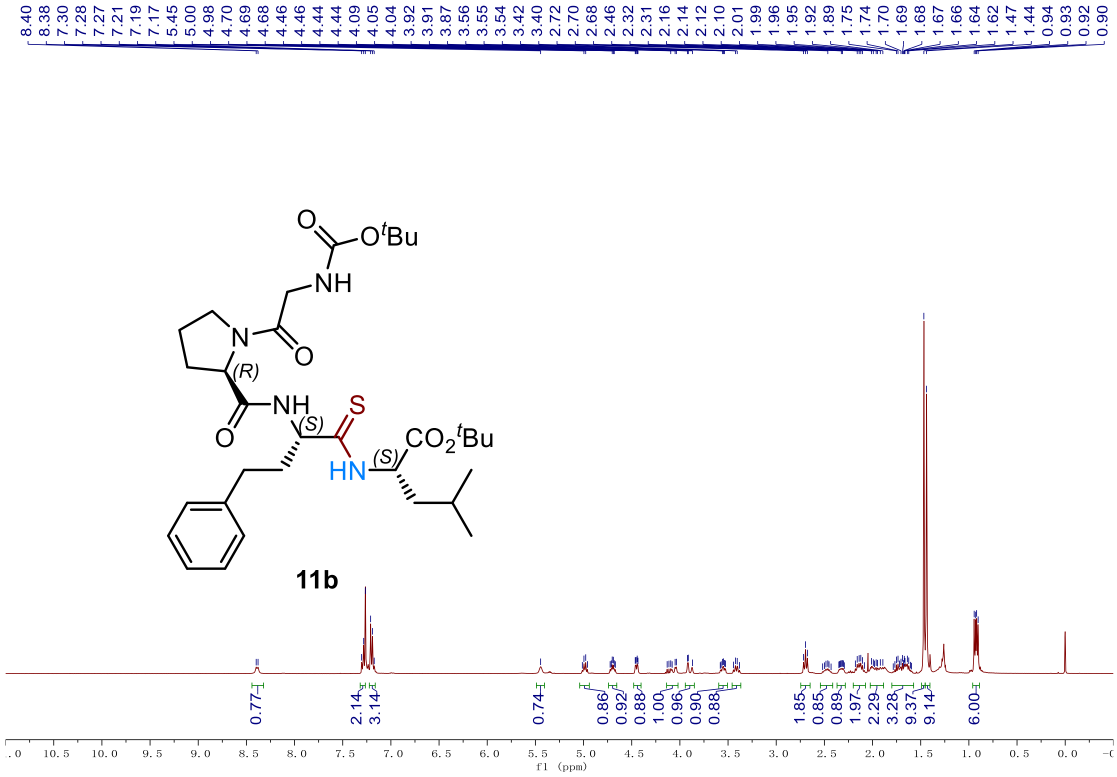

Supplementary Fig. S135 ^{1}H NMR spectrum of compound **11b** (400 MHz, $CDCl_3$)

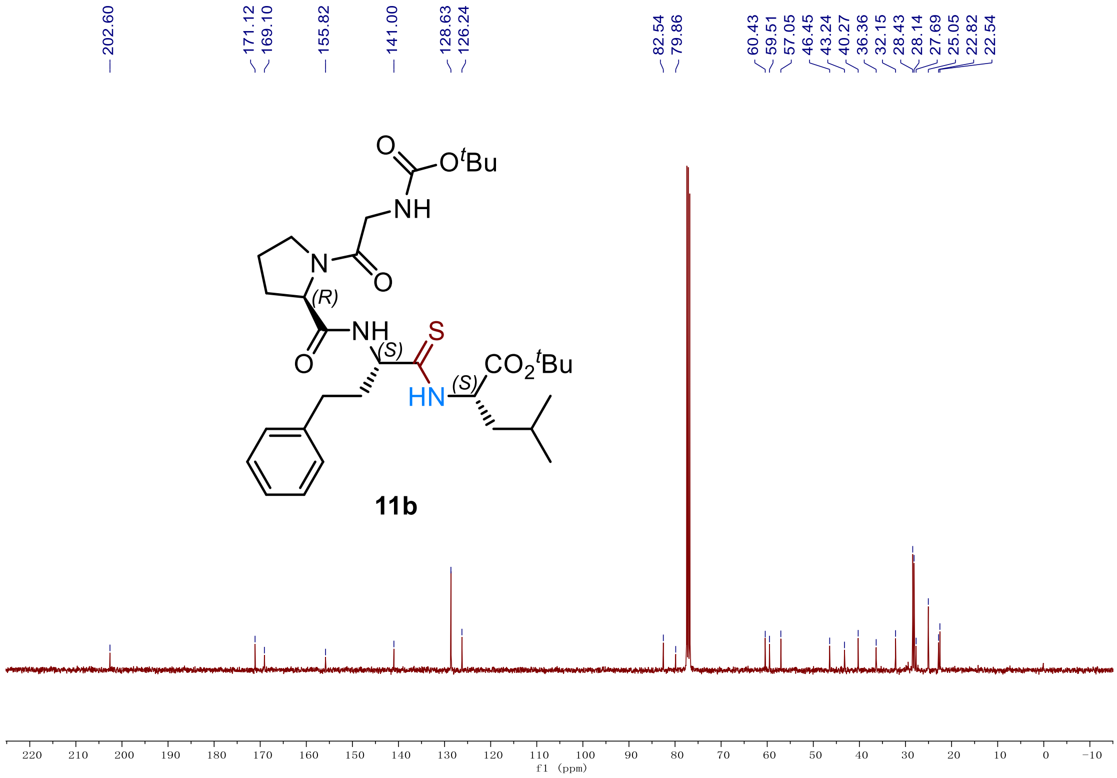

Supplementary Fig. S136 ^{13}C NMR spectrum of compound **11b** (101 MHz, $CDCl_3$)

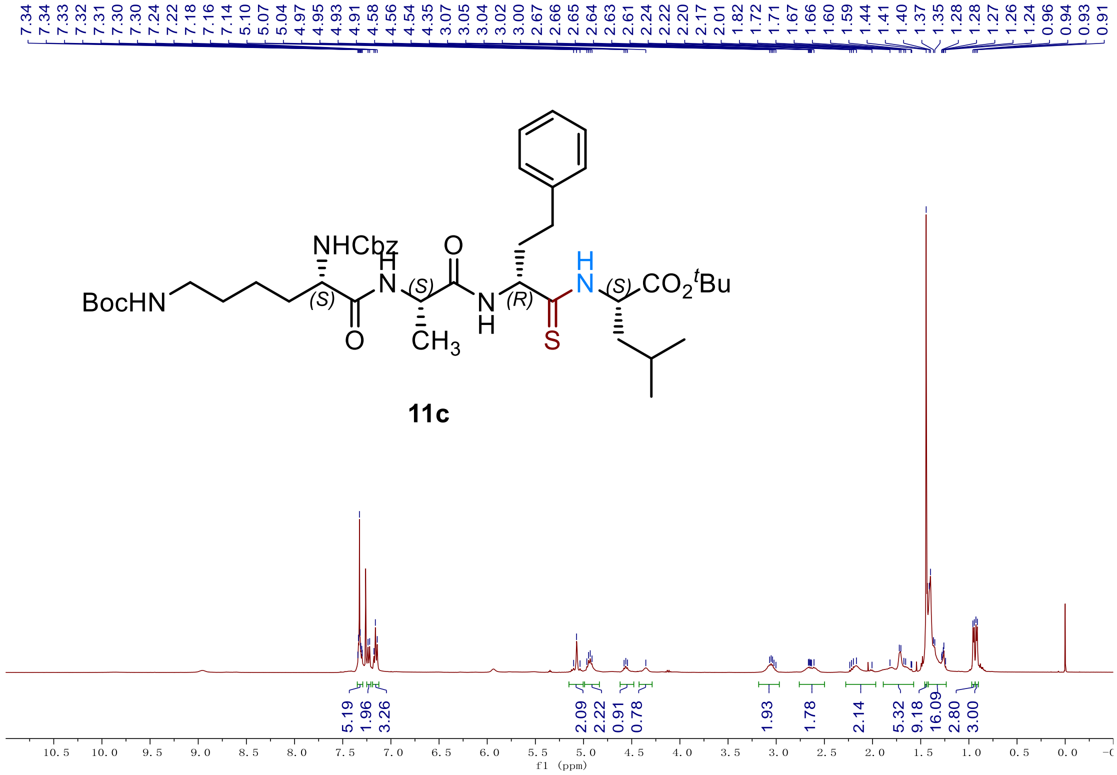

Supplementary Fig. S137 ^{1}H NMR spectrum of compound **11c** (400 MHz, $CDCl_3$)

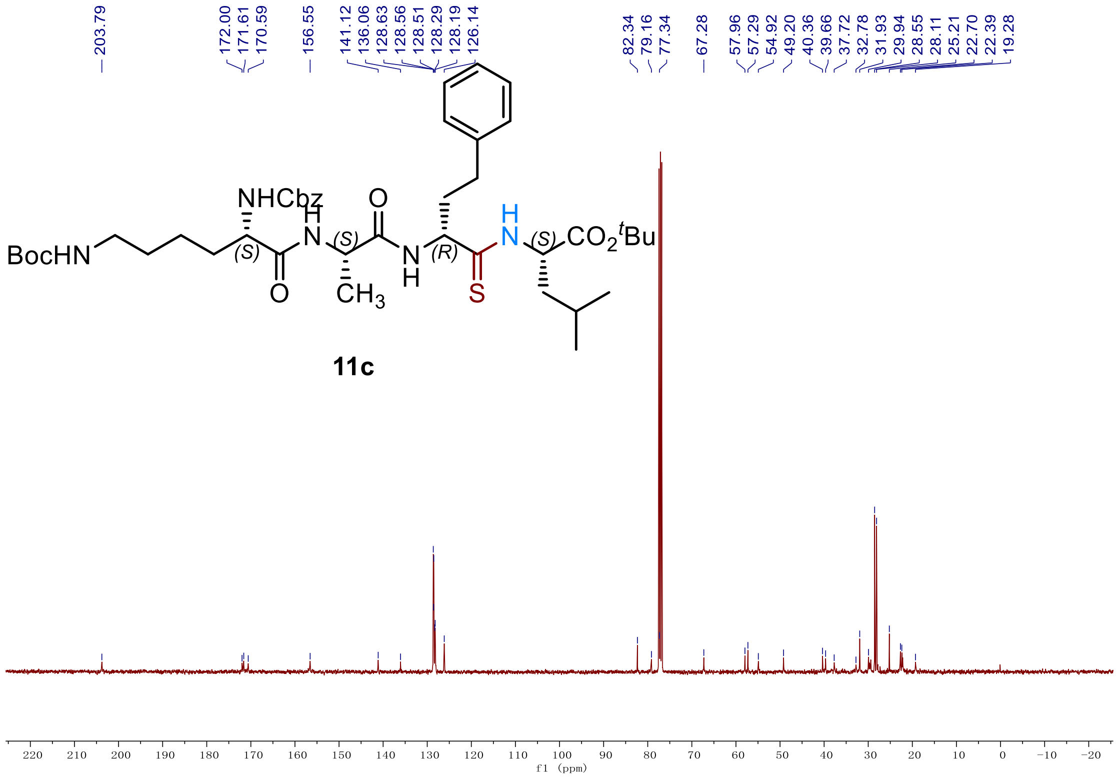

Supplementary Fig. S138 ^{13}C NMR spectrum of compound **11c** (101 MHz, $CDCl_3$)

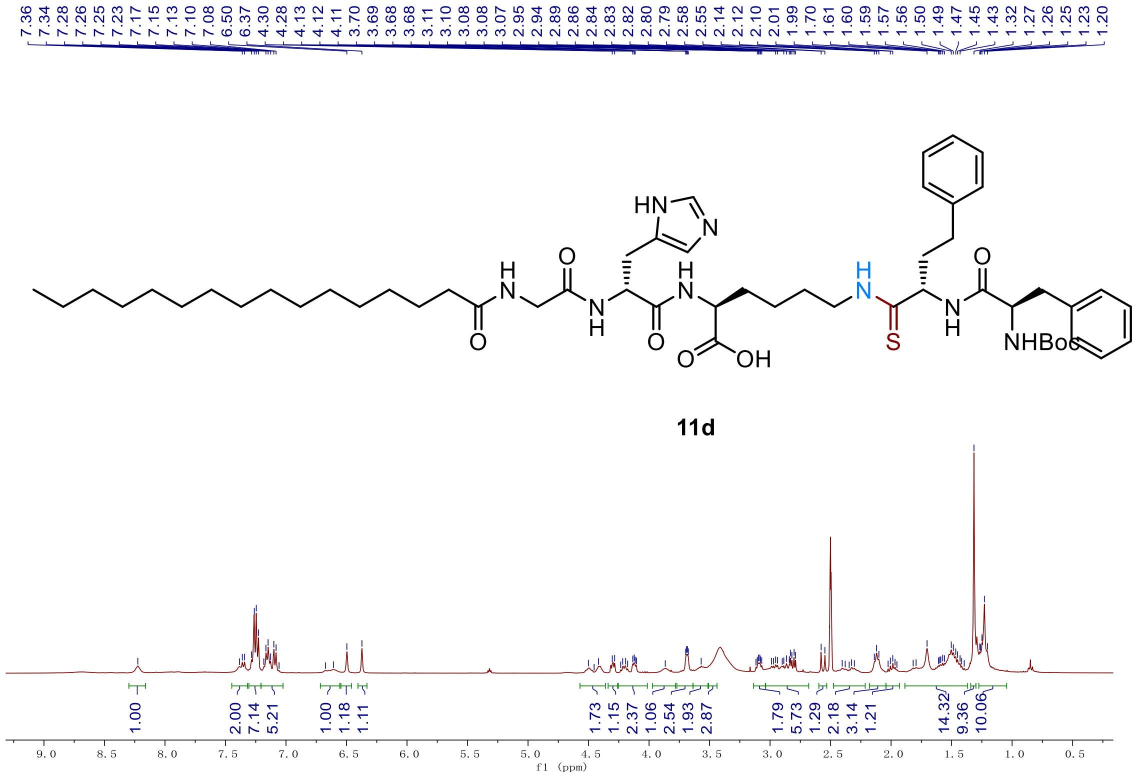

Supplementary Fig. S139 ^{1}H NMR spectrum of compound **11d** (400 MHz, DMSO-d_6)

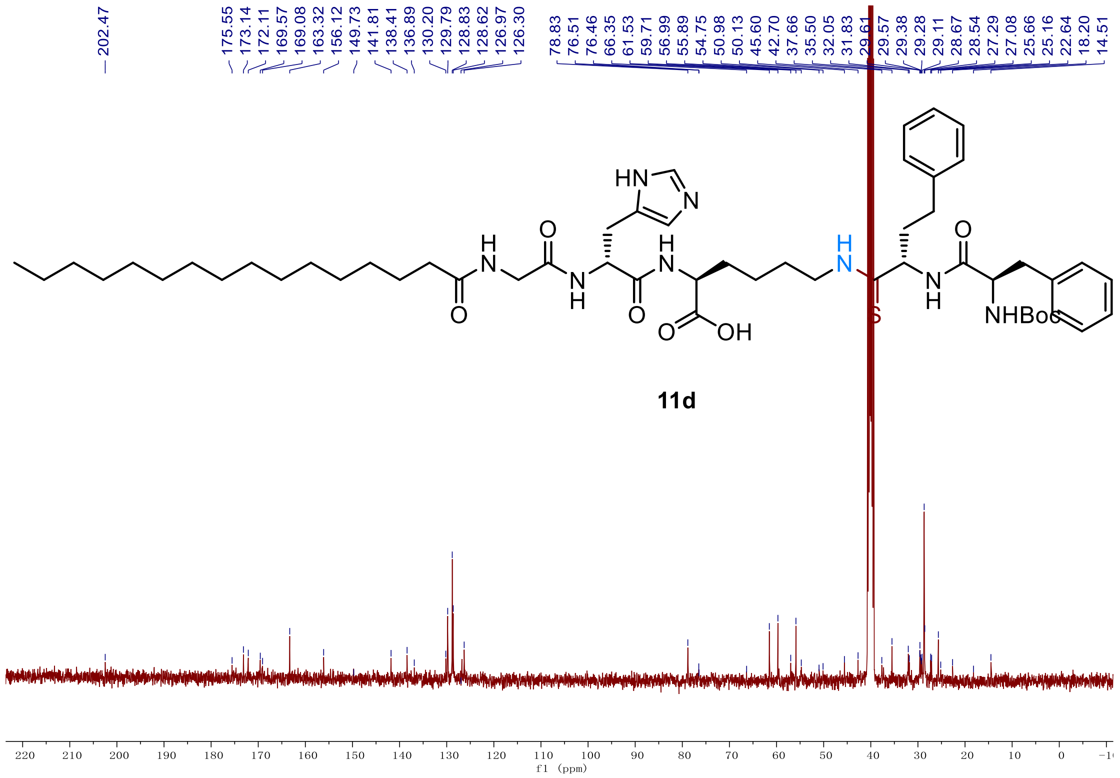

Supplementary Fig. S140 ^{13}C NMR spectrum of compound **11d** (101 MHz, DMSO-d_6)

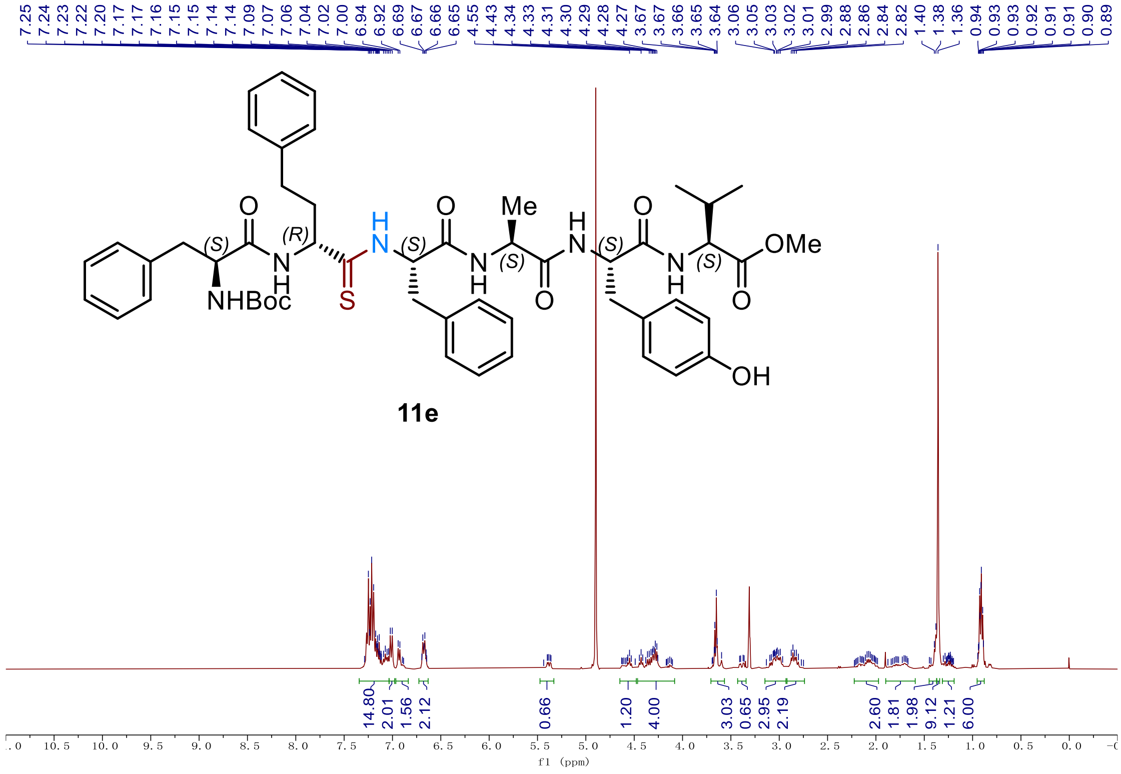

Supplementary Fig. S141 ^{1}H NMR spectrum of compound **11e** (400 MHz, MeOD-d_4)

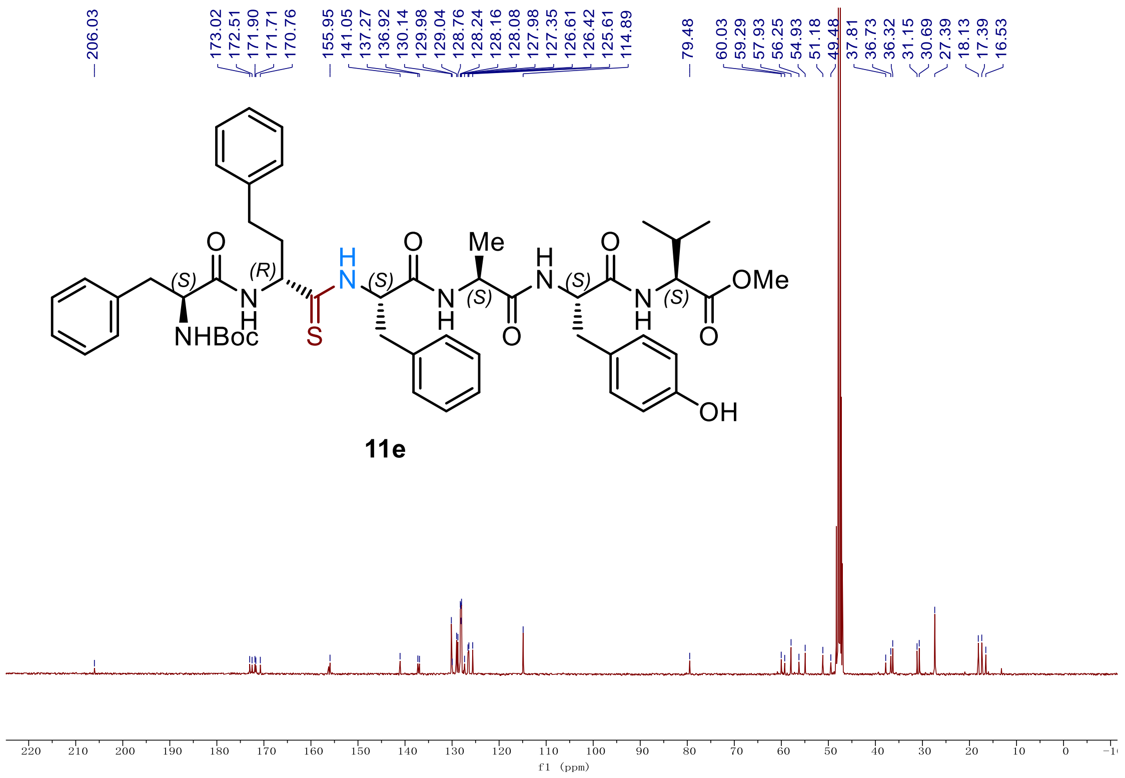

Supplementary Fig. S142 ^{13}C NMR spectrum of compound **11e** (101 MHz, MeOD-d_4)

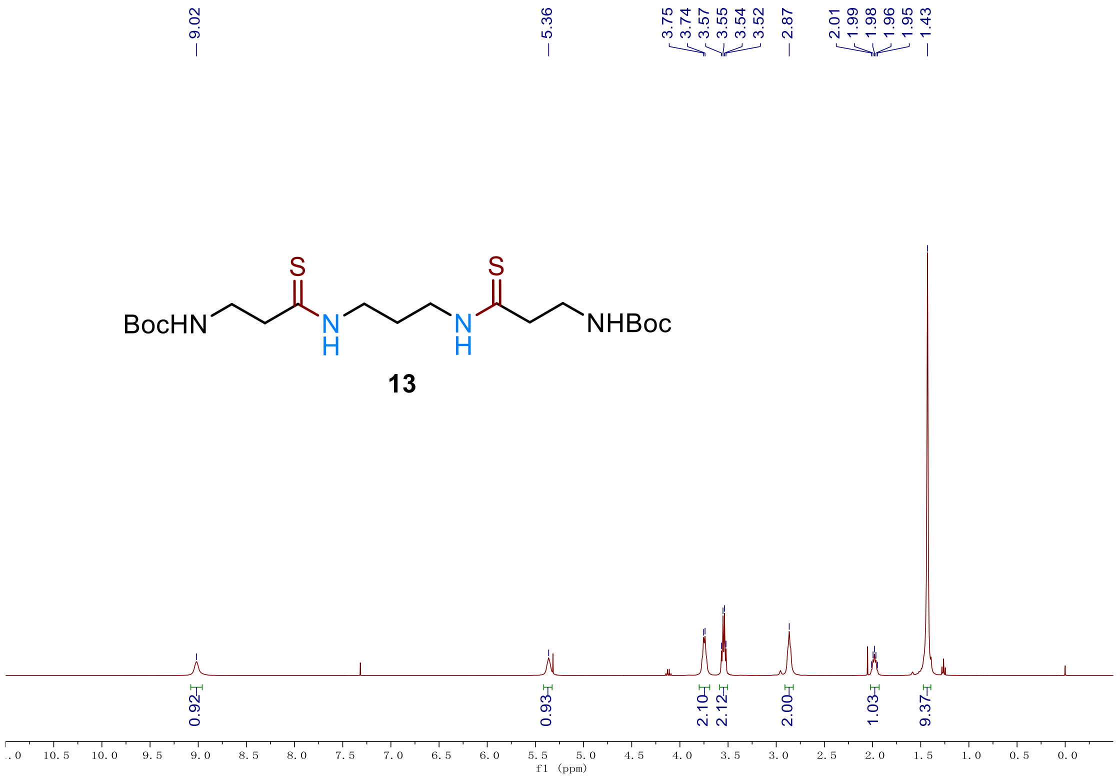

Supplementary Fig. S143 ^{1}H NMR spectrum of compound **13** (400 MHz, $CDCl_3$)

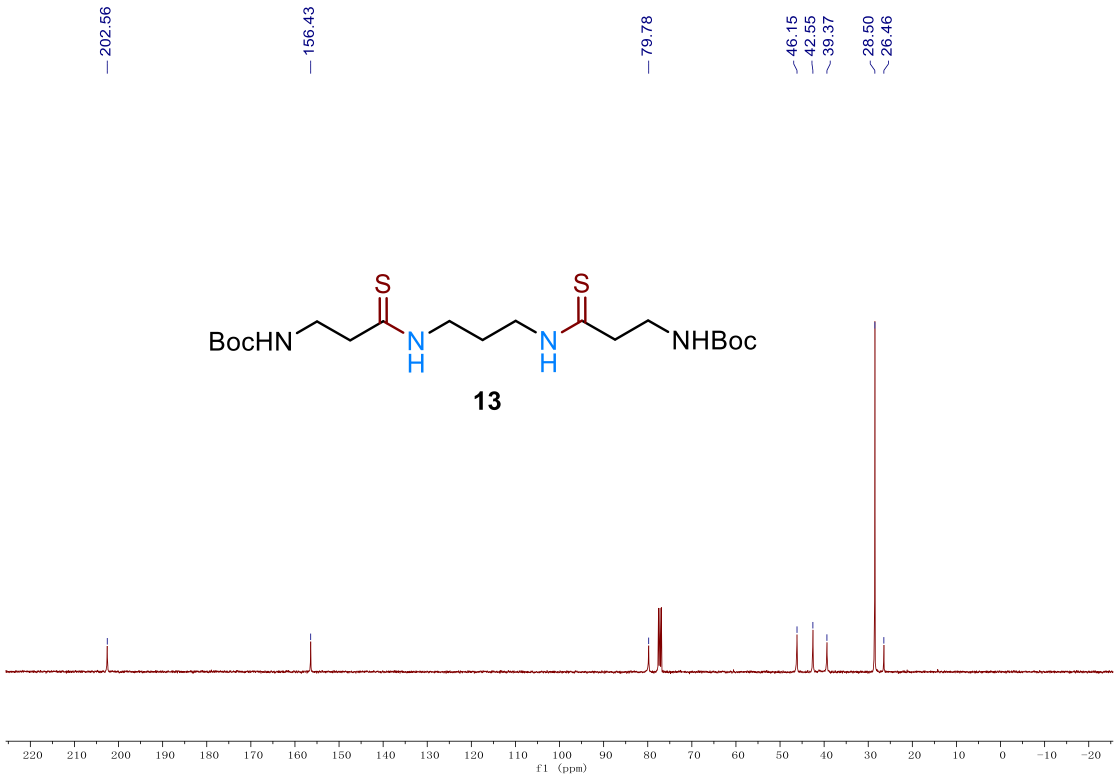

Supplementary Fig. S144 ^{13}C NMR spectrum of compound **13** (101 MHz, $CDCl_3$)

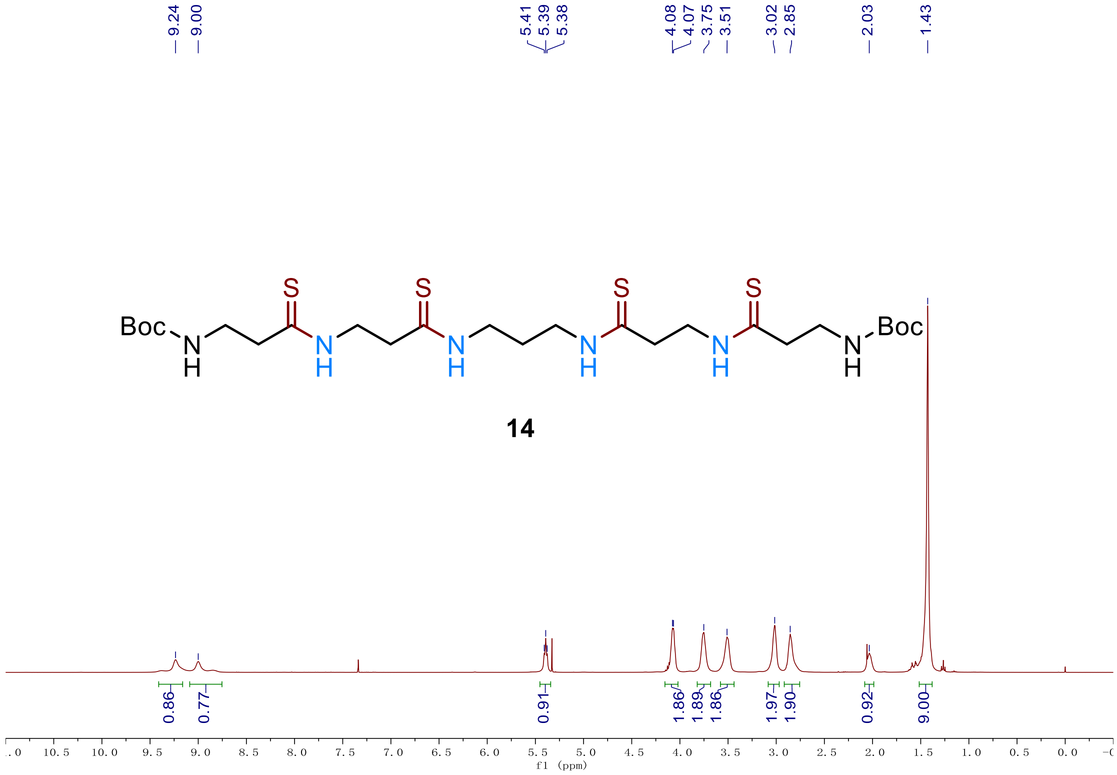

Supplementary Fig. S145 ^{1}H NMR spectrum of compound **14** (400 MHz, $CDCl_3$)

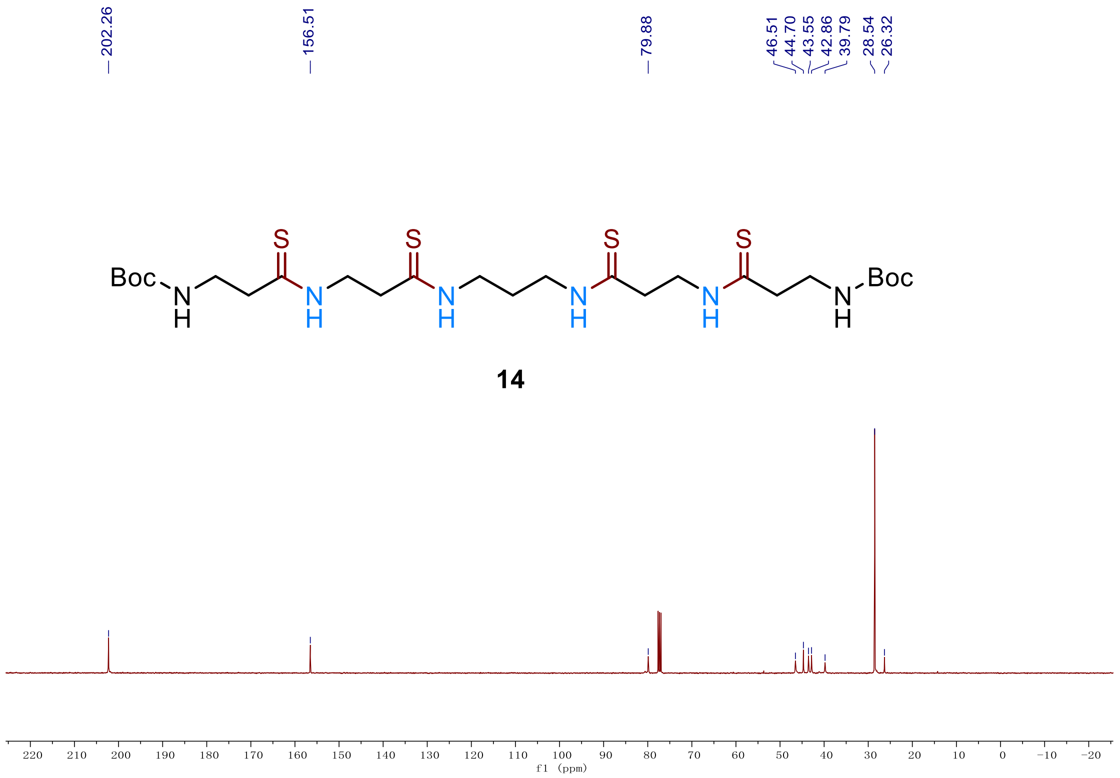

Supplementary Fig. S146 ^{13}C NMR spectrum of compound **14** (101 MHz, $CDCl_3$)

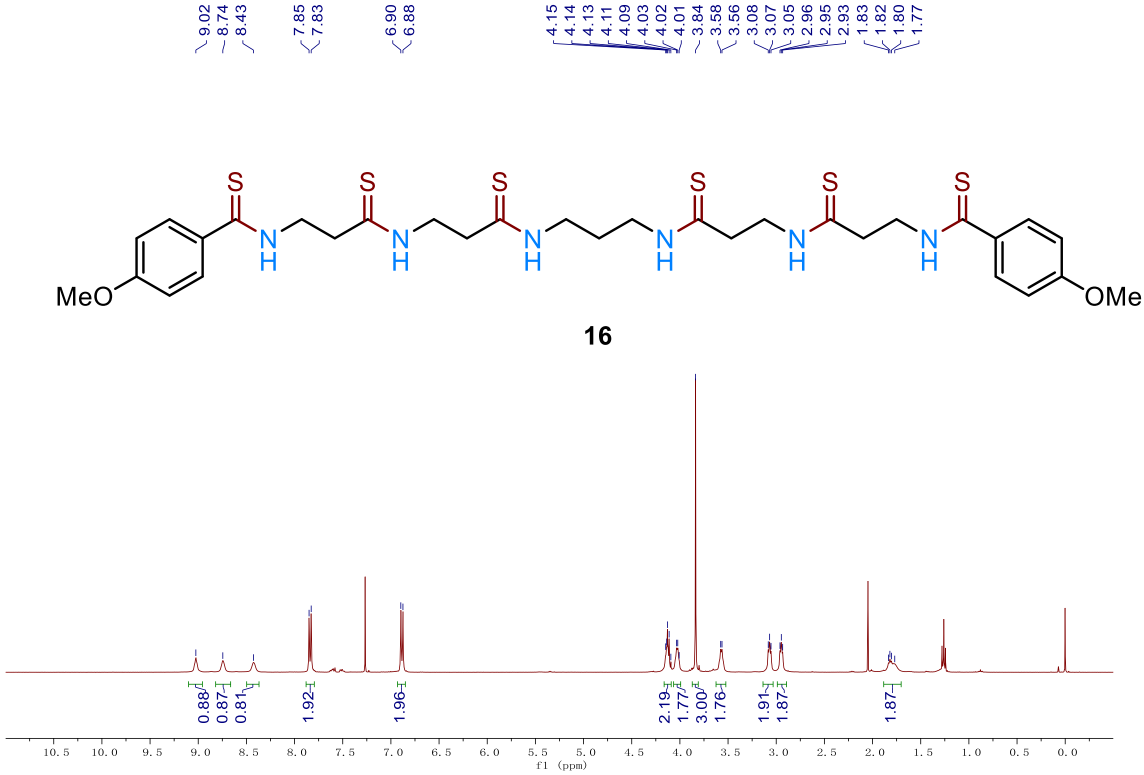

Supplementary Fig. S147 ^{1}H NMR spectrum of compound **16** (400 MHz, $CDCl_3$)

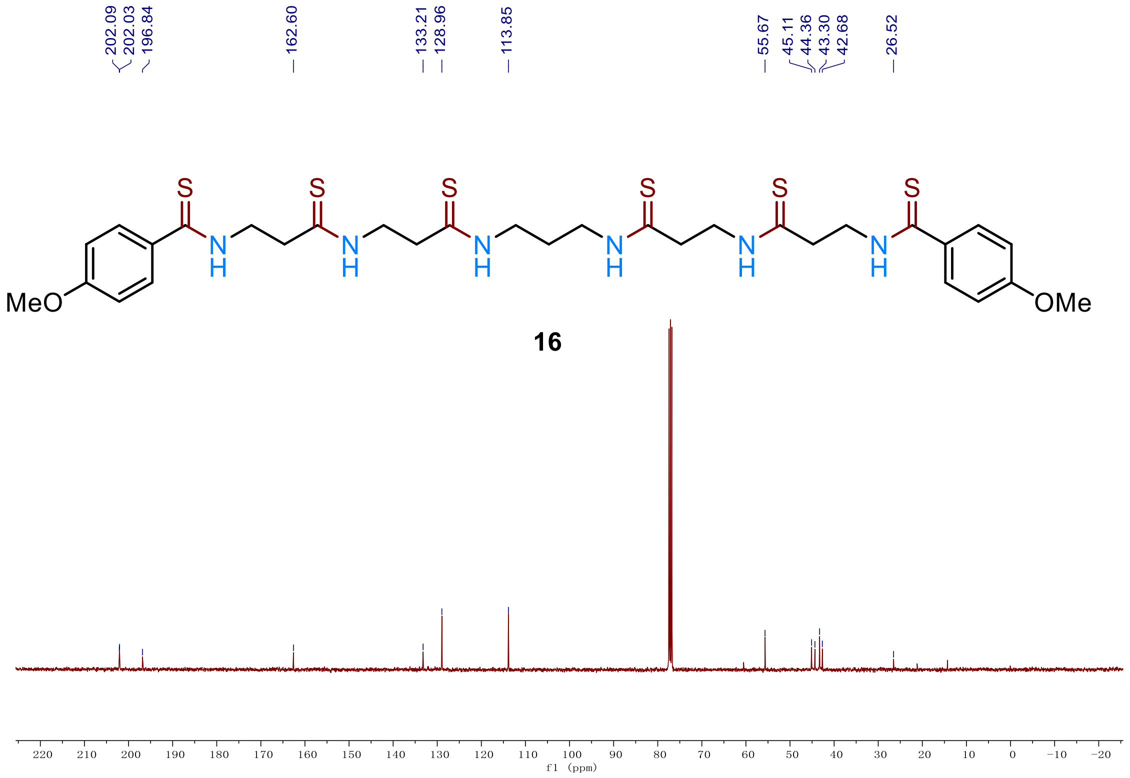

Supplementary Fig. S148 ^{13}C NMR spectrum of compound **16** (101 MHz, $CDCl_3$)

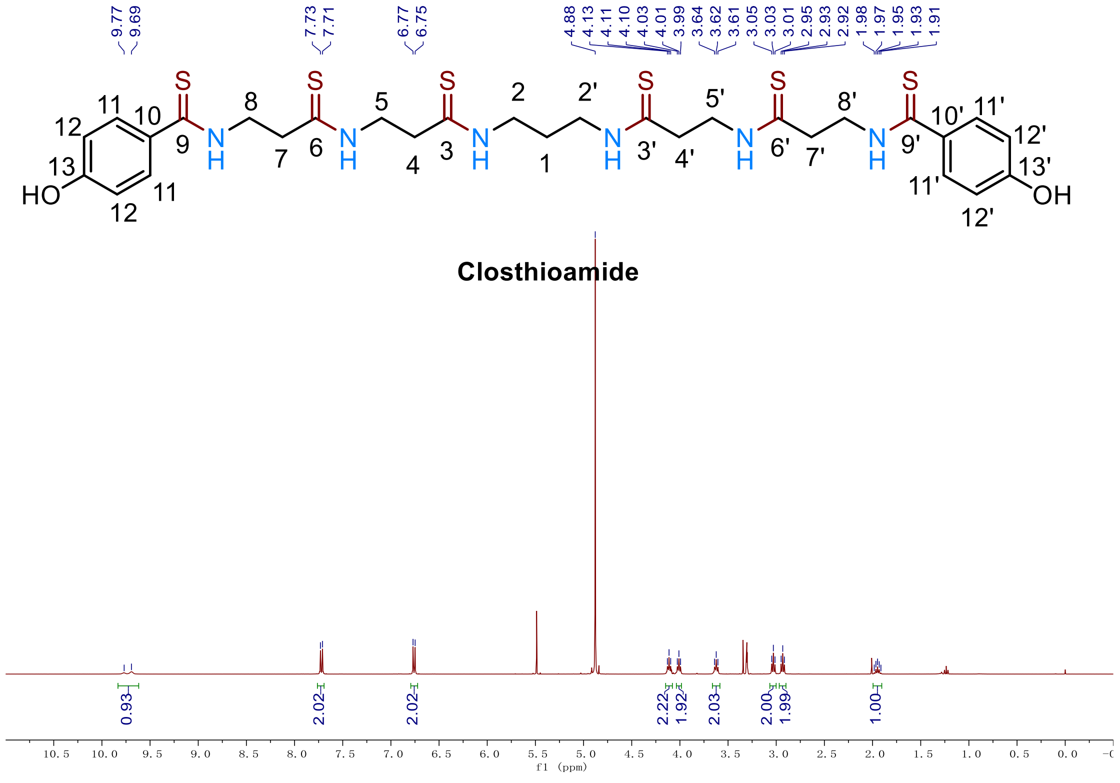

Supplementary Fig. S149 ^{1}H NMR spectrum of compound **Closthioamide** (400 MHz, MeOD-*d*$_4$)

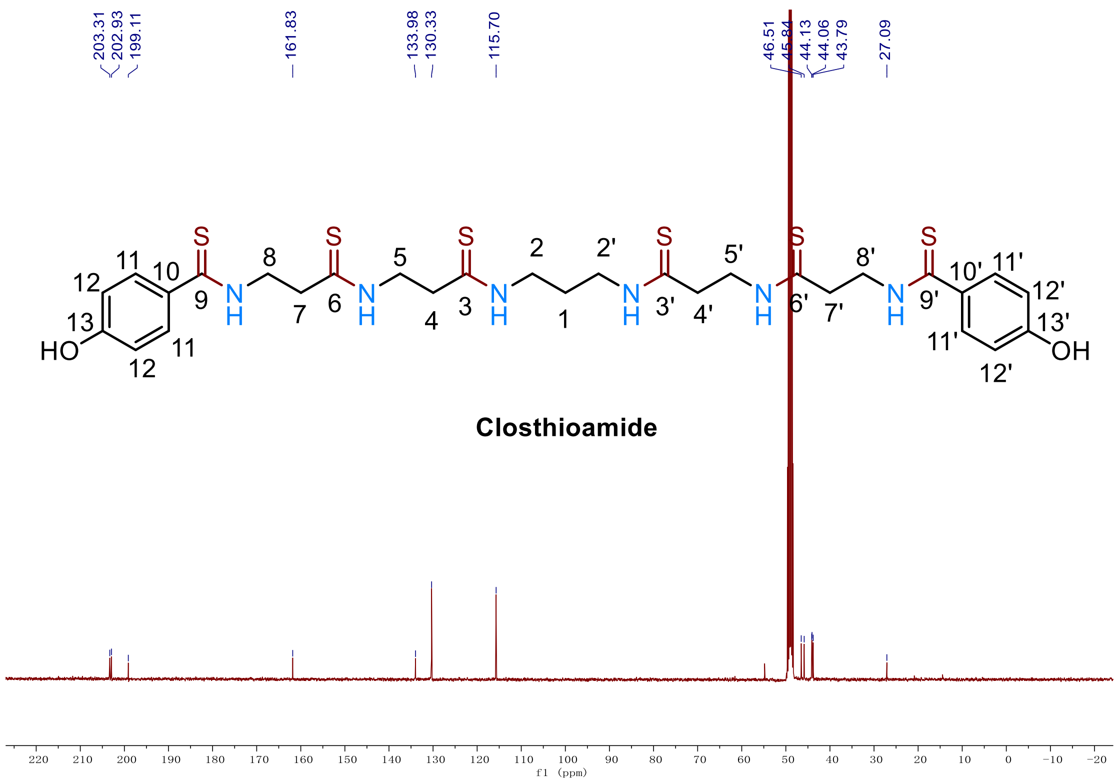

Supplementary Fig. S150 ^{13}C NMR spectrum of compound **Closthioamide** (101 MHz, MeOD-d_4)

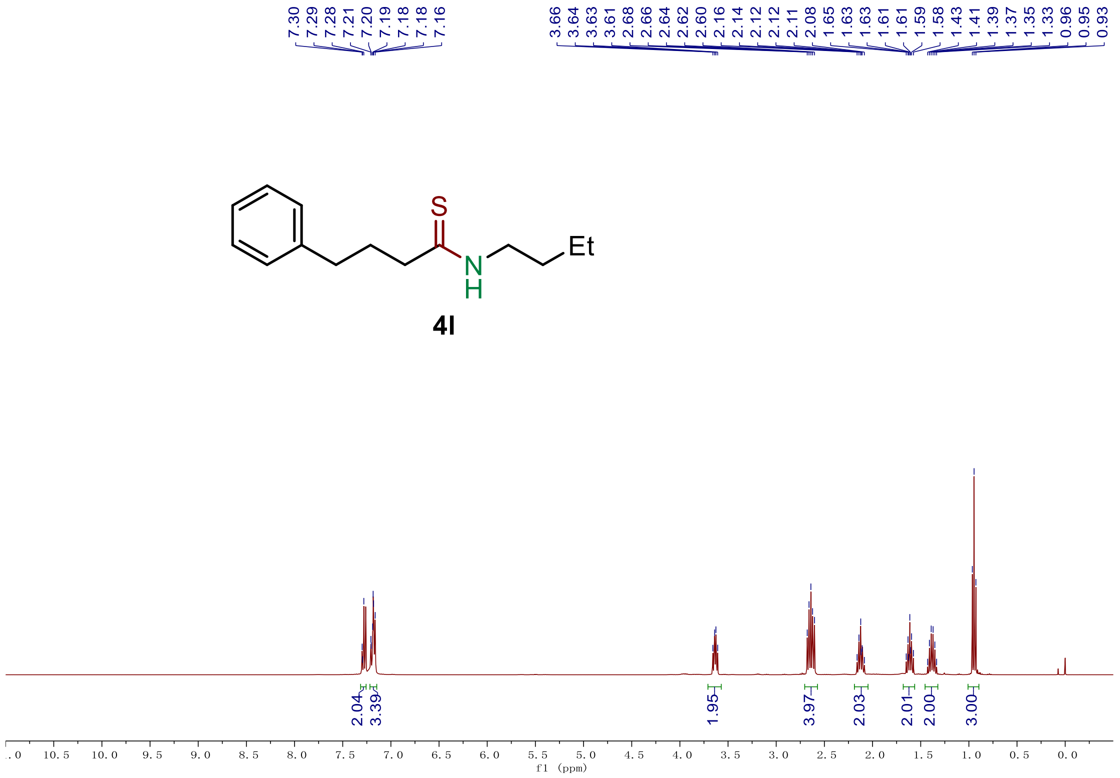

Supplementary Fig. S151 ^{1}H NMR spectrum of compound **4l** (400 MHz, $CDCl_3$)

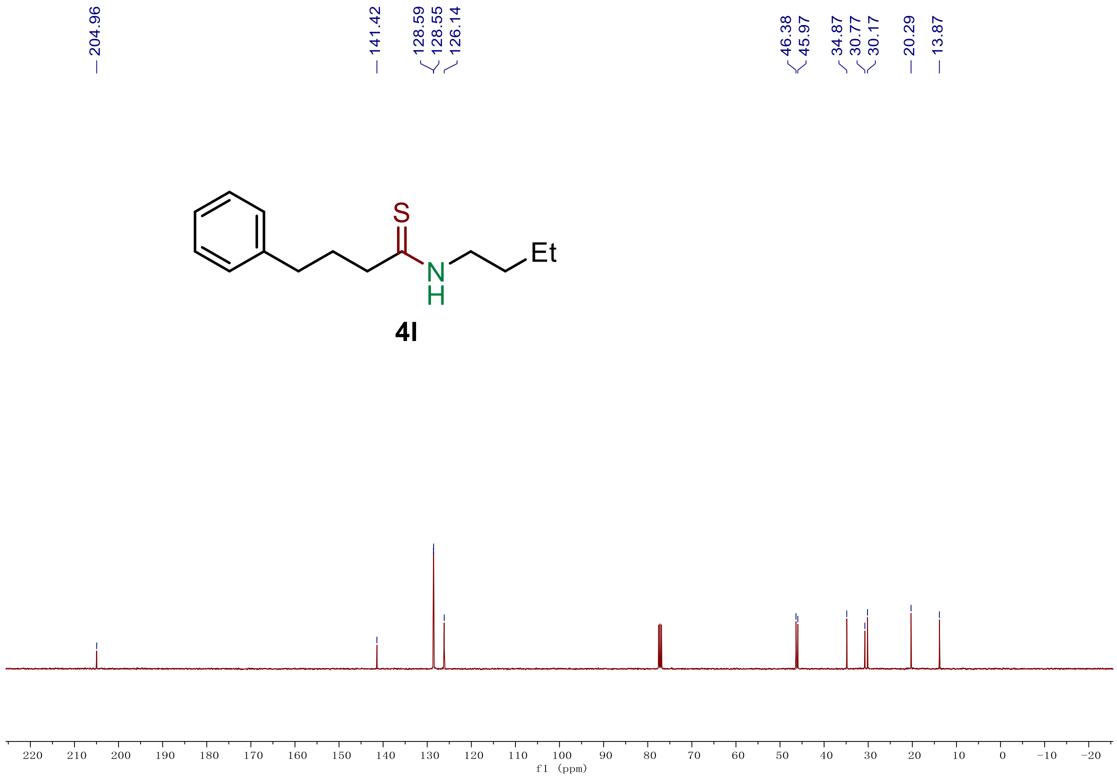

Supplementary Fig. S152 ^{13}C NMR spectrum of compound **4l** (101 MHz, $CDCl_3$)

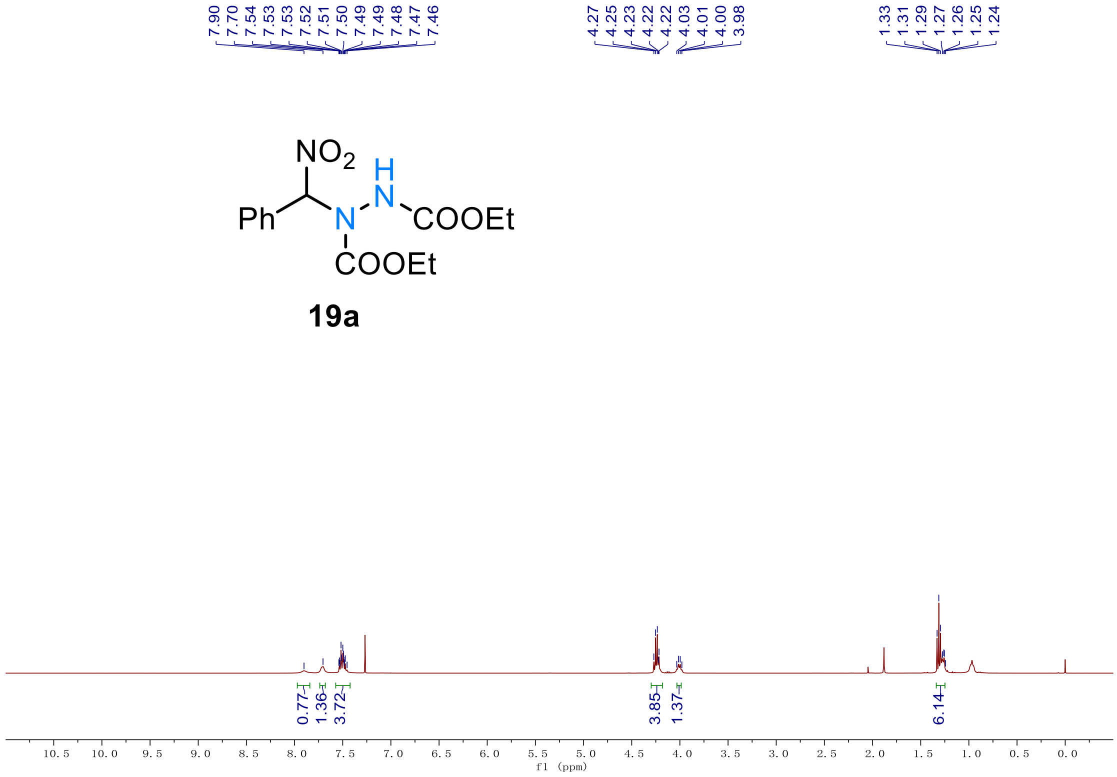

Supplementary Fig. S153 ^{1}H NMR spectrum of compound **19a** (400 MHz, $CDCl_3$)

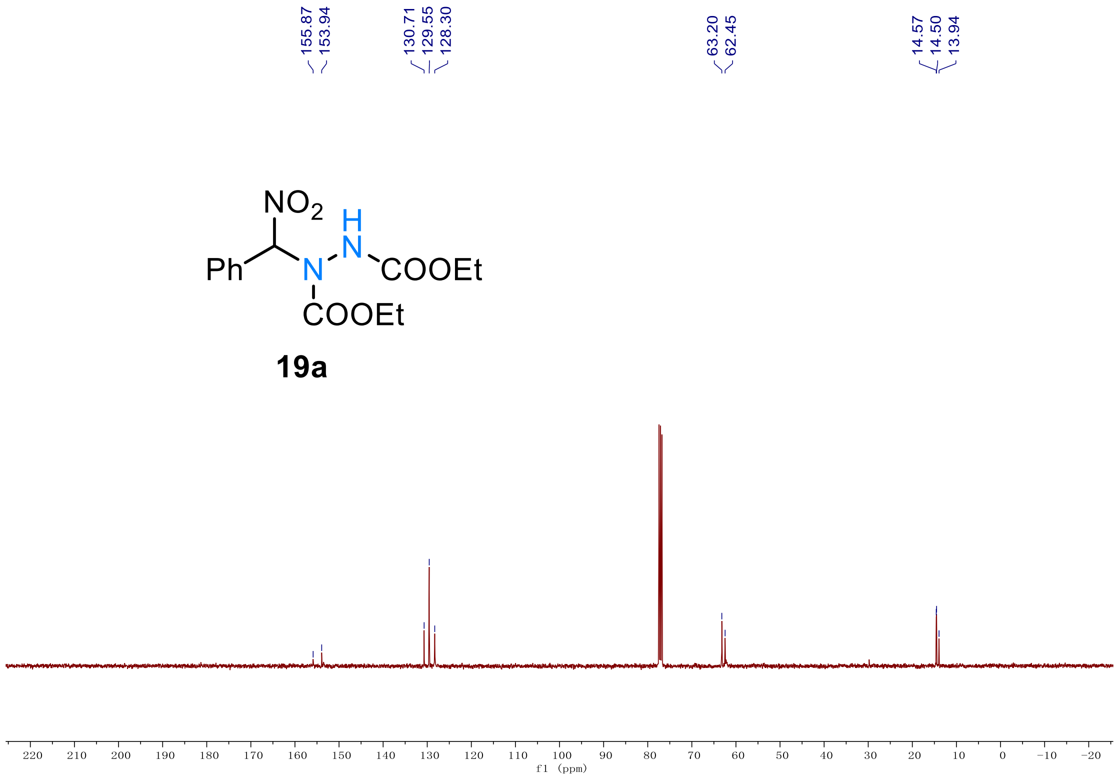

Supplementary Fig. S154 ^{13}C NMR spectrum of compound **19a** (101 MHz, $CDCl_3$)

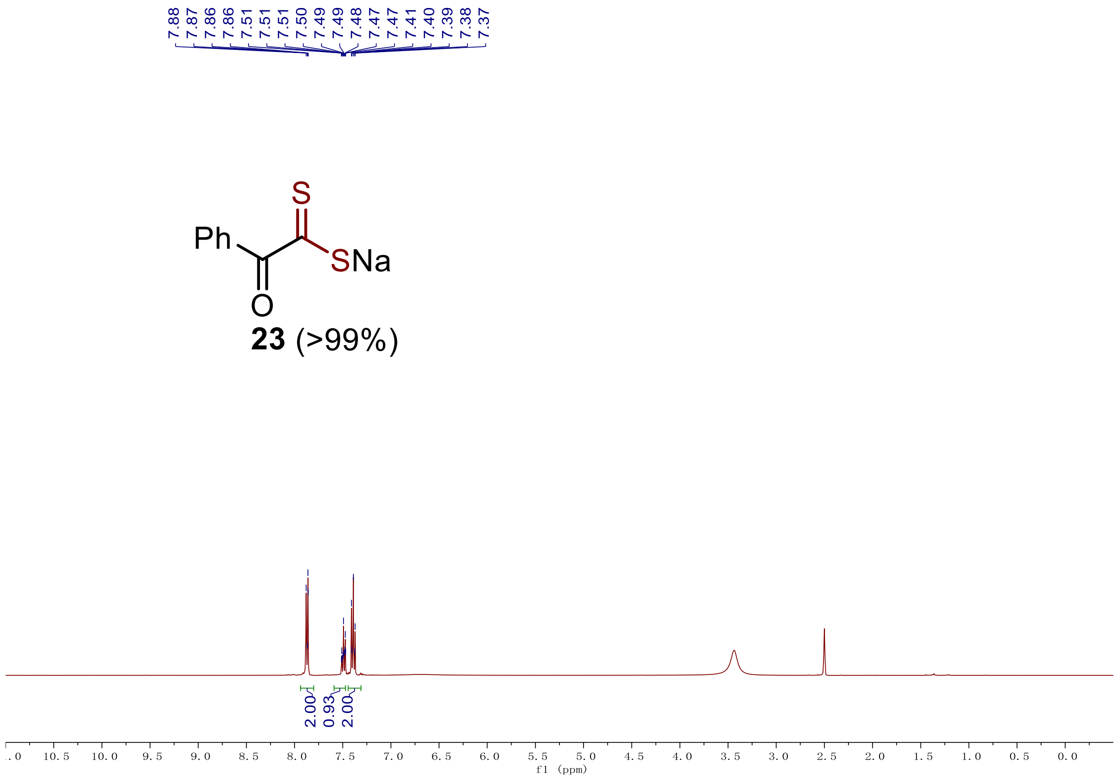

Supplementary Fig. S155 ^{1}H NMR spectrum of compound **23** (400 MHz, DMSO-d_6)

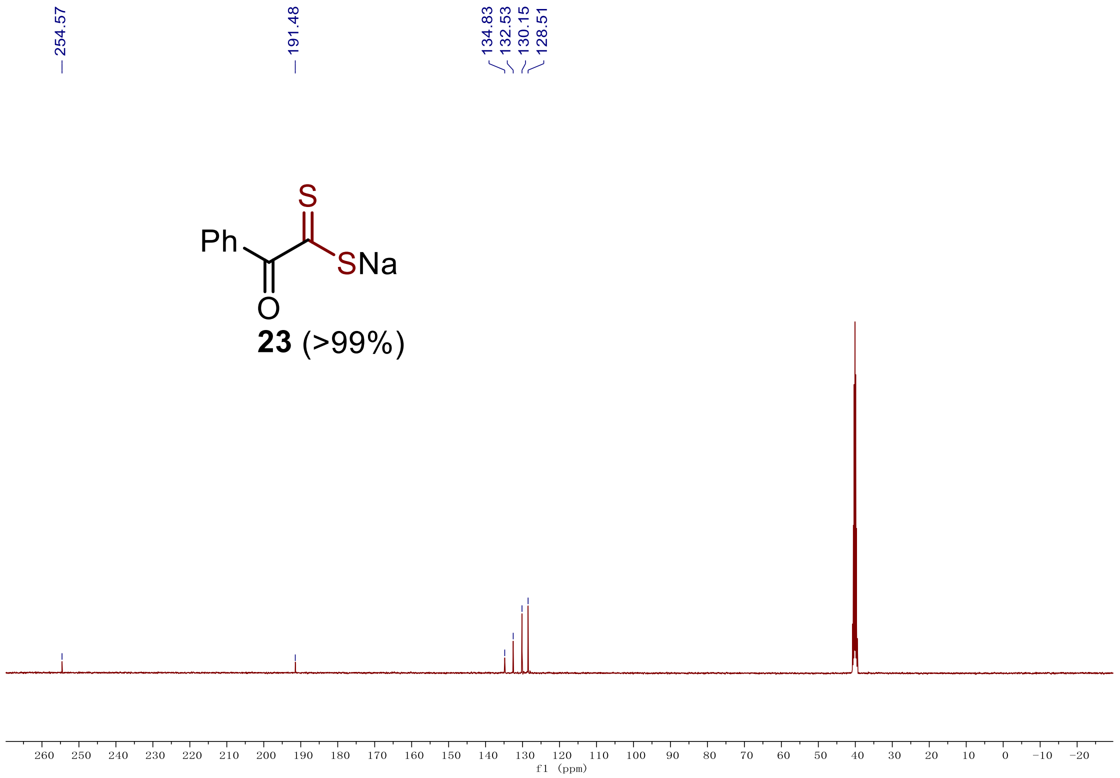

Supplementary Fig. S156 ^{13}C NMR spectrum of compound **23** (101 MHz, DMSO-d_6)

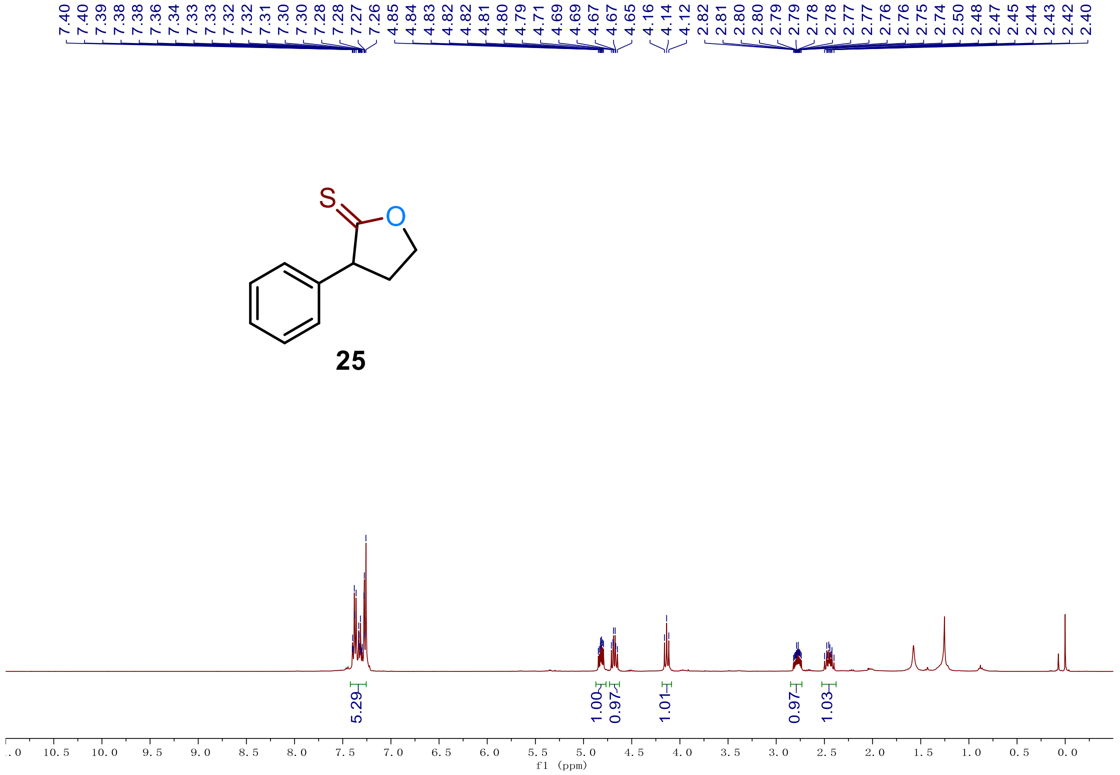

Supplementary Fig. S157 ^{1}H NMR spectrum of compound **25** (400 MHz, $CDCl_3$)

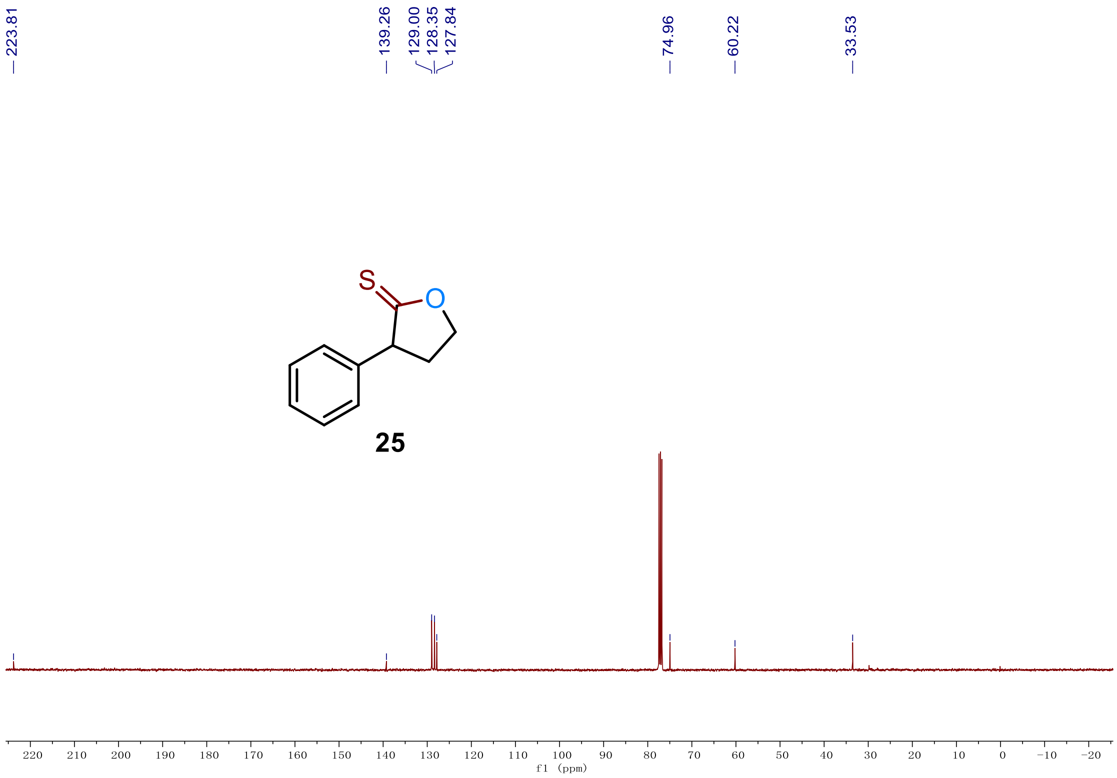

Supplementary Fig. S158 ^{13}C NMR spectrum of compound **25** (101 MHz, $CDCl_3$)

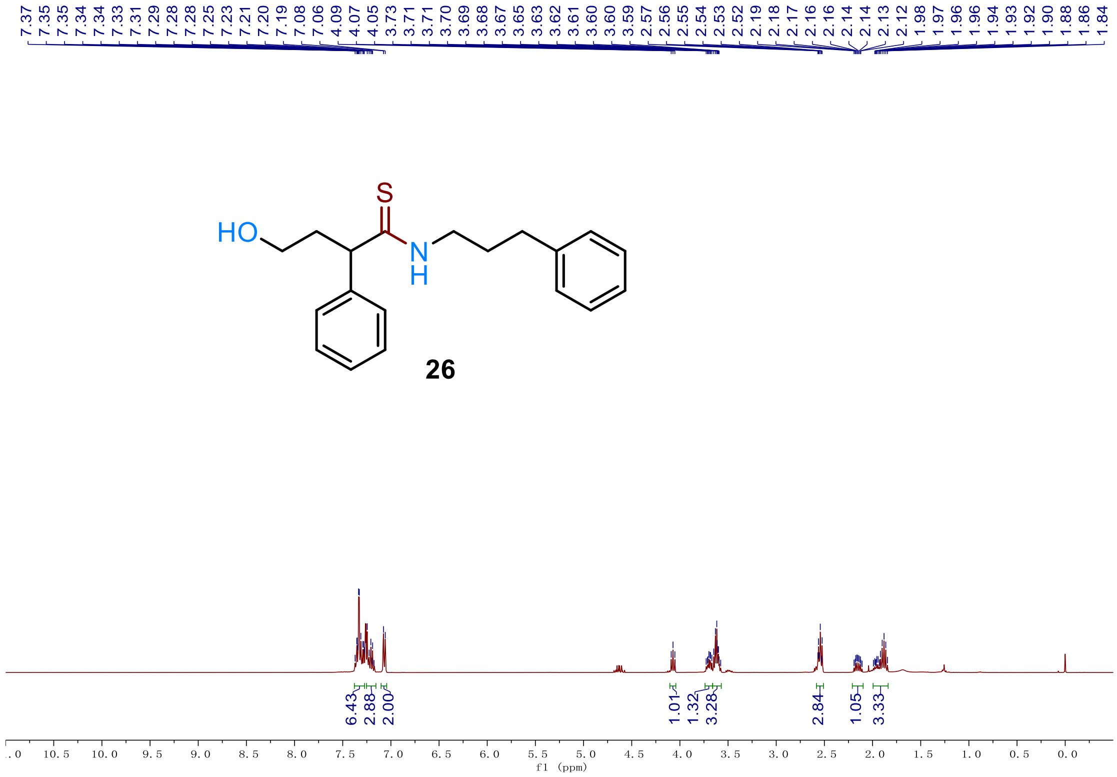

Supplementary Fig. S159 ^{1}H NMR spectrum of compound **26** (400 MHz, $CDCl_3$)

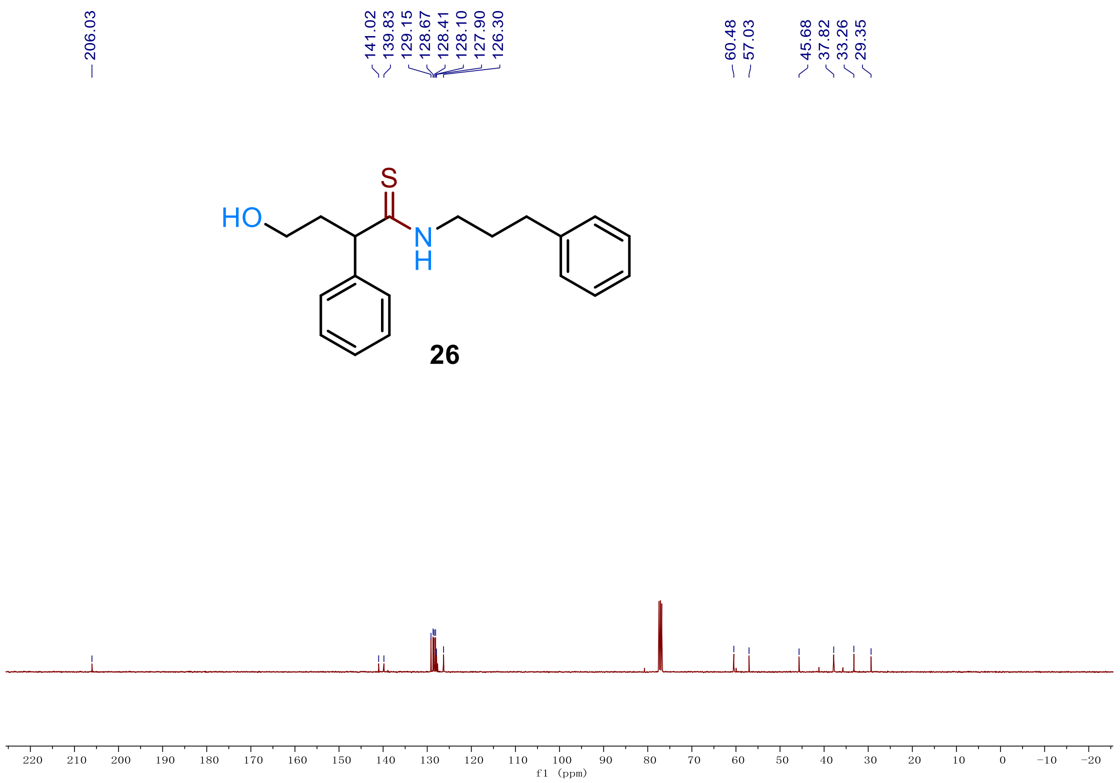

Supplementary Fig. S160 ^{13}C NMR spectrum of compound **26** (101 MHz, $CDCl_3$)

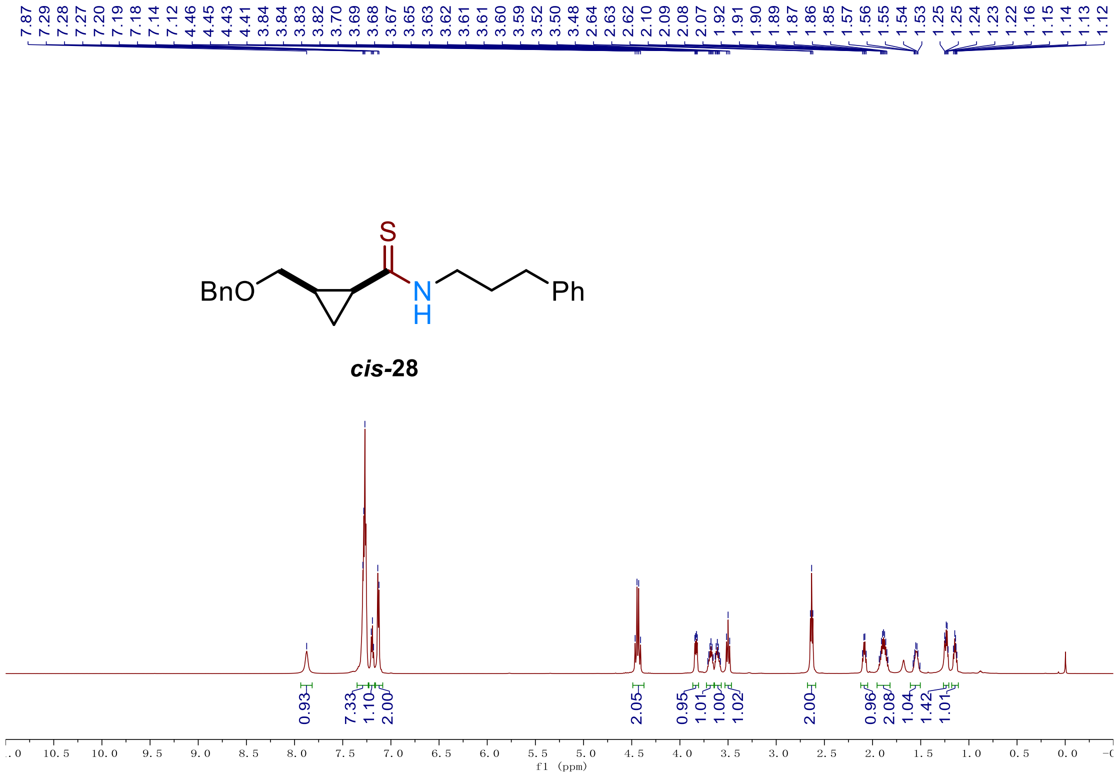

Supplementary Fig. S161 ^{1}H NMR spectrum of compound ***cis*-28** (400 MHz, $CDCl_3$)

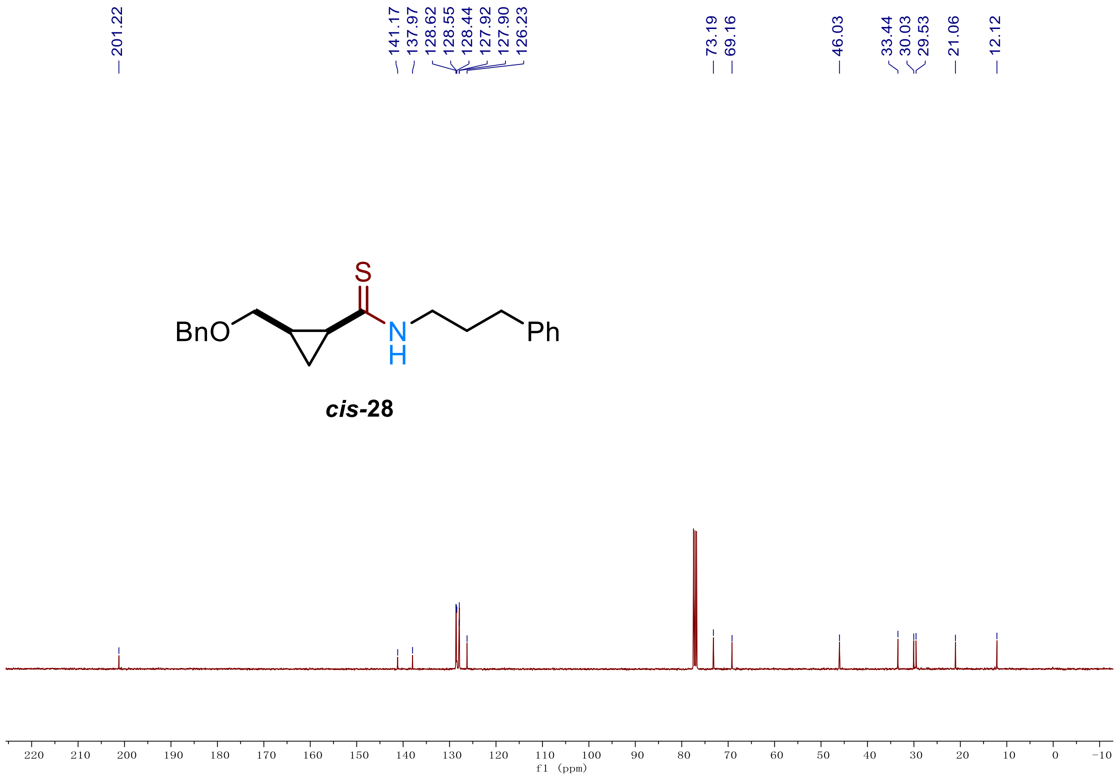

Supplementary Fig. S162 ^{13}C NMR spectrum of compound ***cis*-28** (101 MHz, $CDCl_3$)

4. Supplementary References

1. Gao, W.-C., Liu, J. & Jiang, X. Phthalimide-based-SSCF3 reagent for enantioselective dithiotrifluoromethylation. *Org. Chem. Front.* **8**, 1275–1279 (2021).
2. Ramaraju, P. *et al.* Synthesis and antimicrobial activities of structurally novel S,S′-bis(heterosubstituted) disulfides. *Bioorg. Med. Chem. Lett.* **22**, 3623–3631 (2012).
3. Li, J. *et al.* Oxidative Amidation of Nitroalkanes with Amine Nucleophiles using Molecular Oxygen and Iodine. *Angew. Chem. Int. Ed.* **54**, 12986–12990 (2015).
4. Chen, Y.-H., Sun, X.-L., Guan, H.-S. & Liu, Y.-K. Diversity-Oriented One-Pot Synthesis to Construct Functionalized Chroman-2-one Derivatives and Other Heterocyclic Compounds. *J. Org. Chem.* **82**, 4774–4783 (2017).
5. Clark, P. G. K. *et al.* LP99: Discovery and Synthesis of the First Selective BRD7/9 Bromodomain Inhibitor. *Angew. Chem. Int. Ed.* **54**, 6217–6221 (2015).
6. Kozikowski, A. P. Li, C. S. A Nitrile Oxide Based Entry to 2,3-Dihydropyran-4-ones. Synthesis of a Protected Version of “Compactin Lactone” in Racemic and Optically Active Forms. *J. Org. Chem.* **50**, 778–785 (1985).
7. Marcé, P., Lynch, J., Blacker, A. J. & Williams, J. M. J. Conversion of nitroalkanes into carboxylic acids via iodide catalysis in water. *Chem. Commun.* **52**, 1013–1016 (2016).
8. Richard W. Fitch, Gordon D. Sturgeon, Shaun R. Patel, Thomas F. Spande, H. Martin Garraffo, John W. Daly, and Richard H. Blaauw. A Nitrile Oxide Based Entry to 2,3-Dihydropyran-4-ones. Synthesis of a Protected Version of “Compactin Lactone” in Racemic and Optically Active Forms. *J. Nat. Prod.* **72**, 243–247 (2009).
9. Hostmann, T., Molloy, J. J., Bussmann, K. & Gilmour, R. Light-Enabled Enantiodivergence: Stereospecific Reduction of Activated Alkenes Using a Single Organocatalyst Enantiomer. *Org. Lett.* **21**, 10164–10168 (2019).

10. Massolo, E., Benaglia, M., Orlandi, M., Rossi, S. & Celentano, G. Enantioselective Organocatalytic Reduction of β-Trifluoromethyl Nitroalkenes: An Efficient Strategy for the Synthesis of Chiral β-Trifluoromethyl Amines. *Chem. Eur. J.* **21**, 3589–3595 (2015).
11. Fini, F. *et al.* Phase-Transfer-Catalyzed Asymmetric Aza-Henry Reaction Using N-Carbamoyl Imines Generated In Situ from α-Amido Sulfones. *Angew. Chem. Int. Ed.* **44**, 7975–7978 (2005).
12. Ferraro, A., Bernardi, L. & Fochi, M. Organocatalytic Enantioselective Transfer Hydrogenation of β-Amino Nitroolefins. *Adv. Synth. Catal.* **358**, 1561–1565 (2016).
13. Augustine, R. L. et al. Synthesis of .alpha.-monosubstituted indoles. *J. Org. Chem.* **38**, 3004–3011 (1973).
14. Zeng, C. *et al.* Rhodium-Catalyzed Generation of Anhydrous Hydrogen Iodide: An Effective Method for the Preparation of Iodoalkanes. *Org. Lett.* **20**, 6859–6862 (2018).
15. Toogood, H. S. *et al.* A Site-Saturated Mutagenesis Study of Pentaerythritol Tetranitrate Reductase Reveals that Residues 181 and 184 Influence Ligand Binding, Stereochemistry and Reactivity. *Chem. Bio. Chem.* **12**, 738–749 (2011).
16. Wang, B. *et al.* Asymmetric Phase-Transfer Catalysts Bearing Multiple Hydrogen-Bonding Donors: Highly Efficient Catalysts for Enantio- and Diastereoselective Nitro-Mannich Reaction of Amidosulfones. *Org. Lett.* **16**, 6432–6435 (2014).
17. Holmquist, M., Blay, G. & Pedro, J. R. Highly enantioselective aza-Henry reaction with isatin N-Boc ketimines. *Chem. Commun.* **50**, 9309–9312 (2014).
18. Burkhard, J. A., Tchitchanov, B. H. & Carreira, E. M. Cascade Formation of Isoxazoles: Facile Base-Mediated Rearrangement of Substituted Oxetanes. *Angew. Chem. Int. Ed.* **50**, 5379–5382 (2011).
19. Walvoord, R. R. & Kozlowski, M. C. Minimizing the Amount of Nitromethane in Palladium-Catalyzed Cross-Coupling with Aryl Halides. *J. Org. Chem.* **78**, 8859–8864 (2013).

20. Cao, M.-Y., Ma, B.-J., Gu, Q.-X., Fu, B. & Lu, H.-H. Concise Enantioselective Total Synthesis of Daphenylline Enabled by an Intramolecular Oxidative Dearomatization. *J. Am. Chem. Soc.* **144**, 5750–5755 (2022).
21. Lincke, T., Behnken, S., Ishida, K., Roth, M. & Hertweck, C. Closthioamide: An Unprecedented Polythioamide Antibiotic from the Strictly Anaerobic Bacterium Clostridium cellulolyticum. *Angew. Chem. Int. Ed.* **49**, 2011–2013 (2010).
22. Wang, C. *et al.* GPR52 Antagonist Reduces Huntingtin Levels and Ameliorates Huntington's Disease-Related Phenotypes. *J. Med. Chem.* **64**, 941–957 (2021).
23. Taniguchi, N. Copper-Catalyzed Synthesis of Sulfenamides Utilizing Diaryl Disulfides with Alkyl Amines. *Synlett* **2007**, 1917–1920 (2007).
24. Crocker, M. S. *et al.* Direct Observation and Analysis of the Halo-Amino-Nitro Alkane Functional Group. *Chem* **5**, 1248–1264 (2019).
25. Palomo, C. *et al.* Water-Compatible Iminium Activation: Organocatalytic Michael Reactions of Carbon-Centered Nucleophiles with Enals. *Angew. Chem. Int. Ed.* **46**, 8431–8435 (2007).
26. Umemiya, S., Nishino, K., Sato, I. & Hayashi, Y. Nef Reaction with Molecular Oxygen in the Absence of Metal Additives, and Mechanistic Insights. *Chem. Eur. J.* **20**, 15753–15759 (2014).
27. Zhang, G. et al. Trisulfur radical anion as the key intermediate for the synthesis of thiophene via the interaction between elemental sulfur and NaO^{t}Bu. *Org. Lett.* **16**, 6156–6159 (2014).
